# Network-based integration of molecular and physiological data elucidates regulatory mechanisms underlying adaptation to high-fat diet

**DOI:** 10.1007/s12263-015-0470-6

**Published:** 2015-05-28

**Authors:** Davina Derous, Thomas Kelder, Evert M. van Schothorst, Marjan van Erk, Anja Voigt, Susanne Klaus, Jaap Keijer, Marijana Radonjic

**Affiliations:** Microbiology and Systems Biology, TNO, Zeist, The Netherlands; Human and Animal Physiology, Wageningen University, Wageningen, The Netherlands; EdgeLeap B.V., Utrecht, The Netherlands; Group of Energy Metabolism, German Institute of Human Nutrition in Potsdam, Nuthetal, Germany; Institute of Biological and Environmental Sciences, University of Aberdeen, Aberdeen, Ab24 2TZ Scotland, UK

**Keywords:** Network analysis, Systems biology, Adipose tissue, High-fat diet, Data integration, Transcriptional regulation, Transcriptomics

## Abstract

Health is influenced by interplay of molecular, physiological and environmental factors. To effectively maintain health and prevent disease, health-relevant relations need to be understood at multiple levels of biological complexity. Network-based methods provide a powerful platform for integration and mining of data and knowledge characterizing different aspects of health. Previously, we have reported physiological and gene expression changes associated with adaptation of murine epididymal white adipose tissue (eWAT) to 5 days and 12 weeks of high-fat diet (HFD) and low-fat diet feeding (Voigt et al. in Mol Nutr Food Res 57:1423–1434, 2013. doi:10.1002/mnfr.201200671). In the current study, we apply network analysis on this dataset to comprehensively characterize mechanisms driving the short- and long-term adaptation of eWAT to HFD across multiple levels of complexity. We built a three-layered interaction network comprising enriched biological processes, their transcriptional regulators and associated changes in physiological parameters. The multi-layered network model reveals that early eWAT adaptation to HFD feeding involves major changes at a molecular level, including activation of TGF-β signalling pathway, immune and stress response and downregulation of mitochondrial functioning. Upon prolonged HFD intake, initial transcriptional response tails off, mitochondrial functioning is even further diminished, and in turn the relation between eWAT gene expression and physiological changes becomes more prominent. In particular, eWAT weight and total energy intake negatively correlate with cellular respiration process, revealing mitochondrial dysfunction as a hallmark of late eWAT adaptation to HFD. Apart from global understanding of the time-resolved adaptation to HFD, the multi-layered network model allows several novel mechanistic hypotheses to emerge: (1) early activation of TGF-β signalling as a trigger for structural and morphological changes in mitochondrial organization in eWAT, (2) modulation of cellular respiration as an intervention strategy to effectively deal with excess dietary fat and (3) discovery of putative intervention targets, such those in pathways related to appetite control. In conclusion, the generated network model comprehensively characterizes eWAT adaptation to high-fat diet, spanning from global aspects to mechanistic details. Being open to further exploration by the research community, it provides a resource of health-relevant interactions ready to be used in a broad range of research applications.

**Electronic supplementary material:**

The online version of this article (doi:10.1007/s12263-015-0470-6) contains supplementary material, which is available to authorized users.

## Introduction

Nutrition is an important health-influencing factor. Malnutrition, both in the form of insufficient or excess nutrient intake, is a significant disease risk factor (Must 1999; Bhaskaram 2002). In turn, a health-promoting dietary pattern is a powerful strategy for health maintenance and/or disease prevention (Hu 2002). To design optimal, evidence-based dietary strategies, it is of essence to comprehensively understand the effects of dietary interventions at a systems level. Network-based methods for data integration and mining are emerging as a powerful mean for uncovering complex relations between diet and relevant health aspects (Kelder et al. 2015). In addition to elucidating the complex effects of nutrients at molecular level, network-based integration of molecular and physiological evidence enables understanding of molecular mechanisms driving physiological effects and, ultimately, health and disease outcomes.

White adipose tissue (WAT) is particularly interesting as an organ relaying health outcomes in response to dietary interventions. It is constituted of depots distributed in different parts of the body (Cinti 2005). Its functionality to accumulate fat protects the body from toxic effects of lipids in other tissues, such as muscle or the liver. The overload of WAT capacity to store lipids results in continued low-grade inflammation and, ultimately, pathologies as associated with obesity, such as insulin resistance and type 2 diabetes (Owens 2014). In particular, central obesity (accumulation of visceral fat in humans and epididymal fat in rodents) plays a crucial role in the development of obesity-related disorders (Zimmet et al. 2005). To accommodate the accumulation of excess body fat, WAT expands by increasing adipocyte size (hypertrophy) and/or number (hyperplasia). Upon prolonged high-fat diet (HFD) feeding, adaptive mechanisms exceed their capacity and metabolic dysfunction becomes evident at a physiological level (e.g. insulin resistance, increased plasma cholesterol, and triglyceride levels) (Hill et al. 1992).

To prevent late-stage metabolic dysfunctions caused by excess lipids, it is necessary to understand the regulation of metabolic processes triggered at early stage, before physiological changes occur (Palou et al. 2004). We have previously studied time-resolved, HFD-induced epididymal white adipose tissue (eWAT) gene expression changes in a mouse model (Caesar et al. 2010). Nevertheless, the short- and long-term exposure to HFD has not yet been analysed by bridging molecular regulation to physiological changes in a single model. Emerging network-based methods now allow us to consider such multi-level relations in a comprehensive manner, departing from independent analysis of different assay readouts.

Previously, we have reported physiological and eWAT gene expression changes associated with adaptation to HFD feeding at 5 days and 12 weeks (Voigt et al. 2013). While this earlier study focused only on gene and pathway changes common between early and late timepoints, here, we extract additional value out of this dataset by studying relations between molecular and physiological changes in a single network model and in a time-resolved manner. This enables discovery of regulatory molecular mechanisms associated with, and possibly driving the observed physiological effects during both early and late adaptation to HFD. To achieve this, we have built an integrative, three-layered network model, comprising biological processes, transcriptional regulators and physiological readouts at 5 days and 12 weeks of HFD feeding. The model captures major regulatory mechanisms underlying adaptation to HFD in eWAT and discovers novel insights into physiological effects of short- and long-term HFD feeding. In addition, the resulting network representation enables further exploration of the data towards studies focusing on of molecular interactions relevant for HFD response and eWAT health in general.

## Results

### Network analysis of biological processes underlying eWAT adaptation to high-fat diet

**Fig. 1:**
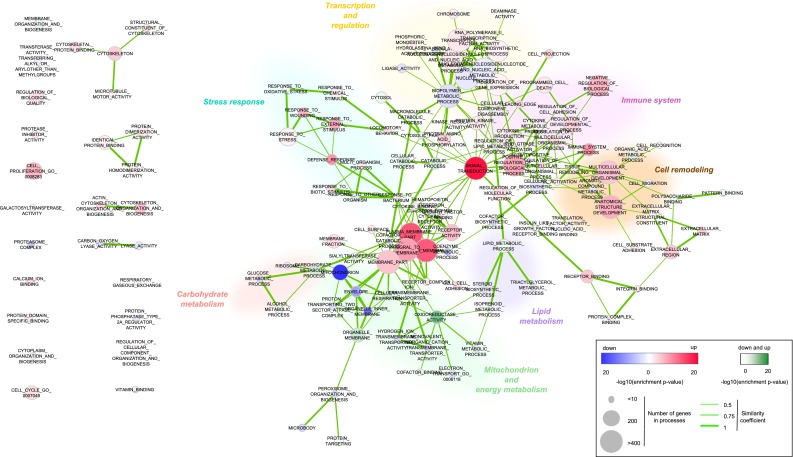
Network of biological processes after 5 days of HFD. Differentially enriched biological processes (HFD vs. LFD) after 5 days of HFD feeding are analysed using Enrichment map (Cytoscape). The nodes represent biological processes, and edges represent overlap between genes in the enriched processes. The colour of the nodes represents the significance and the direction of the expression (*blue*—downregulation; *red*—upregulation; *green*—both up- and downregulation). The size of the nodes corresponds to the size of the gene set. The width of edges is based on similarity coefficients between the nodes, derived from the overlap of the gene set underlying the processes. Related biological processes were grouped into seven main clusters

**Fig. 2:**
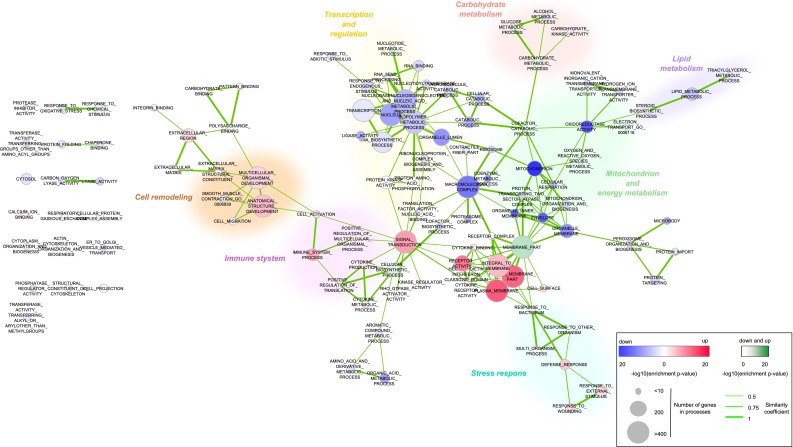
Network of biological processes after 12 weeks of HFD feeding. Similar as in Fig. 1, for timepoint 12 weeks

In a previous study, we have established that eWAT gene expression changes that occur after 5 days of HFD feeding are predictive of changes seen after 12 weeks of HFD (Voigt et al. 2013). Here we apply network analysis to further interpret these changes and to investigate the relations between the pathways involved during both early and late adaptation to HFD feeding. The gene expression changes (HFD vs. LFD) at 5 days and at 12 weeks were used as an input for Gene Set Enrichment Analysis (Subramanian et al. 2005) and Enrichment Map (Merico et al. 2010) (Supplemental data 1, Supplemental data 2). The resulting network is comprised of nodes representing enriched biological processes, connected by edges representing overlap between genes in the enriched processes (Figs. 1, 2; Supplemental data 3, Supplemental data 4). To guide visual interpretation of emerging patterns in the network, related biological processes were manually grouped based on the combination of topology and function into the following main clusters: “stress response”, “immune response”, “cell remodelling”, “transcription and regulation”, “lipid metabolism”, “carbohydrate metabolism” and “mitochondrion and energy metabolism”.

The most central biological process in network at 5 days is “signal transduction”, linking to multiple nodes in “immune system” and “stress response” clusters (Fig. 1). The majority of processes within the “immune system”, “stress response” and “cell remodelling” clusters are upregulated in HFD, whereas most of processes within “mitochondrion and energy metabolism” cluster are downregulated. Biological process “mitochondrion” shows most prominent downregulation in the 5-day network (FDR *p* value 2.03E−34). The cluster “transcription and regulation” is comprised of both up- and downregulated processes, suggesting complex regulation of gene expression during early response to HFD.

The network topology at 12 weeks shows higher clustering compared to 5-day network and a more consistent direction of expression changes among processes within each cluster (Fig. 2; Supplemental table 1, Supplemental data 2). The “signal transduction” process remains the node with highest betweenness centrality, but other highly central nodes appear (e.g. “macromolecular complex” and “membrane part”). The majority of clusters remain regulated in the same direction as at 5 days, except for cluster “transcription and regulation” which is completely downregulated at 12 weeks. Again, the most striking observation is the strong, even more prominent than at 5 days, downregulation of the process “mitochondrion” (FDR *p* value 2.21E−48). Also other processes in the “mitochondrion and energy metabolism” cluster show similar trend, suggesting deterioration of mitochondrial functions as a major hallmark of prolonged HFD intake.

### Multi-level network model of eWAT adaptation to high-fat diet

**Fig. 3:**
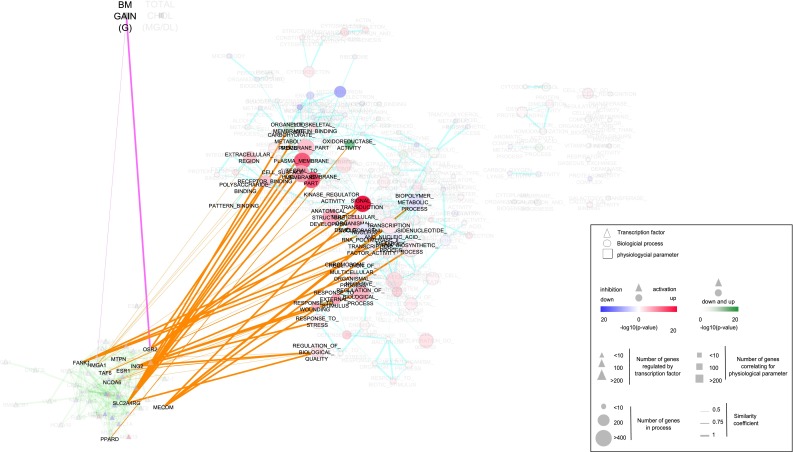
Multi-level network model of eWAT adaptation to 5 days of HFD. The three-layered network model comprising (1) biological processes, (2) transcription regulators (TFs) and (3) physiological parameters associated with eWAT gene expression after 5 days of HFD feeding. The processes layer includes differentially enriched biological processes as described in Fig. 1. The regulatory network layer includes TFs whose targets are enriched among the differentially expressed genes (HFD vs. LFD after 5 days of HFD feeding). TFs with highly overlapping target gene sets are clustered into single nodes. The physiological network layer includes parameters significantly correlated with eWAT expression data. The connections between the three layers are based on the overlap between underlying gene sets. The width of edges is based on the overlap between underlying gene sets. The colour coding of nodes is as described in Fig. 1, where for TFs, the direction of the expression of their targets is represented (*red*—activation, *blue*—repression)

**Fig. 4:**
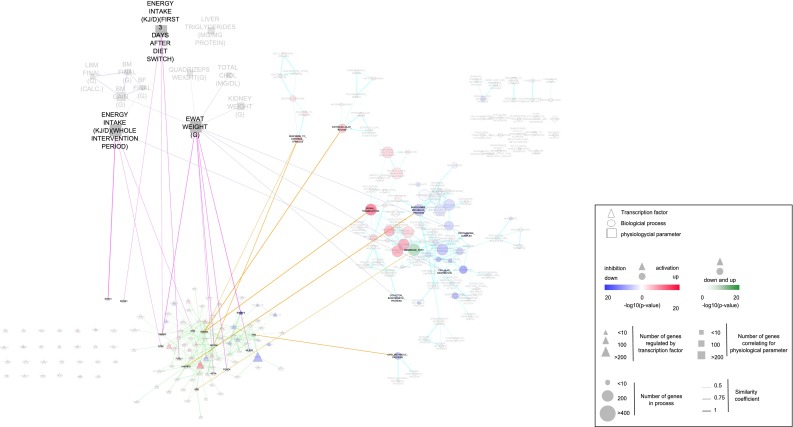
Multi-level network model of eWAT adaptation to 12 weeks of HFD. Similar to Fig. 3, for timepoint 12 weeks. The dashed lines indicate a different use of cut-off for the overlap coefficient (see “Methods” section)

Network analysis of biological processes associated with eWAT adaptation to HFD elucidates their mutual interconnectivity and changes during transition from early to late response. To place these processes in a physiological, systems context and investigate their regulation, we have built a three-layered network model comprising (1) biological processes, (2) transcription regulators and (3) physiological parameters (Figs. 3, 4; Supplemental data 8, Supplemental data 9). The connections between the three layers are based on overlap between underlying gene sets (“Methods” section).

The biological processes layer was generated as described above. The regulatory network layer included transcription factors whose targets are enriched among the differentially expressed genes. In total, 105 and 120 transcription factors are identified as regulators of gene expression changes at 5 days and 12 weeks, respectively (Supplemental data 5). Transcription factors with highly overlapping target gene sets are clustered, resulting in 52 and 111 transcription regulators used as nodes in the network (Supplemental table 2). The third physiological network layer was generated by correlation analysis between changes in physiological parameters and eWAT gene expression. This identified two (“body mass (BM) gain” and “total cholesterol”) and 11 (“body mass (BM) final”, “body mass (BM) gain”, “body fat (BF) final”, “lean body mass (LBM) final”, “eWAT weight”, “energy intake (3 days after diet switch)”, “energy intake (whole intervention period)”, “kidney weight”, “quadriceps weight”, “liver triglycerides”, “total cholesterol”) significantly correlated physiological parameters at 5 days and 12 weeks, respectively (Supplemental data 6).

The connections between three layers reveal differences in organization of the multi-level network model at 5 days and 12 weeks. Namely, at the early timepoint, the only linked physiological parameter (“body mass (BM) gain”) connects exclusively to the transcription regulators layer and there is a high density of links between transcription regulators and biological processes. In contrast, at the late timepoint, physiological parameters (“eWAT weight”, “energy intake (3 days after diet switch)” and “energy intake (whole intervention period)”) connect to both transcription regulators and biological processes, whereas the links between the latter two layers become sparse.

### Regulatory mechanisms associated with physiological adaptation to high-fat diet

The three-layered network model provides a resource of associations that can be further mined for mechanisms of interest (Table 1, Supplemental data 7). Here, we focus on regulatory mechanisms in eWAT that drive observed systems physiological changes.

**Table 1:** Connections between three layers (processes–transcription factors (TF)–physiological parameters) for 5 days and 12 weeks. The relationship indicates between which layers the edge occurs, and the size of overlap shows the amount of genes overlapping. Similarly, coefficient is a measurement of similarity between the two gene set of the two nodes connected by an edge

Edge ID	Relationship	Size of overlap	Similarity coefficient
*5* *days*			
BM gain—HMGA1	Physiology–TF	1	0.5
BM gain—OSR2	Physiology–TF	1	1
ESR1—regulation_of_biological_quality	TF–processes	3	0.6
FANK 1—Chromosome	TF–processes	1	1
FANK1—nucleus	TF–processes	1	1
FANK1—RNA_polymerase_II_transcription_factor_activity	TF–processes	1	1
HMGA1—cytoskeletal_protein_binding	TF–processes	1	0.5
HMGA1—extracellular_region	TF–processes	1	0.5
HMGA1—kinase_regulator_activity	TF–processes	1	0.5
HMGA1—pattern_binding	TF–processes	1	0.5
HMGA1—polysaccharide_binding	TF–processes	1	0.5
HMGA1—signal_transduction	TF–processes	1	0.5
ING2—negative_regulation_of_biological_process	TF–processes	1	1
ING2—regulation_of_biological_quality	TF–processes	1	1
ING2—regulation_of_multicellular_organismal_process	TF–processes	1	1
ING2—response_to_external_stimulus	TF–processes	1	1
ING2—response_to_stress	TF–processes	1	1
ING2—response_to_wounding	TF–processes	1	1
MECOM—biopolymer_metabolic_process	TF–processes	1	1
MECOM—nucleobasenucleosidenucleotide_and_nucleic_acid_metabolic_process	TF–processes	1	1
MECOM—RNA_biosynthetic_process	TF–processes	1	1
MECOM—signal_transduction	TF–processes	1	1
MECOM—transcription	TF–processes	1	1
MTPN—regulation_of_biological_quality	TF–processes	2	0.5
NCOA6—negative_regulation_of_biological_process	TF–processes	1	0.5
NCOA6—regulation_of_biological_quality	TF–processes	1	0.5
NCOA6—response_to_external_stimulus	TF–processes	1	0.5
NCOA6—response_to_stress	TF–processes	1	0.5
NCOA6—response_to_wounding	TF–processes	1	0.5
ORS2—receptor_binding	TF–processes	1	1
OSR2—anatomical_structure_development	TF–processes	1	1
OSR2—multicellular_organismal_development	TF–processes	1	1
OSR2—signal_transduction	TF–processes	1	1
PPARD—oxidoreductase_activity	TF–processes	2	0.666666667
SLC2A4RG—carbohydrate_metabolic_process	TF–processes	1	1
SLC2A4RG—cell_surface	TF–processes	1	1
SLC2A4RG—integral_to_membrane	TF–processes	1	1
SLC2A4RG—membrane_part	TF–processes	1	1
SLC2A4RG—organelle_membrane	TF–processes	1	1
SLC2A4RG—plasma_membrane	TF–processes	1	1
SLC2A4RG—plasma_membrane_part	TF–processes	1	1
SLC2A4RG—regulation_of_biological_quality	TF–processes	1	1
TAF6—chromosome	TF–processes	1	0.5
TAF6—nucleus	TF–processes	1	0.5
TAF6—RNA_polymerase_II_transcription_factor_activity	TF–processes	1	0.5
NCOA6—regulation_of_multicellular_organismal_process	TF–processes	1	0.5
*12* *weeks*			0.4
Energy intake (KJ/D)(whole intervention period)—cellular_respiration	Physiology–processes	6	0.333333333
eWAT weight (g)—cellular_respiration	Physiology–processes	5	0.315789474
eWAT weight (g)—cofactor_biosynthetic_process	Physiology–processes	6	0.388888889
eWAT weight (g)—proteasome_complex	Physiology–processes	7	0.5
ETS2—energy intake (KJ/D)(whole intervention period)	TF–physiology	7	0.571428571
FOSL1—energy intake (KJ/D)(first 3 days after diet switch)	TF–physiology	8	0.615384615
FOXO4—eWAT weight (g)	TF–physiology	8	0.583333333
MLXIPL—eWAT weight (g)	TF–physiology	7	0.6
NCOA6—eWAT weight (g)	TF–physiology	3	0.5
NFYA—eWAT weight (g)	TF–physiology	8	0.5
PDE—energy intake (KJ/D)(whole intervention period)	TF–physiology	2	0.5
RCOR1—energy intake (KJ/D)(first 3 days after diet switch)	TF–physiology	1	0.5
SMAD5—energy intake (KJ/D)(first 3 days after diet switch)	TF–physiology	3	0.666666667
SMAD5—eWAT weight (g)	TF–physiology	4	0.5
SOX10—energy intake (KJ/D)(whole intervention period)	TF–physiology	4	0.53125
SREBF2—eWAT weight (g)	TF–physiology	17	0.5
DEK—membrane_part	TF–processes	2	0.625
FXR—lipid_metabolic_process	TF–processes	5	0.666666667
HNRNPD—biopolymer_metabolic_process	TF–processes	2	0.666666667
KDM3A—extracellular_region	TF–processes	4	0.5
KDM3A—response_to_external_stimulus	TF–processes	3	0.666666667
KDM3A—signal_transduction	TF–processes	4	0.6
NCOA6—response_to_external_stimulus	TF–processes	3	0.4

At the 5-day timepoint, we identified “body mass (BM) gain” as physiological parameter linked to transcriptional regulators in eWAT, namely OSR2 and HMGA1 (Fig. 3; Table 1). The target genes of OSR2 and HMGA1 underlying this association (transforming growth factor, beta 3 (*Tgfb3*) and follistatin-like 1 (*Fstl1*, also known as Tgfb-inducible protein TSC-36), respectively) both positively correlated with BM gain, suggesting that BM gain occurring during the early adaptation to HFD may be mechanistically linked to activation of transforming growth factor beta (TGF-β) signalling pathway. Consistent with this hypothesis, both OSR2 and HMGA1 are linked to “signal transduction” node within biological processes layer, which comprises TGF-β pathway. In addition, another transcription regulator involved in TGF-β signalling (MDS1 and EVI1 complex locus (encoded by *Mecom*)) has been identified at 5-day timepoint, although without direct link to BM gain.

At the 12-week timepoint, physiological parameters “eWAT weight”, “energy intake (3 days after diet switch)” and “energy intake (whole intervention period)” are linked to molecular changes in eWAT, on both transcription regulators and biological processes levels. Identified transcription factors linking to “eWAT weight” (SREBF2, MLXIPL, FOXO4, NFYA, SMAD5 and NCOA6) are involved in lipid and cholesterol metabolism, mitochondrial dysfunction, apoptosis or cell survival and TGF-β signalling pathway. On the level of biological processes, “eWAT weight” links to “cellular respiration”, “cofactor biosynthetic process” and “proteasome complex”. The six genes underlying association with “cellular respiration” are all part of the mitochondrial electron transport chain complex and are negatively correlated with eWAT weight.

The physiological parameter “energy intake (whole intervention period)” also linked to biological process “cellular respiration” via negatively correlated mitochondrial electron transport chain genes, and to transcription factors ETS2, PDE and SOX10. The genes underlying these transcription factor relations are largely involved in oxidative stress, differentiation and development, apoptosis and cholesterol metabolism. Interestingly, parameter “energy intake (3 days after diet switch)” linked to a different set of transcription factors (FOSL1, RCOR1 and SMAD5), suggesting different regulatory mechanisms of this physiological aspect at short- and long-term HFD feeding period.

In all, key regulatory mechanisms occurring in eWAT during HFD intake involve TGF-β signalling mediated processes associated with BM gain (early phase) and shutdown of cellular respiration associated with increase in eWAT weight and energy intake (late phase).

## Discussion

Network-based analysis approaches are well suited for study of complex phenotypes, as they enable insight into relations between different layers of biological complexity and comprehension of a system as a whole (Barabási et al. 2011). Here, we have built a multi-level network model of eWAT adaptation to high-fat diet (HFD) to elucidate regulatory mechanisms driving physiological effects associated with excess fat intake in mice. Our network model spans across three biological complexity levels: biological processes, transcriptional regulators and physiological readouts, and is built to represent early (5 days) and late (12 weeks) phase of adaptation to HFD feeding. In addition to experimental data, the model incorporates current knowledge, i.e. pathway information and transcription factors targets. Such comprehensive view of the HFD response allows discovery of regulatory mechanisms underlying short- and long-term HFD feeding and uncovers novel relations between molecular changes in eWAT and systems physiological effects.

Our work builds upon a previously published dataset, which focused on the predictive aspect of short-term gene expression changes for long-term effects of high-fat feeding. Here we extract additional value out of existing data by integrating data across assays, data types and levels of biological complexity. By breaking the silos among diverse types of data and information, we here accomplish a more complete understanding of the system and its time-resolved adaptation to HFD, allowing several previously unanticipated hypotheses to emerge.

### Short-term adaptation to high-fat diet

The switch to HFD triggers an adaptive response that requires metabolic changes and cell remodelling of eWAT, accompanied by stress response and inflammation. Such abrupt and massive changes in cellular functions are relayed by extensive activation of signal transduction pathways and transcription regulators. On a metabolic level, adaption in handling energy metabolism occurs, both in the management of energy resources (lipid and carbohydrate metabolism) and in the energy expenditure (strong downregulation of mitochondrial function). Nevertheless, observed molecular changes do not yet have broad relations to physiological changes, suggesting that early response of eWAT to HFD primarily involves cellular adaptation.

The only physiological parameter that can be linked to early transcription regulation in eWAT is body mass gain. Molecules underlying this association may therefore be considered as putative eWAT markers of susceptibility to increased body mass gain in individual animals. Activation of TGF-β signalling pathway emerges as the major determinant of this phenotype, providing clues for further biomarker and therapeutics research. In this context, *Tgfb3* and *Fstl1* may especially be of interest for follow-up studies. Considering the role of TGF-β signalling, it is plausible that its early activation sets in motion structural and morphological changes in eWAT, possibly extending to alterations in mitochondrial organization. Namely, shutdown of mitochondrial and cellular respiration genes (triggered at 5 days and aggravated at 12 weeks) may result from reduced density of functional mitochondrial units, possibly mediated by TGF-β signalling (Krick et al. 2008; Casalena et al. 2012).

### Long-term adaptation to high-fat diet

After 12 weeks of HFD feeding, eWAT has reached a more settled state. This is reflected in overall network model topology, downplay of signalling transduction (FDR *p* value of enrichment changes from of 2.76E−20 to 1.35E−09) and particularly in the shutdown of transcription regulation. Importantly, the link between eWAT molecular mechanisms and systems physiological effects becomes evident: 11 systems physiology parameters are significantly correlated with eWAT gene expression at 12 weeks. The eWAT effects on physiology are strongly linked to shutdown of cellular respiration, in line with severe downregulation of mitochondrial function (FDR *p* value of enrichment drops from 2.03E−34 to 2.21E−48).

In all, the architecture of the three-layered network model suggests that early adaptation to HFD mainly involves triggering of a series of transcriptional regulatory events in eWAT, while at the late phase, this initial cellular response tails off and the relation between eWAT gene expression and physiological changes becomes more prominent.

### Translational relevance

Our model shows that mice with lower capacity for energy handling through cellular respiration also have higher overall energy intake and eWAT weight. Although the causality of these aspects is yet to be determined, we hypothesize that dysfunctional cellular respiration may contribute to high eWAT weight and to increased need for energy intake. If so, this finding may be relevant for diagnosis of subjects having these mechanisms compromised, possibly predisposing them to overconsumption on a long term. The ability to detect such dysfunction early (e.g. via biomarkers) may be used as a basis for patient stratification into groups with different susceptibility to obesity. The molecular-level understanding of regulation of these processes may in turn point to intervention strategies to compensate for insufficiency of intrinsic mechanisms to effectively deal with excess fat diets. To this end, transcription factors associated with eWAT weight and energy intake may be good candidates for thorough mechanistic follow-up studies.

To underpin mechanisms underlying predisposition to overconsumption, it may also be of interest to investigate genes correlated with the short-term energy intake (i.e. physiological parameter “energy intake (first 3 days after diet switch)”) in individual mice. Namely, mice in this study have been fed *ad libitum*, and the switch to HFD led to an initial overconsumption, i.e. on average higher levels of daily energy intake in the 5-day group (Voigt et al. 2013). Regulation and mechanisms associated with short-term energy intake may shed light on motivational aspects leading to early onset of overeating behaviour. For instance, the gene underlying the link between short-term energy intake and transcription factor RCOR1 is *Chrm4* (cholinergic receptor, muscarinic 4; correlation coefficient 0.83, *p* value 0.01). CHRM4 is a member of the muscarinic acetylcholine receptor family (M1–M5), and one of its family members (M3) is involved in regulation of food intake, body weight and peripheral fat deposits (Yamada et al. 2001; Serby et al. 2006). It would be interesting to test whether CHRM4 as well directly influences appetite control in our experimental setting.

In all, our multi-level network model comprehensively characterizes eWAT adaptation to high-fat diet (HFD), spanning from global aspects to mechanistic details. The highlighted insights emerging from the model provide promising leads to new research avenues: (1) early activation of TGF-β signalling as a trigger for structural and morphological changes in mitochondrial organization in eWAT, (2) modulation of cellular respiration as an intervention strategy to effectively deal with excess dietary fat and (3) putative role of CHRM4 in appetite control towards discovery of novel intervention targets. Apart from identified research leads, the network model is now open to be further explored by the broad research community, thereby providing a sustainable resource of molecular interactions relevant for HFD response and eWAT health in general.

## Methods

### Data resources

All microarray gene expression data and physiological data used in this study have been previously published (Voigt et al. 2013) and deposited at the Gene Expression Omnibus database (GSE38337).

### Experimental design and diets

Experimental design and diets have been previously described in Voigt et al. (2013). Briefly, male C57BL/6JRccHsd mice, 4 weeks old upon arrival, were fed a standard CHOW diet during an adaptation period of 2 weeks. Thereafter, mice were stratified by body weight and assigned into four experimental groups, being fed *ad libitum* either a semisynthetic standard low-fat diet (LFD) or a semisynthetic experimental high-fat diet (HFD) for either 5 days (*n* = 10/per diet) or 12 weeks (*n* = 12/diet). After 5 days and 12 weeks, respectively, mice were killed, and plasma and epididymal white adipose tissue (eWAT) samples were obtained. Microarray analyses were performed using 4 × 44 k Agilent whole-mouse genome microarray platform (GSE38337).

### Statistical analysis of microarray data

Statistical analysis of microarray data was performed via online available standardized array analysis program ArrayAnalysis (http://www.arrayanalysis.org/new/) using the Illumina (limma) module (Eijssen et al. 2013) (Eijssen et al., accepted for publication). Via this pipeline, *p* values, adjusted *p* values (FDR) and *t* scores were obtained for pairwise comparisons of HFD and LFD groups, at 5-day and 12-week timepoints (Supplementary data 1).

### Biological processes enrichment analyses

Enriched biological processes in HFD group compared to LFD were identified using gene set enrichment analysis (GSEA) (C5: GO gene set, biological processes) (Subramanian et al. 2005). A list of genes and *t* scores obtained by limma statistical analysis were used to generate enrichment scores of processes (Supplemental data 2). Gene sets smaller than 15 and bigger than 500 were excluded from further analyses. The obtained enriched *p* values were log-transformed (−log10) and adjusted for direction based on their significance in up- or downregulation (either up-, down-, or both up- and downregulated) to derive colour codes for nodes in network visualizations. Full GSEA output files containing genes associated with specific biological processes and their rank score representing extent of modulation are provided as Supplemental data 10.

### Transcription factor target analysis

A list of differentially expressed genes (HFD vs. LFD per timepoint, FDR < 0.05) was loaded into Ingenuity Pathway Analysis (IPA) (Ingenuity^®^ Systems, www.ingenuity.com) to identify upstream regulators (transcription factors (TFs)) and corresponding target genes (Supplemental data 5).

TF activation scores were obtained by log-transforming (−log10) the *p* values and adjusting for their *Z* score sign. Because of high overlap between the regulated TF targets as reported by Ingenuity, the TFs were clustered to group TFs with similar target genes. Transcription factors with 100 % overlap between their target genes were clustered. The transcription factor with highest significance was taken as cluster representative (Supplemental Table 2).

### Correlation analysis of gene expression and physiological parameters

Correlations between the physiological parameters and transcriptome data were calculated per individual mouse using Spearman’s rho (Supplemental data 6). An absolute correlation coefficient >0.8 combined with a *p* value < 0.05 was used as a significance cut-off.

### Building of the network model

A three-layered bipartite network model was generated comprising (1) biological processes, (2) transcription regulators and (3) physiological parameters associated with HFD intake for 5 days and 12 weeks. Microarray gene expression data were used for identification of enriched biological processes, identifying transcription factors regulating differentially expressed genes and determining correlation with physiological processes. Network nodes at all three layers are based on sets of genes: (1) genes involved in specific biological process, (2) genes regulated by a transcription factor and (3) genes correlating with a physiological parameters. Edges between nodes are representing the overlap between these respective sets of genes and were generated using Cytoscape version 2.8.3 and plug-in enrichment map (Merico et al. 2010; Smoot et al. 2011).

### Network visualization

The three-layered network was visualized using enrichment map. To increase interpretability of the network model, *p* value cut-off 0.05 and overlap coefficient of 0.5 were used as a cut-off prior to visualization. To visualize the overlap between physiological parameters and processes, an overlap coefficient of 0.3 was used. The enrichment *p* values were used for colour coding of the nodes in the network.

The network topology parameters for biological processes networks at 5 days and 12 weeks were calculated by plug-in advanced network analysis in Cytoscape (Supplemental Table 1).

## Electronic supplementary material

Supplementary material 1 (DOCX 15 kb)

Supplementary material 2 (DOCX 23 kb)

Supplementary material 3 (XLSX 21195 kb)

Supplementary material 4 (ZIP 6984 kb)

Supplementary material 5 (CYS 55 kb)

Supplementary material 6 (CYS 336 kb)

Supplementary material 7 (XLSX 25 kb)

Supplementary material 8 (CYS 516 kb)

Supplementary material 9 (CYS 124 kb)

Supplementary material 10 (XLSX 39 kb)

Supplementary material 11 (XLSX 41550 kb)

Supplementary material 12 (XLSX 12 kb)

Supplementary material 13 (CYS 488 kb)

Supplementary material 14 (CYS 723 kb)

## References

[CR1] Barabási A-L, Gulbahce N, Loscalzo J (2011). Network medicine: a network-based approach to human disease. Nat Rev Genet.

[CR2] Bhaskaram P (2002). Micronutrient malnutrition, infection, and immunity: an overview. Nutr Rev.

[CR3] Caesar R, Manieri M, Kelder T (2010). A combined transcriptomics and lipidomics analysis of subcutaneous, epididymal and mesenteric adipose tissue reveals marked functional differences. PLoS ONE.

[CR4] Casalena G, Daehn I, Bottinger E (2012). Transforming growth factor-β, bioenergetics, and mitochondria in renal disease. Semin Nephrol.

[CR01] Cinti S (2005). The adipose organ. Prostaglandins Leukot Essent Fatty Acids.

[CR5] Eijssen LMT, Jaillard M, Adriaens ME (2013). User-friendly solutions for microarray quality control and pre-processing on ArrayAnalysis.org. Nucleic Acids Res.

[CR6] Hill JO, Lin D, Yakubu F, Peters JC (1992). Development of dietary obesity in rats: influence of amount and composition of dietary fat. Int J Obes Relat Metab Disord.

[CR7] Hu FB (2002). Dietary pattern analysis: a new direction in nutritional epidemiology. Curr Opin Lipidol.

[CR8] Kelder T, Summer G, Caspers M (2015). White adipose tissue reference network: a knowledge resource for exploring health-relevant relations. Genes Nutr.

[CR9] Krick S, Shi S, Ju W (2008). Mpv17 l protects against mitochondrial oxidative stress and apoptosis by activation of Omi/HtrA2 protease. Proc Natl Acad Sci USA.

[CR10] Merico D, Isserlin R, Stueker O (2010). Enrichment map: a network-based method for gene-set enrichment visualization and interpretation. PLoS ONE.

[CR11] Must A (1999). The disease burden associated with overweight and obesity. JAMA.

[CR02] Owens B (2014). Cell physiology: the changing colour of fat. Nature.

[CR12] Palou A, Picó C, Bonet ML (2004). Food safety and functional foods in the European Union: obesity as a paradigmatic example for novel food development. Nutr Rev.

[CR13] Serby MD, Zhao H, Szczepankiewicz BG (2006). 2,4-diaminopyrimidine derivatives as potent growth hormone secretagogue receptor antagonists. J Med Chem.

[CR14] Smoot ME, Ono K, Ruscheinski J (2011). Cytoscape 2.8: new features for data integration and network visualization. Bioinformatics.

[CR15] Subramanian A, Tamayo P, Mootha VK (2005). Gene set enrichment analysis: a knowledge-based approach for interpreting genome-wide expression profiles. Proc Natl Acad Sci USA.

[CR16] Voigt A, Agnew K, van Schothorst EM (2013). Short-term, high fat feeding-induced changes in white adipose tissue gene expression are highly predictive for long-term changes. Mol Nutr Food Res.

[CR17] Yamada M, Miyakawa T, Duttaroy A (2001). Mice lacking the M3 muscarinic acetylcholine receptor are hypophagic and lean. Nature.

[CR18] Zimmet P, Magliano D, Matsuzawa Y (2005). The metabolic syndrome: a global public health problem and a new definition. J Atheroscler Thromb.

