# Supplementary material for: Network-based integration of molecular and physiological data elucidates regulatory mechanisms underlying adaptation to high-fat diet
Source: Genes Nutr. 2015 May 28;10(4):22. doi: 10.1007/s12263-015-0470-6 (PMC4446272; doi:10.1007/s12263-015-0470-6)
Supplement: Supplementary file 1 — Supplementary material 1 (DOCX 15 kb) [file 12263_2015_470_MOESM1_ESM.docx]

Supplemental table 1. Network topology results (without isolated nodes) for 5 days and 12 weeks. The network topology parameters for biological processes networks at 5 days and 12 weeks were calculated by plug-in advanced network analysis in Cytoscape.

|  | 5 days | 12 weeks |
| --- | --- | --- |
| Clustering coefficient | 0.375 | 0.493 |
| Connected components | 20 | 17 |
| Network centralization | 0.129 | 0.138 |
| Avg. number of neighbours | 4.349 | 4.090 |
| Number of nodes | 109 | 98 |
| Network density | 0.040 | 0.046 |
| Network heterogeneity | 0.810 | 0.724 |
