# Supplementary material for: Network-based integration of molecular and physiological data elucidates regulatory mechanisms underlying adaptation to high-fat diet
Source: Genes Nutr. 2015 May 28;10(4):22. doi: 10.1007/s12263-015-0470-6 (PMC4446272; doi:10.1007/s12263-015-0470-6)
Supplement: Supplementary file 2 — Supplementary material 2 (DOCX 23 kb) [file 12263_2015_470_MOESM2_ESM.docx]

Supplemental table 2. Overview of transcription factors found by IPA upstream analysis of DEG for five days and 12 weeks. Transcription factors with 100% overlap between their target genes were clustered. The transcription factor with highest significance was taken as cluster representative. The size of the dataset indicates the number of target genes associated with the representative transcription factor.

| Transcription factor | Clustered transcription factors | Size dataset | p-value |
| --- | --- | --- | --- |
| **5 days** |  |  |  |
| PPARGC1B | NR1H2, NR1H3, PIAS1, MYF6, NC2, RFXANK | 10 | 1.80E-15 |
| MED13 | NC2, RFXANK | 9 | 1.81E-14 |
| PPARG | NR0B2, CEBPA, PIAS1, NC2, RFXANK | 21 | 3.74E-14 |
| SREBF2 | SREBF1, MLX, PIAS1, NC2, RFXANK | 11 | 8.75E-14 |
| MLXIPL | MLX, PIAS1, MYF6, NC2, RFXANK | 8 | 1.11E-13 |
| FOXO1 | NR0B2, PIAS1, NC2, RFXANK | 15 | 6.18E-12 |
| PPARA | NR1H2, NR1H3, PIAS1, NC2, RFXANK | 18 | 7.33E-11 |
| PPARGC1A | PIAS1, MYF6, NC2, RFXANK | 10 | 8.06E-10 |
| FOXO4 | PIAS1, NC2, RFXANK | 6 | 1.86E-08 |
| EPAS1 | PIAS1, SMAD6, SMAD7, MSX2, NC2, RFXANK | 10 | 1.49E-07 |
| NRIP1 | PIAS1, MYF6, NC2, RFXANK | 6 | 2.12E-07 |
| NFYA | NC2, RFXANK, JUNB | 5 | 1.74E-06 |
| RORA | RORC, NC2, RFXANK | 8 | 3.37E-06 |
| THRB | THRA, PIAS1, NC2, RFXANK | 9 | 3.72E-06 |
| N-COR | PIAS1, NC2, RFXANK | 4 | 9.57E-06 |
| HIF1A | SMAD6, SMAD7, MSX2, NC2, RFXANK | 10 | 6.16E-05 |
| NCOA6 | USF2, SP1, NC2, RFXANK | 2 | 1.25E-04 |
| SIRT2 | NC2, RFXANK | 3 | 1.55E-04 |
| MYC | NC2, RFXANK | 14 | 2.19E-04 |
| TP53 | NC2, RFXANK, JUNB | 19 | 3.74E-04 |
| WT1 | SMAD6, SMAD7, MSX2, NC2, RFXANK | 6 | 4.51E-04 |
| ESRRA | NC2, RFXANK | 6 | 6.81E-04 |
| STAT4 | SMAD6, SMAD7, MSX2, NC2, RFXANK | 7 | 8.89E-04 |
| TAF6 | NC2, RFXANK | 2 | 1.39E-03 |
| CREB1 | KLF15, MED30, NR1H, NC2, RFXANK | 6 | 1.46E-03 |
| HTT | NC2, RFXANK | 12 | 1.58E-03 |
| RXRA | MYF6, NC2, RFXANK | 5 | 1.85E-03 |
| MTPN | SMAD6, SMAD7, MSX2, NC2, RFXANK | 4 | 2.89E-03 |
| RXRB | NC2, RFXANK | 3 | 3.63E-03 |
| NR1I2 | NC2, RFXANK | 4 | 3.86E-03 |
| NR4A1 | NR1H, NC2, RFXANK | 3 | 4.04E-03 |
| NR3C2 | NC2, RFXANK | 3 | 4.04E-03 |
| ING2 | HLTF, NC2, DLX1, FOXK2, RFXANK, BRCA2, PRDM16, TSC22D1, KDM3A | 1 | 7.18E-03 |
| FANK1 | FBXW7, MEF2, MEF2C, MEF2A, MEF2D, DACH1, MEF2BNB-MEF2B, CDKN2A, VAV1, TAF4B, SRF, HDAC6, RING1, HDAC9, BETACATENIN/TCF, TEAD4, JDP2, NC2, CYLD, MAGED1, RFXANK, ATF, AATF | 1 | 7.18E-03 |
| SLC2A4RG | NC2, RFXANK, MYBBP1A | 1 | 7.18E-03 |
| NKX2-1 | NC2, RFXANK | 3 | 7.53E-03 |
| MECOM | SMAD3, NC2, SMAD4, RFXANK | 1 | 8.08E-03 |
| FOS | NC2, RFXANK | 6 | 8.23E-03 |
| MYOD1 | NC2, RFXANK | 5 | 8.89E-03 |
| HNF4A | NC2, RFXANK | 19 | 1.23E-02 |
| NR5A2 | NC2, RFXANK | 3 | 1.68E-02 |
| OSR2 | NC2, RFXANK | 1 | 2.14E-02 |
| EGR1 | NC2, RFXANK | 4 | 2.21E-02 |
| MED1 | NC2, RFXANK | 3 | 2.26E-02 |
| SMARCB1 | NC2, RFXANK | 3 | 2.78E-02 |
| DNMT3L | NC2, RFXANK | 1 | 2.84E-02 |
| ESR1 | RFXANK | 5 | 2.97E-02 |
| PPARD | RFXANK | 3 | 3.13E-02 |
| GLI1 | RFXANK | 3 | 3.37E-02 |
| HOXA10 | NCOR1 | 3 | 4.10E-02 |
| LMO4 | LMO4 | 1 | 4.23E-02 |
| HMGA1 | HMGA1 | 2 | 4.49E-02 |
| **12 weeks** |  |  |  |
| TP53 | RFXANK | 382 | 3.38E-18 |
| HNF4A |  | 56 | 4.21E-18 |
| PPARGC1A |  | 103 | 1.77E-15 |
| PPARA |  | 31 | 1.37E-14 |
| SREBF2 |  | 57 | 1.59E-14 |
| SREBF1 | SIRT2 | 56 | 1.22E-13 |
| ESRRA |  | 182 | 7.80E-13 |
| MYC | KHDRBS1, RFXANK | 103 | 2.41E-12 |
| PPARG | RFXANK | 80 | 1.02E-11 |
| HIF1A |  | 157 | 9.33E-11 |
| HTT |  | 21 | 1.78E-10 |
| PPARGC1B |  | 12 | 4.11E-10 |
| MED30 |  | 92 | 1.74E-09 |
| NFE2L2 | MAFG, KEAP1 | 49 | 2.10E-09 |
| EPAS1 |  | 23 | 4.65E-09 |
| NRF1 |  | 106 | 1.05E-08 |
| SP1 | RFXANK | 14 | 2.46E-08 |
| MED13 |  | 43 | 4.77E-08 |
| EGR1 | RFXANK | 39 | 8.00E-07 |
| KLF2 |  | 102 | 1.08E-06 |
| FOS | WWTR1 | 76 | 5.20E-06 |
| CEBPA | RFXANK | 16 | 5.93E-06 |
| KLF15 |  | 21 | 1.10E-05 |
| NRIP1 |  | 42 | 1.32E-05 |
| PDX1 |  | 80 | 1.91E-05 |
| JUN | RFXANK | 32 | 2.30E-05 |
| NR1I2 |  | 46 | 3.11E-05 |
| SP3 | RFXANK | 11 | 4.14E-05 |
| MLXIPL | MLX | 9 | 4.73E-05 |
| TFAM |  | 49 | 5.92E-05 |
| CREB1 |  | 5 | 8.28E-05 |
| KDM3A |  | 23 | 9.05E-05 |
| ARNT |  | 23 | 1.06E-04 |
| NR1H3 |  | 39 | 1.43E-04 |
| HOXA10 |  | 12 | 2.17E-04 |
| FOXO4 |  | 80 | 3.64E-04 |
| NFKBIA | RFXANK | 45 | 3.74E-04 |
| CREB |  | 114 | 4.25E-04 |
| NR3C1 |  | 104 | 4.27E-04 |
| NFKB | RFXANK, WWTR1 | 21 | 4.82E-04 |
| MTPN | RFXANK | 49 | 5.70E-04 |
| FOXO1 |  | 43 | 5.92E-04 |
| STAT4 |  | 50 | 6.05E-04 |
| RXRA |  | 68 | 8.75E-04 |
| CEBPB | RFXANK | 24 | 1.53E-03 |
| SMAD7 | RFXANK | 41 | 1.60E-03 |
| PAX3 | RFXANK | 29 | 2.03E-03 |
| CLOCK |  | 15 | 2.23E-03 |
| NR1H2 |  | 26 | 2.58E-03 |
| ATF4 |  | 15 | 3.14E-03 |
| NR1H | LXR | 28 | 3.18E-03 |
| VHL |  | 4 | 3.23E-03 |
| DEK |  | 33 | 3.51E-03 |
| PPARD |  | 9 | 4.92E-03 |
| KAT2B | RFXANK | 7 | 5.62E-03 |
| MEF2A |  | 59 | 5.75E-03 |
| AHR | RFXANK | 12 | 5.91E-03 |
| CEBPE |  | 45 | 6.55E-03 |
| ESTROGEN |  | 25 | 6.80E-03 |
| POU2F1 |  | 30 | 7.10E-03 |
| PGR |  | 32 | 7.50E-03 |
| SPI1 |  | 77 | 8.71E-03 |
| HNF1A |  | 32 | 8.73E-03 |
| RORA |  | 13 | 1.04E-02 |
| FOSL1 |  | 16 | 1.04E-02 |
| NFYA | RFXANK | 5 | 1.04E-02 |
| SMAD5 |  | 96 | 1.06E-02 |
| CTNNB1 |  | 58 | 1.10E-02 |
| SMARCA4 |  | 15 | 1.11E-02 |
| HOXD10 |  | 4 | 1.29E-02 |
| NCOA6 |  | 7 | 1.43E-02 |
| SOX10 |  | 73 | 1.48E-02 |
| NUPR1 | RFXANK | 19 | 1.58E-02 |
| CEBPD |  | 37 | 1.58E-02 |
| EP300 | RFXANK | 7 | 1.69E-02 |
| HOXA7 |  | 4 | 1.83E-02 |
| MZF1 |  | 16 | 1.92E-02 |
| NR3C2 |  | 19 | 2.04E-02 |
| NR4A1 |  | 39 | 2.04E-02 |
| NKX2-3 |  | 7 | 2.19E-02 |
| MLL2 |  | 28 | 2.56E-02 |
| MITF |  | 8 | 2.56E-02 |
| GTF2B |  | 35 | 2.61E-02 |
| STAT6 | RFXANK | 14 | 2.65E-02 |
| USF2 |  | 20 | 2.71E-02 |
| MKL1 | MKL2 | 20 | 2.77E-02 |
| ERG |  | 24 | 2.90E-02 |
| EGR2 |  | 2 | 2.93E-02 |
| GATA |  | 1 | 3.02E-02 |
| RCOR1 |  | 21 | 3.02E-02 |
| SIRT1 |  | 28 | 3.08E-02 |
| WT1 |  | 13 | 3.10E-02 |
| ETS2 |  | 15 | 3.14E-02 |
| IFI16 |  | 7 | 3.14E-02 |
| ATF6 |  | 32 | 3.19E-02 |
| THRB |  | 8 | 3.22E-02 |
| FXR |  | 17 | 3.26E-02 |
| MED1 |  | 45 | 3.40E-02 |
| NFYB |  | 35 | 3.51E-02 |
| MYOD1 |  | 32 | 3.62E-02 |
| SMAD4 |  | 3 | 3.77E-02 |
| PDE |  | 2 | 3.95E-02 |
| HNRNPD |  | 14 | 3.95E-02 |
| CBFB |  | 25 | 4.04E-02 |
| FOXA2 |  | 10 | 4.07E-02 |
| NCOR1 |  | 10 | 4.11E-02 |
| SIN3A |  | 63 | 4.11E-02 |
| STAT3 |  | 20 | 4.24E-02 |
| JUNB |  | 20 | 4.32E-02 |
| NR1I3 |  | 35 | 4.32E-02 |
| ETS1 |  | 221 | 4.74E-02 |
