# Supplementary material for: Network-based integration of molecular and physiological data elucidates regulatory mechanisms underlying adaptation to high-fat diet
Source: Genes Nutr. 2015 May 28;10(4):22. doi: 10.1007/s12263-015-0470-6 (PMC4446272; doi:10.1007/s12263-015-0470-6)
Supplement: Supplementary file 4 — Supplementary material 4 (ZIP 6984 kb) [file 12263_2015_470_MOESM4_ESM.zip › HF LF 12 w GSEA result/BEHAVIOR.html]

Details for gene set BEHAVIOR[GSEA]

|  || Dataset | HF LF 12w\_collapsed |
| Phenotype | NoPhenotypeAvailable |
| Upregulated in class | na\_pos |
| GeneSet | BEHAVIOR |
| Enrichment Score (ES) | 0.6163911 |
| Normalized Enrichment Score (NES) | 2.3337476 |
| Nominal p-value | 0.0 |
| FDR q-value | 3.3964496E-4 |
| FWER p-Value | 0.001 |
Table: GSEA Results Summary

  

Fig 1: Enrichment plot: BEHAVIOR      
 Profile of the Running ES Score & Positions of GeneSet Members on the Rank Ordered List

  

| PROBE | GENE SYMBOL | GENE\_TITLE | RANK IN GENE LIST | RANK METRIC SCORE | RUNNING ES | CORE ENRICHMENT || 1 | CCL7 |  |  | 17 | 7.530 | 0.0628 | Yes |
| 2 | CCR3 |  |  | 46 | 6.449 | 0.1147 | Yes |
| 3 | ITGB2 |  |  | 86 | 5.478 | 0.1566 | Yes |
| 4 | CCR2 |  |  | 112 | 5.179 | 0.1980 | Yes |
| 5 | PLAUR |  |  | 117 | 5.129 | 0.2418 | Yes |
| 6 | CCL2 |  |  | 148 | 4.900 | 0.2800 | Yes |
| 7 | CCBP2 |  |  | 175 | 4.685 | 0.3169 | Yes |
| 8 | CCL11 |  |  | 222 | 4.439 | 0.3488 | Yes |
| 9 | CCL24 |  |  | 224 | 4.436 | 0.3871 | Yes |
| 10 | LEP |  |  | 227 | 4.387 | 0.4248 | Yes |
| 11 | DOCK2 |  |  | 228 | 4.377 | 0.4627 | Yes |
| 12 | FGF2 |  |  | 348 | 3.718 | 0.4781 | Yes |
| 13 | APOE |  |  | 478 | 3.193 | 0.4874 | Yes |
| 14 | ZIC1 |  |  | 483 | 3.165 | 0.5143 | Yes |
| 15 | CCR5 |  |  | 497 | 3.131 | 0.5395 | Yes |
| 16 | CCRL1 |  |  | 581 | 2.868 | 0.5526 | Yes |
| 17 | PF4 |  |  | 623 | 2.761 | 0.5707 | Yes |
| 18 | CCL17 |  |  | 696 | 2.594 | 0.5830 | Yes |
| 19 | CCL5 |  |  | 752 | 2.451 | 0.5964 | Yes |
| 20 | SPN |  |  | 761 | 2.439 | 0.6164 | Yes |
| 21 | CXCR3 |  |  | 1226 | 1.728 | 0.5655 | No |
| 22 | RALBP1 |  |  | 1286 | 1.653 | 0.5715 | No |
| 23 | CXCL9 |  |  | 1395 | 1.543 | 0.5695 | No |
| 24 | CXCL12 |  |  | 1639 | 1.275 | 0.5461 | No |
| 25 | KLK8 |  |  | 1750 | 1.156 | 0.5405 | No |
| 26 | NOVA1 |  |  | 1956 | 0.905 | 0.5192 | No |
| 27 | OPRM1 |  |  | 2261 | 0.636 | 0.4816 | No |
| 28 | CCL8 |  |  | 2273 | 0.620 | 0.4854 | No |
| 29 | PPYR1 |  |  | 2468 | 0.436 | 0.4617 | No |
| 30 | HPRT1 |  |  | 2696 | 0.248 | 0.4316 | No |
| 31 | NPY |  |  | 2955 | 0.042 | 0.3953 | No |
| 32 | MAPK1 |  |  | 2984 | 0.019 | 0.3915 | No |
| 33 | TGFB2 |  |  | 3518 | -0.356 | 0.3190 | No |
| 34 | ADCY8 |  |  | 3859 | -0.597 | 0.2759 | No |
| 35 | CDH13 |  |  | 4038 | -0.728 | 0.2570 | No |
| 36 | CXCR4 |  |  | 4118 | -0.780 | 0.2525 | No |
| 37 | CX3CL1 |  |  | 4357 | -0.956 | 0.2270 | No |
| 38 | PLAU |  |  | 5056 | -1.483 | 0.1408 | No |
| 39 | RALA |  |  | 5456 | -1.854 | 0.1002 | No |
| 40 | CXCL13 |  |  | 5611 | -2.017 | 0.0959 | No |
| 41 | CX3CR1 |  |  | 5971 | -2.485 | 0.0664 | No |
| 42 | CXCL14 |  |  | 5975 | -2.489 | 0.0876 | No |
| 43 | MAP2K1 |  |  | 6086 | -2.621 | 0.0947 | No |
| 44 | PIK3CB |  |  | 6111 | -2.656 | 0.1143 | No |
| 45 | CCL25 |  |  | 6223 | -2.843 | 0.1232 | No |
Table: GSEA details [plain text format]

  

Fig 2: BEHAVIOR: Random ES distribution      
 Gene set null distribution of ES for **BEHAVIOR**

  
