# Supplementary material for: Network-based integration of molecular and physiological data elucidates regulatory mechanisms underlying adaptation to high-fat diet
Source: Genes Nutr. 2015 May 28;10(4):22. doi: 10.1007/s12263-015-0470-6 (PMC4446272; doi:10.1007/s12263-015-0470-6)
Supplement: Supplementary file 4 — Supplementary material 4 (ZIP 6984 kb) [file 12263_2015_470_MOESM4_ESM.zip › HF LF 12 w GSEA result/CATION_HOMEOSTASIS.html]

Details for gene set CATION\_HOMEOSTASIS[GSEA]

|  || Dataset | HF LF 12w\_collapsed |
| Phenotype | NoPhenotypeAvailable |
| Upregulated in class | na\_pos |
| GeneSet | CATION\_HOMEOSTASIS |
| Enrichment Score (ES) | 0.5707806 |
| Normalized Enrichment Score (NES) | 2.080342 |
| Nominal p-value | 0.0 |
| FDR q-value | 0.0025348556 |
| FWER p-Value | 0.027 |
Table: GSEA Results Summary

  

Fig 1: Enrichment plot: CATION\_HOMEOSTASIS      
 Profile of the Running ES Score & Positions of GeneSet Members on the Rank Ordered List

  

| PROBE | GENE SYMBOL | GENE\_TITLE | RANK IN GENE LIST | RANK METRIC SCORE | RUNNING ES | CORE ENRICHMENT || 1 | CCL7 |  |  | 17 | 7.530 | 0.0892 | Yes |
| 2 | CCR3 |  |  | 46 | 6.449 | 0.1637 | Yes |
| 3 | PLCE1 |  |  | 57 | 6.082 | 0.2363 | Yes |
| 4 | CCR2 |  |  | 112 | 5.179 | 0.2917 | Yes |
| 5 | CALCA |  |  | 138 | 4.957 | 0.3484 | Yes |
| 6 | CCL2 |  |  | 148 | 4.900 | 0.4068 | Yes |
| 7 | CCL11 |  |  | 222 | 4.439 | 0.4505 | Yes |
| 8 | C3AR1 |  |  | 443 | 3.340 | 0.4599 | Yes |
| 9 | CCR5 |  |  | 497 | 3.131 | 0.4905 | Yes |
| 10 | CCL5 |  |  | 752 | 2.451 | 0.4843 | Yes |
| 11 | SRI |  |  | 832 | 2.303 | 0.5012 | Yes |
| 12 | TRPV4 |  |  | 872 | 2.233 | 0.5228 | Yes |
| 13 | AGTR1 |  |  | 1011 | 2.033 | 0.5280 | Yes |
| 14 | CXCR3 |  |  | 1226 | 1.728 | 0.5187 | Yes |
| 15 | GNA15 |  |  | 1306 | 1.640 | 0.5274 | Yes |
| 16 | GCM2 |  |  | 1389 | 1.547 | 0.5347 | Yes |
| 17 | ATP7A |  |  | 1398 | 1.539 | 0.5522 | Yes |
| 18 | BCL2 |  |  | 1486 | 1.441 | 0.5574 | Yes |
| 19 | CALR |  |  | 1514 | 1.410 | 0.5708 | Yes |
| 20 | CXCL12 |  |  | 1639 | 1.275 | 0.5687 | No |
| 21 | SLC40A1 |  |  | 1944 | 0.916 | 0.5368 | No |
| 22 | MT2A |  |  | 2389 | 0.514 | 0.4801 | No |
| 23 | MYC |  |  | 2975 | 0.023 | 0.3975 | No |
| 24 | CLN6 |  |  | 3161 | -0.103 | 0.3726 | No |
| 25 | RGN |  |  | 3193 | -0.129 | 0.3697 | No |
| 26 | ATP1A1 |  |  | 3782 | -0.539 | 0.2930 | No |
| 27 | CD52 |  |  | 3870 | -0.610 | 0.2881 | No |
| 28 | CD24 |  |  | 4094 | -0.768 | 0.2658 | No |
| 29 | CXCR4 |  |  | 4118 | -0.780 | 0.2720 | No |
| 30 | FTH1 |  |  | 4558 | -1.100 | 0.2232 | No |
| 31 | CLN5 |  |  | 4745 | -1.224 | 0.2117 | No |
| 32 | CXCL13 |  |  | 5611 | -2.017 | 0.1137 | No |
| 33 | AVPR1A |  |  | 5777 | -2.236 | 0.1175 | No |
| 34 | EDNRA |  |  | 6138 | -2.696 | 0.0993 | No |
| 35 | ATP1A2 |  |  | 6268 | -2.925 | 0.1166 | No |
Table: GSEA details [plain text format]

  

Fig 2: CATION\_HOMEOSTASIS: Random ES distribution      
 Gene set null distribution of ES for **CATION\_HOMEOSTASIS**

  
