# Supplementary material for: Network-based integration of molecular and physiological data elucidates regulatory mechanisms underlying adaptation to high-fat diet
Source: Genes Nutr. 2015 May 28;10(4):22. doi: 10.1007/s12263-015-0470-6 (PMC4446272; doi:10.1007/s12263-015-0470-6)
Supplement: Supplementary file 4 — Supplementary material 4 (ZIP 6984 kb) [file 12263_2015_470_MOESM4_ESM.zip › HF LF 12 w GSEA result/COFACTOR_METABOLIC_PROCESS.html]

Details for gene set COFACTOR\_METABOLIC\_PROCESS[GSEA]

|  || Dataset | HF LF 12w\_collapsed |
| Phenotype | NoPhenotypeAvailable |
| Upregulated in class | na\_neg |
| GeneSet | COFACTOR\_METABOLIC\_PROCESS |
| Enrichment Score (ES) | -0.68038696 |
| Normalized Enrichment Score (NES) | -1.9302855 |
| Nominal p-value | 0.0 |
| FDR q-value | 0.0035060486 |
| FWER p-Value | 0.083 |
Table: GSEA Results Summary

  

Fig 1: Enrichment plot: COFACTOR\_METABOLIC\_PROCESS      
 Profile of the Running ES Score & Positions of GeneSet Members on the Rank Ordered List

  

| PROBE | GENE SYMBOL | GENE\_TITLE | RANK IN GENE LIST | RANK METRIC SCORE | RUNNING ES | CORE ENRICHMENT || 1 | GPX1 |  |  | 317 | 3.848 | 0.0244 | No |
| 2 | BLVRA |  |  | 2184 | 0.696 | -0.2268 | No |
| 3 | NFS1 |  |  | 2605 | 0.314 | -0.2806 | No |
| 4 | MTHFD2 |  |  | 2836 | 0.129 | -0.3107 | No |
| 5 | ALAS2 |  |  | 4053 | -0.741 | -0.4693 | No |
| 6 | CPOX |  |  | 5024 | -1.458 | -0.5802 | No |
| 7 | PPOX |  |  | 5159 | -1.558 | -0.5711 | No |
| 8 | COX10 |  |  | 5500 | -1.902 | -0.5850 | No |
| 9 | MOCS2 |  |  | 5687 | -2.122 | -0.5731 | No |
| 10 | MLYCD |  |  | 6447 | -3.298 | -0.6211 | Yes |
| 11 | CTNS |  |  | 6698 | -4.004 | -0.5844 | Yes |
| 12 | COX15 |  |  | 6801 | -4.443 | -0.5189 | Yes |
| 13 | SDHD |  |  | 6818 | -4.523 | -0.4398 | Yes |
| 14 | MOCOS |  |  | 6859 | -4.761 | -0.3598 | Yes |
| 15 | GCLM |  |  | 6894 | -5.004 | -0.2746 | Yes |
| 16 | COQ7 |  |  | 6896 | -5.009 | -0.1846 | Yes |
| 17 | GCLC |  |  | 6977 | -5.860 | -0.0905 | Yes |
| 18 | ACO2 |  |  | 6981 | -5.918 | 0.0155 | Yes |
Table: GSEA details [plain text format]

  

Fig 2: COFACTOR\_METABOLIC\_PROCESS: Random ES distribution      
 Gene set null distribution of ES for **COFACTOR\_METABOLIC\_PROCESS**

  
