# Supplementary material for: Network-based integration of molecular and physiological data elucidates regulatory mechanisms underlying adaptation to high-fat diet
Source: Genes Nutr. 2015 May 28;10(4):22. doi: 10.1007/s12263-015-0470-6 (PMC4446272; doi:10.1007/s12263-015-0470-6)
Supplement: Supplementary file 4 — Supplementary material 4 (ZIP 6984 kb) [file 12263_2015_470_MOESM4_ESM.zip › HF LF 12 w GSEA result/DEFENSE_RESPONSE.html]

Details for gene set DEFENSE\_RESPONSE[GSEA]

|  || Dataset | HF LF 12w\_collapsed |
| Phenotype | NoPhenotypeAvailable |
| Upregulated in class | na\_pos |
| GeneSet | DEFENSE\_RESPONSE |
| Enrichment Score (ES) | 0.50705665 |
| Normalized Enrichment Score (NES) | 2.135986 |
| Nominal p-value | 0.0 |
| FDR q-value | 0.0012602949 |
| FWER p-Value | 0.012 |
Table: GSEA Results Summary

  

Fig 1: Enrichment plot: DEFENSE\_RESPONSE      
 Profile of the Running ES Score & Positions of GeneSet Members on the Rank Ordered List

  

| PROBE | GENE SYMBOL | GENE\_TITLE | RANK IN GENE LIST | RANK METRIC SCORE | RUNNING ES | CORE ENRICHMENT || 1 | LTB4R |  |  | 14 | 7.889 | 0.0455 | Yes |
| 2 | TYROBP |  |  | 38 | 6.794 | 0.0832 | Yes |
| 3 | CD83 |  |  | 40 | 6.736 | 0.1237 | Yes |
| 4 | CCR3 |  |  | 46 | 6.449 | 0.1618 | Yes |
| 5 | C2 |  |  | 103 | 5.276 | 0.1856 | Yes |
| 6 | CCR2 |  |  | 112 | 5.179 | 0.2157 | Yes |
| 7 | ALOX5AP |  |  | 139 | 4.956 | 0.2419 | Yes |
| 8 | MEFV |  |  | 143 | 4.917 | 0.2711 | Yes |
| 9 | LBP |  |  | 176 | 4.677 | 0.2947 | Yes |
| 10 | CCL11 |  |  | 222 | 4.439 | 0.3150 | Yes |
| 11 | CCL24 |  |  | 224 | 4.436 | 0.3416 | Yes |
| 12 | FOS |  |  | 265 | 4.135 | 0.3608 | Yes |
| 13 | MST1R |  |  | 419 | 3.441 | 0.3598 | Yes |
| 14 | TLR6 |  |  | 430 | 3.410 | 0.3789 | Yes |
| 15 | C3AR1 |  |  | 443 | 3.340 | 0.3973 | Yes |
| 16 | STAB1 |  |  | 459 | 3.268 | 0.4149 | Yes |
| 17 | CCL4 |  |  | 486 | 3.157 | 0.4302 | Yes |
| 18 | CCR5 |  |  | 497 | 3.131 | 0.4476 | Yes |
| 19 | WAS |  |  | 507 | 3.096 | 0.4650 | Yes |
| 20 | NFATC4 |  |  | 621 | 2.761 | 0.4655 | Yes |
| 21 | AOC3 |  |  | 641 | 2.718 | 0.4792 | Yes |
| 22 | CCL5 |  |  | 752 | 2.451 | 0.4783 | Yes |
| 23 | SPN |  |  | 761 | 2.439 | 0.4919 | Yes |
| 24 | CD81 |  |  | 830 | 2.304 | 0.4961 | Yes |
| 25 | KCNN4 |  |  | 869 | 2.235 | 0.5041 | Yes |
| 26 | TCIRG1 |  |  | 939 | 2.121 | 0.5071 | Yes |
| 27 | TNFRSF1A |  |  | 1146 | 1.835 | 0.4888 | No |
| 28 | AIF1 |  |  | 1336 | 1.603 | 0.4715 | No |
| 29 | S100A8 |  |  | 1340 | 1.600 | 0.4807 | No |
| 30 | CXCL9 |  |  | 1395 | 1.543 | 0.4823 | No |
| 31 | COLEC12 |  |  | 1451 | 1.484 | 0.4834 | No |
| 32 | ITGB1 |  |  | 1459 | 1.476 | 0.4913 | No |
| 33 | BCL2 |  |  | 1486 | 1.441 | 0.4963 | No |
| 34 | HDAC4 |  |  | 1596 | 1.330 | 0.4888 | No |
| 35 | PARP4 |  |  | 1628 | 1.290 | 0.4921 | No |
| 36 | ZNF148 |  |  | 1764 | 1.130 | 0.4797 | No |
| 37 | ABCF1 |  |  | 1787 | 1.105 | 0.4832 | No |
| 38 | RNASE6 |  |  | 1933 | 0.926 | 0.4682 | No |
| 39 | PTPRCAP |  |  | 1967 | 0.893 | 0.4688 | No |
| 40 | TGFB1 |  |  | 2164 | 0.718 | 0.4452 | No |
| 41 | RAC1 |  |  | 2194 | 0.686 | 0.4452 | No |
| 42 | LYST |  |  | 2268 | 0.629 | 0.4386 | No |
| 43 | LY96 |  |  | 2742 | 0.214 | 0.3725 | No |
| 44 | INHBA |  |  | 2769 | 0.194 | 0.3700 | No |
| 45 | AHSG |  |  | 2834 | 0.131 | 0.3617 | No |
| 46 | PLA2G2D |  |  | 2954 | 0.044 | 0.3450 | No |
| 47 | NFATC3 |  |  | 3127 | -0.085 | 0.3210 | No |
| 48 | NFRKB |  |  | 3325 | -0.216 | 0.2942 | No |
| 49 | BECN1 |  |  | 3334 | -0.224 | 0.2944 | No |
| 50 | VEZF1 |  |  | 3696 | -0.482 | 0.2459 | No |
| 51 | CLEC1A |  |  | 3708 | -0.489 | 0.2473 | No |
| 52 | F11R |  |  | 4020 | -0.711 | 0.2073 | No |
| 53 | CXCR4 |  |  | 4118 | -0.780 | 0.1981 | No |
| 54 | MX2 |  |  | 4152 | -0.800 | 0.1983 | No |
| 55 | CD5L |  |  | 4293 | -0.908 | 0.1838 | No |
| 56 | WFDC12 |  |  | 4317 | -0.923 | 0.1861 | No |
| 57 | FAIM3 |  |  | 4323 | -0.929 | 0.1910 | No |
| 58 | CX3CL1 |  |  | 4357 | -0.956 | 0.1920 | No |
| 59 | LGALS3BP |  |  | 4377 | -0.971 | 0.1952 | No |
| 60 | CEBPG |  |  | 4668 | -1.174 | 0.1609 | No |
| 61 | TIAL1 |  |  | 4779 | -1.255 | 0.1528 | No |
| 62 | NFX1 |  |  | 5027 | -1.459 | 0.1264 | No |
| 63 | ELF3 |  |  | 5164 | -1.567 | 0.1165 | No |
| 64 | AOX1 |  |  | 5181 | -1.579 | 0.1237 | No |
| 65 | IL28RA |  |  | 5450 | -1.848 | 0.0967 | No |
| 66 | BCL10 |  |  | 5604 | -2.007 | 0.0870 | No |
| 67 | CAMLG |  |  | 5685 | -2.119 | 0.0883 | No |
| 68 | CX3CR1 |  |  | 5971 | -2.485 | 0.0627 | No |
| 69 | ALOX15 |  |  | 6081 | -2.614 | 0.0629 | No |
| 70 | RSAD2 |  |  | 6354 | -3.088 | 0.0428 | No |
| 71 | ORM1 |  |  | 6398 | -3.186 | 0.0559 | No |
| 72 | ORM2 |  |  | 6469 | -3.363 | 0.0662 | No |
| 73 | CDO1 |  |  | 6608 | -3.703 | 0.0688 | No |
Table: GSEA details [plain text format]

  

Fig 2: DEFENSE\_RESPONSE: Random ES distribution      
 Gene set null distribution of ES for **DEFENSE\_RESPONSE**

  
