# Supplementary material for: Network-based integration of molecular and physiological data elucidates regulatory mechanisms underlying adaptation to high-fat diet
Source: Genes Nutr. 2015 May 28;10(4):22. doi: 10.1007/s12263-015-0470-6 (PMC4446272; doi:10.1007/s12263-015-0470-6)
Supplement: Supplementary file 4 — Supplementary material 4 (ZIP 6984 kb) [file 12263_2015_470_MOESM4_ESM.zip › HF LF 12 w GSEA result/ENVELOPE.html]

Details for gene set ENVELOPE[GSEA]

|  || Dataset | HF LF 12w\_collapsed |
| Phenotype | NoPhenotypeAvailable |
| Upregulated in class | na\_neg |
| GeneSet | ENVELOPE |
| Enrichment Score (ES) | -0.56581146 |
| Normalized Enrichment Score (NES) | -2.15666 |
| Nominal p-value | 0.0 |
| FDR q-value | 1.695915E-4 |
| FWER p-Value | 0.003 |
Table: GSEA Results Summary

  

Fig 1: Enrichment plot: ENVELOPE      
 Profile of the Running ES Score & Positions of GeneSet Members on the Rank Ordered List

  

| PROBE | GENE SYMBOL | GENE\_TITLE | RANK IN GENE LIST | RANK METRIC SCORE | RUNNING ES | CORE ENRICHMENT || 1 | MCL1 |  |  | 214 | 4.478 | -0.0079 | No |
| 2 | RAB11FIP5 |  |  | 339 | 3.759 | -0.0065 | No |
| 3 | SCRN1 |  |  | 487 | 3.157 | -0.0115 | No |
| 4 | NUP98 |  |  | 563 | 2.931 | -0.0074 | No |
| 5 | LBR |  |  | 697 | 2.591 | -0.0133 | No |
| 6 | GATM |  |  | 808 | 2.348 | -0.0171 | No |
| 7 | COX6B2 |  |  | 1180 | 1.785 | -0.0609 | No |
| 8 | LMNB1 |  |  | 1185 | 1.777 | -0.0525 | No |
| 9 | BCL2 |  |  | 1486 | 1.441 | -0.0880 | No |
| 10 | DDX19B |  |  | 1505 | 1.420 | -0.0833 | No |
| 11 | XPO1 |  |  | 1510 | 1.413 | -0.0768 | No |
| 12 | EPC1 |  |  | 1997 | 0.859 | -0.1417 | No |
| 13 | NUP50 |  |  | 2125 | 0.748 | -0.1560 | No |
| 14 | NRM |  |  | 2283 | 0.610 | -0.1753 | No |
| 15 | PSEN1 |  |  | 2304 | 0.587 | -0.1752 | No |
| 16 | RANGAP1 |  |  | 2427 | 0.478 | -0.1902 | No |
| 17 | NUP160 |  |  | 2544 | 0.368 | -0.2048 | No |
| 18 | NUP153 |  |  | 2926 | 0.064 | -0.2588 | No |
| 19 | AAAS |  |  | 3317 | -0.209 | -0.3133 | No |
| 20 | OXA1L |  |  | 3411 | -0.282 | -0.3252 | No |
| 21 | UCP3 |  |  | 3585 | -0.399 | -0.3478 | No |
| 22 | CASP7 |  |  | 3823 | -0.573 | -0.3787 | No |
| 23 | MPV17 |  |  | 3962 | -0.683 | -0.3949 | No |
| 24 | NUTF2 |  |  | 4039 | -0.729 | -0.4020 | No |
| 25 | ALAS2 |  |  | 4053 | -0.741 | -0.4001 | No |
| 26 | NUP133 |  |  | 4218 | -0.852 | -0.4192 | No |
| 27 | IPO7 |  |  | 4235 | -0.864 | -0.4171 | No |
| 28 | NPC1 |  |  | 4314 | -0.922 | -0.4236 | No |
| 29 | ABCB6 |  |  | 4326 | -0.930 | -0.4204 | No |
| 30 | RANBP2 |  |  | 4589 | -1.133 | -0.4521 | No |
| 31 | MFN2 |  |  | 4803 | -1.272 | -0.4760 | No |
| 32 | MATR3 |  |  | 4888 | -1.346 | -0.4811 | No |
| 33 | PPOX |  |  | 5159 | -1.558 | -0.5117 | No |
| 34 | RTN4 |  |  | 5364 | -1.744 | -0.5320 | No |
| 35 | ABCF2 |  |  | 5491 | -1.892 | -0.5404 | No |
| 36 | KPNA3 |  |  | 5560 | -1.959 | -0.5402 | No |
| 37 | ABCB7 |  |  | 5622 | -2.034 | -0.5386 | No |
| 38 | TIMM17A |  |  | 5814 | -2.288 | -0.5542 | Yes |
| 39 | PHB |  |  | 5891 | -2.384 | -0.5530 | Yes |
| 40 | KPNB1 |  |  | 5916 | -2.423 | -0.5442 | Yes |
| 41 | NDUFA2 |  |  | 5941 | -2.459 | -0.5352 | Yes |
| 42 | KPNA1 |  |  | 5969 | -2.485 | -0.5265 | Yes |
| 43 | ATP5E |  |  | 6002 | -2.522 | -0.5183 | Yes |
| 44 | POLA1 |  |  | 6023 | -2.537 | -0.5083 | Yes |
| 45 | PARP1 |  |  | 6092 | -2.629 | -0.5047 | Yes |
| 46 | MAOB |  |  | 6130 | -2.689 | -0.4964 | Yes |
| 47 | NUP54 |  |  | 6235 | -2.876 | -0.4967 | Yes |
| 48 | RHOT2 |  |  | 6381 | -3.140 | -0.5015 | Yes |
| 49 | TIMM50 |  |  | 6437 | -3.271 | -0.4928 | Yes |
| 50 | MRPL32 |  |  | 6491 | -3.417 | -0.4831 | Yes |
| 51 | MTX2 |  |  | 6514 | -3.473 | -0.4686 | Yes |
| 52 | NDUFS2 |  |  | 6521 | -3.486 | -0.4519 | Yes |
| 53 | HTATIP2 |  |  | 6534 | -3.515 | -0.4358 | Yes |
| 54 | TIMM9 |  |  | 6556 | -3.553 | -0.4208 | Yes |
| 55 | SURF1 |  |  | 6573 | -3.596 | -0.4049 | Yes |
| 56 | SLC25A11 |  |  | 6582 | -3.619 | -0.3878 | Yes |
| 57 | UQCRC1 |  |  | 6607 | -3.702 | -0.3725 | Yes |
| 58 | TIMM8B |  |  | 6651 | -3.835 | -0.3592 | Yes |
| 59 | MRPL19 |  |  | 6652 | -3.837 | -0.3398 | Yes |
| 60 | RAE1 |  |  | 6654 | -3.844 | -0.3206 | Yes |
| 61 | CYCS |  |  | 6766 | -4.280 | -0.3147 | Yes |
| 62 | NDUFA9 |  |  | 6784 | -4.356 | -0.2951 | Yes |
| 63 | ACN9 |  |  | 6793 | -4.400 | -0.2740 | Yes |
| 64 | COX15 |  |  | 6801 | -4.443 | -0.2526 | Yes |
| 65 | PMPCA |  |  | 6817 | -4.514 | -0.2319 | Yes |
| 66 | SDHD |  |  | 6818 | -4.523 | -0.2090 | Yes |
| 67 | OPA1 |  |  | 6841 | -4.614 | -0.1889 | Yes |
| 68 | PTGER3 |  |  | 6866 | -4.787 | -0.1681 | Yes |
| 69 | ATP5B |  |  | 6904 | -5.072 | -0.1477 | Yes |
| 70 | NDUFS4 |  |  | 6939 | -5.391 | -0.1253 | Yes |
| 71 | NDUFA1 |  |  | 6941 | -5.397 | -0.0982 | Yes |
| 72 | NDUFAB1 |  |  | 6957 | -5.581 | -0.0721 | Yes |
| 73 | TIMM10 |  |  | 6965 | -5.684 | -0.0444 | Yes |
| 74 | NDUFA6 |  |  | 6987 | -6.084 | -0.0166 | Yes |
| 75 | NDUFS1 |  |  | 6998 | -6.186 | 0.0133 | Yes |
Table: GSEA details [plain text format]

  

Fig 2: ENVELOPE: Random ES distribution      
 Gene set null distribution of ES for **ENVELOPE**

  
