# Supplementary material for: Network-based integration of molecular and physiological data elucidates regulatory mechanisms underlying adaptation to high-fat diet
Source: Genes Nutr. 2015 May 28;10(4):22. doi: 10.1007/s12263-015-0470-6 (PMC4446272; doi:10.1007/s12263-015-0470-6)
Supplement: Supplementary file 4 — Supplementary material 4 (ZIP 6984 kb) [file 12263_2015_470_MOESM4_ESM.zip › HF LF 12 w GSEA result/EXTRACELLULAR_MATRIX.html]

Details for gene set EXTRACELLULAR\_MATRIX[GSEA]

|  || Dataset | HF LF 12w\_collapsed |
| Phenotype | NoPhenotypeAvailable |
| Upregulated in class | na\_pos |
| GeneSet | EXTRACELLULAR\_MATRIX |
| Enrichment Score (ES) | 0.59278524 |
| Normalized Enrichment Score (NES) | 2.16837 |
| Nominal p-value | 0.0 |
| FDR q-value | 8.7988464E-4 |
| FWER p-Value | 0.006 |
Table: GSEA Results Summary

  

Fig 1: Enrichment plot: EXTRACELLULAR\_MATRIX      
 Profile of the Running ES Score & Positions of GeneSet Members on the Rank Ordered List

  

| PROBE | GENE SYMBOL | GENE\_TITLE | RANK IN GENE LIST | RANK METRIC SCORE | RUNNING ES | CORE ENRICHMENT || 1 | DST |  |  | 26 | 7.072 | 0.0834 | Yes |
| 2 | CD248 |  |  | 59 | 6.073 | 0.1537 | Yes |
| 3 | EFEMP2 |  |  | 90 | 5.433 | 0.2163 | Yes |
| 4 | FBLN2 |  |  | 162 | 4.842 | 0.2659 | Yes |
| 5 | ECM1 |  |  | 173 | 4.694 | 0.3223 | Yes |
| 6 | FBN1 |  |  | 210 | 4.504 | 0.3726 | Yes |
| 7 | COL1A2 |  |  | 243 | 4.294 | 0.4210 | Yes |
| 8 | COL6A3 |  |  | 308 | 3.880 | 0.4597 | Yes |
| 9 | SGCD |  |  | 332 | 3.795 | 0.5032 | Yes |
| 10 | COL5A1 |  |  | 473 | 3.215 | 0.5229 | Yes |
| 11 | LTBP4 |  |  | 610 | 2.794 | 0.5381 | Yes |
| 12 | LAMB2 |  |  | 679 | 2.625 | 0.5608 | Yes |
| 13 | COL8A1 |  |  | 753 | 2.451 | 0.5806 | Yes |
| 14 | FMOD |  |  | 967 | 2.091 | 0.5762 | Yes |
| 15 | CTGF |  |  | 1033 | 1.994 | 0.5915 | Yes |
| 16 | COL3A1 |  |  | 1222 | 1.732 | 0.5862 | Yes |
| 17 | LUM |  |  | 1357 | 1.582 | 0.5867 | Yes |
| 18 | COL15A1 |  |  | 1506 | 1.416 | 0.5832 | Yes |
| 19 | COMP |  |  | 1589 | 1.335 | 0.5880 | Yes |
| 20 | LAMA4 |  |  | 1664 | 1.240 | 0.5928 | Yes |
| 21 | SNTB1 |  |  | 1934 | 0.925 | 0.5661 | No |
| 22 | FBLN1 |  |  | 1980 | 0.877 | 0.5705 | No |
| 23 | TGFB1 |  |  | 2164 | 0.718 | 0.5534 | No |
| 24 | SNTB2 |  |  | 2357 | 0.546 | 0.5329 | No |
| 25 | COL4A2 |  |  | 3059 | -0.039 | 0.4341 | No |
| 26 | COL5A3 |  |  | 3685 | -0.475 | 0.3514 | No |
| 27 | DMD |  |  | 3717 | -0.496 | 0.3531 | No |
| 28 | CHAD |  |  | 4085 | -0.762 | 0.3105 | No |
| 29 | MAGEE1 |  |  | 4270 | -0.891 | 0.2954 | No |
| 30 | COL4A3 |  |  | 4340 | -0.943 | 0.2972 | No |
| 31 | MGP |  |  | 4369 | -0.965 | 0.3051 | No |
| 32 | NID2 |  |  | 5234 | -1.622 | 0.2027 | No |
| 33 | ADAMTS9 |  |  | 5598 | -1.998 | 0.1759 | No |
| 34 | TINAG |  |  | 6242 | -2.885 | 0.1203 | No |
Table: GSEA details [plain text format]

  

Fig 2: EXTRACELLULAR\_MATRIX: Random ES distribution      
 Gene set null distribution of ES for **EXTRACELLULAR\_MATRIX**

  
