# Supplementary material for: Network-based integration of molecular and physiological data elucidates regulatory mechanisms underlying adaptation to high-fat diet
Source: Genes Nutr. 2015 May 28;10(4):22. doi: 10.1007/s12263-015-0470-6 (PMC4446272; doi:10.1007/s12263-015-0470-6)
Supplement: Supplementary file 4 — Supplementary material 4 (ZIP 6984 kb) [file 12263_2015_470_MOESM4_ESM.zip › HF LF 12 w GSEA result/EXTRACELLULAR_REGION.html]

Details for gene set EXTRACELLULAR\_REGION[GSEA]

|  || Dataset | HF LF 12w\_collapsed |
| Phenotype | NoPhenotypeAvailable |
| Upregulated in class | na\_pos |
| GeneSet | EXTRACELLULAR\_REGION |
| Enrichment Score (ES) | 0.5461409 |
| Normalized Enrichment Score (NES) | 2.5736094 |
| Nominal p-value | 0.0 |
| FDR q-value | 0.0 |
| FWER p-Value | 0.0 |
Table: GSEA Results Summary

  

Fig 1: Enrichment plot: EXTRACELLULAR\_REGION      
 Profile of the Running ES Score & Positions of GeneSet Members on the Rank Ordered List

  

| PROBE | GENE SYMBOL | GENE\_TITLE | RANK IN GENE LIST | RANK METRIC SCORE | RUNNING ES | CORE ENRICHMENT || 1 | SFRP4 |  |  | 5 | 8.683 | 0.0262 | Yes |
| 2 | EMILIN2 |  |  | 10 | 8.199 | 0.0511 | Yes |
| 3 | CCL7 |  |  | 17 | 7.530 | 0.0736 | Yes |
| 4 | DST |  |  | 26 | 7.072 | 0.0944 | Yes |
| 5 | LOXL2 |  |  | 53 | 6.171 | 0.1098 | Yes |
| 6 | CD248 |  |  | 59 | 6.073 | 0.1279 | Yes |
| 7 | SLIT3 |  |  | 60 | 6.052 | 0.1466 | Yes |
| 8 | TNFAIP2 |  |  | 66 | 5.939 | 0.1644 | Yes |
| 9 | EFEMP2 |  |  | 90 | 5.433 | 0.1779 | Yes |
| 10 | C2 |  |  | 103 | 5.276 | 0.1925 | Yes |
| 11 | EDN1 |  |  | 136 | 4.966 | 0.2033 | Yes |
| 12 | CALCA |  |  | 138 | 4.957 | 0.2186 | Yes |
| 13 | PTHLH |  |  | 145 | 4.909 | 0.2329 | Yes |
| 14 | CCL2 |  |  | 148 | 4.900 | 0.2478 | Yes |
| 15 | FBLN2 |  |  | 162 | 4.842 | 0.2610 | Yes |
| 16 | ECM1 |  |  | 173 | 4.694 | 0.2741 | Yes |
| 17 | LBP |  |  | 176 | 4.677 | 0.2883 | Yes |
| 18 | TGFBI |  |  | 181 | 4.636 | 0.3021 | Yes |
| 19 | CPB2 |  |  | 197 | 4.579 | 0.3142 | Yes |
| 20 | F13A1 |  |  | 201 | 4.543 | 0.3278 | Yes |
| 21 | FBN1 |  |  | 210 | 4.504 | 0.3407 | Yes |
| 22 | LOXL1 |  |  | 211 | 4.489 | 0.3546 | Yes |
| 23 | LEP |  |  | 227 | 4.387 | 0.3660 | Yes |
| 24 | COL1A2 |  |  | 243 | 4.294 | 0.3772 | Yes |
| 25 | C1QB |  |  | 264 | 4.144 | 0.3872 | Yes |
| 26 | PVR |  |  | 284 | 4.051 | 0.3970 | Yes |
| 27 | ADM |  |  | 301 | 3.945 | 0.4069 | Yes |
| 28 | COL6A3 |  |  | 308 | 3.880 | 0.4181 | Yes |
| 29 | GPC1 |  |  | 322 | 3.844 | 0.4282 | Yes |
| 30 | SGCD |  |  | 332 | 3.795 | 0.4386 | Yes |
| 31 | TNFRSF11B |  |  | 341 | 3.753 | 0.4491 | Yes |
| 32 | FGF2 |  |  | 348 | 3.718 | 0.4598 | Yes |
| 33 | C1QA |  |  | 376 | 3.596 | 0.4671 | Yes |
| 34 | LGALS7 |  |  | 448 | 3.322 | 0.4672 | Yes |
| 35 | COL5A1 |  |  | 473 | 3.215 | 0.4737 | Yes |
| 36 | APOE |  |  | 478 | 3.193 | 0.4830 | Yes |
| 37 | CCL4 |  |  | 486 | 3.157 | 0.4918 | Yes |
| 38 | PCSK5 |  |  | 489 | 3.153 | 0.5013 | Yes |
| 39 | SERPINF1 |  |  | 516 | 3.066 | 0.5070 | Yes |
| 40 | PCSK2 |  |  | 588 | 2.849 | 0.5057 | Yes |
| 41 | LTBP4 |  |  | 610 | 2.794 | 0.5113 | Yes |
| 42 | EBI3 |  |  | 654 | 2.682 | 0.5134 | Yes |
| 43 | LAMB2 |  |  | 679 | 2.625 | 0.5181 | Yes |
| 44 | IL27 |  |  | 684 | 2.615 | 0.5257 | Yes |
| 45 | IFI30 |  |  | 703 | 2.577 | 0.5311 | Yes |
| 46 | PCSK1N |  |  | 724 | 2.521 | 0.5360 | Yes |
| 47 | COL8A1 |  |  | 753 | 2.451 | 0.5396 | Yes |
| 48 | SPN |  |  | 761 | 2.439 | 0.5461 | Yes |
| 49 | GPX3 |  |  | 930 | 2.136 | 0.5286 | No |
| 50 | FMOD |  |  | 967 | 2.091 | 0.5299 | No |
| 51 | CTRL |  |  | 991 | 2.050 | 0.5329 | No |
| 52 | CTGF |  |  | 1033 | 1.994 | 0.5332 | No |
| 53 | HBEGF |  |  | 1139 | 1.848 | 0.5238 | No |
| 54 | PSAP |  |  | 1179 | 1.785 | 0.5238 | No |
| 55 | COL3A1 |  |  | 1222 | 1.732 | 0.5231 | No |
| 56 | NOG |  |  | 1310 | 1.638 | 0.5156 | No |
| 57 | VWF |  |  | 1356 | 1.583 | 0.5141 | No |
| 58 | LUM |  |  | 1357 | 1.582 | 0.5190 | No |
| 59 | CXCL9 |  |  | 1395 | 1.543 | 0.5184 | No |
| 60 | COL15A1 |  |  | 1506 | 1.416 | 0.5070 | No |
| 61 | MMP2 |  |  | 1524 | 1.406 | 0.5089 | No |
| 62 | COMP |  |  | 1589 | 1.335 | 0.5038 | No |
| 63 | ANGPTL4 |  |  | 1633 | 1.284 | 0.5016 | No |
| 64 | LAMA4 |  |  | 1664 | 1.240 | 0.5012 | No |
| 65 | KLK8 |  |  | 1750 | 1.156 | 0.4925 | No |
| 66 | RAB35 |  |  | 1759 | 1.139 | 0.4949 | No |
| 67 | DKKL1 |  |  | 1793 | 1.100 | 0.4936 | No |
| 68 | RNASE6 |  |  | 1933 | 0.926 | 0.4764 | No |
| 69 | SNTB1 |  |  | 1934 | 0.925 | 0.4793 | No |
| 70 | FBLN1 |  |  | 1980 | 0.877 | 0.4755 | No |
| 71 | ANGPT2 |  |  | 1999 | 0.859 | 0.4756 | No |
| 72 | IL16 |  |  | 2050 | 0.812 | 0.4709 | No |
| 73 | TGFB1 |  |  | 2164 | 0.718 | 0.4569 | No |
| 74 | NUCB2 |  |  | 2260 | 0.637 | 0.4452 | No |
| 75 | SNTB2 |  |  | 2357 | 0.546 | 0.4331 | No |
| 76 | FLT1 |  |  | 2449 | 0.451 | 0.4214 | No |
| 77 | INHA |  |  | 2493 | 0.417 | 0.4165 | No |
| 78 | PRG2 |  |  | 2635 | 0.288 | 0.3971 | No |
| 79 | CFH |  |  | 2652 | 0.275 | 0.3956 | No |
| 80 | FGG |  |  | 2668 | 0.261 | 0.3943 | No |
| 81 | APOA1 |  |  | 2721 | 0.230 | 0.3875 | No |
| 82 | COPA |  |  | 2729 | 0.224 | 0.3872 | No |
| 83 | HYAL1 |  |  | 2737 | 0.220 | 0.3868 | No |
| 84 | INHBA |  |  | 2769 | 0.194 | 0.3830 | No |
| 85 | KLK10 |  |  | 2846 | 0.126 | 0.3724 | No |
| 86 | FXYD6 |  |  | 2902 | 0.078 | 0.3648 | No |
| 87 | PLA2G2D |  |  | 2954 | 0.044 | 0.3576 | No |
| 88 | NPY |  |  | 2955 | 0.042 | 0.3577 | No |
| 89 | FGL2 |  |  | 2956 | 0.041 | 0.3578 | No |
| 90 | COL4A2 |  |  | 3059 | -0.039 | 0.3433 | No |
| 91 | RNH1 |  |  | 3125 | -0.084 | 0.3342 | No |
| 92 | FGB |  |  | 3145 | -0.096 | 0.3317 | No |
| 93 | FGF10 |  |  | 3147 | -0.097 | 0.3319 | No |
| 94 | MMP9 |  |  | 3178 | -0.120 | 0.3279 | No |
| 95 | RTN3 |  |  | 3227 | -0.152 | 0.3215 | No |
| 96 | IL15 |  |  | 3302 | -0.200 | 0.3115 | No |
| 97 | ERBB2 |  |  | 3335 | -0.226 | 0.3076 | No |
| 98 | TGFB2 |  |  | 3518 | -0.356 | 0.2825 | No |
| 99 | CLEC11A |  |  | 3542 | -0.369 | 0.2803 | No |
| 100 | TFRC |  |  | 3560 | -0.382 | 0.2790 | No |
| 101 | NUCB1 |  |  | 3639 | -0.443 | 0.2692 | No |
| 102 | MYOC |  |  | 3658 | -0.457 | 0.2680 | No |
| 103 | COL5A3 |  |  | 3685 | -0.475 | 0.2657 | No |
| 104 | DMD |  |  | 3717 | -0.496 | 0.2628 | No |
| 105 | CABP4 |  |  | 3728 | -0.502 | 0.2629 | No |
| 106 | LIPE |  |  | 3816 | -0.564 | 0.2522 | No |
| 107 | RNPEP |  |  | 3863 | -0.601 | 0.2474 | No |
| 108 | CDH13 |  |  | 4038 | -0.728 | 0.2246 | No |
| 109 | CHAD |  |  | 4085 | -0.762 | 0.2203 | No |
| 110 | LPL |  |  | 4122 | -0.783 | 0.2176 | No |
| 111 | IK |  |  | 4228 | -0.857 | 0.2051 | No |
| 112 | MAGEE1 |  |  | 4270 | -0.891 | 0.2020 | No |
| 113 | CD5L |  |  | 4293 | -0.908 | 0.2017 | No |
| 114 | WFDC12 |  |  | 4317 | -0.923 | 0.2012 | No |
| 115 | COL4A3 |  |  | 4340 | -0.943 | 0.2010 | No |
| 116 | CX3CL1 |  |  | 4357 | -0.956 | 0.2016 | No |
| 117 | MGP |  |  | 4369 | -0.965 | 0.2030 | No |
| 118 | LGALS3BP |  |  | 4377 | -0.971 | 0.2050 | No |
| 119 | CRISP1 |  |  | 4427 | -1.008 | 0.2011 | No |
| 120 | SECTM1 |  |  | 4442 | -1.017 | 0.2022 | No |
| 121 | PNLIPRP1 |  |  | 4463 | -1.029 | 0.2026 | No |
| 122 | VTN |  |  | 4496 | -1.059 | 0.2012 | No |
| 123 | IGFBP1 |  |  | 4662 | -1.170 | 0.1811 | No |
| 124 | IFNAR2 |  |  | 4675 | -1.183 | 0.1831 | No |
| 125 | ANGPTL3 |  |  | 4934 | -1.372 | 0.1502 | No |
| 126 | IGFALS |  |  | 5017 | -1.447 | 0.1429 | No |
| 127 | IL18BP |  |  | 5101 | -1.517 | 0.1356 | No |
| 128 | HDGF |  |  | 5136 | -1.545 | 0.1355 | No |
| 129 | KL |  |  | 5203 | -1.597 | 0.1310 | No |
| 130 | NID2 |  |  | 5234 | -1.622 | 0.1317 | No |
| 131 | MMP7 |  |  | 5262 | -1.642 | 0.1329 | No |
| 132 | CDA |  |  | 5351 | -1.734 | 0.1256 | No |
| 133 | SDF2 |  |  | 5391 | -1.785 | 0.1255 | No |
| 134 | SPINT1 |  |  | 5537 | -1.935 | 0.1107 | No |
| 135 | EFNA5 |  |  | 5588 | -1.991 | 0.1096 | No |
| 136 | ADAMTS9 |  |  | 5598 | -1.998 | 0.1145 | No |
| 137 | ANGPTL1 |  |  | 5702 | -2.151 | 0.1064 | No |
| 138 | FIGF |  |  | 6015 | -2.530 | 0.0693 | No |
| 139 | TINAG |  |  | 6242 | -2.885 | 0.0457 | No |
| 140 | PLA2G4B |  |  | 6322 | -3.027 | 0.0438 | No |
| 141 | ORM1 |  |  | 6398 | -3.186 | 0.0428 | No |
| 142 | ORM2 |  |  | 6469 | -3.363 | 0.0432 | No |
| 143 | CTF1 |  |  | 6770 | -4.290 | 0.0133 | No |
| 144 | APOA1BP |  |  | 6850 | -4.694 | 0.0165 | No |
| 145 | IDE |  |  | 6976 | -5.813 | 0.0166 | No |
Table: GSEA details [plain text format]

  

Fig 2: EXTRACELLULAR\_REGION: Random ES distribution      
 Gene set null distribution of ES for **EXTRACELLULAR\_REGION**

  
