# Supplementary material for: Network-based integration of molecular and physiological data elucidates regulatory mechanisms underlying adaptation to high-fat diet
Source: Genes Nutr. 2015 May 28;10(4):22. doi: 10.1007/s12263-015-0470-6 (PMC4446272; doi:10.1007/s12263-015-0470-6)
Supplement: Supplementary file 4 — Supplementary material 4 (ZIP 6984 kb) [file 12263_2015_470_MOESM4_ESM.zip › HF LF 12 w GSEA result/EXTRACELLULAR_REGION_PART.html]

Details for gene set EXTRACELLULAR\_REGION\_PART[GSEA]

|  || Dataset | HF LF 12w\_collapsed |
| Phenotype | NoPhenotypeAvailable |
| Upregulated in class | na\_pos |
| GeneSet | EXTRACELLULAR\_REGION\_PART |
| Enrichment Score (ES) | 0.5742433 |
| Normalized Enrichment Score (NES) | 2.5932522 |
| Nominal p-value | 0.0 |
| FDR q-value | 0.0 |
| FWER p-Value | 0.0 |
Table: GSEA Results Summary

  

Fig 1: Enrichment plot: EXTRACELLULAR\_REGION\_PART      
 Profile of the Running ES Score & Positions of GeneSet Members on the Rank Ordered List

  

| PROBE | GENE SYMBOL | GENE\_TITLE | RANK IN GENE LIST | RANK METRIC SCORE | RUNNING ES | CORE ENRICHMENT || 1 | SFRP4 |  |  | 5 | 8.683 | 0.0321 | Yes |
| 2 | CCL7 |  |  | 17 | 7.530 | 0.0590 | Yes |
| 3 | DST |  |  | 26 | 7.072 | 0.0845 | Yes |
| 4 | LOXL2 |  |  | 53 | 6.171 | 0.1041 | Yes |
| 5 | CD248 |  |  | 59 | 6.073 | 0.1263 | Yes |
| 6 | SLIT3 |  |  | 60 | 6.052 | 0.1492 | Yes |
| 7 | TNFAIP2 |  |  | 66 | 5.939 | 0.1709 | Yes |
| 8 | EFEMP2 |  |  | 90 | 5.433 | 0.1881 | Yes |
| 9 | C2 |  |  | 103 | 5.276 | 0.2063 | Yes |
| 10 | EDN1 |  |  | 136 | 4.966 | 0.2205 | Yes |
| 11 | CALCA |  |  | 138 | 4.957 | 0.2391 | Yes |
| 12 | PTHLH |  |  | 145 | 4.909 | 0.2568 | Yes |
| 13 | CCL2 |  |  | 148 | 4.900 | 0.2750 | Yes |
| 14 | FBLN2 |  |  | 162 | 4.842 | 0.2914 | Yes |
| 15 | ECM1 |  |  | 173 | 4.694 | 0.3077 | Yes |
| 16 | LBP |  |  | 176 | 4.677 | 0.3251 | Yes |
| 17 | TGFBI |  |  | 181 | 4.636 | 0.3421 | Yes |
| 18 | CPB2 |  |  | 197 | 4.579 | 0.3572 | Yes |
| 19 | FBN1 |  |  | 210 | 4.504 | 0.3725 | Yes |
| 20 | LEP |  |  | 227 | 4.387 | 0.3868 | Yes |
| 21 | COL1A2 |  |  | 243 | 4.294 | 0.4009 | Yes |
| 22 | C1QB |  |  | 264 | 4.144 | 0.4136 | Yes |
| 23 | PVR |  |  | 284 | 4.051 | 0.4262 | Yes |
| 24 | ADM |  |  | 301 | 3.945 | 0.4388 | Yes |
| 25 | COL6A3 |  |  | 308 | 3.880 | 0.4526 | Yes |
| 26 | GPC1 |  |  | 322 | 3.844 | 0.4653 | Yes |
| 27 | SGCD |  |  | 332 | 3.795 | 0.4783 | Yes |
| 28 | FGF2 |  |  | 348 | 3.718 | 0.4902 | Yes |
| 29 | C1QA |  |  | 376 | 3.596 | 0.4999 | Yes |
| 30 | LGALS7 |  |  | 448 | 3.322 | 0.5023 | Yes |
| 31 | COL5A1 |  |  | 473 | 3.215 | 0.5110 | Yes |
| 32 | CCL4 |  |  | 486 | 3.157 | 0.5212 | Yes |
| 33 | PCSK5 |  |  | 489 | 3.153 | 0.5328 | Yes |
| 34 | PCSK2 |  |  | 588 | 2.849 | 0.5296 | Yes |
| 35 | LTBP4 |  |  | 610 | 2.794 | 0.5371 | Yes |
| 36 | EBI3 |  |  | 654 | 2.682 | 0.5411 | Yes |
| 37 | LAMB2 |  |  | 679 | 2.625 | 0.5475 | Yes |
| 38 | IL27 |  |  | 684 | 2.615 | 0.5569 | Yes |
| 39 | PCSK1N |  |  | 724 | 2.521 | 0.5608 | Yes |
| 40 | COL8A1 |  |  | 753 | 2.451 | 0.5660 | Yes |
| 41 | SPN |  |  | 761 | 2.439 | 0.5742 | Yes |
| 42 | GPX3 |  |  | 930 | 2.136 | 0.5582 | No |
| 43 | FMOD |  |  | 967 | 2.091 | 0.5610 | No |
| 44 | CTRL |  |  | 991 | 2.050 | 0.5654 | No |
| 45 | CTGF |  |  | 1033 | 1.994 | 0.5671 | No |
| 46 | HBEGF |  |  | 1139 | 1.848 | 0.5590 | No |
| 47 | PSAP |  |  | 1179 | 1.785 | 0.5602 | No |
| 48 | COL3A1 |  |  | 1222 | 1.732 | 0.5607 | No |
| 49 | NOG |  |  | 1310 | 1.638 | 0.5544 | No |
| 50 | LUM |  |  | 1357 | 1.582 | 0.5538 | No |
| 51 | CXCL9 |  |  | 1395 | 1.543 | 0.5543 | No |
| 52 | COL15A1 |  |  | 1506 | 1.416 | 0.5439 | No |
| 53 | MMP2 |  |  | 1524 | 1.406 | 0.5468 | No |
| 54 | COMP |  |  | 1589 | 1.335 | 0.5426 | No |
| 55 | LAMA4 |  |  | 1664 | 1.240 | 0.5367 | No |
| 56 | KLK8 |  |  | 1750 | 1.156 | 0.5289 | No |
| 57 | RAB35 |  |  | 1759 | 1.139 | 0.5320 | No |
| 58 | DKKL1 |  |  | 1793 | 1.100 | 0.5315 | No |
| 59 | SNTB1 |  |  | 1934 | 0.925 | 0.5149 | No |
| 60 | FBLN1 |  |  | 1980 | 0.877 | 0.5117 | No |
| 61 | ANGPT2 |  |  | 1999 | 0.859 | 0.5124 | No |
| 62 | IL16 |  |  | 2050 | 0.812 | 0.5083 | No |
| 63 | TGFB1 |  |  | 2164 | 0.718 | 0.4948 | No |
| 64 | NUCB2 |  |  | 2260 | 0.637 | 0.4836 | No |
| 65 | SNTB2 |  |  | 2357 | 0.546 | 0.4719 | No |
| 66 | FLT1 |  |  | 2449 | 0.451 | 0.4606 | No |
| 67 | INHA |  |  | 2493 | 0.417 | 0.4560 | No |
| 68 | CFH |  |  | 2652 | 0.275 | 0.4344 | No |
| 69 | FGG |  |  | 2668 | 0.261 | 0.4332 | No |
| 70 | APOA1 |  |  | 2721 | 0.230 | 0.4266 | No |
| 71 | COPA |  |  | 2729 | 0.224 | 0.4265 | No |
| 72 | HYAL1 |  |  | 2737 | 0.220 | 0.4263 | No |
| 73 | INHBA |  |  | 2769 | 0.194 | 0.4226 | No |
| 74 | FXYD6 |  |  | 2902 | 0.078 | 0.4039 | No |
| 75 | NPY |  |  | 2955 | 0.042 | 0.3967 | No |
| 76 | FGL2 |  |  | 2956 | 0.041 | 0.3968 | No |
| 77 | COL4A2 |  |  | 3059 | -0.039 | 0.3823 | No |
| 78 | RNH1 |  |  | 3125 | -0.084 | 0.3733 | No |
| 79 | FGB |  |  | 3145 | -0.096 | 0.3710 | No |
| 80 | FGF10 |  |  | 3147 | -0.097 | 0.3712 | No |
| 81 | MMP9 |  |  | 3178 | -0.120 | 0.3673 | No |
| 82 | RTN3 |  |  | 3227 | -0.152 | 0.3610 | No |
| 83 | IL15 |  |  | 3302 | -0.200 | 0.3512 | No |
| 84 | NUCB1 |  |  | 3639 | -0.443 | 0.3047 | No |
| 85 | MYOC |  |  | 3658 | -0.457 | 0.3038 | No |
| 86 | COL5A3 |  |  | 3685 | -0.475 | 0.3019 | No |
| 87 | DMD |  |  | 3717 | -0.496 | 0.2993 | No |
| 88 | LIPE |  |  | 3816 | -0.564 | 0.2874 | No |
| 89 | CDH13 |  |  | 4038 | -0.728 | 0.2585 | No |
| 90 | CHAD |  |  | 4085 | -0.762 | 0.2548 | No |
| 91 | IK |  |  | 4228 | -0.857 | 0.2377 | No |
| 92 | MAGEE1 |  |  | 4270 | -0.891 | 0.2351 | No |
| 93 | CD5L |  |  | 4293 | -0.908 | 0.2354 | No |
| 94 | WFDC12 |  |  | 4317 | -0.923 | 0.2356 | No |
| 95 | COL4A3 |  |  | 4340 | -0.943 | 0.2360 | No |
| 96 | MGP |  |  | 4369 | -0.965 | 0.2356 | No |
| 97 | LGALS3BP |  |  | 4377 | -0.971 | 0.2383 | No |
| 98 | CRISP1 |  |  | 4427 | -1.008 | 0.2351 | No |
| 99 | SECTM1 |  |  | 4442 | -1.017 | 0.2369 | No |
| 100 | VTN |  |  | 4496 | -1.059 | 0.2333 | No |
| 101 | IGFBP1 |  |  | 4662 | -1.170 | 0.2141 | No |
| 102 | ANGPTL3 |  |  | 4934 | -1.372 | 0.1804 | No |
| 103 | IGFALS |  |  | 5017 | -1.447 | 0.1741 | No |
| 104 | HDGF |  |  | 5136 | -1.545 | 0.1631 | No |
| 105 | KL |  |  | 5203 | -1.597 | 0.1596 | No |
| 106 | NID2 |  |  | 5234 | -1.622 | 0.1615 | No |
| 107 | MMP7 |  |  | 5262 | -1.642 | 0.1638 | No |
| 108 | SDF2 |  |  | 5391 | -1.785 | 0.1522 | No |
| 109 | EFNA5 |  |  | 5588 | -1.991 | 0.1316 | No |
| 110 | ADAMTS9 |  |  | 5598 | -1.998 | 0.1379 | No |
| 111 | ANGPTL1 |  |  | 5702 | -2.151 | 0.1312 | No |
| 112 | FIGF |  |  | 6015 | -2.530 | 0.0961 | No |
| 113 | TINAG |  |  | 6242 | -2.885 | 0.0746 | No |
| 114 | ORM1 |  |  | 6398 | -3.186 | 0.0644 | No |
| 115 | ORM2 |  |  | 6469 | -3.363 | 0.0671 | No |
| 116 | IDE |  |  | 6976 | -5.813 | 0.0165 | No |
Table: GSEA details [plain text format]

  

Fig 2: EXTRACELLULAR\_REGION\_PART: Random ES distribution      
 Gene set null distribution of ES for **EXTRACELLULAR\_REGION\_PART**

  
