# Supplementary material for: Network-based integration of molecular and physiological data elucidates regulatory mechanisms underlying adaptation to high-fat diet
Source: Genes Nutr. 2015 May 28;10(4):22. doi: 10.1007/s12263-015-0470-6 (PMC4446272; doi:10.1007/s12263-015-0470-6)
Supplement: Supplementary file 4 — Supplementary material 4 (ZIP 6984 kb) [file 12263_2015_470_MOESM4_ESM.zip › HF LF 12 w GSEA result/EXTRACELLULAR_SPACE.html]

Details for gene set EXTRACELLULAR\_SPACE[GSEA]

|  || Dataset | HF LF 12w\_collapsed |
| Phenotype | NoPhenotypeAvailable |
| Upregulated in class | na\_pos |
| GeneSet | EXTRACELLULAR\_SPACE |
| Enrichment Score (ES) | 0.5759436 |
| Normalized Enrichment Score (NES) | 2.4576156 |
| Nominal p-value | 0.0 |
| FDR q-value | 0.0 |
| FWER p-Value | 0.0 |
Table: GSEA Results Summary

  

Fig 1: Enrichment plot: EXTRACELLULAR\_SPACE      
 Profile of the Running ES Score & Positions of GeneSet Members on the Rank Ordered List

  

| PROBE | GENE SYMBOL | GENE\_TITLE | RANK IN GENE LIST | RANK METRIC SCORE | RUNNING ES | CORE ENRICHMENT || 1 | SFRP4 |  |  | 5 | 8.683 | 0.0454 | Yes |
| 2 | CCL7 |  |  | 17 | 7.530 | 0.0838 | Yes |
| 3 | LOXL2 |  |  | 53 | 6.171 | 0.1115 | Yes |
| 4 | SLIT3 |  |  | 60 | 6.052 | 0.1428 | Yes |
| 5 | TNFAIP2 |  |  | 66 | 5.939 | 0.1736 | Yes |
| 6 | C2 |  |  | 103 | 5.276 | 0.1965 | Yes |
| 7 | EDN1 |  |  | 136 | 4.966 | 0.2183 | Yes |
| 8 | CALCA |  |  | 138 | 4.957 | 0.2445 | Yes |
| 9 | PTHLH |  |  | 145 | 4.909 | 0.2697 | Yes |
| 10 | CCL2 |  |  | 148 | 4.900 | 0.2954 | Yes |
| 11 | LBP |  |  | 176 | 4.677 | 0.3164 | Yes |
| 12 | TGFBI |  |  | 181 | 4.636 | 0.3404 | Yes |
| 13 | CPB2 |  |  | 197 | 4.579 | 0.3626 | Yes |
| 14 | FBN1 |  |  | 210 | 4.504 | 0.3848 | Yes |
| 15 | LEP |  |  | 227 | 4.387 | 0.4058 | Yes |
| 16 | C1QB |  |  | 264 | 4.144 | 0.4226 | Yes |
| 17 | PVR |  |  | 284 | 4.051 | 0.4414 | Yes |
| 18 | ADM |  |  | 301 | 3.945 | 0.4601 | Yes |
| 19 | GPC1 |  |  | 322 | 3.844 | 0.4776 | Yes |
| 20 | FGF2 |  |  | 348 | 3.718 | 0.4938 | Yes |
| 21 | C1QA |  |  | 376 | 3.596 | 0.5090 | Yes |
| 22 | LGALS7 |  |  | 448 | 3.322 | 0.5165 | Yes |
| 23 | CCL4 |  |  | 486 | 3.157 | 0.5280 | Yes |
| 24 | PCSK5 |  |  | 489 | 3.153 | 0.5445 | Yes |
| 25 | PCSK2 |  |  | 588 | 2.849 | 0.5456 | Yes |
| 26 | EBI3 |  |  | 654 | 2.682 | 0.5506 | Yes |
| 27 | IL27 |  |  | 684 | 2.615 | 0.5603 | Yes |
| 28 | PCSK1N |  |  | 724 | 2.521 | 0.5681 | Yes |
| 29 | SPN |  |  | 761 | 2.439 | 0.5759 | Yes |
| 30 | GPX3 |  |  | 930 | 2.136 | 0.5633 | No |
| 31 | CTRL |  |  | 991 | 2.050 | 0.5656 | No |
| 32 | HBEGF |  |  | 1139 | 1.848 | 0.5545 | No |
| 33 | PSAP |  |  | 1179 | 1.785 | 0.5584 | No |
| 34 | NOG |  |  | 1310 | 1.638 | 0.5485 | No |
| 35 | CXCL9 |  |  | 1395 | 1.543 | 0.5447 | No |
| 36 | MMP2 |  |  | 1524 | 1.406 | 0.5339 | No |
| 37 | KLK8 |  |  | 1750 | 1.156 | 0.5080 | No |
| 38 | DKKL1 |  |  | 1793 | 1.100 | 0.5078 | No |
| 39 | FBLN1 |  |  | 1980 | 0.877 | 0.4859 | No |
| 40 | ANGPT2 |  |  | 1999 | 0.859 | 0.4879 | No |
| 41 | IL16 |  |  | 2050 | 0.812 | 0.4851 | No |
| 42 | TGFB1 |  |  | 2164 | 0.718 | 0.4728 | No |
| 43 | NUCB2 |  |  | 2260 | 0.637 | 0.4626 | No |
| 44 | FLT1 |  |  | 2449 | 0.451 | 0.4382 | No |
| 45 | INHA |  |  | 2493 | 0.417 | 0.4343 | No |
| 46 | CFH |  |  | 2652 | 0.275 | 0.4132 | No |
| 47 | FGG |  |  | 2668 | 0.261 | 0.4124 | No |
| 48 | APOA1 |  |  | 2721 | 0.230 | 0.4062 | No |
| 49 | COPA |  |  | 2729 | 0.224 | 0.4064 | No |
| 50 | HYAL1 |  |  | 2737 | 0.220 | 0.4066 | No |
| 51 | INHBA |  |  | 2769 | 0.194 | 0.4032 | No |
| 52 | FXYD6 |  |  | 2902 | 0.078 | 0.3848 | No |
| 53 | NPY |  |  | 2955 | 0.042 | 0.3776 | No |
| 54 | FGL2 |  |  | 2956 | 0.041 | 0.3778 | No |
| 55 | FGB |  |  | 3145 | -0.096 | 0.3515 | No |
| 56 | FGF10 |  |  | 3147 | -0.097 | 0.3518 | No |
| 57 | MMP9 |  |  | 3178 | -0.120 | 0.3482 | No |
| 58 | RTN3 |  |  | 3227 | -0.152 | 0.3422 | No |
| 59 | IL15 |  |  | 3302 | -0.200 | 0.3327 | No |
| 60 | NUCB1 |  |  | 3639 | -0.443 | 0.2871 | No |
| 61 | MYOC |  |  | 3658 | -0.457 | 0.2869 | No |
| 62 | LIPE |  |  | 3816 | -0.564 | 0.2675 | No |
| 63 | CDH13 |  |  | 4038 | -0.728 | 0.2399 | No |
| 64 | IK |  |  | 4228 | -0.857 | 0.2174 | No |
| 65 | CD5L |  |  | 4293 | -0.908 | 0.2131 | No |
| 66 | WFDC12 |  |  | 4317 | -0.923 | 0.2147 | No |
| 67 | LGALS3BP |  |  | 4377 | -0.971 | 0.2115 | No |
| 68 | CRISP1 |  |  | 4427 | -1.008 | 0.2098 | No |
| 69 | SECTM1 |  |  | 4442 | -1.017 | 0.2132 | No |
| 70 | VTN |  |  | 4496 | -1.059 | 0.2113 | No |
| 71 | IGFBP1 |  |  | 4662 | -1.170 | 0.1940 | No |
| 72 | ANGPTL3 |  |  | 4934 | -1.372 | 0.1626 | No |
| 73 | IGFALS |  |  | 5017 | -1.447 | 0.1586 | No |
| 74 | HDGF |  |  | 5136 | -1.545 | 0.1499 | No |
| 75 | KL |  |  | 5203 | -1.597 | 0.1490 | No |
| 76 | MMP7 |  |  | 5262 | -1.642 | 0.1494 | No |
| 77 | SDF2 |  |  | 5391 | -1.785 | 0.1407 | No |
| 78 | EFNA5 |  |  | 5588 | -1.991 | 0.1233 | No |
| 79 | ANGPTL1 |  |  | 5702 | -2.151 | 0.1186 | No |
| 80 | FIGF |  |  | 6015 | -2.530 | 0.0875 | No |
| 81 | ORM1 |  |  | 6398 | -3.186 | 0.0499 | No |
| 82 | ORM2 |  |  | 6469 | -3.363 | 0.0577 | No |
| 83 | IDE |  |  | 6976 | -5.813 | 0.0164 | No |
Table: GSEA details [plain text format]

  

Fig 2: EXTRACELLULAR\_SPACE: Random ES distribution      
 Gene set null distribution of ES for **EXTRACELLULAR\_SPACE**

  
