# Supplementary material for: Network-based integration of molecular and physiological data elucidates regulatory mechanisms underlying adaptation to high-fat diet
Source: Genes Nutr. 2015 May 28;10(4):22. doi: 10.1007/s12263-015-0470-6 (PMC4446272; doi:10.1007/s12263-015-0470-6)
Supplement: Supplementary file 4 — Supplementary material 4 (ZIP 6984 kb) [file 12263_2015_470_MOESM4_ESM.zip › HF LF 12 w GSEA result/gsea_report_for_na_neg_1365169146545.html]

Report for na\_neg 1365169146545 [GSEA]

| GS  follow link to MSigDB | GS DETAILS | SIZE | ES | NES | NOM p-val | FDR q-val | FWER p-val | RANK AT MAX | LEADING EDGE || 1 | MITOCHONDRION | Details ... | 153 | -0.60 | -2.56 | 0.000 | 0.000 | 0.000 | 1314 | tags=56%, list=19%, signal=67% |
| 2 | MITOCHONDRIAL\_PART | Details ... | 67 | -0.65 | -2.47 | 0.000 | 0.000 | 0.000 | 1302 | tags=64%, list=18%, signal=78% |
| 3 | MITOCHONDRIAL\_INNER\_MEMBRANE | Details ... | 31 | -0.75 | -2.41 | 0.000 | 0.000 | 0.000 | 1279 | tags=77%, list=18%, signal=94% |
| 4 | ORGANELLE\_INNER\_MEMBRANE | Details ... | 35 | -0.70 | -2.32 | 0.000 | 0.000 | 0.000 | 656 | tags=57%, list=9%, signal=63% |
| 5 | MITOCHONDRIAL\_MEMBRANE\_PART | Details ... | 24 | -0.77 | -2.29 | 0.000 | 0.000 | 0.000 | 1279 | tags=83%, list=18%, signal=101% |
| 6 | MITOCHONDRIAL\_MEMBRANE | Details ... | 38 | -0.67 | -2.29 | 0.000 | 0.000 | 0.000 | 712 | tags=58%, list=10%, signal=64% |
| 7 | MITOCHONDRIAL\_ENVELOPE | Details ... | 44 | -0.65 | -2.28 | 0.000 | 0.000 | 0.000 | 1279 | tags=66%, list=18%, signal=80% |
| 8 | MICROBODY | Details ... | 25 | -0.73 | -2.27 | 0.000 | 0.000 | 0.000 | 1575 | tags=72%, list=22%, signal=92% |
| 9 | PEROXISOME | Details ... | 25 | -0.73 | -2.23 | 0.000 | 0.000 | 0.000 | 1575 | tags=72%, list=22%, signal=92% |
| 10 | ORGANELLE\_ENVELOPE | Details ... | 75 | -0.57 | -2.18 | 0.000 | 0.000 | 0.000 | 1279 | tags=51%, list=18%, signal=61% |
| 11 | OXIDOREDUCTASE\_ACTIVITY\_ACTING\_ON\_CH\_OH\_GROUP\_OF\_DONORS | Details ... | 20 | -0.76 | -2.16 | 0.000 | 0.000 | 0.002 | 513 | tags=55%, list=7%, signal=59% |
| 12 | ENVELOPE | Details ... | 75 | -0.57 | -2.16 | 0.000 | 0.000 | 0.003 | 1279 | tags=51%, list=18%, signal=61% |
| 13 | MITOCHONDRIAL\_LUMEN | Details ... | 24 | -0.68 | -2.09 | 0.000 | 0.000 | 0.008 | 1534 | tags=71%, list=22%, signal=90% |
| 14 | OXIDOREDUCTASE\_ACTIVITY | Details ... | 112 | -0.51 | -2.07 | 0.000 | 0.001 | 0.011 | 860 | tags=38%, list=12%, signal=42% |
| 15 | MITOCHONDRIAL\_MATRIX | Details ... | 24 | -0.68 | -2.06 | 0.000 | 0.001 | 0.012 | 1534 | tags=71%, list=22%, signal=90% |
| 16 | OXIDOREDUCTASE\_ACTIVITY\_GO\_0016616 | Details ... | 18 | -0.71 | -1.98 | 0.002 | 0.002 | 0.036 | 820 | tags=56%, list=12%, signal=63% |
| 17 | COFACTOR\_METABOLIC\_PROCESS | Details ... | 18 | -0.68 | -1.93 | 0.000 | 0.004 | 0.083 | 646 | tags=50%, list=9%, signal=55% |
| 18 | LYASE\_ACTIVITY | Details ... | 27 | -0.62 | -1.91 | 0.002 | 0.004 | 0.111 | 1527 | tags=56%, list=22%, signal=71% |
| 19 | ORGANIC\_ACID\_METABOLIC\_PROCESS | Details ... | 65 | -0.50 | -1.89 | 0.000 | 0.006 | 0.147 | 725 | tags=31%, list=10%, signal=34% |
| 20 | TRANSFERASE\_ACTIVITY\_TRANSFERRING\_ALKYL\_OR\_ARYLOTHER\_THAN\_METHYLGROUPS | Details ... | 16 | -0.68 | -1.87 | 0.000 | 0.007 | 0.193 | 340 | tags=25%, list=5%, signal=26% |
| 21 | ORGANELLE\_MEMBRANE |  | 133 | -0.44 | -1.85 | 0.000 | 0.008 | 0.230 | 1312 | tags=35%, list=18%, signal=42% |
| 22 | CARBOXYLIC\_ACID\_METABOLIC\_PROCESS |  | 64 | -0.49 | -1.83 | 0.000 | 0.010 | 0.272 | 725 | tags=30%, list=10%, signal=33% |
| 23 | ELECTRON\_CARRIER\_ACTIVITY |  | 27 | -0.58 | -1.81 | 0.002 | 0.013 | 0.344 | 572 | tags=52%, list=8%, signal=56% |
| 24 | CARBOHYDRATE\_METABOLIC\_PROCESS |  | 53 | -0.50 | -1.78 | 0.000 | 0.018 | 0.447 | 821 | tags=34%, list=12%, signal=38% |
| 25 | MITOCHONDRION\_ORGANIZATION\_AND\_BIOGENESIS |  | 21 | -0.61 | -1.76 | 0.002 | 0.021 | 0.524 | 656 | tags=48%, list=9%, signal=52% |
| 26 | GENERATION\_OF\_PRECURSOR\_METABOLITES\_AND\_ENERGY |  | 47 | -0.50 | -1.74 | 0.002 | 0.028 | 0.629 | 528 | tags=32%, list=7%, signal=34% |
| 27 | DNA\_DEPENDENT\_DNA\_REPLICATION |  | 20 | -0.59 | -1.67 | 0.005 | 0.053 | 0.846 | 1870 | tags=60%, list=26%, signal=81% |
| 28 | ORGANELLE\_LUMEN |  | 155 | -0.39 | -1.66 | 0.001 | 0.055 | 0.859 | 2246 | tags=47%, list=32%, signal=67% |
| 29 | MEMBRANE\_ENCLOSED\_LUMEN |  | 155 | -0.39 | -1.65 | 0.000 | 0.061 | 0.900 | 2246 | tags=47%, list=32%, signal=67% |
| 30 | LIGASE\_ACTIVITY |  | 48 | -0.47 | -1.64 | 0.005 | 0.070 | 0.934 | 2007 | tags=48%, list=28%, signal=66% |
| 31 | INORGANIC\_CATION\_TRANSMEMBRANE\_TRANSPORTER\_ACTIVITY |  | 21 | -0.56 | -1.63 | 0.013 | 0.069 | 0.940 | 520 | tags=33%, list=7%, signal=36% |
| 32 | CELLULAR\_CARBOHYDRATE\_METABOLIC\_PROCESS |  | 38 | -0.48 | -1.61 | 0.013 | 0.085 | 0.979 | 821 | tags=34%, list=12%, signal=38% |
| 33 | ALCOHOL\_METABOLIC\_PROCESS |  | 35 | -0.48 | -1.60 | 0.014 | 0.089 | 0.984 | 821 | tags=26%, list=12%, signal=29% |
| 34 | ORGANELLE\_PART |  | 418 | -0.34 | -1.59 | 0.000 | 0.089 | 0.986 | 1934 | tags=35%, list=27%, signal=45% |
| 35 | INTRACELLULAR\_ORGANELLE\_PART |  | 415 | -0.34 | -1.59 | 0.000 | 0.090 | 0.990 | 1934 | tags=35%, list=27%, signal=45% |
| 36 | AMINO\_ACID\_METABOLIC\_PROCESS |  | 32 | -0.48 | -1.57 | 0.025 | 0.101 | 0.995 | 725 | tags=31%, list=10%, signal=35% |
| 37 | MONOCARBOXYLIC\_ACID\_METABOLIC\_PROCESS |  | 27 | -0.50 | -1.56 | 0.025 | 0.107 | 0.997 | 2121 | tags=52%, list=30%, signal=74% |
| 38 | AMINO\_ACID\_AND\_DERIVATIVE\_METABOLIC\_PROCESS |  | 43 | -0.46 | -1.55 | 0.008 | 0.111 | 0.998 | 725 | tags=26%, list=10%, signal=28% |
| 39 | AROMATIC\_COMPOUND\_METABOLIC\_PROCESS |  | 15 | -0.57 | -1.54 | 0.040 | 0.121 | 0.999 | 2328 | tags=53%, list=33%, signal=79% |
| 40 | PROTEIN\_FOLDING |  | 21 | -0.53 | -1.53 | 0.038 | 0.124 | 0.999 | 1262 | tags=38%, list=18%, signal=46% |
| 41 | FATTY\_ACID\_METABOLIC\_PROCESS |  | 21 | -0.52 | -1.51 | 0.047 | 0.146 | 1.000 | 1042 | tags=33%, list=15%, signal=39% |
| 42 | CELLULAR\_CATABOLIC\_PROCESS |  | 84 | -0.38 | -1.50 | 0.017 | 0.154 | 1.000 | 1940 | tags=43%, list=27%, signal=58% |
| 43 | MACROMOLECULE\_CATABOLIC\_PROCESS |  | 53 | -0.41 | -1.49 | 0.030 | 0.162 | 1.000 | 2007 | tags=47%, list=28%, signal=65% |
| 44 | CATABOLIC\_PROCESS |  | 87 | -0.38 | -1.49 | 0.012 | 0.163 | 1.000 | 1940 | tags=43%, list=27%, signal=58% |
| 45 | BIOPOLYMER\_CATABOLIC\_PROCESS |  | 48 | -0.41 | -1.42 | 0.048 | 0.254 | 1.000 | 2383 | tags=54%, list=34%, signal=81% |
| 46 | DNA\_REPAIR |  | 58 | -0.39 | -1.42 | 0.037 | 0.254 | 1.000 | 2448 | tags=52%, list=35%, signal=78% |
| 47 | RESPONSE\_TO\_DNA\_DAMAGE\_STIMULUS |  | 70 | -0.38 | -1.42 | 0.042 | 0.252 | 1.000 | 2448 | tags=49%, list=35%, signal=73% |
| 48 | NUCLEOTIDYLTRANSFERASE\_ACTIVITY |  | 21 | -0.47 | -1.38 | 0.083 | 0.317 | 1.000 | 1721 | tags=48%, list=24%, signal=63% |
| 49 | DNA\_METABOLIC\_PROCESS |  | 107 | -0.34 | -1.35 | 0.047 | 0.388 | 1.000 | 2151 | tags=46%, list=30%, signal=65% |
| 50 | LIPID\_METABOLIC\_PROCESS |  | 97 | -0.33 | -1.34 | 0.049 | 0.397 | 1.000 | 933 | tags=22%, list=13%, signal=25% |
| 51 | SENSORY\_PERCEPTION |  | 36 | -0.40 | -1.33 | 0.103 | 0.407 | 1.000 | 557 | tags=22%, list=8%, signal=24% |
| 52 | NITROGEN\_COMPOUND\_METABOLIC\_PROCESS |  | 63 | -0.35 | -1.31 | 0.083 | 0.458 | 1.000 | 767 | tags=24%, list=11%, signal=26% |
| 53 | ORGANELLE\_ORGANIZATION\_AND\_BIOGENESIS |  | 168 | -0.30 | -1.31 | 0.049 | 0.455 | 1.000 | 1329 | tags=26%, list=19%, signal=31% |
| 54 | CELLULAR\_BIOSYNTHETIC\_PROCESS |  | 112 | -0.32 | -1.31 | 0.086 | 0.448 | 1.000 | 767 | tags=21%, list=11%, signal=24% |
| 55 | CHROMATIN\_MODIFICATION |  | 16 | -0.48 | -1.29 | 0.150 | 0.481 | 1.000 | 1202 | tags=31%, list=17%, signal=38% |
| 56 | DNA\_DIRECTED\_RNA\_POLYMERASEII\_HOLOENZYME |  | 17 | -0.47 | -1.29 | 0.152 | 0.479 | 1.000 | 2246 | tags=53%, list=32%, signal=77% |
| 57 | CHROMOSOME |  | 41 | -0.39 | -1.29 | 0.132 | 0.475 | 1.000 | 1877 | tags=39%, list=26%, signal=53% |
| 58 | MACROMOLECULAR\_COMPLEX |  | 331 | -0.28 | -1.29 | 0.020 | 0.478 | 1.000 | 1669 | tags=28%, list=24%, signal=35% |
| 59 | PROTEIN\_TARGETING |  | 43 | -0.38 | -1.29 | 0.111 | 0.471 | 1.000 | 1726 | tags=35%, list=24%, signal=46% |
| 60 | RESPONSE\_TO\_OXIDATIVE\_STRESS |  | 17 | -0.47 | -1.28 | 0.178 | 0.473 | 1.000 | 681 | tags=35%, list=10%, signal=39% |
| 61 | RESPONSE\_TO\_ENDOGENOUS\_STIMULUS |  | 84 | -0.33 | -1.28 | 0.105 | 0.479 | 1.000 | 2448 | tags=45%, list=35%, signal=68% |
| 62 | NUCLEAR\_PART |  | 186 | -0.29 | -1.28 | 0.065 | 0.471 | 1.000 | 2307 | tags=39%, list=33%, signal=57% |
| 63 | CELLULAR\_MACROMOLECULE\_CATABOLIC\_PROCESS |  | 41 | -0.37 | -1.27 | 0.138 | 0.493 | 1.000 | 2007 | tags=44%, list=28%, signal=61% |
| 64 | DNA\_REPLICATION |  | 39 | -0.37 | -1.26 | 0.155 | 0.495 | 1.000 | 1870 | tags=49%, list=26%, signal=66% |
| 65 | PROTEIN\_COMPLEX |  | 291 | -0.27 | -1.26 | 0.044 | 0.501 | 1.000 | 1661 | tags=27%, list=23%, signal=34% |
| 66 | INTEGRAL\_TO\_ORGANELLE\_MEMBRANE |  | 23 | -0.42 | -1.26 | 0.170 | 0.493 | 1.000 | 730 | tags=26%, list=10%, signal=29% |
| 67 | STRUCTURE\_SPECIFIC\_DNA\_BINDING |  | 18 | -0.45 | -1.26 | 0.190 | 0.488 | 1.000 | 1870 | tags=44%, list=26%, signal=60% |
| 68 | RIBOSOME |  | 16 | -0.46 | -1.26 | 0.205 | 0.486 | 1.000 | 1534 | tags=56%, list=22%, signal=72% |
| 69 | PROTEIN\_IMPORT |  | 23 | -0.42 | -1.25 | 0.162 | 0.486 | 1.000 | 1726 | tags=39%, list=24%, signal=52% |
| 70 | AMINE\_METABOLIC\_PROCESS |  | 58 | -0.34 | -1.25 | 0.138 | 0.497 | 1.000 | 725 | tags=22%, list=10%, signal=25% |
| 71 | PORE\_COMPLEX |  | 17 | -0.45 | -1.24 | 0.206 | 0.510 | 1.000 | 1533 | tags=35%, list=22%, signal=45% |
| 72 | CELLULAR\_PROTEIN\_CATABOLIC\_PROCESS |  | 26 | -0.40 | -1.23 | 0.198 | 0.523 | 1.000 | 2383 | tags=54%, list=34%, signal=81% |
| 73 | PROTEIN\_CATABOLIC\_PROCESS |  | 28 | -0.39 | -1.23 | 0.198 | 0.534 | 1.000 | 2383 | tags=54%, list=34%, signal=80% |
| 74 | TRANSMEMBRANE\_TRANSPORTER\_ACTIVITY |  | 106 | -0.31 | -1.23 | 0.118 | 0.527 | 1.000 | 1593 | tags=35%, list=22%, signal=44% |
| 75 | LIGASE\_ACTIVITY\_FORMING\_CARBON\_NITROGEN\_BONDS |  | 36 | -0.37 | -1.22 | 0.195 | 0.535 | 1.000 | 2007 | tags=39%, list=28%, signal=54% |
| 76 | BIOSYNTHETIC\_PROCESS |  | 151 | -0.28 | -1.21 | 0.118 | 0.567 | 1.000 | 974 | tags=21%, list=14%, signal=24% |
| 77 | APOPTOTIC\_PROGRAM |  | 25 | -0.39 | -1.21 | 0.214 | 0.566 | 1.000 | 327 | tags=20%, list=5%, signal=21% |
| 78 | SUBSTRATE\_SPECIFIC\_TRANSMEMBRANE\_TRANSPORTER\_ACTIVITY |  | 96 | -0.30 | -1.19 | 0.167 | 0.593 | 1.000 | 1593 | tags=34%, list=22%, signal=44% |
| 79 | CELLULAR\_COMPONENT\_DISASSEMBLY |  | 15 | -0.44 | -1.19 | 0.241 | 0.585 | 1.000 | 1319 | tags=47%, list=19%, signal=57% |
| 80 | RIBONUCLEOPROTEIN\_COMPLEX |  | 48 | -0.34 | -1.19 | 0.214 | 0.586 | 1.000 | 1849 | tags=42%, list=26%, signal=56% |
| 81 | RNA\_BINDING |  | 80 | -0.30 | -1.17 | 0.195 | 0.633 | 1.000 | 2021 | tags=39%, list=28%, signal=54% |
| 82 | INTRINSIC\_TO\_ORGANELLE\_MEMBRANE |  | 25 | -0.39 | -1.17 | 0.236 | 0.655 | 1.000 | 730 | tags=24%, list=10%, signal=27% |
| 83 | INTRACELLULAR\_PROTEIN\_TRANSPORT |  | 58 | -0.31 | -1.16 | 0.226 | 0.653 | 1.000 | 1726 | tags=28%, list=24%, signal=36% |
| 84 | LIPID\_BIOSYNTHETIC\_PROCESS |  | 25 | -0.37 | -1.16 | 0.250 | 0.654 | 1.000 | 1178 | tags=28%, list=17%, signal=33% |
| 85 | NUCLEAR\_ENVELOPE |  | 32 | -0.34 | -1.15 | 0.235 | 0.679 | 1.000 | 1177 | tags=28%, list=17%, signal=34% |
| 86 | NUCLEAR\_LUMEN |  | 122 | -0.28 | -1.15 | 0.216 | 0.678 | 1.000 | 2246 | tags=40%, list=32%, signal=58% |
| 87 | TRANSLATION\_FACTOR\_ACTIVITY\_NUCLEIC\_ACID\_BINDING |  | 19 | -0.39 | -1.13 | 0.309 | 0.737 | 1.000 | 1631 | tags=37%, list=23%, signal=48% |
| 88 | RNA\_PROCESSING |  | 62 | -0.30 | -1.13 | 0.251 | 0.733 | 1.000 | 2380 | tags=37%, list=34%, signal=55% |
| 89 | RNA\_SPLICING |  | 34 | -0.34 | -1.11 | 0.313 | 0.786 | 1.000 | 3010 | tags=47%, list=42%, signal=81% |
| 90 | TRANSFERASE\_ACTIVITY\_TRANSFERRING\_GROUPS\_OTHER\_THAN\_AMINO\_ACYL\_GROUPS |  | 17 | -0.40 | -1.11 | 0.311 | 0.783 | 1.000 | 2357 | tags=65%, list=33%, signal=97% |
| 91 | CHROMOSOMAL\_PART |  | 36 | -0.33 | -1.10 | 0.341 | 0.816 | 1.000 | 1877 | tags=33%, list=26%, signal=45% |
| 92 | CELL\_CYCLE\_CHECKPOINT\_GO\_0000075 |  | 18 | -0.39 | -1.09 | 0.345 | 0.831 | 1.000 | 2307 | tags=44%, list=33%, signal=66% |
| 93 | PROTEIN\_N\_TERMINUS\_BINDING |  | 17 | -0.39 | -1.09 | 0.337 | 0.830 | 1.000 | 2307 | tags=53%, list=33%, signal=78% |
| 94 | CELLULAR\_LIPID\_METABOLIC\_PROCESS |  | 78 | -0.28 | -1.09 | 0.313 | 0.821 | 1.000 | 933 | tags=21%, list=13%, signal=23% |
| 95 | TRANSITION\_METAL\_ION\_BINDING |  | 36 | -0.33 | -1.09 | 0.335 | 0.822 | 1.000 | 694 | tags=19%, list=10%, signal=21% |
| 96 | HYDROLASE\_ACTIVITY\_ACTING\_ON\_CARBON\_NITROGEN\_NOT\_PEPTIDEBONDS |  | 20 | -0.37 | -1.08 | 0.313 | 0.834 | 1.000 | 1742 | tags=40%, list=25%, signal=53% |
| 97 | DNA\_RECOMBINATION |  | 17 | -0.39 | -1.08 | 0.352 | 0.830 | 1.000 | 2054 | tags=47%, list=29%, signal=66% |
| 98 | TRANSFERASE\_ACTIVITY\_TRANSFERRING\_ACYL\_GROUPS |  | 21 | -0.36 | -1.08 | 0.352 | 0.833 | 1.000 | 1789 | tags=48%, list=25%, signal=63% |
| 99 | ZINC\_ION\_BINDING |  | 30 | -0.34 | -1.07 | 0.365 | 0.839 | 1.000 | 694 | tags=20%, list=10%, signal=22% |
| 100 | ION\_TRANSMEMBRANE\_TRANSPORTER\_ACTIVITY |  | 70 | -0.28 | -1.07 | 0.336 | 0.850 | 1.000 | 1593 | tags=33%, list=22%, signal=42% |
| 101 | NUCLEOLUS |  | 35 | -0.32 | -1.07 | 0.373 | 0.845 | 1.000 | 2246 | tags=43%, list=32%, signal=62% |
| 102 | NUCLEASE\_ACTIVITY |  | 17 | -0.38 | -1.07 | 0.379 | 0.837 | 1.000 | 1940 | tags=41%, list=27%, signal=57% |
| 103 | METALLOPEPTIDASE\_ACTIVITY |  | 18 | -0.37 | -1.06 | 0.374 | 0.839 | 1.000 | 503 | tags=22%, list=7%, signal=24% |
| 104 | NUCLEOBASENUCLEOSIDENUCLEOTIDE\_AND\_NUCLEIC\_ACID\_METABOLIC\_PROCESS |  | 447 | -0.22 | -1.06 | 0.295 | 0.838 | 1.000 | 2429 | tags=39%, list=34%, signal=55% |
| 105 | REGULATION\_OF\_DNA\_METABOLIC\_PROCESS |  | 18 | -0.37 | -1.06 | 0.396 | 0.837 | 1.000 | 2425 | tags=56%, list=34%, signal=84% |
| 106 | TRANSLATION\_REGULATOR\_ACTIVITY |  | 20 | -0.37 | -1.05 | 0.394 | 0.841 | 1.000 | 1631 | tags=35%, list=23%, signal=45% |
| 107 | NUCLEAR\_BODY |  | 15 | -0.39 | -1.05 | 0.387 | 0.834 | 1.000 | 1870 | tags=47%, list=26%, signal=63% |
| 108 | PHOSPHOLIPID\_METABOLIC\_PROCESS |  | 23 | -0.35 | -1.05 | 0.390 | 0.840 | 1.000 | 773 | tags=17%, list=11%, signal=19% |
| 109 | MRNA\_METABOLIC\_PROCESS |  | 32 | -0.32 | -1.05 | 0.398 | 0.843 | 1.000 | 524 | tags=13%, list=7%, signal=13% |
| 110 | CARBOHYDRATE\_BIOSYNTHETIC\_PROCESS |  | 15 | -0.39 | -1.04 | 0.400 | 0.859 | 1.000 | 687 | tags=27%, list=10%, signal=29% |
| 111 | REGULATION\_OF\_BINDING |  | 20 | -0.36 | -1.04 | 0.408 | 0.868 | 1.000 | 692 | tags=20%, list=10%, signal=22% |
| 112 | OXIDOREDUCTASE\_ACTIVITY\_GO\_0016705 |  | 15 | -0.39 | -1.03 | 0.421 | 0.881 | 1.000 | 78 | tags=13%, list=1%, signal=13% |
| 113 | NUCLEOPLASM\_PART |  | 69 | -0.27 | -1.03 | 0.389 | 0.877 | 1.000 | 2246 | tags=42%, list=32%, signal=61% |
| 114 | NEGATIVE\_REGULATION\_OF\_NUCLEOBASENUCLEOSIDENUCLEOTIDE\_AND\_NUCLEIC\_ACID\_METABOLIC\_PROCESS |  | 67 | -0.27 | -1.03 | 0.414 | 0.873 | 1.000 | 1860 | tags=30%, list=26%, signal=40% |
| 115 | ENDOPEPTIDASE\_ACTIVITY |  | 36 | -0.31 | -1.03 | 0.407 | 0.866 | 1.000 | 1358 | tags=28%, list=19%, signal=34% |
| 116 | MRNA\_PROCESSING\_GO\_0006397 |  | 27 | -0.33 | -1.03 | 0.412 | 0.863 | 1.000 | 3390 | tags=63%, list=48%, signal=120% |
| 117 | ACID\_AMINO\_ACID\_LIGASE\_ACTIVITY |  | 29 | -0.33 | -1.02 | 0.445 | 0.881 | 1.000 | 2007 | tags=34%, list=28%, signal=48% |
| 118 | CYTOSOL |  | 73 | -0.27 | -1.02 | 0.419 | 0.874 | 1.000 | 1915 | tags=30%, list=27%, signal=41% |
| 119 | PROTEIN\_TRANSPORT |  | 62 | -0.27 | -1.02 | 0.417 | 0.867 | 1.000 | 2343 | tags=35%, list=33%, signal=53% |
| 120 | SUBSTRATE\_SPECIFIC\_TRANSPORTER\_ACTIVITY |  | 116 | -0.25 | -1.02 | 0.440 | 0.860 | 1.000 | 1593 | tags=29%, list=22%, signal=37% |
| 121 | PHOSPHORIC\_MONOESTER\_HYDROLASE\_ACTIVITY |  | 41 | -0.29 | -1.01 | 0.436 | 0.868 | 1.000 | 1476 | tags=27%, list=21%, signal=34% |
| 122 | UNFOLDED\_PROTEIN\_BINDING |  | 16 | -0.37 | -1.01 | 0.461 | 0.866 | 1.000 | 1409 | tags=25%, list=20%, signal=31% |
| 123 | PROTEIN\_RNA\_COMPLEX\_ASSEMBLY |  | 16 | -0.36 | -1.01 | 0.428 | 0.865 | 1.000 | 620 | tags=19%, list=9%, signal=20% |
| 124 | NON\_MEMBRANE\_BOUND\_ORGANELLE |  | 197 | -0.23 | -1.01 | 0.428 | 0.860 | 1.000 | 1922 | tags=31%, list=27%, signal=41% |
| 125 | INTRACELLULAR\_NON\_MEMBRANE\_BOUND\_ORGANELLE |  | 197 | -0.23 | -1.00 | 0.481 | 0.877 | 1.000 | 1922 | tags=31%, list=27%, signal=41% |
| 126 | NUCLEAR\_PORE |  | 16 | -0.37 | -1.00 | 0.450 | 0.873 | 1.000 | 1533 | tags=31%, list=22%, signal=40% |
| 127 | NEGATIVE\_REGULATION\_OF\_TRANSCRIPTION\_DNA\_DEPENDENT |  | 41 | -0.29 | -1.00 | 0.468 | 0.872 | 1.000 | 1291 | tags=24%, list=18%, signal=30% |
| 128 | NUCLEUS |  | 485 | -0.21 | -1.00 | 0.496 | 0.876 | 1.000 | 2425 | tags=37%, list=34%, signal=52% |
| 129 | CELLULAR\_COMPONENT\_ASSEMBLY |  | 109 | -0.24 | -0.99 | 0.482 | 0.877 | 1.000 | 800 | tags=15%, list=11%, signal=16% |
| 130 | NUCLEOPLASM |  | 91 | -0.25 | -0.99 | 0.492 | 0.881 | 1.000 | 2246 | tags=40%, list=32%, signal=57% |
| 131 | ESTABLISHMENT\_AND\_OR\_MAINTENANCE\_OF\_CHROMATIN\_ARCHITECTURE |  | 27 | -0.31 | -0.98 | 0.494 | 0.893 | 1.000 | 2372 | tags=48%, list=33%, signal=72% |
| 132 | CHROMOSOME\_ORGANIZATION\_AND\_BIOGENESIS |  | 45 | -0.28 | -0.98 | 0.490 | 0.898 | 1.000 | 2372 | tags=42%, list=33%, signal=63% |
| 133 | NEUROLOGICAL\_SYSTEM\_PROCESS |  | 80 | -0.25 | -0.98 | 0.493 | 0.899 | 1.000 | 557 | tags=13%, list=8%, signal=13% |
| 134 | NEGATIVE\_REGULATION\_OF\_RNA\_METABOLIC\_PROCESS |  | 41 | -0.29 | -0.98 | 0.502 | 0.895 | 1.000 | 1291 | tags=24%, list=18%, signal=30% |
| 135 | ATPASE\_ACTIVITY |  | 46 | -0.28 | -0.97 | 0.483 | 0.897 | 1.000 | 1296 | tags=24%, list=18%, signal=29% |
| 136 | NEGATIVE\_REGULATION\_OF\_TRANSCRIPTION |  | 60 | -0.27 | -0.97 | 0.501 | 0.894 | 1.000 | 1425 | tags=23%, list=20%, signal=29% |
| 137 | CATION\_BINDING |  | 69 | -0.26 | -0.97 | 0.521 | 0.893 | 1.000 | 537 | tags=12%, list=8%, signal=12% |
| 138 | NEGATIVE\_REGULATION\_OF\_METABOLIC\_PROCESS |  | 82 | -0.25 | -0.97 | 0.521 | 0.893 | 1.000 | 1860 | tags=27%, list=26%, signal=36% |
| 139 | SPLICEOSOME |  | 16 | -0.36 | -0.96 | 0.526 | 0.911 | 1.000 | 2840 | tags=56%, list=40%, signal=94% |
| 140 | NEGATIVE\_REGULATION\_OF\_PROGRAMMED\_CELL\_DEATH |  | 55 | -0.26 | -0.96 | 0.527 | 0.908 | 1.000 | 915 | tags=18%, list=13%, signal=21% |
| 141 | ENDOMEMBRANE\_SYSTEM |  | 95 | -0.24 | -0.96 | 0.552 | 0.908 | 1.000 | 1197 | tags=19%, list=17%, signal=22% |
| 142 | NEGATIVE\_REGULATION\_OF\_APOPTOSIS |  | 55 | -0.26 | -0.95 | 0.532 | 0.926 | 1.000 | 915 | tags=18%, list=13%, signal=21% |
| 143 | STRUCTURAL\_CONSTITUENT\_OF\_RIBOSOME |  | 16 | -0.35 | -0.95 | 0.538 | 0.927 | 1.000 | 1817 | tags=50%, list=26%, signal=67% |
| 144 | PROTEIN\_COMPLEX\_ASSEMBLY |  | 71 | -0.25 | -0.95 | 0.555 | 0.922 | 1.000 | 1593 | tags=24%, list=22%, signal=31% |
| 145 | RIBONUCLEOPROTEIN\_COMPLEX\_BIOGENESIS\_AND\_ASSEMBLY |  | 21 | -0.32 | -0.94 | 0.532 | 0.948 | 1.000 | 1072 | tags=19%, list=15%, signal=22% |
| 146 | INTRACELLULAR\_TRANSPORT |  | 116 | -0.23 | -0.93 | 0.614 | 0.961 | 1.000 | 1726 | tags=24%, list=24%, signal=31% |
| 147 | ION\_BINDING |  | 89 | -0.24 | -0.93 | 0.590 | 0.962 | 1.000 | 537 | tags=10%, list=8%, signal=11% |
| 148 | LIPID\_BINDING |  | 30 | -0.29 | -0.93 | 0.586 | 0.958 | 1.000 | 337 | tags=13%, list=5%, signal=14% |
| 149 | PYROPHOSPHATASE\_ACTIVITY |  | 92 | -0.23 | -0.93 | 0.608 | 0.953 | 1.000 | 967 | tags=14%, list=14%, signal=16% |
| 150 | HYDROLASE\_ACTIVITY\_ACTING\_ON\_ACID\_ANHYDRIDES |  | 92 | -0.23 | -0.92 | 0.624 | 0.954 | 1.000 | 967 | tags=14%, list=14%, signal=16% |
| 151 | GUANYL\_NUCLEOTIDE\_BINDING |  | 19 | -0.31 | -0.91 | 0.579 | 0.977 | 1.000 | 3178 | tags=47%, list=45%, signal=86% |
| 152 | PEPTIDASE\_ACTIVITY |  | 58 | -0.25 | -0.91 | 0.629 | 0.975 | 1.000 | 1358 | tags=24%, list=19%, signal=30% |
| 153 | ATPASE\_ACTIVITY\_COUPLED |  | 37 | -0.27 | -0.91 | 0.622 | 0.970 | 1.000 | 967 | tags=19%, list=14%, signal=22% |
| 154 | ESTABLISHMENT\_OF\_PROTEIN\_LOCALIZATION |  | 70 | -0.24 | -0.91 | 0.638 | 0.966 | 1.000 | 1726 | tags=24%, list=24%, signal=32% |
| 155 | PROTEIN\_LOCALIZATION |  | 76 | -0.24 | -0.91 | 0.623 | 0.965 | 1.000 | 2343 | tags=34%, list=33%, signal=51% |
| 156 | MEMBRANE\_LIPID\_METABOLIC\_PROCESS |  | 33 | -0.28 | -0.90 | 0.600 | 0.973 | 1.000 | 773 | tags=15%, list=11%, signal=17% |
| 157 | GTP\_BINDING |  | 19 | -0.31 | -0.90 | 0.608 | 0.974 | 1.000 | 3178 | tags=47%, list=45%, signal=86% |
| 158 | NUCLEOSIDE\_TRIPHOSPHATASE\_ACTIVITY |  | 88 | -0.23 | -0.90 | 0.666 | 0.973 | 1.000 | 967 | tags=14%, list=14%, signal=16% |
| 159 | PERINUCLEAR\_REGION\_OF\_CYTOPLASM |  | 21 | -0.30 | -0.89 | 0.634 | 0.977 | 1.000 | 1982 | tags=43%, list=28%, signal=59% |
| 160 | TRANSFERASE\_ACTIVITY\_TRANSFERRING\_GLYCOSYL\_GROUPS |  | 41 | -0.26 | -0.89 | 0.651 | 0.976 | 1.000 | 1082 | tags=20%, list=15%, signal=23% |
| 161 | ESTABLISHMENT\_OF\_CELLULAR\_LOCALIZATION |  | 142 | -0.21 | -0.89 | 0.724 | 0.980 | 1.000 | 1596 | tags=23%, list=23%, signal=28% |
| 162 | SMALL\_PROTEIN\_CONJUGATING\_ENZYME\_ACTIVITY |  | 25 | -0.29 | -0.88 | 0.657 | 1.000 | 1.000 | 2007 | tags=32%, list=28%, signal=44% |
| 163 | REGULATION\_OF\_TRANSCRIPTION\_FACTOR\_ACTIVITY |  | 15 | -0.32 | -0.88 | 0.651 | 1.000 | 1.000 | 692 | tags=20%, list=10%, signal=22% |
| 164 | ACTIVE\_TRANSMEMBRANE\_TRANSPORTER\_ACTIVITY |  | 39 | -0.26 | -0.88 | 0.642 | 0.997 | 1.000 | 1538 | tags=28%, list=22%, signal=36% |
| 165 | NUCLEAR\_ENVELOPE\_ENDOPLASMIC\_RETICULUM\_NETWORK |  | 39 | -0.26 | -0.88 | 0.653 | 0.992 | 1.000 | 2000 | tags=33%, list=28%, signal=46% |
| 166 | MACROMOLECULAR\_COMPLEX\_ASSEMBLY |  | 104 | -0.21 | -0.88 | 0.718 | 0.988 | 1.000 | 800 | tags=13%, list=11%, signal=15% |
| 167 | CELLULAR\_LOCALIZATION |  | 147 | -0.21 | -0.87 | 0.750 | 0.985 | 1.000 | 1596 | tags=22%, list=23%, signal=28% |
| 168 | DEPHOSPHORYLATION |  | 22 | -0.29 | -0.87 | 0.671 | 0.996 | 1.000 | 1458 | tags=32%, list=21%, signal=40% |
| 169 | NEGATIVE\_REGULATION\_OF\_CELLULAR\_METABOLIC\_PROCESS |  | 80 | -0.23 | -0.87 | 0.730 | 0.993 | 1.000 | 1860 | tags=26%, list=26%, signal=35% |
| 170 | CATION\_TRANSMEMBRANE\_TRANSPORTER\_ACTIVITY |  | 53 | -0.24 | -0.87 | 0.696 | 0.988 | 1.000 | 1593 | tags=30%, list=22%, signal=39% |
| 171 | HYDROLASE\_ACTIVITY\_ACTING\_ON\_ACID\_ANHYDRIDESCATALYZING\_TRANSMEMBRANE\_MOVEMENT\_OF\_SUBSTANCES |  | 15 | -0.32 | -0.86 | 0.651 | 0.986 | 1.000 | 1471 | tags=33%, list=21%, signal=42% |
| 172 | PRIMARY\_ACTIVE\_TRANSMEMBRANE\_TRANSPORTER\_ACTIVITY |  | 15 | -0.32 | -0.86 | 0.639 | 0.983 | 1.000 | 1471 | tags=33%, list=21%, signal=42% |
| 173 | CYSTEINE\_TYPE\_PEPTIDASE\_ACTIVITY |  | 16 | -0.32 | -0.86 | 0.647 | 0.982 | 1.000 | 1270 | tags=25%, list=18%, signal=30% |
| 174 | REGULATION\_OF\_DNA\_BINDING |  | 17 | -0.31 | -0.85 | 0.639 | 0.992 | 1.000 | 1783 | tags=35%, list=25%, signal=47% |
| 175 | TRANSFERASE\_ACTIVITY\_TRANSFERRING\_PHOSPHORUS\_CONTAINING\_GROUPS |  | 176 | -0.20 | -0.85 | 0.809 | 0.987 | 1.000 | 1284 | tags=16%, list=18%, signal=20% |
| 176 | MACROMOLECULE\_BIOSYNTHETIC\_PROCESS |  | 97 | -0.21 | -0.85 | 0.755 | 0.985 | 1.000 | 974 | tags=18%, list=14%, signal=20% |
| 177 | RESPONSE\_TO\_ABIOTIC\_STIMULUS |  | 26 | -0.27 | -0.85 | 0.704 | 0.983 | 1.000 | 1861 | tags=38%, list=26%, signal=52% |
| 178 | NUCLEAR\_IMPORT |  | 19 | -0.29 | -0.85 | 0.693 | 0.989 | 1.000 | 1726 | tags=32%, list=24%, signal=42% |
| 179 | POSITIVE\_REGULATION\_OF\_HYDROLASE\_ACTIVITY |  | 20 | -0.29 | -0.83 | 0.713 | 1.000 | 1.000 | 327 | tags=15%, list=5%, signal=16% |
| 180 | NUCLEOTIDE\_METABOLIC\_PROCESS |  | 18 | -0.29 | -0.83 | 0.671 | 1.000 | 1.000 | 1742 | tags=39%, list=25%, signal=51% |
| 181 | ENDOPLASMIC\_RETICULUM\_MEMBRANE |  | 36 | -0.25 | -0.83 | 0.761 | 1.000 | 1.000 | 2000 | tags=33%, list=28%, signal=46% |
| 182 | RNA\_METABOLIC\_PROCESS |  | 290 | -0.18 | -0.83 | 0.904 | 1.000 | 1.000 | 2429 | tags=35%, list=34%, signal=51% |
| 183 | LIPID\_CATABOLIC\_PROCESS |  | 15 | -0.30 | -0.82 | 0.718 | 1.000 | 1.000 | 1042 | tags=27%, list=15%, signal=31% |
| 184 | DNA\_PACKAGING |  | 18 | -0.29 | -0.82 | 0.723 | 1.000 | 1.000 | 1149 | tags=22%, list=16%, signal=26% |
| 185 | ELECTRON\_TRANSPORT\_GO\_0006118 |  | 22 | -0.28 | -0.82 | 0.732 | 1.000 | 1.000 | 1934 | tags=41%, list=27%, signal=56% |
| 186 | SMALL\_CONJUGATING\_PROTEIN\_LIGASE\_ACTIVITY |  | 25 | -0.26 | -0.81 | 0.735 | 1.000 | 1.000 | 2007 | tags=32%, list=28%, signal=44% |
| 187 | TRANSMEMBRANE\_RECEPTOR\_PROTEIN\_TYROSINE\_KINASE\_SIGNALING\_PATHWAY |  | 26 | -0.26 | -0.80 | 0.764 | 1.000 | 1.000 | 1784 | tags=38%, list=25%, signal=51% |
| 188 | ENZYME\_BINDING |  | 65 | -0.22 | -0.80 | 0.807 | 1.000 | 1.000 | 1689 | tags=31%, list=24%, signal=40% |
| 189 | ATPASE\_ACTIVITY\_COUPLED\_TO\_MOVEMENT\_OF\_SUBSTANCES |  | 16 | -0.29 | -0.80 | 0.740 | 1.000 | 1.000 | 1471 | tags=31%, list=21%, signal=39% |
| 190 | REGULATION\_OF\_PHOSPHORYLATION |  | 21 | -0.26 | -0.78 | 0.766 | 1.000 | 1.000 | 1697 | tags=33%, list=24%, signal=44% |
| 191 | APOPTOSIS\_GO |  | 157 | -0.18 | -0.78 | 0.930 | 1.000 | 1.000 | 1264 | tags=17%, list=18%, signal=20% |
| 192 | MACROMOLECULE\_LOCALIZATION |  | 84 | -0.20 | -0.78 | 0.882 | 1.000 | 1.000 | 2343 | tags=32%, list=33%, signal=47% |
| 193 | PROGRAMMED\_CELL\_DEATH |  | 157 | -0.18 | -0.77 | 0.934 | 1.000 | 1.000 | 1264 | tags=17%, list=18%, signal=20% |
| 194 | REGULATION\_OF\_APOPTOSIS |  | 125 | -0.18 | -0.77 | 0.908 | 1.000 | 1.000 | 1214 | tags=17%, list=17%, signal=20% |
| 195 | TRANSCRIPTION\_COACTIVATOR\_ACTIVITY |  | 34 | -0.24 | -0.77 | 0.826 | 1.000 | 1.000 | 1489 | tags=24%, list=21%, signal=30% |
| 196 | REGULATION\_OF\_PROTEIN\_MODIFICATION\_PROCESS |  | 17 | -0.27 | -0.77 | 0.796 | 1.000 | 1.000 | 187 | tags=12%, list=3%, signal=12% |
| 197 | TRANSPORT |  | 274 | -0.17 | -0.76 | 0.965 | 1.000 | 1.000 | 1610 | tags=22%, list=23%, signal=27% |
| 198 | RNA\_POLYMERASE\_II\_TRANSCRIPTION\_FACTOR\_ACTIVITY |  | 54 | -0.21 | -0.76 | 0.861 | 1.000 | 1.000 | 1552 | tags=20%, list=22%, signal=26% |
| 199 | REGULATION\_OF\_PROGRAMMED\_CELL\_DEATH |  | 125 | -0.18 | -0.76 | 0.929 | 1.000 | 1.000 | 1214 | tags=17%, list=17%, signal=20% |
| 200 | KINASE\_BINDING |  | 24 | -0.25 | -0.75 | 0.813 | 1.000 | 1.000 | 2062 | tags=46%, list=29%, signal=64% |
| 201 | TRANSCRIPTION\_ACTIVATOR\_ACTIVITY |  | 51 | -0.21 | -0.75 | 0.876 | 1.000 | 1.000 | 1959 | tags=29%, list=28%, signal=40% |
| 202 | MEMBRANE\_ORGANIZATION\_AND\_BIOGENESIS |  | 50 | -0.21 | -0.75 | 0.882 | 1.000 | 1.000 | 1610 | tags=28%, list=23%, signal=36% |
| 203 | MICROTUBULE\_BASED\_PROCESS |  | 21 | -0.26 | -0.74 | 0.830 | 1.000 | 1.000 | 1509 | tags=29%, list=21%, signal=36% |
| 204 | GOLGI\_VESICLE\_TRANSPORT |  | 22 | -0.25 | -0.74 | 0.836 | 1.000 | 1.000 | 2444 | tags=36%, list=34%, signal=55% |
| 205 | PROTEOLYSIS |  | 78 | -0.19 | -0.74 | 0.933 | 1.000 | 1.000 | 2250 | tags=36%, list=32%, signal=52% |
| 206 | ESTABLISHMENT\_OF\_LOCALIZATION |  | 294 | -0.16 | -0.73 | 0.986 | 1.000 | 1.000 | 1610 | tags=21%, list=23%, signal=26% |
| 207 | PROTEIN\_OLIGOMERIZATION |  | 15 | -0.28 | -0.73 | 0.812 | 1.000 | 1.000 | 1742 | tags=33%, list=25%, signal=44% |
| 208 | RNA\_BIOSYNTHETIC\_PROCESS |  | 217 | -0.17 | -0.73 | 0.976 | 1.000 | 1.000 | 2429 | tags=35%, list=34%, signal=51% |
| 209 | PROTEIN\_MODIFICATION\_BY\_SMALL\_PROTEIN\_CONJUGATION |  | 20 | -0.25 | -0.73 | 0.858 | 1.000 | 1.000 | 3420 | tags=65%, list=48%, signal=125% |
| 210 | INTERCELLULAR\_JUNCTION |  | 20 | -0.25 | -0.73 | 0.826 | 1.000 | 1.000 | 1956 | tags=30%, list=28%, signal=41% |
| 211 | PROTEIN\_KINASE\_BINDING |  | 19 | -0.25 | -0.72 | 0.819 | 1.000 | 1.000 | 2062 | tags=47%, list=29%, signal=67% |
| 212 | UBIQUITIN\_PROTEIN\_LIGASE\_ACTIVITY |  | 23 | -0.25 | -0.72 | 0.867 | 1.000 | 1.000 | 2007 | tags=30%, list=28%, signal=42% |
| 213 | TRANSCRIPTION\_DNA\_DEPENDENT |  | 216 | -0.16 | -0.72 | 0.994 | 1.000 | 1.000 | 2429 | tags=34%, list=34%, signal=51% |
| 214 | ENDOPLASMIC\_RETICULUM\_PART |  | 40 | -0.21 | -0.72 | 0.901 | 1.000 | 1.000 | 2000 | tags=30%, list=28%, signal=42% |
| 215 | TRANSCRIPTION |  | 252 | -0.16 | -0.72 | 0.990 | 1.000 | 1.000 | 1861 | tags=24%, list=26%, signal=31% |
| 216 | SECRETORY\_PATHWAY |  | 34 | -0.22 | -0.71 | 0.877 | 1.000 | 1.000 | 1565 | tags=24%, list=22%, signal=30% |
| 217 | PEPTIDE\_BINDING |  | 24 | -0.23 | -0.71 | 0.896 | 1.000 | 1.000 | 1712 | tags=33%, list=24%, signal=44% |
| 218 | GOLGI\_APPARATUS\_PART |  | 39 | -0.21 | -0.70 | 0.916 | 1.000 | 1.000 | 2754 | tags=38%, list=39%, signal=63% |
| 219 | NUCLEAR\_MEMBRANE\_PART |  | 20 | -0.24 | -0.70 | 0.887 | 1.000 | 1.000 | 1533 | tags=25%, list=22%, signal=32% |
| 220 | SECRETION\_BY\_CELL |  | 41 | -0.21 | -0.70 | 0.908 | 1.000 | 1.000 | 1565 | tags=22%, list=22%, signal=28% |
| 221 | NUCLEAR\_MEMBRANE |  | 24 | -0.23 | -0.69 | 0.888 | 1.000 | 1.000 | 1533 | tags=25%, list=22%, signal=32% |
| 222 | PROTEIN\_UBIQUITINATION |  | 19 | -0.23 | -0.68 | 0.890 | 1.000 | 1.000 | 3420 | tags=63%, list=48%, signal=122% |
| 223 | REGULATION\_OF\_CYCLIN\_DEPENDENT\_PROTEIN\_KINASE\_ACTIVITY |  | 18 | -0.24 | -0.68 | 0.884 | 1.000 | 1.000 | 1436 | tags=22%, list=20%, signal=28% |
| 224 | CELL\_CYCLE\_GO\_0007049 |  | 114 | -0.16 | -0.67 | 0.993 | 1.000 | 1.000 | 2610 | tags=38%, list=37%, signal=59% |
| 225 | REGULATION\_OF\_CELLULAR\_COMPONENT\_ORGANIZATION\_AND\_BIOGENESIS |  | 43 | -0.19 | -0.66 | 0.936 | 1.000 | 1.000 | 1746 | tags=26%, list=25%, signal=34% |
| 226 | PROTEIN\_IMPORT\_INTO\_NUCLEUS |  | 18 | -0.24 | -0.66 | 0.913 | 1.000 | 1.000 | 2834 | tags=50%, list=40%, signal=83% |
| 227 | REGULATION\_OF\_HYDROLASE\_ACTIVITY |  | 30 | -0.21 | -0.66 | 0.925 | 1.000 | 1.000 | 611 | tags=13%, list=9%, signal=15% |
| 228 | MICROTUBULE\_ORGANIZING\_CENTER |  | 19 | -0.23 | -0.65 | 0.902 | 1.000 | 1.000 | 2760 | tags=42%, list=39%, signal=69% |
| 229 | SECRETION |  | 56 | -0.17 | -0.62 | 0.988 | 1.000 | 1.000 | 1565 | tags=20%, list=22%, signal=25% |
| 230 | NEGATIVE\_REGULATION\_OF\_CELL\_CYCLE |  | 36 | -0.18 | -0.62 | 0.962 | 1.000 | 1.000 | 2336 | tags=36%, list=33%, signal=54% |
| 231 | CELL\_CYCLE\_ARREST\_GO\_0007050 |  | 32 | -0.19 | -0.61 | 0.969 | 1.000 | 1.000 | 2336 | tags=34%, list=33%, signal=51% |
| 232 | LYSOSOME |  | 21 | -0.21 | -0.61 | 0.954 | 1.000 | 1.000 | 2760 | tags=52%, list=39%, signal=85% |
| 233 | LYTIC\_VACUOLE |  | 21 | -0.21 | -0.61 | 0.957 | 1.000 | 1.000 | 2760 | tags=52%, list=39%, signal=85% |
| 234 | VESICLE\_MEDIATED\_TRANSPORT |  | 78 | -0.15 | -0.59 | 0.997 | 1.000 | 1.000 | 1610 | tags=21%, list=23%, signal=26% |
| 235 | REGULATION\_OF\_CELL\_CYCLE |  | 78 | -0.15 | -0.59 | 0.993 | 1.000 | 1.000 | 2566 | tags=36%, list=36%, signal=56% |
| 236 | TRANSMEMBRANE\_RECEPTOR\_PROTEIN\_SERINE\_THREONINE\_KINASE\_SIGNALING\_PATHWAY |  | 17 | -0.21 | -0.57 | 0.953 | 1.000 | 1.000 | 100 | tags=6%, list=1%, signal=6% |
| 237 | GLYCOPROTEIN\_BIOSYNTHETIC\_PROCESS |  | 22 | -0.19 | -0.55 | 0.987 | 1.000 | 1.000 | 1702 | tags=23%, list=24%, signal=30% |
| 238 | ENDOSOME |  | 29 | -0.17 | -0.55 | 0.992 | 0.998 | 1.000 | 1908 | tags=24%, list=27%, signal=33% |
| 239 | UBIQUITIN\_CYCLE |  | 23 | -0.18 | -0.55 | 0.979 | 0.994 | 1.000 | 3420 | tags=65%, list=48%, signal=126% |
| 240 | VACUOLE |  | 25 | -0.15 | -0.44 | 0.997 | 0.999 | 1.000 | 2760 | tags=48%, list=39%, signal=78% |
Table: Gene sets enriched in phenotype **na**[plain text format]****

  
