# Supplementary material for: Network-based integration of molecular and physiological data elucidates regulatory mechanisms underlying adaptation to high-fat diet
Source: Genes Nutr. 2015 May 28;10(4):22. doi: 10.1007/s12263-015-0470-6 (PMC4446272; doi:10.1007/s12263-015-0470-6)
Supplement: Supplementary file 4 — Supplementary material 4 (ZIP 6984 kb) [file 12263_2015_470_MOESM4_ESM.zip › HF LF 12 w GSEA result/gsea_report_for_na_pos_1365169146545.html]

Report for na\_pos 1365169146545 [GSEA]

| GS  follow link to MSigDB | GS DETAILS | SIZE | ES | NES | NOM p-val | FDR q-val | FWER p-val | RANK AT MAX | LEADING EDGE || 1 | EXTRACELLULAR\_REGION\_PART | Details ... | 116 | 0.57 | 2.59 | 0.000 | 0.000 | 0.000 | 761 | tags=35%, list=11%, signal=39% |
| 2 | EXTRACELLULAR\_REGION | Details ... | 145 | 0.55 | 2.57 | 0.000 | 0.000 | 0.000 | 761 | tags=33%, list=11%, signal=36% |
| 3 | IMMUNE\_SYSTEM\_PROCESS | Details ... | 127 | 0.53 | 2.50 | 0.000 | 0.000 | 0.000 | 883 | tags=35%, list=12%, signal=40% |
| 4 | EXTRACELLULAR\_SPACE | Details ... | 83 | 0.58 | 2.46 | 0.000 | 0.000 | 0.000 | 761 | tags=35%, list=11%, signal=39% |
| 5 | BEHAVIOR | Details ... | 45 | 0.62 | 2.33 | 0.000 | 0.000 | 0.001 | 761 | tags=44%, list=11%, signal=49% |
| 6 | RESPONSE\_TO\_WOUNDING | Details ... | 65 | 0.56 | 2.32 | 0.000 | 0.000 | 0.001 | 1395 | tags=49%, list=20%, signal=61% |
| 7 | RESPONSE\_TO\_EXTERNAL\_STIMULUS | Details ... | 101 | 0.51 | 2.29 | 0.000 | 0.000 | 0.002 | 898 | tags=39%, list=13%, signal=44% |
| 8 | LOCOMOTORY\_BEHAVIOR | Details ... | 36 | 0.61 | 2.25 | 0.000 | 0.001 | 0.003 | 761 | tags=47%, list=11%, signal=53% |
| 9 | HEMOPOIESIS | Details ... | 35 | 0.61 | 2.20 | 0.000 | 0.001 | 0.003 | 686 | tags=31%, list=10%, signal=35% |
| 10 | HEMOPOIETIC\_OR\_LYMPHOID\_ORGAN\_DEVELOPMENT | Details ... | 36 | 0.61 | 2.17 | 0.000 | 0.001 | 0.006 | 686 | tags=31%, list=10%, signal=34% |
| 11 | EXTRACELLULAR\_MATRIX | Details ... | 34 | 0.59 | 2.17 | 0.000 | 0.001 | 0.006 | 1664 | tags=59%, list=23%, signal=76% |
| 12 | PROTEINACEOUS\_EXTRACELLULAR\_MATRIX | Details ... | 34 | 0.59 | 2.15 | 0.000 | 0.001 | 0.009 | 1664 | tags=59%, list=23%, signal=76% |
| 13 | RECEPTOR\_ACTIVITY | Details ... | 141 | 0.44 | 2.14 | 0.000 | 0.001 | 0.011 | 1649 | tags=47%, list=23%, signal=60% |
| 14 | IMMUNE\_SYSTEM\_DEVELOPMENT | Details ... | 37 | 0.60 | 2.14 | 0.000 | 0.001 | 0.011 | 686 | tags=30%, list=10%, signal=33% |
| 15 | DEFENSE\_RESPONSE | Details ... | 73 | 0.51 | 2.14 | 0.000 | 0.001 | 0.012 | 939 | tags=36%, list=13%, signal=41% |
| 16 | INFLAMMATORY\_RESPONSE | Details ... | 39 | 0.58 | 2.13 | 0.000 | 0.001 | 0.012 | 752 | tags=41%, list=11%, signal=46% |
| 17 | CATION\_HOMEOSTASIS | Details ... | 35 | 0.57 | 2.08 | 0.000 | 0.003 | 0.027 | 1514 | tags=54%, list=21%, signal=69% |
| 18 | CELLULAR\_CATION\_HOMEOSTASIS | Details ... | 35 | 0.57 | 2.07 | 0.000 | 0.003 | 0.029 | 1514 | tags=54%, list=21%, signal=69% |
| 19 | T\_CELL\_ACTIVATION | Details ... | 15 | 0.73 | 2.07 | 0.000 | 0.003 | 0.031 | 1335 | tags=67%, list=19%, signal=82% |
| 20 | CELL\_ACTIVATION | Details ... | 24 | 0.62 | 2.06 | 0.000 | 0.002 | 0.031 | 1596 | tags=54%, list=23%, signal=70% |
| 21 | MYELOID\_CELL\_DIFFERENTIATION |  | 21 | 0.63 | 2.04 | 0.000 | 0.003 | 0.037 | 623 | tags=33%, list=9%, signal=36% |
| 22 | POSITIVE\_REGULATION\_OF\_MULTICELLULAR\_ORGANISMAL\_PROCESS |  | 19 | 0.65 | 2.01 | 0.000 | 0.004 | 0.059 | 1116 | tags=42%, list=16%, signal=50% |
| 23 | IMMUNE\_RESPONSE |  | 87 | 0.47 | 2.00 | 0.000 | 0.005 | 0.063 | 943 | tags=34%, list=13%, signal=39% |
| 24 | LYMPHOCYTE\_ACTIVATION |  | 21 | 0.63 | 2.00 | 0.000 | 0.004 | 0.065 | 1798 | tags=57%, list=25%, signal=76% |
| 25 | CELL\_SURFACE\_RECEPTOR\_LINKED\_SIGNAL\_TRANSDUCTION\_GO\_0007166 |  | 179 | 0.41 | 2.00 | 0.000 | 0.004 | 0.068 | 1454 | tags=36%, list=21%, signal=44% |
| 26 | COAGULATION |  | 19 | 0.63 | 1.98 | 0.000 | 0.005 | 0.084 | 898 | tags=42%, list=13%, signal=48% |
| 27 | ORGAN\_DEVELOPMENT |  | 169 | 0.41 | 1.97 | 0.000 | 0.005 | 0.089 | 864 | tags=24%, list=12%, signal=27% |
| 28 | LEUKOCYTE\_ACTIVATION |  | 22 | 0.60 | 1.95 | 0.000 | 0.006 | 0.106 | 1798 | tags=55%, list=25%, signal=73% |
| 29 | EXTRACELLULAR\_MATRIX\_PART |  | 22 | 0.61 | 1.95 | 0.000 | 0.006 | 0.108 | 753 | tags=41%, list=11%, signal=46% |
| 30 | HEMOSTASIS |  | 20 | 0.62 | 1.94 | 0.000 | 0.007 | 0.128 | 898 | tags=40%, list=13%, signal=46% |
| 31 | G\_PROTEIN\_COUPLED\_RECEPTOR\_PROTEIN\_SIGNALING\_PATHWAY |  | 82 | 0.44 | 1.93 | 0.000 | 0.008 | 0.144 | 1777 | tags=48%, list=25%, signal=63% |
| 32 | BLOOD\_COAGULATION |  | 19 | 0.63 | 1.91 | 0.003 | 0.009 | 0.170 | 898 | tags=42%, list=13%, signal=48% |
| 33 | WOUND\_HEALING |  | 23 | 0.58 | 1.91 | 0.000 | 0.009 | 0.172 | 1356 | tags=43%, list=19%, signal=54% |
| 34 | CELLULAR\_DEFENSE\_RESPONSE |  | 19 | 0.62 | 1.91 | 0.000 | 0.009 | 0.177 | 939 | tags=42%, list=13%, signal=48% |
| 35 | REGULATION\_OF\_BODY\_FLUID\_LEVELS |  | 26 | 0.57 | 1.91 | 0.000 | 0.009 | 0.179 | 1356 | tags=46%, list=19%, signal=57% |
| 36 | PLASMA\_MEMBRANE\_PART |  | 322 | 0.36 | 1.90 | 0.000 | 0.009 | 0.188 | 1337 | tags=32%, list=19%, signal=38% |
| 37 | G\_PROTEIN\_COUPLED\_RECEPTOR\_BINDING |  | 20 | 0.61 | 1.89 | 0.000 | 0.010 | 0.205 | 752 | tags=50%, list=11%, signal=56% |
| 38 | REGULATION\_OF\_IMMUNE\_SYSTEM\_PROCESS |  | 19 | 0.61 | 1.89 | 0.005 | 0.010 | 0.210 | 849 | tags=32%, list=12%, signal=36% |
| 39 | CHEMOKINE\_RECEPTOR\_BINDING |  | 17 | 0.63 | 1.89 | 0.002 | 0.009 | 0.213 | 752 | tags=53%, list=11%, signal=59% |
| 40 | RAS\_PROTEIN\_SIGNAL\_TRANSDUCTION |  | 34 | 0.52 | 1.88 | 0.000 | 0.010 | 0.220 | 798 | tags=29%, list=11%, signal=33% |
| 41 | SECOND\_MESSENGER\_MEDIATED\_SIGNALING |  | 40 | 0.51 | 1.88 | 0.000 | 0.009 | 0.222 | 1306 | tags=43%, list=18%, signal=52% |
| 42 | TRANSMEMBRANE\_RECEPTOR\_ACTIVITY |  | 86 | 0.43 | 1.88 | 0.000 | 0.010 | 0.238 | 1649 | tags=48%, list=23%, signal=61% |
| 43 | TRANSCRIPTION\_FACTOR\_ACTIVITY |  | 90 | 0.42 | 1.87 | 0.000 | 0.010 | 0.251 | 1377 | tags=39%, list=19%, signal=48% |
| 44 | INTEGRAL\_TO\_PLASMA\_MEMBRANE |  | 258 | 0.36 | 1.87 | 0.000 | 0.010 | 0.260 | 943 | tags=27%, list=13%, signal=30% |
| 45 | G\_PROTEIN\_SIGNALING\_COUPLED\_TO\_CYCLIC\_NUCLEOTIDE\_SECOND\_MESSENGER |  | 23 | 0.56 | 1.87 | 0.005 | 0.010 | 0.261 | 219 | tags=26%, list=3%, signal=27% |
| 46 | ORGAN\_MORPHOGENESIS |  | 50 | 0.47 | 1.86 | 0.000 | 0.010 | 0.261 | 722 | tags=26%, list=10%, signal=29% |
| 47 | MULTICELLULAR\_ORGANISMAL\_DEVELOPMENT |  | 287 | 0.36 | 1.86 | 0.000 | 0.010 | 0.266 | 917 | tags=24%, list=13%, signal=27% |
| 48 | PLASMA\_MEMBRANE |  | 407 | 0.35 | 1.86 | 0.000 | 0.010 | 0.276 | 1337 | tags=32%, list=19%, signal=37% |
| 49 | LEUKOCYTE\_DIFFERENTIATION |  | 16 | 0.64 | 1.85 | 0.008 | 0.010 | 0.283 | 686 | tags=31%, list=10%, signal=35% |
| 50 | INTRINSIC\_TO\_PLASMA\_MEMBRANE |  | 266 | 0.35 | 1.84 | 0.000 | 0.012 | 0.318 | 1337 | tags=33%, list=19%, signal=39% |
| 51 | CELL\_PROLIFERATION\_GO\_0008283 |  | 168 | 0.38 | 1.83 | 0.000 | 0.012 | 0.332 | 1441 | tags=32%, list=20%, signal=39% |
| 52 | RECEPTOR\_BINDING |  | 116 | 0.40 | 1.82 | 0.000 | 0.013 | 0.344 | 1397 | tags=36%, list=20%, signal=44% |
| 53 | PROTEIN\_KINASE\_CASCADE |  | 108 | 0.40 | 1.82 | 0.000 | 0.014 | 0.371 | 1580 | tags=36%, list=22%, signal=46% |
| 54 | REGULATION\_OF\_MAP\_KINASE\_ACTIVITY |  | 28 | 0.53 | 1.80 | 0.000 | 0.016 | 0.415 | 430 | tags=25%, list=6%, signal=27% |
| 55 | CYTOKINE\_ACTIVITY |  | 29 | 0.52 | 1.77 | 0.000 | 0.019 | 0.487 | 752 | tags=38%, list=11%, signal=42% |
| 56 | CYTOKINE\_PRODUCTION |  | 23 | 0.55 | 1.76 | 0.005 | 0.021 | 0.535 | 1006 | tags=43%, list=14%, signal=51% |
| 57 | SYSTEM\_DEVELOPMENT |  | 237 | 0.35 | 1.75 | 0.000 | 0.023 | 0.563 | 864 | tags=22%, list=12%, signal=24% |
| 58 | RECEPTOR\_COMPLEX |  | 15 | 0.60 | 1.74 | 0.002 | 0.025 | 0.596 | 570 | tags=40%, list=8%, signal=43% |
| 59 | POSITIVE\_REGULATION\_OF\_CELL\_PROLIFERATION |  | 50 | 0.43 | 1.74 | 0.000 | 0.024 | 0.597 | 1377 | tags=36%, list=19%, signal=44% |
| 60 | CHEMOKINE\_ACTIVITY |  | 16 | 0.60 | 1.74 | 0.010 | 0.024 | 0.605 | 752 | tags=50%, list=11%, signal=56% |
| 61 | RECEPTOR\_SIGNALING\_PROTEIN\_ACTIVITY |  | 33 | 0.49 | 1.73 | 0.006 | 0.025 | 0.621 | 1360 | tags=36%, list=19%, signal=45% |
| 62 | ION\_HOMEOSTASIS |  | 42 | 0.45 | 1.72 | 0.008 | 0.027 | 0.652 | 1639 | tags=52%, list=23%, signal=68% |
| 63 | ANATOMICAL\_STRUCTURE\_DEVELOPMENT |  | 280 | 0.33 | 1.71 | 0.000 | 0.030 | 0.707 | 1596 | tags=32%, list=23%, signal=40% |
| 64 | SMALL\_GTPASE\_REGULATOR\_ACTIVITY |  | 23 | 0.53 | 1.71 | 0.019 | 0.030 | 0.712 | 1355 | tags=43%, list=19%, signal=54% |
| 65 | G\_PROTEIN\_SIGNALING\_COUPLED\_TO\_CAMP\_NUCLEOTIDE\_SECOND\_MESSENGER |  | 15 | 0.59 | 1.70 | 0.017 | 0.033 | 0.739 | 219 | tags=33%, list=3%, signal=34% |
| 66 | INTEGRAL\_TO\_MEMBRANE |  | 378 | 0.32 | 1.69 | 0.000 | 0.033 | 0.745 | 943 | tags=24%, list=13%, signal=26% |
| 67 | MULTI\_ORGANISM\_PROCESS |  | 45 | 0.43 | 1.69 | 0.015 | 0.032 | 0.745 | 830 | tags=36%, list=12%, signal=40% |
| 68 | REPRODUCTIVE\_PROCESS |  | 42 | 0.45 | 1.69 | 0.000 | 0.032 | 0.746 | 917 | tags=38%, list=13%, signal=43% |
| 69 | INTRACELLULAR\_SIGNALING\_CASCADE |  | 240 | 0.33 | 1.69 | 0.000 | 0.033 | 0.761 | 1446 | tags=31%, list=20%, signal=37% |
| 70 | REGULATION\_OF\_CELL\_PROLIFERATION |  | 99 | 0.37 | 1.68 | 0.000 | 0.034 | 0.772 | 1441 | tags=30%, list=20%, signal=37% |
| 71 | REGULATION\_OF\_BIOLOGICAL\_QUALITY |  | 152 | 0.36 | 1.68 | 0.000 | 0.034 | 0.776 | 1514 | tags=36%, list=21%, signal=45% |
| 72 | RESPONSE\_TO\_CHEMICAL\_STIMULUS |  | 105 | 0.37 | 1.68 | 0.000 | 0.033 | 0.779 | 930 | tags=30%, list=13%, signal=35% |
| 73 | INTRINSIC\_TO\_MEMBRANE |  | 388 | 0.31 | 1.68 | 0.000 | 0.033 | 0.785 | 943 | tags=23%, list=13%, signal=25% |
| 74 | ANATOMICAL\_STRUCTURE\_MORPHOGENESIS |  | 119 | 0.36 | 1.67 | 0.000 | 0.034 | 0.793 | 1750 | tags=37%, list=25%, signal=48% |
| 75 | ACTIVATION\_OF\_MAPK\_ACTIVITY |  | 16 | 0.58 | 1.67 | 0.017 | 0.033 | 0.793 | 830 | tags=38%, list=12%, signal=42% |
| 76 | REGULATION\_OF\_CELL\_DIFFERENTIATION |  | 24 | 0.51 | 1.67 | 0.008 | 0.033 | 0.794 | 684 | tags=29%, list=10%, signal=32% |
| 77 | POSITIVE\_REGULATION\_OF\_MAP\_KINASE\_ACTIVITY |  | 22 | 0.52 | 1.65 | 0.017 | 0.038 | 0.838 | 1261 | tags=41%, list=18%, signal=50% |
| 78 | CYCLIC\_NUCLEOTIDE\_MEDIATED\_SIGNALING |  | 24 | 0.50 | 1.65 | 0.022 | 0.039 | 0.852 | 219 | tags=25%, list=3%, signal=26% |
| 79 | TRANSMEMBRANE\_RECEPTOR\_PROTEIN\_KINASE\_ACTIVITY |  | 19 | 0.53 | 1.65 | 0.016 | 0.039 | 0.857 | 1225 | tags=42%, list=17%, signal=51% |
| 80 | GTPASE\_REGULATOR\_ACTIVITY |  | 46 | 0.43 | 1.64 | 0.006 | 0.041 | 0.873 | 754 | tags=28%, list=11%, signal=31% |
| 81 | CATION\_CHANNEL\_ACTIVITY |  | 22 | 0.51 | 1.64 | 0.008 | 0.042 | 0.888 | 1122 | tags=45%, list=16%, signal=54% |
| 82 | GATED\_CHANNEL\_ACTIVITY |  | 21 | 0.52 | 1.63 | 0.016 | 0.041 | 0.888 | 1122 | tags=48%, list=16%, signal=56% |
| 83 | ENZYME\_INHIBITOR\_ACTIVITY |  | 38 | 0.44 | 1.63 | 0.011 | 0.043 | 0.901 | 557 | tags=29%, list=8%, signal=31% |
| 84 | RECEPTOR\_SIGNALING\_PROTEIN\_SERINE\_THREONINE\_KINASE\_ACTIVITY |  | 16 | 0.55 | 1.63 | 0.017 | 0.043 | 0.901 | 109 | tags=13%, list=2%, signal=13% |
| 85 | STRESS\_ACTIVATED\_PROTEIN\_KINASE\_SIGNALING\_PATHWAY |  | 15 | 0.55 | 1.62 | 0.025 | 0.045 | 0.915 | 1261 | tags=33%, list=18%, signal=40% |
| 86 | REGULATION\_OF\_MULTICELLULAR\_ORGANISMAL\_PROCESS |  | 41 | 0.42 | 1.61 | 0.011 | 0.046 | 0.926 | 1116 | tags=34%, list=16%, signal=40% |
| 87 | CELL\_MIGRATION |  | 28 | 0.47 | 1.61 | 0.020 | 0.047 | 0.935 | 1459 | tags=43%, list=21%, signal=54% |
| 88 | ENZYME\_REGULATOR\_ACTIVITY |  | 122 | 0.34 | 1.60 | 0.000 | 0.049 | 0.941 | 557 | tags=20%, list=8%, signal=21% |
| 89 | JNK\_CASCADE |  | 15 | 0.55 | 1.59 | 0.021 | 0.051 | 0.948 | 1261 | tags=33%, list=18%, signal=40% |
| 90 | CELL\_CELL\_SIGNALING |  | 95 | 0.35 | 1.59 | 0.000 | 0.051 | 0.953 | 1253 | tags=31%, list=18%, signal=37% |
| 91 | MAPKKK\_CASCADE\_GO\_0000165 |  | 35 | 0.44 | 1.59 | 0.018 | 0.053 | 0.958 | 1006 | tags=26%, list=14%, signal=30% |
| 92 | REPRODUCTION |  | 69 | 0.37 | 1.56 | 0.013 | 0.062 | 0.974 | 1083 | tags=30%, list=15%, signal=36% |
| 93 | CELLULAR\_HOMEOSTASIS |  | 46 | 0.41 | 1.56 | 0.013 | 0.062 | 0.975 | 1639 | tags=50%, list=23%, signal=65% |
| 94 | CELL\_PROJECTION |  | 38 | 0.42 | 1.54 | 0.019 | 0.071 | 0.991 | 2226 | tags=53%, list=31%, signal=76% |
| 95 | SMALL\_GTPASE\_MEDIATED\_SIGNAL\_TRANSDUCTION |  | 42 | 0.41 | 1.54 | 0.017 | 0.072 | 0.991 | 798 | tags=26%, list=11%, signal=29% |
| 96 | GENERATION\_OF\_NEURONS |  | 20 | 0.49 | 1.53 | 0.032 | 0.074 | 0.992 | 1316 | tags=40%, list=19%, signal=49% |
| 97 | CAMP\_MEDIATED\_SIGNALING |  | 16 | 0.52 | 1.51 | 0.054 | 0.085 | 0.998 | 219 | tags=31%, list=3%, signal=32% |
| 98 | MOLECULAR\_ADAPTOR\_ACTIVITY |  | 18 | 0.49 | 1.51 | 0.056 | 0.085 | 0.998 | 1360 | tags=44%, list=19%, signal=55% |
| 99 | POSITIVE\_REGULATION\_OF\_BIOLOGICAL\_PROCESS |  | 241 | 0.29 | 1.51 | 0.000 | 0.085 | 0.998 | 1583 | tags=32%, list=22%, signal=40% |
| 100 | METAL\_ION\_TRANSMEMBRANE\_TRANSPORTER\_ACTIVITY |  | 30 | 0.43 | 1.49 | 0.038 | 0.091 | 0.998 | 1122 | tags=37%, list=16%, signal=43% |
| 101 | ION\_CHANNEL\_ACTIVITY |  | 24 | 0.46 | 1.49 | 0.062 | 0.094 | 0.999 | 1122 | tags=42%, list=16%, signal=49% |
| 102 | ANGIOGENESIS |  | 23 | 0.46 | 1.48 | 0.028 | 0.098 | 1.000 | 623 | tags=26%, list=9%, signal=29% |
| 103 | SH3\_SH2\_ADAPTOR\_ACTIVITY |  | 17 | 0.50 | 1.48 | 0.084 | 0.098 | 1.000 | 1360 | tags=47%, list=19%, signal=58% |
| 104 | ION\_TRANSPORT |  | 46 | 0.39 | 1.47 | 0.034 | 0.101 | 1.000 | 1398 | tags=39%, list=20%, signal=48% |
| 105 | REGULATION\_OF\_SIGNAL\_TRANSDUCTION |  | 82 | 0.33 | 1.45 | 0.010 | 0.112 | 1.000 | 1580 | tags=33%, list=22%, signal=42% |
| 106 | PROTEIN\_AMINO\_ACID\_PHOSPHORYLATION |  | 100 | 0.33 | 1.45 | 0.013 | 0.111 | 1.000 | 1599 | tags=30%, list=23%, signal=38% |
| 107 | CHEMICAL\_HOMEOSTASIS |  | 55 | 0.37 | 1.45 | 0.037 | 0.112 | 1.000 | 1639 | tags=44%, list=23%, signal=56% |
| 108 | RHO\_PROTEIN\_SIGNAL\_TRANSDUCTION |  | 20 | 0.47 | 1.45 | 0.047 | 0.112 | 1.000 | 754 | tags=25%, list=11%, signal=28% |
| 109 | NEURON\_DIFFERENTIATION |  | 19 | 0.46 | 1.44 | 0.064 | 0.115 | 1.000 | 1316 | tags=37%, list=19%, signal=45% |
| 110 | ANATOMICAL\_STRUCTURE\_FORMATION |  | 24 | 0.44 | 1.44 | 0.050 | 0.114 | 1.000 | 917 | tags=29%, list=13%, signal=33% |
| 111 | GUANYL\_NUCLEOTIDE\_EXCHANGE\_FACTOR\_ACTIVITY |  | 18 | 0.48 | 1.44 | 0.079 | 0.113 | 1.000 | 754 | tags=28%, list=11%, signal=31% |
| 112 | RESPONSE\_TO\_OTHER\_ORGANISM |  | 27 | 0.42 | 1.44 | 0.075 | 0.114 | 1.000 | 761 | tags=33%, list=11%, signal=37% |
| 113 | CATION\_TRANSPORT |  | 36 | 0.39 | 1.43 | 0.034 | 0.119 | 1.000 | 1122 | tags=36%, list=16%, signal=43% |
| 114 | PROTEIN\_COMPLEX\_BINDING |  | 22 | 0.45 | 1.43 | 0.065 | 0.122 | 1.000 | 426 | tags=18%, list=6%, signal=19% |
| 115 | SUBSTRATE\_SPECIFIC\_CHANNEL\_ACTIVITY |  | 25 | 0.43 | 1.43 | 0.062 | 0.122 | 1.000 | 1122 | tags=40%, list=16%, signal=47% |
| 116 | NEURON\_DEVELOPMENT |  | 15 | 0.49 | 1.43 | 0.077 | 0.122 | 1.000 | 1316 | tags=40%, list=19%, signal=49% |
| 117 | PROTEIN\_DOMAIN\_SPECIFIC\_BINDING |  | 21 | 0.45 | 1.43 | 0.067 | 0.121 | 1.000 | 1486 | tags=38%, list=21%, signal=48% |
| 118 | AMINE\_TRANSPORT |  | 15 | 0.50 | 1.42 | 0.079 | 0.123 | 1.000 | 914 | tags=33%, list=13%, signal=38% |
| 119 | POSITIVE\_REGULATION\_OF\_SIGNAL\_TRANSDUCTION |  | 51 | 0.36 | 1.42 | 0.047 | 0.126 | 1.000 | 1580 | tags=35%, list=22%, signal=45% |
| 120 | PEPTIDYL\_AMINO\_ACID\_MODIFICATION |  | 19 | 0.46 | 1.41 | 0.078 | 0.127 | 1.000 | 1545 | tags=53%, list=22%, signal=67% |
| 121 | POSITIVE\_REGULATION\_OF\_CELLULAR\_PROCESS |  | 230 | 0.28 | 1.41 | 0.005 | 0.128 | 1.000 | 1583 | tags=31%, list=22%, signal=39% |
| 122 | VASCULATURE\_DEVELOPMENT |  | 25 | 0.43 | 1.41 | 0.067 | 0.130 | 1.000 | 623 | tags=24%, list=9%, signal=26% |
| 123 | HOMEOSTATIC\_PROCESS |  | 74 | 0.34 | 1.40 | 0.032 | 0.134 | 1.000 | 497 | tags=19%, list=7%, signal=20% |
| 124 | TRANSMEMBRANE\_RECEPTOR\_PROTEIN\_TYROSINE\_KINASE\_ACTIVITY |  | 17 | 0.45 | 1.39 | 0.110 | 0.146 | 1.000 | 1225 | tags=35%, list=17%, signal=43% |
| 125 | LIPASE\_ACTIVITY |  | 15 | 0.49 | 1.38 | 0.106 | 0.147 | 1.000 | 578 | tags=33%, list=8%, signal=36% |
| 126 | SOLUBLE\_FRACTION |  | 57 | 0.33 | 1.37 | 0.045 | 0.160 | 1.000 | 363 | tags=16%, list=5%, signal=17% |
| 127 | SKELETAL\_DEVELOPMENT |  | 28 | 0.39 | 1.36 | 0.091 | 0.162 | 1.000 | 864 | tags=29%, list=12%, signal=32% |
| 128 | REGULATION\_OF\_CELL\_GROWTH |  | 17 | 0.45 | 1.36 | 0.122 | 0.161 | 1.000 | 109 | tags=12%, list=2%, signal=12% |
| 129 | GROWTH\_FACTOR\_ACTIVITY |  | 19 | 0.43 | 1.36 | 0.093 | 0.164 | 1.000 | 909 | tags=26%, list=13%, signal=30% |
| 130 | POSITIVE\_REGULATION\_OF\_CELLULAR\_PROTEIN\_METABOLIC\_PROCESS |  | 23 | 0.41 | 1.34 | 0.099 | 0.178 | 1.000 | 1545 | tags=48%, list=22%, signal=61% |
| 131 | MONOVALENT\_INORGANIC\_CATION\_TRANSPORT |  | 17 | 0.44 | 1.34 | 0.099 | 0.178 | 1.000 | 939 | tags=35%, list=13%, signal=41% |
| 132 | ACTIN\_BINDING |  | 33 | 0.38 | 1.34 | 0.100 | 0.178 | 1.000 | 725 | tags=24%, list=10%, signal=27% |
| 133 | REGULATION\_OF\_RESPONSE\_TO\_STIMULUS |  | 19 | 0.43 | 1.33 | 0.126 | 0.187 | 1.000 | 1146 | tags=21%, list=16%, signal=25% |
| 134 | GTPASE\_ACTIVATOR\_ACTIVITY |  | 19 | 0.44 | 1.33 | 0.124 | 0.191 | 1.000 | 1286 | tags=42%, list=18%, signal=51% |
| 135 | ACTIN\_FILAMENT\_BASED\_PROCESS |  | 37 | 0.36 | 1.32 | 0.101 | 0.195 | 1.000 | 2099 | tags=51%, list=30%, signal=73% |
| 136 | POSITIVE\_REGULATION\_OF\_TRANSFERASE\_ACTIVITY |  | 37 | 0.36 | 1.32 | 0.091 | 0.198 | 1.000 | 1261 | tags=30%, list=18%, signal=36% |
| 137 | RESPONSE\_TO\_BIOTIC\_STIMULUS |  | 40 | 0.35 | 1.31 | 0.111 | 0.208 | 1.000 | 761 | tags=28%, list=11%, signal=31% |
| 138 | CYTOPLASMIC\_VESICLE |  | 43 | 0.34 | 1.31 | 0.110 | 0.209 | 1.000 | 1792 | tags=33%, list=25%, signal=43% |
| 139 | ACTIN\_CYTOSKELETON\_ORGANIZATION\_AND\_BIOGENESIS |  | 33 | 0.37 | 1.30 | 0.108 | 0.210 | 1.000 | 2099 | tags=55%, list=30%, signal=77% |
| 140 | NEUROGENESIS |  | 24 | 0.39 | 1.30 | 0.136 | 0.211 | 1.000 | 1316 | tags=33%, list=19%, signal=41% |
| 141 | CYTOPLASMIC\_MEMBRANE\_BOUND\_VESICLE |  | 43 | 0.34 | 1.29 | 0.099 | 0.222 | 1.000 | 1792 | tags=33%, list=25%, signal=43% |
| 142 | REGULATION\_OF\_PROTEIN\_KINASE\_ACTIVITY |  | 61 | 0.31 | 1.29 | 0.085 | 0.226 | 1.000 | 830 | tags=18%, list=12%, signal=20% |
| 143 | PROTEIN\_SERINE\_THREONINE\_KINASE\_ACTIVITY |  | 82 | 0.29 | 1.28 | 0.091 | 0.232 | 1.000 | 2236 | tags=39%, list=32%, signal=56% |
| 144 | STRUCTURAL\_MOLECULE\_ACTIVITY |  | 58 | 0.32 | 1.28 | 0.121 | 0.232 | 1.000 | 628 | tags=19%, list=9%, signal=21% |
| 145 | PROTEIN\_TYROSINE\_KINASE\_ACTIVITY |  | 25 | 0.38 | 1.27 | 0.140 | 0.236 | 1.000 | 1367 | tags=32%, list=19%, signal=40% |
| 146 | CELL\_CELL\_ADHESION |  | 28 | 0.37 | 1.27 | 0.152 | 0.243 | 1.000 | 1459 | tags=36%, list=21%, signal=45% |
| 147 | REGULATION\_OF\_TRANSFERASE\_ACTIVITY |  | 63 | 0.30 | 1.26 | 0.116 | 0.247 | 1.000 | 1261 | tags=24%, list=18%, signal=29% |
| 148 | REGULATION\_OF\_KINASE\_ACTIVITY |  | 63 | 0.30 | 1.26 | 0.108 | 0.246 | 1.000 | 1261 | tags=24%, list=18%, signal=29% |
| 149 | CARBOHYDRATE\_BINDING |  | 17 | 0.42 | 1.26 | 0.174 | 0.251 | 1.000 | 1139 | tags=35%, list=16%, signal=42% |
| 150 | PROTEIN\_KINASE\_ACTIVITY |  | 117 | 0.28 | 1.26 | 0.084 | 0.252 | 1.000 | 1225 | tags=20%, list=17%, signal=23% |
| 151 | CYTOSKELETON\_ORGANIZATION\_AND\_BIOGENESIS |  | 66 | 0.31 | 1.26 | 0.108 | 0.251 | 1.000 | 1639 | tags=38%, list=23%, signal=49% |
| 152 | REGULATION\_OF\_I\_KAPPAB\_KINASE\_NF\_KAPPAB\_CASCADE |  | 38 | 0.34 | 1.25 | 0.175 | 0.253 | 1.000 | 1192 | tags=26%, list=17%, signal=31% |
| 153 | CELL\_SURFACE |  | 25 | 0.37 | 1.25 | 0.145 | 0.252 | 1.000 | 1143 | tags=48%, list=16%, signal=57% |
| 154 | POSITIVE\_REGULATION\_OF\_I\_KAPPAB\_KINASE\_NF\_KAPPAB\_CASCADE |  | 38 | 0.34 | 1.25 | 0.118 | 0.254 | 1.000 | 1192 | tags=26%, list=17%, signal=31% |
| 155 | POSITIVE\_REGULATION\_OF\_PROTEIN\_METABOLIC\_PROCESS |  | 24 | 0.38 | 1.25 | 0.158 | 0.254 | 1.000 | 1545 | tags=46%, list=22%, signal=58% |
| 156 | POSITIVE\_REGULATION\_OF\_TRANSCRIPTION\_FROM\_RNA\_POLYMERASE\_II\_PROMOTER |  | 21 | 0.39 | 1.25 | 0.170 | 0.254 | 1.000 | 1233 | tags=29%, list=17%, signal=34% |
| 157 | PROTEIN\_TYROSINE\_PHOSPHATASE\_ACTIVITY |  | 19 | 0.39 | 1.24 | 0.168 | 0.269 | 1.000 | 2402 | tags=74%, list=34%, signal=111% |
| 158 | PROTEIN\_DIMERIZATION\_ACTIVITY |  | 67 | 0.30 | 1.23 | 0.126 | 0.269 | 1.000 | 686 | tags=18%, list=10%, signal=20% |
| 159 | REGULATION\_OF\_CATALYTIC\_ACTIVITY |  | 104 | 0.27 | 1.23 | 0.085 | 0.270 | 1.000 | 830 | tags=18%, list=12%, signal=20% |
| 160 | RESPONSE\_TO\_STRESS |  | 196 | 0.25 | 1.23 | 0.043 | 0.273 | 1.000 | 1033 | tags=21%, list=15%, signal=24% |
| 161 | TRANSCRIPTION\_COREPRESSOR\_ACTIVITY |  | 39 | 0.33 | 1.23 | 0.172 | 0.272 | 1.000 | 582 | tags=15%, list=8%, signal=17% |
| 162 | STRUCTURAL\_CONSTITUENT\_OF\_CYTOSKELETON |  | 15 | 0.42 | 1.22 | 0.203 | 0.278 | 1.000 | 1801 | tags=53%, list=25%, signal=71% |
| 163 | IDENTICAL\_PROTEIN\_BINDING |  | 123 | 0.26 | 1.22 | 0.073 | 0.277 | 1.000 | 1526 | tags=28%, list=22%, signal=36% |
| 164 | SEXUAL\_REPRODUCTION |  | 35 | 0.34 | 1.22 | 0.155 | 0.277 | 1.000 | 1402 | tags=31%, list=20%, signal=39% |
| 165 | I\_KAPPAB\_KINASE\_NF\_KAPPAB\_CASCADE |  | 44 | 0.32 | 1.21 | 0.155 | 0.288 | 1.000 | 1192 | tags=25%, list=17%, signal=30% |
| 166 | POST\_TRANSLATIONAL\_PROTEIN\_MODIFICATION |  | 174 | 0.25 | 1.21 | 0.076 | 0.290 | 1.000 | 919 | tags=17%, list=13%, signal=19% |
| 167 | G\_PROTEIN\_COUPLED\_RECEPTOR\_ACTIVITY |  | 28 | 0.35 | 1.20 | 0.194 | 0.307 | 1.000 | 1777 | tags=39%, list=25%, signal=52% |
| 168 | POSITIVE\_REGULATION\_OF\_CELLULAR\_METABOLIC\_PROCESS |  | 70 | 0.29 | 1.19 | 0.146 | 0.321 | 1.000 | 1545 | tags=34%, list=22%, signal=43% |
| 169 | PROTEIN\_HOMODIMERIZATION\_ACTIVITY |  | 46 | 0.31 | 1.19 | 0.194 | 0.319 | 1.000 | 641 | tags=17%, list=9%, signal=19% |
| 170 | LEADING\_EDGE |  | 20 | 0.38 | 1.19 | 0.218 | 0.318 | 1.000 | 2226 | tags=55%, list=31%, signal=80% |
| 171 | BIOPOLYMER\_MODIFICATION |  | 236 | 0.23 | 1.19 | 0.056 | 0.318 | 1.000 | 840 | tags=15%, list=12%, signal=16% |
| 172 | PROTEIN\_BINDING\_BRIDGING |  | 23 | 0.37 | 1.18 | 0.215 | 0.322 | 1.000 | 1360 | tags=35%, list=19%, signal=43% |
| 173 | PROTEIN\_MODIFICATION\_PROCESS |  | 229 | 0.23 | 1.18 | 0.073 | 0.322 | 1.000 | 919 | tags=16%, list=13%, signal=17% |
| 174 | REGULATION\_OF\_PROTEIN\_METABOLIC\_PROCESS |  | 57 | 0.29 | 1.18 | 0.198 | 0.320 | 1.000 | 1545 | tags=39%, list=22%, signal=49% |
| 175 | NEGATIVE\_REGULATION\_OF\_CATALYTIC\_ACTIVITY |  | 20 | 0.37 | 1.18 | 0.224 | 0.320 | 1.000 | 1066 | tags=25%, list=15%, signal=29% |
| 176 | DNA\_BINDING |  | 175 | 0.24 | 1.18 | 0.081 | 0.323 | 1.000 | 1389 | tags=27%, list=20%, signal=33% |
| 177 | POSITIVE\_REGULATION\_OF\_CATALYTIC\_ACTIVITY |  | 64 | 0.29 | 1.18 | 0.179 | 0.321 | 1.000 | 1306 | tags=28%, list=18%, signal=34% |
| 178 | CELL\_FRACTION |  | 157 | 0.24 | 1.17 | 0.107 | 0.327 | 1.000 | 928 | tags=16%, list=13%, signal=18% |
| 179 | PHOSPHORYLATION |  | 115 | 0.25 | 1.16 | 0.146 | 0.352 | 1.000 | 894 | tags=17%, list=13%, signal=19% |
| 180 | ENZYME\_ACTIVATOR\_ACTIVITY |  | 47 | 0.30 | 1.15 | 0.206 | 0.361 | 1.000 | 1286 | tags=30%, list=18%, signal=36% |
| 181 | ADENYL\_RIBONUCLEOTIDE\_BINDING |  | 61 | 0.28 | 1.15 | 0.218 | 0.359 | 1.000 | 320 | tags=11%, list=5%, signal=12% |
| 182 | POSITIVE\_REGULATION\_OF\_METABOLIC\_PROCESS |  | 73 | 0.27 | 1.15 | 0.171 | 0.358 | 1.000 | 1545 | tags=33%, list=22%, signal=42% |
| 183 | PROTEIN\_C\_TERMINUS\_BINDING |  | 29 | 0.33 | 1.15 | 0.257 | 0.359 | 1.000 | 756 | tags=21%, list=11%, signal=23% |
| 184 | NERVOUS\_SYSTEM\_DEVELOPMENT |  | 90 | 0.26 | 1.14 | 0.218 | 0.365 | 1.000 | 1318 | tags=28%, list=19%, signal=34% |
| 185 | GROWTH |  | 26 | 0.34 | 1.14 | 0.289 | 0.365 | 1.000 | 1438 | tags=27%, list=20%, signal=34% |
| 186 | CALCIUM\_ION\_BINDING |  | 33 | 0.32 | 1.14 | 0.235 | 0.369 | 1.000 | 1716 | tags=52%, list=24%, signal=68% |
| 187 | KINASE\_REGULATOR\_ACTIVITY |  | 16 | 0.39 | 1.14 | 0.268 | 0.373 | 1.000 | 1010 | tags=25%, list=14%, signal=29% |
| 188 | MEMBRANE\_FRACTION |  | 110 | 0.25 | 1.12 | 0.196 | 0.394 | 1.000 | 1663 | tags=27%, list=23%, signal=35% |
| 189 | REGULATION\_OF\_MOLECULAR\_FUNCTION |  | 122 | 0.24 | 1.12 | 0.220 | 0.405 | 1.000 | 830 | tags=18%, list=12%, signal=20% |
| 190 | ADENYL\_NUCLEOTIDE\_BINDING |  | 62 | 0.27 | 1.11 | 0.246 | 0.408 | 1.000 | 320 | tags=11%, list=5%, signal=12% |
| 191 | NEGATIVE\_REGULATION\_OF\_CELL\_PROLIFERATION |  | 47 | 0.29 | 1.11 | 0.261 | 0.407 | 1.000 | 615 | tags=13%, list=9%, signal=14% |
| 192 | CYTOSKELETAL\_PROTEIN\_BINDING |  | 60 | 0.27 | 1.11 | 0.305 | 0.409 | 1.000 | 725 | tags=18%, list=10%, signal=20% |
| 193 | REGULATION\_OF\_TRANSLATION |  | 27 | 0.32 | 1.10 | 0.298 | 0.430 | 1.000 | 761 | tags=30%, list=11%, signal=33% |
| 194 | NUCLEOTIDE\_BINDING |  | 87 | 0.25 | 1.09 | 0.261 | 0.444 | 1.000 | 320 | tags=9%, list=5%, signal=10% |
| 195 | REGULATION\_OF\_GROWTH |  | 20 | 0.35 | 1.09 | 0.330 | 0.442 | 1.000 | 109 | tags=10%, list=2%, signal=10% |
| 196 | REGULATION\_OF\_DEVELOPMENTAL\_PROCESS |  | 163 | 0.22 | 1.09 | 0.247 | 0.447 | 1.000 | 754 | tags=15%, list=11%, signal=17% |
| 197 | METAL\_ION\_TRANSPORT |  | 23 | 0.34 | 1.08 | 0.332 | 0.461 | 1.000 | 1122 | tags=30%, list=16%, signal=36% |
| 198 | REGULATION\_OF\_CELLULAR\_PROTEIN\_METABOLIC\_PROCESS |  | 52 | 0.28 | 1.08 | 0.335 | 0.467 | 1.000 | 1545 | tags=38%, list=22%, signal=49% |
| 199 | ATP\_BINDING |  | 58 | 0.27 | 1.08 | 0.316 | 0.466 | 1.000 | 320 | tags=10%, list=5%, signal=11% |
| 200 | PROTEIN\_METABOLIC\_PROCESS |  | 442 | 0.19 | 1.07 | 0.139 | 0.474 | 1.000 | 1010 | tags=17%, list=14%, signal=19% |
| 201 | EPIDERMIS\_DEVELOPMENT |  | 16 | 0.36 | 1.07 | 0.363 | 0.482 | 1.000 | 1033 | tags=31%, list=15%, signal=36% |
| 202 | ECTODERM\_DEVELOPMENT |  | 17 | 0.35 | 1.06 | 0.354 | 0.484 | 1.000 | 1033 | tags=29%, list=15%, signal=34% |
| 203 | NEGATIVE\_REGULATION\_OF\_DEVELOPMENTAL\_PROCESS |  | 77 | 0.25 | 1.06 | 0.327 | 0.487 | 1.000 | 682 | tags=16%, list=10%, signal=17% |
| 204 | CYTOSKELETON |  | 113 | 0.23 | 1.04 | 0.330 | 0.524 | 1.000 | 1388 | tags=24%, list=20%, signal=29% |
| 205 | MEMBRANE\_BOUND\_VESICLE |  | 44 | 0.28 | 1.04 | 0.400 | 0.532 | 1.000 | 1792 | tags=32%, list=25%, signal=42% |
| 206 | PROTEIN\_HETERODIMERIZATION\_ACTIVITY |  | 30 | 0.29 | 1.04 | 0.376 | 0.530 | 1.000 | 686 | tags=20%, list=10%, signal=22% |
| 207 | NEGATIVE\_REGULATION\_OF\_BIOLOGICAL\_PROCESS |  | 234 | 0.21 | 1.03 | 0.336 | 0.538 | 1.000 | 1441 | tags=23%, list=20%, signal=27% |
| 208 | VESICLE |  | 45 | 0.27 | 1.03 | 0.397 | 0.551 | 1.000 | 1792 | tags=31%, list=25%, signal=41% |
| 209 | NEGATIVE\_REGULATION\_OF\_CELLULAR\_PROCESS |  | 222 | 0.20 | 1.03 | 0.368 | 0.552 | 1.000 | 1441 | tags=23%, list=20%, signal=27% |
| 210 | MUSCLE\_DEVELOPMENT |  | 27 | 0.30 | 1.03 | 0.400 | 0.553 | 1.000 | 832 | tags=19%, list=12%, signal=21% |
| 211 | PURINE\_RIBONUCLEOTIDE\_BINDING |  | 78 | 0.24 | 1.03 | 0.371 | 0.551 | 1.000 | 320 | tags=9%, list=5%, signal=9% |
| 212 | PHOSPHORIC\_ESTER\_HYDROLASE\_ACTIVITY |  | 53 | 0.26 | 1.03 | 0.417 | 0.548 | 1.000 | 907 | tags=23%, list=13%, signal=26% |
| 213 | MAGNESIUM\_ION\_BINDING |  | 20 | 0.33 | 1.02 | 0.413 | 0.553 | 1.000 | 147 | tags=10%, list=2%, signal=10% |
| 214 | RHODOPSIN\_LIKE\_RECEPTOR\_ACTIVITY |  | 17 | 0.35 | 1.02 | 0.457 | 0.554 | 1.000 | 781 | tags=24%, list=11%, signal=26% |
| 215 | PURINE\_NUCLEOTIDE\_BINDING |  | 79 | 0.23 | 1.02 | 0.413 | 0.554 | 1.000 | 320 | tags=9%, list=5%, signal=9% |
| 216 | PROTEIN\_PROCESSING |  | 17 | 0.34 | 1.02 | 0.433 | 0.561 | 1.000 | 651 | tags=18%, list=9%, signal=19% |
| 217 | REGULATION\_OF\_GENE\_EXPRESSION |  | 207 | 0.20 | 1.01 | 0.434 | 0.572 | 1.000 | 1536 | tags=27%, list=22%, signal=34% |
| 218 | CYTOSKELETAL\_PART |  | 60 | 0.25 | 1.00 | 0.477 | 0.585 | 1.000 | 1696 | tags=28%, list=24%, signal=37% |
| 219 | SYSTEM\_PROCESS |  | 137 | 0.21 | 1.00 | 0.428 | 0.584 | 1.000 | 1240 | tags=23%, list=17%, signal=27% |
| 220 | CELLULAR\_PROTEIN\_METABOLIC\_PROCESS |  | 395 | 0.19 | 0.99 | 0.466 | 0.603 | 1.000 | 919 | tags=16%, list=13%, signal=17% |
| 221 | RESPONSE\_TO\_VIRUS |  | 20 | 0.31 | 0.99 | 0.485 | 0.612 | 1.000 | 1639 | tags=45%, list=23%, signal=58% |
| 222 | PROTEIN\_DNA\_COMPLEX\_ASSEMBLY |  | 20 | 0.32 | 0.98 | 0.492 | 0.636 | 1.000 | 96 | tags=10%, list=1%, signal=10% |
| 223 | CELLULAR\_MACROMOLECULE\_METABOLIC\_PROCESS |  | 398 | 0.18 | 0.97 | 0.591 | 0.644 | 1.000 | 919 | tags=16%, list=13%, signal=17% |
| 224 | TRANSCRIPTION\_REPRESSOR\_ACTIVITY |  | 58 | 0.24 | 0.97 | 0.515 | 0.650 | 1.000 | 718 | tags=12%, list=10%, signal=13% |
| 225 | CELL\_JUNCTION |  | 27 | 0.28 | 0.96 | 0.486 | 0.664 | 1.000 | 844 | tags=15%, list=12%, signal=17% |
| 226 | GAMETE\_GENERATION |  | 30 | 0.28 | 0.96 | 0.531 | 0.679 | 1.000 | 1402 | tags=27%, list=20%, signal=33% |
| 227 | HYDROLASE\_ACTIVITY\_ACTING\_ON\_ESTER\_BONDS |  | 86 | 0.22 | 0.95 | 0.556 | 0.684 | 1.000 | 907 | tags=19%, list=13%, signal=21% |
| 228 | TISSUE\_DEVELOPMENT |  | 31 | 0.27 | 0.94 | 0.525 | 0.702 | 1.000 | 1033 | tags=19%, list=15%, signal=23% |
| 229 | TRANSCRIPTION\_FACTOR\_BINDING |  | 103 | 0.21 | 0.94 | 0.606 | 0.716 | 1.000 | 1596 | tags=27%, list=23%, signal=35% |
| 230 | TRANSLATION |  | 54 | 0.24 | 0.94 | 0.576 | 0.718 | 1.000 | 761 | tags=22%, list=11%, signal=25% |
| 231 | GTPASE\_BINDING |  | 15 | 0.32 | 0.92 | 0.559 | 0.741 | 1.000 | 2045 | tags=47%, list=29%, signal=65% |
| 232 | ACTIN\_CYTOSKELETON |  | 48 | 0.24 | 0.92 | 0.591 | 0.739 | 1.000 | 725 | tags=17%, list=10%, signal=18% |
| 233 | REGULATION\_OF\_MITOSIS |  | 18 | 0.30 | 0.92 | 0.596 | 0.751 | 1.000 | 599 | tags=17%, list=8%, signal=18% |
| 234 | SMALL\_GTPASE\_BINDING |  | 15 | 0.32 | 0.90 | 0.620 | 0.781 | 1.000 | 2045 | tags=47%, list=29%, signal=65% |
| 235 | PHOSPHOTRANSFERASE\_ACTIVITY\_ALCOHOL\_GROUP\_AS\_ACCEPTOR |  | 140 | 0.19 | 0.90 | 0.724 | 0.778 | 1.000 | 1225 | tags=17%, list=17%, signal=20% |
| 236 | ANTI\_APOPTOSIS |  | 47 | 0.24 | 0.90 | 0.663 | 0.789 | 1.000 | 401 | tags=13%, list=6%, signal=13% |
| 237 | POSITIVE\_REGULATION\_OF\_DEVELOPMENTAL\_PROCESS |  | 80 | 0.21 | 0.90 | 0.678 | 0.788 | 1.000 | 1633 | tags=31%, list=23%, signal=40% |
| 238 | NUCLEOBASENUCLEOSIDE\_AND\_NUCLEOTIDE\_METABOLIC\_PROCESS |  | 22 | 0.28 | 0.88 | 0.637 | 0.829 | 1.000 | 373 | tags=18%, list=5%, signal=19% |
| 239 | REGULATION\_OF\_METABOLIC\_PROCESS |  | 256 | 0.17 | 0.87 | 0.877 | 0.836 | 1.000 | 1545 | tags=26%, list=22%, signal=32% |
| 240 | TRANSFERASE\_ACTIVITY\_TRANSFERRING\_HEXOSYL\_GROUPS |  | 27 | 0.26 | 0.87 | 0.665 | 0.838 | 1.000 | 1020 | tags=26%, list=14%, signal=30% |
| 241 | REGULATION\_OF\_CELLULAR\_METABOLIC\_PROCESS |  | 251 | 0.17 | 0.87 | 0.877 | 0.839 | 1.000 | 1545 | tags=25%, list=22%, signal=31% |
| 242 | STEROID\_METABOLIC\_PROCESS |  | 20 | 0.27 | 0.86 | 0.714 | 0.864 | 1.000 | 1575 | tags=40%, list=22%, signal=51% |
| 243 | KINASE\_ACTIVITY |  | 151 | 0.18 | 0.85 | 0.850 | 0.866 | 1.000 | 1248 | tags=19%, list=18%, signal=22% |
| 244 | CENTRAL\_NERVOUS\_SYSTEM\_DEVELOPMENT |  | 30 | 0.25 | 0.85 | 0.712 | 0.874 | 1.000 | 483 | tags=13%, list=7%, signal=14% |
| 245 | CELL\_DEVELOPMENT |  | 200 | 0.17 | 0.85 | 0.925 | 0.872 | 1.000 | 682 | tags=12%, list=10%, signal=13% |
| 246 | TRANSCRIPTION\_FROM\_RNA\_POLYMERASE\_II\_PROMOTER |  | 155 | 0.18 | 0.85 | 0.869 | 0.870 | 1.000 | 1146 | tags=19%, list=16%, signal=23% |
| 247 | INTERPHASE\_OF\_MITOTIC\_CELL\_CYCLE |  | 27 | 0.24 | 0.85 | 0.717 | 0.866 | 1.000 | 738 | tags=15%, list=10%, signal=16% |
| 248 | REGULATION\_OF\_TRANSCRIPTION |  | 177 | 0.17 | 0.84 | 0.904 | 0.865 | 1.000 | 1536 | tags=25%, list=22%, signal=31% |
| 249 | GLYCOPROTEIN\_METABOLIC\_PROCESS |  | 29 | 0.24 | 0.84 | 0.705 | 0.877 | 1.000 | 324 | tags=10%, list=5%, signal=11% |
| 250 | MITOTIC\_CELL\_CYCLE |  | 59 | 0.20 | 0.83 | 0.783 | 0.892 | 1.000 | 912 | tags=15%, list=13%, signal=17% |
| 251 | ENZYME\_LINKED\_RECEPTOR\_PROTEIN\_SIGNALING\_PATHWAY |  | 45 | 0.21 | 0.82 | 0.808 | 0.905 | 1.000 | 1367 | tags=29%, list=19%, signal=36% |
| 252 | POSITIVE\_REGULATION\_OF\_TRANSCRIPTION |  | 44 | 0.21 | 0.81 | 0.805 | 0.911 | 1.000 | 1770 | tags=32%, list=25%, signal=42% |
| 253 | ENDOPLASMIC\_RETICULUM |  | 107 | 0.18 | 0.81 | 0.890 | 0.917 | 1.000 | 1575 | tags=27%, list=22%, signal=34% |
| 254 | REGULATION\_OF\_NUCLEOBASENUCLEOSIDENUCLEOTIDE\_AND\_NUCLEIC\_ACID\_METABOLIC\_PROCESS |  | 196 | 0.16 | 0.80 | 0.973 | 0.923 | 1.000 | 908 | tags=15%, list=13%, signal=16% |
| 255 | REGULATION\_OF\_RNA\_METABOLIC\_PROCESS |  | 148 | 0.17 | 0.79 | 0.950 | 0.929 | 1.000 | 1536 | tags=24%, list=22%, signal=30% |
| 256 | POSITIVE\_REGULATION\_OF\_TRANSCRIPTIONDNA\_DEPENDENT |  | 38 | 0.21 | 0.79 | 0.819 | 0.934 | 1.000 | 1536 | tags=26%, list=22%, signal=33% |
| 257 | PROTEIN\_AMINO\_ACID\_DEPHOSPHORYLATION |  | 21 | 0.25 | 0.79 | 0.777 | 0.935 | 1.000 | 919 | tags=19%, list=13%, signal=22% |
| 258 | INTERPHASE |  | 28 | 0.23 | 0.78 | 0.803 | 0.945 | 1.000 | 738 | tags=14%, list=10%, signal=16% |
| 259 | POSITIVE\_REGULATION\_OF\_RNA\_METABOLIC\_PROCESS |  | 38 | 0.21 | 0.77 | 0.843 | 0.949 | 1.000 | 1536 | tags=26%, list=22%, signal=33% |
| 260 | PHOSPHOPROTEIN\_PHOSPHATASE\_ACTIVITY |  | 28 | 0.22 | 0.77 | 0.847 | 0.948 | 1.000 | 827 | tags=18%, list=12%, signal=20% |
| 261 | CELL\_CYCLE\_PHASE |  | 60 | 0.19 | 0.77 | 0.884 | 0.948 | 1.000 | 912 | tags=15%, list=13%, signal=17% |
| 262 | REGULATION\_OF\_TRANSCRIPTIONDNA\_DEPENDENT |  | 145 | 0.16 | 0.76 | 0.965 | 0.947 | 1.000 | 1536 | tags=24%, list=22%, signal=30% |
| 263 | TRANSCRIPTION\_COFACTOR\_ACTIVITY |  | 75 | 0.18 | 0.76 | 0.939 | 0.953 | 1.000 | 1109 | tags=16%, list=16%, signal=19% |
| 264 | MITOSIS |  | 31 | 0.21 | 0.74 | 0.868 | 0.971 | 1.000 | 1392 | tags=23%, list=20%, signal=28% |
| 265 | GTPASE\_ACTIVITY |  | 41 | 0.20 | 0.73 | 0.910 | 0.973 | 1.000 | 2355 | tags=44%, list=33%, signal=65% |
| 266 | M\_PHASE\_OF\_MITOTIC\_CELL\_CYCLE |  | 32 | 0.21 | 0.73 | 0.888 | 0.977 | 1.000 | 1392 | tags=22%, list=20%, signal=27% |
| 267 | GOLGI\_APPARATUS |  | 86 | 0.17 | 0.72 | 0.978 | 0.983 | 1.000 | 1513 | tags=20%, list=21%, signal=25% |
| 268 | HYDROLASE\_ACTIVITY\_ACTING\_ON\_GLYCOSYL\_BONDS |  | 20 | 0.22 | 0.70 | 0.904 | 0.993 | 1.000 | 324 | tags=10%, list=5%, signal=10% |
| 269 | REGULATION\_OF\_TRANSCRIPTION\_FROM\_RNA\_POLYMERASE\_II\_PROMOTER |  | 89 | 0.16 | 0.70 | 0.983 | 0.993 | 1.000 | 1233 | tags=16%, list=17%, signal=19% |
| 270 | NUCLEAR\_TRANSPORT |  | 35 | 0.19 | 0.69 | 0.928 | 0.992 | 1.000 | 618 | tags=11%, list=9%, signal=12% |
| 271 | NUCLEOCYTOPLASMIC\_TRANSPORT |  | 35 | 0.19 | 0.68 | 0.953 | 0.994 | 1.000 | 618 | tags=11%, list=9%, signal=12% |
| 272 | CELL\_CYCLE\_PROCESS |  | 67 | 0.16 | 0.68 | 0.983 | 0.996 | 1.000 | 755 | tags=12%, list=11%, signal=13% |
| 273 | TRANSMISSION\_OF\_NERVE\_IMPULSE |  | 42 | 0.17 | 0.67 | 0.962 | 0.999 | 1.000 | 2109 | tags=36%, list=30%, signal=51% |
| 274 | NEGATIVE\_REGULATION\_OF\_GROWTH |  | 15 | 0.22 | 0.65 | 0.918 | 1.000 | 1.000 | 109 | tags=7%, list=2%, signal=7% |
| 275 | REGULATION\_OF\_TRANSPORT |  | 19 | 0.21 | 0.65 | 0.945 | 1.000 | 1.000 | 2194 | tags=42%, list=31%, signal=61% |
| 276 | HELICASE\_ACTIVITY |  | 19 | 0.20 | 0.65 | 0.944 | 0.998 | 1.000 | 612 | tags=11%, list=9%, signal=11% |
| 277 | SERINE\_TYPE\_PEPTIDASE\_ACTIVITY |  | 15 | 0.22 | 0.64 | 0.934 | 0.998 | 1.000 | 1255 | tags=27%, list=18%, signal=32% |
| 278 | MICROTUBULE\_CYTOSKELETON |  | 45 | 0.17 | 0.64 | 0.977 | 0.995 | 1.000 | 1388 | tags=20%, list=20%, signal=25% |
| 279 | SEQUENCE\_SPECIFIC\_DNA\_BINDING |  | 17 | 0.21 | 0.64 | 0.914 | 0.991 | 1.000 | 1757 | tags=35%, list=25%, signal=47% |
| 280 | POSITIVE\_REGULATION\_OF\_NUCLEOBASENUCLEOSIDENUCLEOTIDE\_AND\_NUCLEIC\_ACID\_METABOLIC\_PROCESS |  | 48 | 0.16 | 0.63 | 0.988 | 0.992 | 1.000 | 1536 | tags=25%, list=22%, signal=32% |
| 281 | SERINE\_HYDROLASE\_ACTIVITY |  | 15 | 0.22 | 0.63 | 0.914 | 0.988 | 1.000 | 1255 | tags=27%, list=18%, signal=32% |
| 282 | TRANSCRIPTION\_FACTOR\_COMPLEX |  | 23 | 0.19 | 0.63 | 0.964 | 0.985 | 1.000 | 5736 | tags=100%, list=81%, signal=521% |
| 283 | NEGATIVE\_REGULATION\_OF\_TRANSCRIPTION\_FROM\_RNA\_POLYMERASE\_II\_PROMOTER |  | 25 | 0.19 | 0.63 | 0.951 | 0.984 | 1.000 | 1141 | tags=20%, list=16%, signal=24% |
| 284 | M\_PHASE |  | 35 | 0.17 | 0.62 | 0.980 | 0.984 | 1.000 | 912 | tags=14%, list=13%, signal=16% |
| 285 | COATED\_VESICLE |  | 20 | 0.17 | 0.55 | 0.987 | 0.997 | 1.000 | 5861 | tags=100%, list=83%, signal=574% |
| 286 | GOLGI\_MEMBRANE |  | 18 | 0.18 | 0.54 | 0.990 | 0.995 | 1.000 | 4649 | tags=94%, list=66%, signal=273% |
| 287 | SYNAPTIC\_TRANSMISSION |  | 38 | 0.13 | 0.50 | 1.000 | 0.996 | 1.000 | 2109 | tags=34%, list=30%, signal=48% |
Table: Gene sets enriched in phenotype **na**[plain text format]****

  
