# Supplementary material for: Network-based integration of molecular and physiological data elucidates regulatory mechanisms underlying adaptation to high-fat diet
Source: Genes Nutr. 2015 May 28;10(4):22. doi: 10.1007/s12263-015-0470-6 (PMC4446272; doi:10.1007/s12263-015-0470-6)
Supplement: Supplementary file 4 — Supplementary material 4 (ZIP 6984 kb) [file 12263_2015_470_MOESM4_ESM.zip › HF LF 12 w GSEA result/HEMOPOIETIC_OR_LYMPHOID_ORGAN_DEVELOPMENT.html]

Details for gene set HEMOPOIETIC\_OR\_LYMPHOID\_ORGAN\_DEVELOPMENT[GSEA]

|  || Dataset | HF LF 12w\_collapsed |
| Phenotype | NoPhenotypeAvailable |
| Upregulated in class | na\_pos |
| GeneSet | HEMOPOIETIC\_OR\_LYMPHOID\_ORGAN\_DEVELOPMENT |
| Enrichment Score (ES) | 0.60512394 |
| Normalized Enrichment Score (NES) | 2.1699367 |
| Nominal p-value | 0.0 |
| FDR q-value | 9.678731E-4 |
| FWER p-Value | 0.006 |
Table: GSEA Results Summary

  

Fig 1: Enrichment plot: HEMOPOIETIC\_OR\_LYMPHOID\_ORGAN\_DEVELOPMENT      
 Profile of the Running ES Score & Positions of GeneSet Members on the Rank Ordered List

  

| PROBE | GENE SYMBOL | GENE\_TITLE | RANK IN GENE LIST | RANK METRIC SCORE | RUNNING ES | CORE ENRICHMENT || 1 | MAP4K1 |  |  | 43 | 6.505 | 0.0941 | Yes |
| 2 | MAFB |  |  | 78 | 5.738 | 0.1776 | Yes |
| 3 | ACVR1B |  |  | 109 | 5.209 | 0.2536 | Yes |
| 4 | CALCA |  |  | 138 | 4.957 | 0.3260 | Yes |
| 5 | RUNX1 |  |  | 202 | 4.538 | 0.3869 | Yes |
| 6 | RASGRP4 |  |  | 331 | 3.801 | 0.4273 | Yes |
| 7 | CSF1 |  |  | 350 | 3.714 | 0.4820 | Yes |
| 8 | LRMP |  |  | 517 | 3.063 | 0.5056 | Yes |
| 9 | PF4 |  |  | 623 | 2.761 | 0.5333 | Yes |
| 10 | IL27 |  |  | 684 | 2.615 | 0.5650 | Yes |
| 11 | CD3D |  |  | 686 | 2.612 | 0.6051 | Yes |
| 12 | HCLS1 |  |  | 1377 | 1.562 | 0.5314 | No |
| 13 | ETS1 |  |  | 1428 | 1.504 | 0.5475 | No |
| 14 | NOTCH2 |  |  | 1438 | 1.496 | 0.5692 | No |
| 15 | IL31RA |  |  | 1545 | 1.384 | 0.5755 | No |
| 16 | HDAC4 |  |  | 1596 | 1.330 | 0.5889 | No |
| 17 | ACIN1 |  |  | 1870 | 1.001 | 0.5656 | No |
| 18 | NOTCH4 |  |  | 2008 | 0.850 | 0.5593 | No |
| 19 | RAB3D |  |  | 2021 | 0.839 | 0.5705 | No |
| 20 | TGFB1 |  |  | 2164 | 0.718 | 0.5615 | No |
| 21 | CDC42 |  |  | 2463 | 0.440 | 0.5260 | No |
| 22 | INHA |  |  | 2493 | 0.417 | 0.5283 | No |
| 23 | LDB1 |  |  | 2650 | 0.275 | 0.5104 | No |
| 24 | INHBA |  |  | 2769 | 0.194 | 0.4967 | No |
| 25 | DYRK3 |  |  | 2827 | 0.134 | 0.4907 | No |
| 26 | MLF1 |  |  | 2964 | 0.036 | 0.4720 | No |
| 27 | JAG2 |  |  | 3166 | -0.108 | 0.4452 | No |
| 28 | MMP9 |  |  | 3178 | -0.120 | 0.4454 | No |
| 29 | SOCS5 |  |  | 3209 | -0.142 | 0.4434 | No |
| 30 | TPD52 |  |  | 3393 | -0.264 | 0.4215 | No |
| 31 | TGFB2 |  |  | 3518 | -0.356 | 0.4094 | No |
| 32 | ALAS2 |  |  | 4053 | -0.741 | 0.3451 | No |
| 33 | SNRK |  |  | 4165 | -0.811 | 0.3419 | No |
| 34 | MAP4K2 |  |  | 4601 | -1.139 | 0.2978 | No |
| 35 | CEBPG |  |  | 4668 | -1.174 | 0.3065 | No |
| 36 | SCIN |  |  | 5892 | -2.386 | 0.1699 | No |
Table: GSEA details [plain text format]

  

Fig 2: HEMOPOIETIC\_OR\_LYMPHOID\_ORGAN\_DEVELOPMENT: Random ES distribution      
 Gene set null distribution of ES for **HEMOPOIETIC\_OR\_LYMPHOID\_ORGAN\_DEVELOPMENT**

  
