# Supplementary material for: Network-based integration of molecular and physiological data elucidates regulatory mechanisms underlying adaptation to high-fat diet
Source: Genes Nutr. 2015 May 28;10(4):22. doi: 10.1007/s12263-015-0470-6 (PMC4446272; doi:10.1007/s12263-015-0470-6)
Supplement: Supplementary file 4 — Supplementary material 4 (ZIP 6984 kb) [file 12263_2015_470_MOESM4_ESM.zip › HF LF 12 w GSEA result/IMMUNE_SYSTEM_PROCESS.html]

Details for gene set IMMUNE\_SYSTEM\_PROCESS[GSEA]

|  || Dataset | HF LF 12w\_collapsed |
| Phenotype | NoPhenotypeAvailable |
| Upregulated in class | na\_pos |
| GeneSet | IMMUNE\_SYSTEM\_PROCESS |
| Enrichment Score (ES) | 0.52583665 |
| Normalized Enrichment Score (NES) | 2.4960654 |
| Nominal p-value | 0.0 |
| FDR q-value | 0.0 |
| FWER p-Value | 0.0 |
Table: GSEA Results Summary

  

Fig 1: Enrichment plot: IMMUNE\_SYSTEM\_PROCESS      
 Profile of the Running ES Score & Positions of GeneSet Members on the Rank Ordered List

  

| PROBE | GENE SYMBOL | GENE\_TITLE | RANK IN GENE LIST | RANK METRIC SCORE | RUNNING ES | CORE ENRICHMENT || 1 | LTB4R |  |  | 14 | 7.889 | 0.0266 | Yes |
| 2 | CD83 |  |  | 40 | 6.736 | 0.0474 | Yes |
| 3 | MAP4K1 |  |  | 43 | 6.505 | 0.0706 | Yes |
| 4 | NCF4 |  |  | 75 | 5.806 | 0.0872 | Yes |
| 5 | MAFB |  |  | 78 | 5.738 | 0.1077 | Yes |
| 6 | ITGB2 |  |  | 86 | 5.478 | 0.1265 | Yes |
| 7 | IL7R |  |  | 99 | 5.294 | 0.1440 | Yes |
| 8 | C2 |  |  | 103 | 5.276 | 0.1627 | Yes |
| 9 | ACVR1B |  |  | 109 | 5.209 | 0.1808 | Yes |
| 10 | CCR2 |  |  | 112 | 5.179 | 0.1993 | Yes |
| 11 | CLEC7A |  |  | 121 | 5.093 | 0.2166 | Yes |
| 12 | CALCA |  |  | 138 | 4.957 | 0.2323 | Yes |
| 13 | CCL2 |  |  | 148 | 4.900 | 0.2487 | Yes |
| 14 | ARHGDIB |  |  | 159 | 4.863 | 0.2649 | Yes |
| 15 | CCBP2 |  |  | 175 | 4.685 | 0.2797 | Yes |
| 16 | BST1 |  |  | 199 | 4.554 | 0.2929 | Yes |
| 17 | RUNX1 |  |  | 202 | 4.538 | 0.3090 | Yes |
| 18 | CCL24 |  |  | 224 | 4.436 | 0.3221 | Yes |
| 19 | DOCK2 |  |  | 228 | 4.377 | 0.3375 | Yes |
| 20 | IGSF6 |  |  | 240 | 4.319 | 0.3516 | Yes |
| 21 | FCGR2B |  |  | 249 | 4.280 | 0.3659 | Yes |
| 22 | RASGRP4 |  |  | 331 | 3.801 | 0.3681 | Yes |
| 23 | CSF1 |  |  | 350 | 3.714 | 0.3789 | Yes |
| 24 | IRF8 |  |  | 447 | 3.323 | 0.3772 | Yes |
| 25 | CD2 |  |  | 451 | 3.299 | 0.3887 | Yes |
| 26 | LY86 |  |  | 457 | 3.277 | 0.3999 | Yes |
| 27 | CCL4 |  |  | 486 | 3.157 | 0.4073 | Yes |
| 28 | CCR5 |  |  | 497 | 3.131 | 0.4172 | Yes |
| 29 | WAS |  |  | 507 | 3.096 | 0.4271 | Yes |
| 30 | LRMP |  |  | 517 | 3.063 | 0.4369 | Yes |
| 31 | IL6ST |  |  | 544 | 2.976 | 0.4439 | Yes |
| 32 | ICOSLG |  |  | 546 | 2.975 | 0.4546 | Yes |
| 33 | CCRL1 |  |  | 581 | 2.868 | 0.4601 | Yes |
| 34 | PF4 |  |  | 623 | 2.761 | 0.4642 | Yes |
| 35 | EBI3 |  |  | 654 | 2.682 | 0.4696 | Yes |
| 36 | IL27 |  |  | 684 | 2.615 | 0.4749 | Yes |
| 37 | CD3D |  |  | 686 | 2.612 | 0.4842 | Yes |
| 38 | FCGRT |  |  | 698 | 2.580 | 0.4920 | Yes |
| 39 | FCGR3A |  |  | 737 | 2.484 | 0.4955 | Yes |
| 40 | CCL5 |  |  | 752 | 2.451 | 0.5024 | Yes |
| 41 | IFITM3 |  |  | 786 | 2.380 | 0.5063 | Yes |
| 42 | SIT1 |  |  | 811 | 2.338 | 0.5113 | Yes |
| 43 | SLA2 |  |  | 849 | 2.272 | 0.5142 | Yes |
| 44 | APLN |  |  | 858 | 2.253 | 0.5212 | Yes |
| 45 | ST6GAL1 |  |  | 883 | 2.224 | 0.5258 | Yes |
| 46 | IL27RA |  |  | 943 | 2.116 | 0.5250 | No |
| 47 | CD34 |  |  | 1032 | 1.995 | 0.5196 | No |
| 48 | RFX1 |  |  | 1077 | 1.939 | 0.5203 | No |
| 49 | SEMA3C |  |  | 1142 | 1.846 | 0.5178 | No |
| 50 | MBP |  |  | 1224 | 1.729 | 0.5125 | No |
| 51 | DPP4 |  |  | 1232 | 1.723 | 0.5177 | No |
| 52 | ELF4 |  |  | 1233 | 1.722 | 0.5239 | No |
| 53 | CD7 |  |  | 1335 | 1.604 | 0.5152 | No |
| 54 | HCLS1 |  |  | 1377 | 1.562 | 0.5150 | No |
| 55 | ETS1 |  |  | 1428 | 1.504 | 0.5133 | No |
| 56 | NOTCH2 |  |  | 1438 | 1.496 | 0.5174 | No |
| 57 | COLEC12 |  |  | 1451 | 1.484 | 0.5210 | No |
| 58 | BCL2 |  |  | 1486 | 1.441 | 0.5214 | No |
| 59 | IL31RA |  |  | 1545 | 1.384 | 0.5181 | No |
| 60 | HDAC4 |  |  | 1596 | 1.330 | 0.5157 | No |
| 61 | CXCL12 |  |  | 1639 | 1.275 | 0.5143 | No |
| 62 | GBP2 |  |  | 1655 | 1.252 | 0.5167 | No |
| 63 | GPR65 |  |  | 1749 | 1.157 | 0.5075 | No |
| 64 | LAT |  |  | 1798 | 1.084 | 0.5046 | No |
| 65 | ACIN1 |  |  | 1870 | 1.001 | 0.4980 | No |
| 66 | VIPR1 |  |  | 1990 | 0.868 | 0.4840 | No |
| 67 | NOTCH4 |  |  | 2008 | 0.850 | 0.4847 | No |
| 68 | RAB3D |  |  | 2021 | 0.839 | 0.4860 | No |
| 69 | BST2 |  |  | 2029 | 0.829 | 0.4880 | No |
| 70 | IL16 |  |  | 2050 | 0.812 | 0.4881 | No |
| 71 | PSMB10 |  |  | 2104 | 0.761 | 0.4832 | No |
| 72 | TGFB1 |  |  | 2164 | 0.718 | 0.4773 | No |
| 73 | IKBKAP |  |  | 2211 | 0.670 | 0.4732 | No |
| 74 | CDC42 |  |  | 2463 | 0.440 | 0.4387 | No |
| 75 | INHA |  |  | 2493 | 0.417 | 0.4361 | No |
| 76 | PTGER4 |  |  | 2543 | 0.368 | 0.4304 | No |
| 77 | LDB1 |  |  | 2650 | 0.275 | 0.4161 | No |
| 78 | APOA1 |  |  | 2721 | 0.230 | 0.4069 | No |
| 79 | MAP3K7 |  |  | 2733 | 0.222 | 0.4061 | No |
| 80 | INHBA |  |  | 2769 | 0.194 | 0.4018 | No |
| 81 | IKBKG |  |  | 2807 | 0.159 | 0.3971 | No |
| 82 | DYRK3 |  |  | 2827 | 0.134 | 0.3948 | No |
| 83 | TCF7 |  |  | 2936 | 0.056 | 0.3795 | No |
| 84 | MLF1 |  |  | 2964 | 0.036 | 0.3758 | No |
| 85 | JAG2 |  |  | 3166 | -0.108 | 0.3473 | No |
| 86 | MMP9 |  |  | 3178 | -0.120 | 0.3462 | No |
| 87 | SOCS5 |  |  | 3209 | -0.142 | 0.3424 | No |
| 88 | CD47 |  |  | 3284 | -0.189 | 0.3325 | No |
| 89 | IL15 |  |  | 3302 | -0.200 | 0.3307 | No |
| 90 | TRAF6 |  |  | 3354 | -0.239 | 0.3243 | No |
| 91 | CD274 |  |  | 3385 | -0.260 | 0.3209 | No |
| 92 | TPD52 |  |  | 3393 | -0.264 | 0.3209 | No |
| 93 | TGFB2 |  |  | 3518 | -0.356 | 0.3043 | No |
| 94 | UBE2N |  |  | 3766 | -0.533 | 0.2708 | No |
| 95 | LIG1 |  |  | 3907 | -0.641 | 0.2530 | No |
| 96 | LTF |  |  | 3976 | -0.690 | 0.2458 | No |
| 97 | ALAS2 |  |  | 4053 | -0.741 | 0.2375 | No |
| 98 | CD79B |  |  | 4060 | -0.745 | 0.2394 | No |
| 99 | CD24 |  |  | 4094 | -0.768 | 0.2374 | No |
| 100 | DPP8 |  |  | 4104 | -0.772 | 0.2389 | No |
| 101 | CXCR4 |  |  | 4118 | -0.780 | 0.2399 | No |
| 102 | SNRK |  |  | 4165 | -0.811 | 0.2362 | No |
| 103 | TCF12 |  |  | 4216 | -0.850 | 0.2321 | No |
| 104 | IK |  |  | 4228 | -0.857 | 0.2336 | No |
| 105 | CNIH |  |  | 4343 | -0.947 | 0.2207 | No |
| 106 | CX3CL1 |  |  | 4357 | -0.956 | 0.2223 | No |
| 107 | SECTM1 |  |  | 4442 | -1.017 | 0.2139 | No |
| 108 | VTN |  |  | 4496 | -1.059 | 0.2102 | No |
| 109 | FTH1 |  |  | 4558 | -1.100 | 0.2054 | No |
| 110 | MAP4K2 |  |  | 4601 | -1.139 | 0.2035 | No |
| 111 | CEBPG |  |  | 4668 | -1.174 | 0.1982 | No |
| 112 | GTPBP1 |  |  | 4743 | -1.223 | 0.1921 | No |
| 113 | ATP6V0A2 |  |  | 4967 | -1.405 | 0.1651 | No |
| 114 | MS4A1 |  |  | 5033 | -1.464 | 0.1611 | No |
| 115 | IL18BP |  |  | 5101 | -1.517 | 0.1570 | No |
| 116 | CD164 |  |  | 5141 | -1.547 | 0.1570 | No |
| 117 | SEMA7A |  |  | 5316 | -1.699 | 0.1381 | No |
| 118 | CTSC |  |  | 5413 | -1.805 | 0.1309 | No |
| 119 | IL28RA |  |  | 5450 | -1.848 | 0.1324 | No |
| 120 | BCL10 |  |  | 5604 | -2.007 | 0.1177 | No |
| 121 | CXCL13 |  |  | 5611 | -2.017 | 0.1242 | No |
| 122 | PRKRA |  |  | 5859 | -2.338 | 0.0972 | No |
| 123 | SCIN |  |  | 5892 | -2.386 | 0.1012 | No |
| 124 | CCL25 |  |  | 6223 | -2.843 | 0.0641 | No |
| 125 | RSAD2 |  |  | 6354 | -3.088 | 0.0567 | No |
| 126 | C1QBP |  |  | 6835 | -4.587 | 0.0044 | No |
| 127 | MR1 |  |  | 7075 | -8.907 | 0.0023 | No |
Table: GSEA details [plain text format]

  

Fig 2: IMMUNE\_SYSTEM\_PROCESS: Random ES distribution      
 Gene set null distribution of ES for **IMMUNE\_SYSTEM\_PROCESS**

  
