# Supplementary material for: Network-based integration of molecular and physiological data elucidates regulatory mechanisms underlying adaptation to high-fat diet
Source: Genes Nutr. 2015 May 28;10(4):22. doi: 10.1007/s12263-015-0470-6 (PMC4446272; doi:10.1007/s12263-015-0470-6)
Supplement: Supplementary file 4 — Supplementary material 4 (ZIP 6984 kb) [file 12263_2015_470_MOESM4_ESM.zip › HF LF 12 w GSEA result/INFLAMMATORY_RESPONSE.html]

Details for gene set INFLAMMATORY\_RESPONSE[GSEA]

|  || Dataset | HF LF 12w\_collapsed |
| Phenotype | NoPhenotypeAvailable |
| Upregulated in class | na\_pos |
| GeneSet | INFLAMMATORY\_RESPONSE |
| Enrichment Score (ES) | 0.5808063 |
| Normalized Enrichment Score (NES) | 2.1313365 |
| Nominal p-value | 0.0 |
| FDR q-value | 0.0011815266 |
| FWER p-Value | 0.012 |
Table: GSEA Results Summary

  

Fig 1: Enrichment plot: INFLAMMATORY\_RESPONSE      
 Profile of the Running ES Score & Positions of GeneSet Members on the Rank Ordered List

  

| PROBE | GENE SYMBOL | GENE\_TITLE | RANK IN GENE LIST | RANK METRIC SCORE | RUNNING ES | CORE ENRICHMENT || 1 | LTB4R |  |  | 14 | 7.889 | 0.0753 | Yes |
| 2 | CCR3 |  |  | 46 | 6.449 | 0.1342 | Yes |
| 3 | C2 |  |  | 103 | 5.276 | 0.1779 | Yes |
| 4 | CCR2 |  |  | 112 | 5.179 | 0.2276 | Yes |
| 5 | ALOX5AP |  |  | 139 | 4.956 | 0.2725 | Yes |
| 6 | MEFV |  |  | 143 | 4.917 | 0.3202 | Yes |
| 7 | LBP |  |  | 176 | 4.677 | 0.3616 | Yes |
| 8 | CCL11 |  |  | 222 | 4.439 | 0.3987 | Yes |
| 9 | CCL24 |  |  | 224 | 4.436 | 0.4420 | Yes |
| 10 | FOS |  |  | 265 | 4.135 | 0.4769 | Yes |
| 11 | C3AR1 |  |  | 443 | 3.340 | 0.4845 | Yes |
| 12 | CCL4 |  |  | 486 | 3.157 | 0.5095 | Yes |
| 13 | CCR5 |  |  | 497 | 3.131 | 0.5388 | Yes |
| 14 | NFATC4 |  |  | 621 | 2.761 | 0.5484 | Yes |
| 15 | AOC3 |  |  | 641 | 2.718 | 0.5724 | Yes |
| 16 | CCL5 |  |  | 752 | 2.451 | 0.5808 | Yes |
| 17 | TNFRSF1A |  |  | 1146 | 1.835 | 0.5431 | No |
| 18 | AIF1 |  |  | 1336 | 1.603 | 0.5320 | No |
| 19 | S100A8 |  |  | 1340 | 1.600 | 0.5472 | No |
| 20 | CXCL9 |  |  | 1395 | 1.543 | 0.5547 | No |
| 21 | HDAC4 |  |  | 1596 | 1.330 | 0.5394 | No |
| 22 | PARP4 |  |  | 1628 | 1.290 | 0.5476 | No |
| 23 | ABCF1 |  |  | 1787 | 1.105 | 0.5361 | No |
| 24 | TGFB1 |  |  | 2164 | 0.718 | 0.4898 | No |
| 25 | RAC1 |  |  | 2194 | 0.686 | 0.4924 | No |
| 26 | AHSG |  |  | 2834 | 0.131 | 0.4031 | No |
| 27 | PLA2G2D |  |  | 2954 | 0.044 | 0.3867 | No |
| 28 | NFATC3 |  |  | 3127 | -0.085 | 0.3631 | No |
| 29 | NFRKB |  |  | 3325 | -0.216 | 0.3373 | No |
| 30 | F11R |  |  | 4020 | -0.711 | 0.2459 | No |
| 31 | CXCR4 |  |  | 4118 | -0.780 | 0.2397 | No |
| 32 | CX3CL1 |  |  | 4357 | -0.956 | 0.2154 | No |
| 33 | NFX1 |  |  | 5027 | -1.459 | 0.1348 | No |
| 34 | ELF3 |  |  | 5164 | -1.567 | 0.1309 | No |
| 35 | AOX1 |  |  | 5181 | -1.579 | 0.1441 | No |
| 36 | ALOX15 |  |  | 6081 | -2.614 | 0.0423 | No |
| 37 | ORM1 |  |  | 6398 | -3.186 | 0.0287 | No |
| 38 | ORM2 |  |  | 6469 | -3.363 | 0.0517 | No |
| 39 | CDO1 |  |  | 6608 | -3.703 | 0.0685 | No |
Table: GSEA details [plain text format]

  

Fig 2: INFLAMMATORY\_RESPONSE: Random ES distribution      
 Gene set null distribution of ES for **INFLAMMATORY\_RESPONSE**

  
