# Supplementary material for: Network-based integration of molecular and physiological data elucidates regulatory mechanisms underlying adaptation to high-fat diet
Source: Genes Nutr. 2015 May 28;10(4):22. doi: 10.1007/s12263-015-0470-6 (PMC4446272; doi:10.1007/s12263-015-0470-6)
Supplement: Supplementary file 4 — Supplementary material 4 (ZIP 6984 kb) [file 12263_2015_470_MOESM4_ESM.zip › HF LF 12 w GSEA result/LYASE_ACTIVITY.html]

Details for gene set LYASE\_ACTIVITY[GSEA]

|  || Dataset | HF LF 12w\_collapsed |
| Phenotype | NoPhenotypeAvailable |
| Upregulated in class | na\_neg |
| GeneSet | LYASE\_ACTIVITY |
| Enrichment Score (ES) | -0.6158476 |
| Normalized Enrichment Score (NES) | -1.9097273 |
| Nominal p-value | 0.0016447369 |
| FDR q-value | 0.004458257 |
| FWER p-Value | 0.111 |
Table: GSEA Results Summary

  

Fig 1: Enrichment plot: LYASE\_ACTIVITY      
 Profile of the Running ES Score & Positions of GeneSet Members on the Rank Ordered List

  

| PROBE | GENE SYMBOL | GENE\_TITLE | RANK IN GENE LIST | RANK METRIC SCORE | RUNNING ES | CORE ENRICHMENT || 1 | ADCY7 |  |  | 219 | 4.459 | 0.0348 | No |
| 2 | GUCY2C |  |  | 1329 | 1.615 | -0.0983 | No |
| 3 | ODC1 |  |  | 1908 | 0.951 | -0.1661 | No |
| 4 | ECH1 |  |  | 2274 | 0.620 | -0.2086 | No |
| 5 | CRYM |  |  | 2441 | 0.458 | -0.2253 | No |
| 6 | GGCX |  |  | 2901 | 0.079 | -0.2891 | No |
| 7 | GUCY1B3 |  |  | 3486 | -0.337 | -0.3668 | No |
| 8 | UMPS |  |  | 3573 | -0.389 | -0.3732 | No |
| 9 | OGG1 |  |  | 3805 | -0.557 | -0.3977 | No |
| 10 | ADCY8 |  |  | 3859 | -0.597 | -0.3964 | No |
| 11 | HDC |  |  | 3948 | -0.676 | -0.3989 | No |
| 12 | ASL |  |  | 4141 | -0.795 | -0.4143 | No |
| 13 | ALDOB |  |  | 5566 | -1.966 | -0.5868 | Yes |
| 14 | NTHL1 |  |  | 5771 | -2.231 | -0.5828 | Yes |
| 15 | APEX1 |  |  | 5813 | -2.288 | -0.5548 | Yes |
| 16 | ADSL |  |  | 6018 | -2.535 | -0.5462 | Yes |
| 17 | GLO1 |  |  | 6178 | -2.771 | -0.5278 | Yes |
| 18 | MLYCD |  |  | 6447 | -3.298 | -0.5171 | Yes |
| 19 | UROD |  |  | 6474 | -3.377 | -0.4709 | Yes |
| 20 | SCLY |  |  | 6709 | -4.031 | -0.4445 | Yes |
| 21 | EHHADH |  |  | 6728 | -4.077 | -0.3868 | Yes |
| 22 | HADHA |  |  | 6795 | -4.417 | -0.3309 | Yes |
| 23 | PTS |  |  | 6849 | -4.692 | -0.2692 | Yes |
| 24 | AUH |  |  | 6873 | -4.832 | -0.2011 | Yes |
| 25 | BCKDHA |  |  | 6876 | -4.864 | -0.1295 | Yes |
| 26 | ECHS1 |  |  | 6909 | -5.097 | -0.0588 | Yes |
| 27 | MVD |  |  | 6966 | -5.718 | 0.0177 | Yes |
Table: GSEA details [plain text format]

  

Fig 2: LYASE\_ACTIVITY: Random ES distribution      
 Gene set null distribution of ES for **LYASE\_ACTIVITY**

  
