# Supplementary material for: Network-based integration of molecular and physiological data elucidates regulatory mechanisms underlying adaptation to high-fat diet
Source: Genes Nutr. 2015 May 28;10(4):22. doi: 10.1007/s12263-015-0470-6 (PMC4446272; doi:10.1007/s12263-015-0470-6)
Supplement: Supplementary file 4 — Supplementary material 4 (ZIP 6984 kb) [file 12263_2015_470_MOESM4_ESM.zip › HF LF 12 w GSEA result/MICROBODY.html]

Details for gene set MICROBODY[GSEA]

|  || Dataset | HF LF 12w\_collapsed |
| Phenotype | NoPhenotypeAvailable |
| Upregulated in class | na\_neg |
| GeneSet | MICROBODY |
| Enrichment Score (ES) | -0.7289712 |
| Normalized Enrichment Score (NES) | -2.2674127 |
| Nominal p-value | 0.0 |
| FDR q-value | 0.0 |
| FWER p-Value | 0.0 |
Table: GSEA Results Summary

  

Fig 1: Enrichment plot: MICROBODY      
 Profile of the Running ES Score & Positions of GeneSet Members on the Rank Ordered List

  

| PROBE | GENE SYMBOL | GENE\_TITLE | RANK IN GENE LIST | RANK METRIC SCORE | RUNNING ES | CORE ENRICHMENT || 1 | ACOX3 |  |  | 2093 | 0.768 | -0.2856 | No |
| 2 | ABCD2 |  |  | 2117 | 0.753 | -0.2786 | No |
| 3 | ECH1 |  |  | 2274 | 0.620 | -0.2921 | No |
| 4 | ISOC1 |  |  | 2677 | 0.257 | -0.3455 | No |
| 5 | HSD17B4 |  |  | 3449 | -0.310 | -0.4503 | No |
| 6 | PEX6 |  |  | 3552 | -0.376 | -0.4596 | No |
| 7 | PIPOX |  |  | 3886 | -0.623 | -0.4982 | No |
| 8 | SLC25A17 |  |  | 5518 | -1.916 | -0.7027 | Yes |
| 9 | PXMP4 |  |  | 5631 | -2.043 | -0.6905 | Yes |
| 10 | GNPAT |  |  | 5672 | -2.100 | -0.6673 | Yes |
| 11 | PHYH |  |  | 5957 | -2.475 | -0.6735 | Yes |
| 12 | SCP2 |  |  | 6160 | -2.729 | -0.6647 | Yes |
| 13 | PEX19 |  |  | 6277 | -2.948 | -0.6406 | Yes |
| 14 | PEX12 |  |  | 6399 | -3.189 | -0.6140 | Yes |
| 15 | MLYCD |  |  | 6447 | -3.298 | -0.5754 | Yes |
| 16 | PEX16 |  |  | 6545 | -3.534 | -0.5406 | Yes |
| 17 | CROT |  |  | 6565 | -3.576 | -0.4942 | Yes |
| 18 | PEX7 |  |  | 6606 | -3.701 | -0.4491 | Yes |
| 19 | PEX14 |  |  | 6609 | -3.704 | -0.3985 | Yes |
| 20 | ABCD3 |  |  | 6685 | -3.957 | -0.3548 | Yes |
| 21 | EHHADH |  |  | 6728 | -4.077 | -0.3048 | Yes |
| 22 | IDE |  |  | 6976 | -5.813 | -0.2600 | Yes |
| 23 | AMACR |  |  | 6984 | -6.063 | -0.1778 | Yes |
| 24 | PEX11A |  |  | 7011 | -6.380 | -0.0939 | Yes |
| 25 | PMVK |  |  | 7056 | -7.654 | 0.0050 | Yes |
Table: GSEA details [plain text format]

  

Fig 2: MICROBODY: Random ES distribution      
 Gene set null distribution of ES for **MICROBODY**

  
