# Supplementary material for: Network-based integration of molecular and physiological data elucidates regulatory mechanisms underlying adaptation to high-fat diet
Source: Genes Nutr. 2015 May 28;10(4):22. doi: 10.1007/s12263-015-0470-6 (PMC4446272; doi:10.1007/s12263-015-0470-6)
Supplement: Supplementary file 4 — Supplementary material 4 (ZIP 6984 kb) [file 12263_2015_470_MOESM4_ESM.zip › HF LF 12 w GSEA result/MITOCHONDRIAL_ENVELOPE.html]

Details for gene set MITOCHONDRIAL\_ENVELOPE[GSEA]

|  || Dataset | HF LF 12w\_collapsed |
| Phenotype | NoPhenotypeAvailable |
| Upregulated in class | na\_neg |
| GeneSet | MITOCHONDRIAL\_ENVELOPE |
| Enrichment Score (ES) | -0.65270084 |
| Normalized Enrichment Score (NES) | -2.2831223 |
| Nominal p-value | 0.0 |
| FDR q-value | 0.0 |
| FWER p-Value | 0.0 |
Table: GSEA Results Summary

  

Fig 1: Enrichment plot: MITOCHONDRIAL\_ENVELOPE      
 Profile of the Running ES Score & Positions of GeneSet Members on the Rank Ordered List

  

| PROBE | GENE SYMBOL | GENE\_TITLE | RANK IN GENE LIST | RANK METRIC SCORE | RUNNING ES | CORE ENRICHMENT || 1 | MCL1 |  |  | 214 | 4.478 | 0.0011 | No |
| 2 | RAB11FIP5 |  |  | 339 | 3.759 | 0.0100 | No |
| 3 | GATM |  |  | 808 | 2.348 | -0.0399 | No |
| 4 | COX6B2 |  |  | 1180 | 1.785 | -0.0800 | No |
| 5 | BCL2 |  |  | 1486 | 1.441 | -0.1131 | No |
| 6 | OXA1L |  |  | 3411 | -0.282 | -0.3841 | No |
| 7 | UCP3 |  |  | 3585 | -0.399 | -0.4058 | No |
| 8 | CASP7 |  |  | 3823 | -0.573 | -0.4354 | No |
| 9 | MPV17 |  |  | 3962 | -0.683 | -0.4502 | No |
| 10 | ALAS2 |  |  | 4053 | -0.741 | -0.4578 | No |
| 11 | ABCB6 |  |  | 4326 | -0.930 | -0.4898 | No |
| 12 | MFN2 |  |  | 4803 | -1.272 | -0.5484 | No |
| 13 | PPOX |  |  | 5159 | -1.558 | -0.5878 | No |
| 14 | ABCF2 |  |  | 5491 | -1.892 | -0.6215 | No |
| 15 | ABCB7 |  |  | 5622 | -2.034 | -0.6256 | No |
| 16 | TIMM17A |  |  | 5814 | -2.288 | -0.6366 | Yes |
| 17 | PHB |  |  | 5891 | -2.384 | -0.6306 | Yes |
| 18 | NDUFA2 |  |  | 5941 | -2.459 | -0.6203 | Yes |
| 19 | ATP5E |  |  | 6002 | -2.522 | -0.6110 | Yes |
| 20 | MAOB |  |  | 6130 | -2.689 | -0.6101 | Yes |
| 21 | RHOT2 |  |  | 6381 | -3.140 | -0.6235 | Yes |
| 22 | TIMM50 |  |  | 6437 | -3.271 | -0.6083 | Yes |
| 23 | MRPL32 |  |  | 6491 | -3.417 | -0.5918 | Yes |
| 24 | MTX2 |  |  | 6514 | -3.473 | -0.5705 | Yes |
| 25 | NDUFS2 |  |  | 6521 | -3.486 | -0.5468 | Yes |
| 26 | TIMM9 |  |  | 6556 | -3.553 | -0.5266 | Yes |
| 27 | SURF1 |  |  | 6573 | -3.596 | -0.5036 | Yes |
| 28 | SLC25A11 |  |  | 6582 | -3.619 | -0.4793 | Yes |
| 29 | UQCRC1 |  |  | 6607 | -3.702 | -0.4566 | Yes |
| 30 | TIMM8B |  |  | 6651 | -3.835 | -0.4357 | Yes |
| 31 | CYCS |  |  | 6766 | -4.280 | -0.4218 | Yes |
| 32 | NDUFA9 |  |  | 6784 | -4.356 | -0.3936 | Yes |
| 33 | ACN9 |  |  | 6793 | -4.400 | -0.3638 | Yes |
| 34 | COX15 |  |  | 6801 | -4.443 | -0.3335 | Yes |
| 35 | PMPCA |  |  | 6817 | -4.514 | -0.3039 | Yes |
| 36 | SDHD |  |  | 6818 | -4.523 | -0.2720 | Yes |
| 37 | OPA1 |  |  | 6841 | -4.614 | -0.2427 | Yes |
| 38 | ATP5B |  |  | 6904 | -5.072 | -0.2158 | Yes |
| 39 | NDUFS4 |  |  | 6939 | -5.391 | -0.1827 | Yes |
| 40 | NDUFA1 |  |  | 6941 | -5.397 | -0.1449 | Yes |
| 41 | NDUFAB1 |  |  | 6957 | -5.581 | -0.1077 | Yes |
| 42 | TIMM10 |  |  | 6965 | -5.684 | -0.0687 | Yes |
| 43 | NDUFA6 |  |  | 6987 | -6.084 | -0.0289 | Yes |
| 44 | NDUFS1 |  |  | 6998 | -6.186 | 0.0132 | Yes |
Table: GSEA details [plain text format]

  

Fig 2: MITOCHONDRIAL\_ENVELOPE: Random ES distribution      
 Gene set null distribution of ES for **MITOCHONDRIAL\_ENVELOPE**

  
