# Supplementary material for: Network-based integration of molecular and physiological data elucidates regulatory mechanisms underlying adaptation to high-fat diet
Source: Genes Nutr. 2015 May 28;10(4):22. doi: 10.1007/s12263-015-0470-6 (PMC4446272; doi:10.1007/s12263-015-0470-6)
Supplement: Supplementary file 4 — Supplementary material 4 (ZIP 6984 kb) [file 12263_2015_470_MOESM4_ESM.zip › HF LF 12 w GSEA result/MITOCHONDRIAL_INNER_MEMBRANE.html]

Details for gene set MITOCHONDRIAL\_INNER\_MEMBRANE[GSEA]

|  || Dataset | HF LF 12w\_collapsed |
| Phenotype | NoPhenotypeAvailable |
| Upregulated in class | na\_neg |
| GeneSet | MITOCHONDRIAL\_INNER\_MEMBRANE |
| Enrichment Score (ES) | -0.75278145 |
| Normalized Enrichment Score (NES) | -2.4116995 |
| Nominal p-value | 0.0 |
| FDR q-value | 0.0 |
| FWER p-Value | 0.0 |
Table: GSEA Results Summary

  

Fig 1: Enrichment plot: MITOCHONDRIAL\_INNER\_MEMBRANE      
 Profile of the Running ES Score & Positions of GeneSet Members on the Rank Ordered List

  

| PROBE | GENE SYMBOL | GENE\_TITLE | RANK IN GENE LIST | RANK METRIC SCORE | RUNNING ES | CORE ENRICHMENT || 1 | COX6B2 |  |  | 1180 | 1.785 | -0.1505 | No |
| 2 | OXA1L |  |  | 3411 | -0.282 | -0.4637 | No |
| 3 | UCP3 |  |  | 3585 | -0.399 | -0.4845 | No |
| 4 | MPV17 |  |  | 3962 | -0.683 | -0.5314 | No |
| 5 | ALAS2 |  |  | 4053 | -0.741 | -0.5372 | No |
| 6 | PPOX |  |  | 5159 | -1.558 | -0.6792 | No |
| 7 | ABCB7 |  |  | 5622 | -2.034 | -0.7257 | No |
| 8 | TIMM17A |  |  | 5814 | -2.288 | -0.7315 | Yes |
| 9 | PHB |  |  | 5891 | -2.384 | -0.7201 | Yes |
| 10 | NDUFA2 |  |  | 5941 | -2.459 | -0.7041 | Yes |
| 11 | ATP5E |  |  | 6002 | -2.522 | -0.6891 | Yes |
| 12 | TIMM50 |  |  | 6437 | -3.271 | -0.7202 | Yes |
| 13 | MRPL32 |  |  | 6491 | -3.417 | -0.6959 | Yes |
| 14 | NDUFS2 |  |  | 6521 | -3.486 | -0.6675 | Yes |
| 15 | TIMM9 |  |  | 6556 | -3.553 | -0.6393 | Yes |
| 16 | SURF1 |  |  | 6573 | -3.596 | -0.6081 | Yes |
| 17 | SLC25A11 |  |  | 6582 | -3.619 | -0.5756 | Yes |
| 18 | UQCRC1 |  |  | 6607 | -3.702 | -0.5445 | Yes |
| 19 | TIMM8B |  |  | 6651 | -3.835 | -0.5149 | Yes |
| 20 | NDUFA9 |  |  | 6784 | -4.356 | -0.4931 | Yes |
| 21 | COX15 |  |  | 6801 | -4.443 | -0.4540 | Yes |
| 22 | PMPCA |  |  | 6817 | -4.514 | -0.4141 | Yes |
| 23 | SDHD |  |  | 6818 | -4.523 | -0.3720 | Yes |
| 24 | OPA1 |  |  | 6841 | -4.614 | -0.3322 | Yes |
| 25 | ATP5B |  |  | 6904 | -5.072 | -0.2938 | Yes |
| 26 | NDUFS4 |  |  | 6939 | -5.391 | -0.2484 | Yes |
| 27 | NDUFA1 |  |  | 6941 | -5.397 | -0.1983 | Yes |
| 28 | NDUFAB1 |  |  | 6957 | -5.581 | -0.1485 | Yes |
| 29 | TIMM10 |  |  | 6965 | -5.684 | -0.0966 | Yes |
| 30 | NDUFA6 |  |  | 6987 | -6.084 | -0.0430 | Yes |
| 31 | NDUFS1 |  |  | 6998 | -6.186 | 0.0132 | Yes |
Table: GSEA details [plain text format]

  

Fig 2: MITOCHONDRIAL\_INNER\_MEMBRANE: Random ES distribution      
 Gene set null distribution of ES for **MITOCHONDRIAL\_INNER\_MEMBRANE**

  
