# Supplementary material for: Network-based integration of molecular and physiological data elucidates regulatory mechanisms underlying adaptation to high-fat diet
Source: Genes Nutr. 2015 May 28;10(4):22. doi: 10.1007/s12263-015-0470-6 (PMC4446272; doi:10.1007/s12263-015-0470-6)
Supplement: Supplementary file 4 — Supplementary material 4 (ZIP 6984 kb) [file 12263_2015_470_MOESM4_ESM.zip › HF LF 12 w GSEA result/MITOCHONDRIAL_LUMEN.html]

Details for gene set MITOCHONDRIAL\_LUMEN[GSEA]

|  || Dataset | HF LF 12w\_collapsed |
| Phenotype | NoPhenotypeAvailable |
| Upregulated in class | na\_neg |
| GeneSet | MITOCHONDRIAL\_LUMEN |
| Enrichment Score (ES) | -0.6824324 |
| Normalized Enrichment Score (NES) | -2.0939686 |
| Nominal p-value | 0.0 |
| FDR q-value | 4.2089005E-4 |
| FWER p-Value | 0.008 |
Table: GSEA Results Summary

  

Fig 1: Enrichment plot: MITOCHONDRIAL\_LUMEN      
 Profile of the Running ES Score & Positions of GeneSet Members on the Rank Ordered List

  

| PROBE | GENE SYMBOL | GENE\_TITLE | RANK IN GENE LIST | RANK METRIC SCORE | RUNNING ES | CORE ENRICHMENT || 1 | NR3C1 |  |  | 1631 | 1.286 | -0.2140 | No |
| 2 | NFS1 |  |  | 2605 | 0.314 | -0.3476 | No |
| 3 | MRPS28 |  |  | 3990 | -0.697 | -0.5344 | No |
| 4 | MRPS11 |  |  | 4693 | -1.193 | -0.6182 | No |
| 5 | MRPS18A |  |  | 4919 | -1.365 | -0.6323 | No |
| 6 | DBT |  |  | 4988 | -1.423 | -0.6234 | No |
| 7 | MRPL52 |  |  | 5276 | -1.655 | -0.6425 | No |
| 8 | MRPL10 |  |  | 5559 | -1.956 | -0.6570 | Yes |
| 9 | MRPS12 |  |  | 5572 | -1.969 | -0.6331 | Yes |
| 10 | MRPS22 |  |  | 5791 | -2.251 | -0.6347 | Yes |
| 11 | MRPL51 |  |  | 5910 | -2.421 | -0.6200 | Yes |
| 12 | ACADM |  |  | 6051 | -2.581 | -0.6062 | Yes |
| 13 | SUPV3L1 |  |  | 6127 | -2.687 | -0.5819 | Yes |
| 14 | BCKDK |  |  | 6425 | -3.258 | -0.5816 | Yes |
| 15 | GRPEL1 |  |  | 6487 | -3.405 | -0.5460 | Yes |
| 16 | MRPL12 |  |  | 6785 | -4.359 | -0.5314 | Yes |
| 17 | MRPS24 |  |  | 6789 | -4.377 | -0.4749 | Yes |
| 18 | MRPS15 |  |  | 6798 | -4.430 | -0.4185 | Yes |
| 19 | BCKDHA |  |  | 6876 | -4.864 | -0.3662 | Yes |
| 20 | TIMM44 |  |  | 6877 | -4.881 | -0.3028 | Yes |
| 21 | NDUFAB1 |  |  | 6957 | -5.581 | -0.2414 | Yes |
| 22 | CS |  |  | 6996 | -6.174 | -0.1666 | Yes |
| 23 | ALDH4A1 |  |  | 7025 | -6.784 | -0.0824 | Yes |
| 24 | MRPS35 |  |  | 7036 | -7.049 | 0.0078 | Yes |
Table: GSEA details [plain text format]

  

Fig 2: MITOCHONDRIAL\_LUMEN: Random ES distribution      
 Gene set null distribution of ES for **MITOCHONDRIAL\_LUMEN**

  
