# Supplementary material for: Network-based integration of molecular and physiological data elucidates regulatory mechanisms underlying adaptation to high-fat diet
Source: Genes Nutr. 2015 May 28;10(4):22. doi: 10.1007/s12263-015-0470-6 (PMC4446272; doi:10.1007/s12263-015-0470-6)
Supplement: Supplementary file 4 — Supplementary material 4 (ZIP 6984 kb) [file 12263_2015_470_MOESM4_ESM.zip › HF LF 12 w GSEA result/MITOCHONDRIAL_PART.html]

Details for gene set MITOCHONDRIAL\_PART[GSEA]

|  || Dataset | HF LF 12w\_collapsed |
| Phenotype | NoPhenotypeAvailable |
| Upregulated in class | na\_neg |
| GeneSet | MITOCHONDRIAL\_PART |
| Enrichment Score (ES) | -0.6521616 |
| Normalized Enrichment Score (NES) | -2.469677 |
| Nominal p-value | 0.0 |
| FDR q-value | 0.0 |
| FWER p-Value | 0.0 |
Table: GSEA Results Summary

  

Fig 1: Enrichment plot: MITOCHONDRIAL\_PART      
 Profile of the Running ES Score & Positions of GeneSet Members on the Rank Ordered List

  

| PROBE | GENE SYMBOL | GENE\_TITLE | RANK IN GENE LIST | RANK METRIC SCORE | RUNNING ES | CORE ENRICHMENT || 1 | MCL1 |  |  | 214 | 4.478 | -0.0095 | No |
| 2 | RAB11FIP5 |  |  | 339 | 3.759 | -0.0095 | No |
| 3 | GATM |  |  | 808 | 2.348 | -0.0652 | No |
| 4 | COX6B2 |  |  | 1180 | 1.785 | -0.1096 | No |
| 5 | BCL2 |  |  | 1486 | 1.441 | -0.1463 | No |
| 6 | NR3C1 |  |  | 1631 | 1.286 | -0.1607 | No |
| 7 | NFS1 |  |  | 2605 | 0.314 | -0.2978 | No |
| 8 | OXA1L |  |  | 3411 | -0.282 | -0.4111 | No |
| 9 | UCP3 |  |  | 3585 | -0.399 | -0.4338 | No |
| 10 | CASP7 |  |  | 3823 | -0.573 | -0.4649 | No |
| 11 | MPV17 |  |  | 3962 | -0.683 | -0.4813 | No |
| 12 | MRPS28 |  |  | 3990 | -0.697 | -0.4819 | No |
| 13 | ALAS2 |  |  | 4053 | -0.741 | -0.4872 | No |
| 14 | ABCB6 |  |  | 4326 | -0.930 | -0.5216 | No |
| 15 | MRPS11 |  |  | 4693 | -1.193 | -0.5681 | No |
| 16 | MFN2 |  |  | 4803 | -1.272 | -0.5777 | No |
| 17 | MRPS18A |  |  | 4919 | -1.365 | -0.5877 | No |
| 18 | DBT |  |  | 4988 | -1.423 | -0.5907 | No |
| 19 | PPOX |  |  | 5159 | -1.558 | -0.6076 | No |
| 20 | MRPL52 |  |  | 5276 | -1.655 | -0.6163 | No |
| 21 | ABCF2 |  |  | 5491 | -1.892 | -0.6379 | No |
| 22 | MRPL10 |  |  | 5559 | -1.956 | -0.6383 | No |
| 23 | MRPS12 |  |  | 5572 | -1.969 | -0.6308 | No |
| 24 | ABCB7 |  |  | 5622 | -2.034 | -0.6282 | No |
| 25 | MRPS22 |  |  | 5791 | -2.251 | -0.6416 | Yes |
| 26 | TIMM17A |  |  | 5814 | -2.288 | -0.6340 | Yes |
| 27 | PHB |  |  | 5891 | -2.384 | -0.6337 | Yes |
| 28 | MRPL51 |  |  | 5910 | -2.421 | -0.6249 | Yes |
| 29 | NDUFA2 |  |  | 5941 | -2.459 | -0.6177 | Yes |
| 30 | ATP5E |  |  | 6002 | -2.522 | -0.6144 | Yes |
| 31 | ACADM |  |  | 6051 | -2.581 | -0.6091 | Yes |
| 32 | SUPV3L1 |  |  | 6127 | -2.687 | -0.6072 | Yes |
| 33 | MAOB |  |  | 6130 | -2.689 | -0.5949 | Yes |
| 34 | RHOT2 |  |  | 6381 | -3.140 | -0.6158 | Yes |
| 35 | BCKDK |  |  | 6425 | -3.258 | -0.6067 | Yes |
| 36 | TIMM50 |  |  | 6437 | -3.271 | -0.5929 | Yes |
| 37 | GRPEL1 |  |  | 6487 | -3.405 | -0.5839 | Yes |
| 38 | MRPL32 |  |  | 6491 | -3.417 | -0.5684 | Yes |
| 39 | MTX2 |  |  | 6514 | -3.473 | -0.5552 | Yes |
| 40 | NDUFS2 |  |  | 6521 | -3.486 | -0.5398 | Yes |
| 41 | TIMM9 |  |  | 6556 | -3.553 | -0.5280 | Yes |
| 42 | SURF1 |  |  | 6573 | -3.596 | -0.5134 | Yes |
| 43 | SLC25A11 |  |  | 6582 | -3.619 | -0.4976 | Yes |
| 44 | UQCRC1 |  |  | 6607 | -3.702 | -0.4837 | Yes |
| 45 | TIMM8B |  |  | 6651 | -3.835 | -0.4718 | Yes |
| 46 | CYCS |  |  | 6766 | -4.280 | -0.4680 | Yes |
| 47 | NDUFA9 |  |  | 6784 | -4.356 | -0.4500 | Yes |
| 48 | MRPL12 |  |  | 6785 | -4.359 | -0.4296 | Yes |
| 49 | MRPS24 |  |  | 6789 | -4.377 | -0.4095 | Yes |
| 50 | ACN9 |  |  | 6793 | -4.400 | -0.3893 | Yes |
| 51 | MRPS15 |  |  | 6798 | -4.430 | -0.3692 | Yes |
| 52 | COX15 |  |  | 6801 | -4.443 | -0.3486 | Yes |
| 53 | PMPCA |  |  | 6817 | -4.514 | -0.3296 | Yes |
| 54 | SDHD |  |  | 6818 | -4.523 | -0.3085 | Yes |
| 55 | OPA1 |  |  | 6841 | -4.614 | -0.2900 | Yes |
| 56 | BCKDHA |  |  | 6876 | -4.864 | -0.2720 | Yes |
| 57 | TIMM44 |  |  | 6877 | -4.881 | -0.2492 | Yes |
| 58 | ATP5B |  |  | 6904 | -5.072 | -0.2291 | Yes |
| 59 | NDUFS4 |  |  | 6939 | -5.391 | -0.2087 | Yes |
| 60 | NDUFA1 |  |  | 6941 | -5.397 | -0.1836 | Yes |
| 61 | NDUFAB1 |  |  | 6957 | -5.581 | -0.1596 | Yes |
| 62 | TIMM10 |  |  | 6965 | -5.684 | -0.1340 | Yes |
| 63 | NDUFA6 |  |  | 6987 | -6.084 | -0.1084 | Yes |
| 64 | CS |  |  | 6996 | -6.174 | -0.0807 | Yes |
| 65 | NDUFS1 |  |  | 6998 | -6.186 | -0.0518 | Yes |
| 66 | ALDH4A1 |  |  | 7025 | -6.784 | -0.0238 | Yes |
| 67 | MRPS35 |  |  | 7036 | -7.049 | 0.0078 | Yes |
Table: GSEA details [plain text format]

  

Fig 2: MITOCHONDRIAL\_PART: Random ES distribution      
 Gene set null distribution of ES for **MITOCHONDRIAL\_PART**

  
