# Supplementary material for: Network-based integration of molecular and physiological data elucidates regulatory mechanisms underlying adaptation to high-fat diet
Source: Genes Nutr. 2015 May 28;10(4):22. doi: 10.1007/s12263-015-0470-6 (PMC4446272; doi:10.1007/s12263-015-0470-6)
Supplement: Supplementary file 4 — Supplementary material 4 (ZIP 6984 kb) [file 12263_2015_470_MOESM4_ESM.zip › HF LF 12 w GSEA result/MITOCHONDRION.html]

Details for gene set MITOCHONDRION[GSEA]

|  || Dataset | HF LF 12w\_collapsed |
| Phenotype | NoPhenotypeAvailable |
| Upregulated in class | na\_neg |
| GeneSet | MITOCHONDRION |
| Enrichment Score (ES) | -0.60081005 |
| Normalized Enrichment Score (NES) | -2.5600672 |
| Nominal p-value | 0.0 |
| FDR q-value | 0.0 |
| FWER p-Value | 0.0 |
Table: GSEA Results Summary

  

Fig 1: Enrichment plot: MITOCHONDRION      
 Profile of the Running ES Score & Positions of GeneSet Members on the Rank Ordered List

  

| PROBE | GENE SYMBOL | GENE\_TITLE | RANK IN GENE LIST | RANK METRIC SCORE | RUNNING ES | CORE ENRICHMENT || 1 | PTRF |  |  | 96 | 5.343 | -0.0023 | No |
| 2 | OAT |  |  | 123 | 5.046 | 0.0048 | No |
| 3 | MCL1 |  |  | 214 | 4.478 | 0.0015 | No |
| 4 | ASAH2 |  |  | 277 | 4.093 | 0.0014 | No |
| 5 | PDK3 |  |  | 304 | 3.914 | 0.0061 | No |
| 6 | RAB11FIP5 |  |  | 339 | 3.759 | 0.0093 | No |
| 7 | NME4 |  |  | 373 | 3.606 | 0.0123 | No |
| 8 | CPT1A |  |  | 441 | 3.344 | 0.0099 | No |
| 9 | PMAIP1 |  |  | 636 | 2.727 | -0.0122 | No |
| 10 | GATM |  |  | 808 | 2.348 | -0.0318 | No |
| 11 | COX6B2 |  |  | 1180 | 1.785 | -0.0814 | No |
| 12 | SLC9A6 |  |  | 1303 | 1.641 | -0.0955 | No |
| 13 | BCL2L1 |  |  | 1323 | 1.625 | -0.0947 | No |
| 14 | BCL2 |  |  | 1486 | 1.441 | -0.1149 | No |
| 15 | GPX4 |  |  | 1564 | 1.362 | -0.1231 | No |
| 16 | COX8C |  |  | 1568 | 1.358 | -0.1206 | No |
| 17 | NR3C1 |  |  | 1631 | 1.286 | -0.1268 | No |
| 18 | CASP8 |  |  | 1886 | 0.981 | -0.1613 | No |
| 19 | SLC25A27 |  |  | 2195 | 0.686 | -0.2042 | No |
| 20 | PSEN1 |  |  | 2304 | 0.587 | -0.2185 | No |
| 21 | MSRB3 |  |  | 2421 | 0.482 | -0.2341 | No |
| 22 | NFS1 |  |  | 2605 | 0.314 | -0.2598 | No |
| 23 | MTHFD2 |  |  | 2836 | 0.129 | -0.2927 | No |
| 24 | BID |  |  | 3123 | -0.083 | -0.3337 | No |
| 25 | SLC25A12 |  |  | 3322 | -0.214 | -0.3618 | No |
| 26 | POLG |  |  | 3375 | -0.250 | -0.3688 | No |
| 27 | OXA1L |  |  | 3411 | -0.282 | -0.3732 | No |
| 28 | AASS |  |  | 3412 | -0.282 | -0.3726 | No |
| 29 | DHRS4 |  |  | 3463 | -0.315 | -0.3791 | No |
| 30 | ALDH5A1 |  |  | 3496 | -0.341 | -0.3830 | No |
| 31 | GOT2 |  |  | 3500 | -0.346 | -0.3827 | No |
| 32 | UCP3 |  |  | 3585 | -0.399 | -0.3939 | No |
| 33 | AGPAT5 |  |  | 3688 | -0.476 | -0.4076 | No |
| 34 | CASP7 |  |  | 3823 | -0.573 | -0.4257 | No |
| 35 | ATPIF1 |  |  | 3838 | -0.586 | -0.4264 | No |
| 36 | DUT |  |  | 3857 | -0.596 | -0.4277 | No |
| 37 | MPV17 |  |  | 3962 | -0.683 | -0.4413 | No |
| 38 | MRPS28 |  |  | 3990 | -0.697 | -0.4436 | No |
| 39 | ALAS2 |  |  | 4053 | -0.741 | -0.4510 | No |
| 40 | SHMT2 |  |  | 4079 | -0.756 | -0.4530 | No |
| 41 | SLC25A13 |  |  | 4127 | -0.786 | -0.4580 | No |
| 42 | ABCB6 |  |  | 4326 | -0.930 | -0.4846 | No |
| 43 | BCL2L10 |  |  | 4404 | -0.992 | -0.4935 | No |
| 44 | MRPS11 |  |  | 4693 | -1.193 | -0.5325 | No |
| 45 | SARS2 |  |  | 4730 | -1.214 | -0.5350 | No |
| 46 | GSTZ1 |  |  | 4765 | -1.242 | -0.5373 | No |
| 47 | PDK4 |  |  | 4798 | -1.269 | -0.5391 | No |
| 48 | MFN2 |  |  | 4803 | -1.272 | -0.5370 | No |
| 49 | ABCE1 |  |  | 4826 | -1.290 | -0.5374 | No |
| 50 | MRPS18A |  |  | 4919 | -1.365 | -0.5477 | No |
| 51 | TBRG4 |  |  | 4923 | -1.367 | -0.5452 | No |
| 52 | DBT |  |  | 4988 | -1.423 | -0.5513 | No |
| 53 | CPOX |  |  | 5024 | -1.458 | -0.5532 | No |
| 54 | PPOX |  |  | 5159 | -1.558 | -0.5692 | No |
| 55 | AK3 |  |  | 5170 | -1.570 | -0.5672 | No |
| 56 | GHITM |  |  | 5212 | -1.607 | -0.5697 | No |
| 57 | MRPL52 |  |  | 5276 | -1.655 | -0.5752 | No |
| 58 | FIBP |  |  | 5309 | -1.690 | -0.5761 | No |
| 59 | TRNT1 |  |  | 5372 | -1.758 | -0.5813 | No |
| 60 | DECR1 |  |  | 5429 | -1.824 | -0.5854 | No |
| 61 | ACADS |  |  | 5464 | -1.864 | -0.5863 | No |
| 62 | ABCF2 |  |  | 5491 | -1.892 | -0.5860 | No |
| 63 | DIABLO |  |  | 5502 | -1.903 | -0.5833 | No |
| 64 | SLC22A4 |  |  | 5555 | -1.951 | -0.5866 | No |
| 65 | MRPL10 |  |  | 5559 | -1.956 | -0.5828 | No |
| 66 | MRPS12 |  |  | 5572 | -1.969 | -0.5803 | No |
| 67 | ABCB7 |  |  | 5622 | -2.034 | -0.5830 | No |
| 68 | COQ4 |  |  | 5665 | -2.090 | -0.5845 | No |
| 69 | NAPG |  |  | 5779 | -2.236 | -0.5960 | Yes |
| 70 | MRPS22 |  |  | 5791 | -2.251 | -0.5927 | Yes |
| 71 | TIMM17A |  |  | 5814 | -2.288 | -0.5910 | Yes |
| 72 | COX7A1 |  |  | 5855 | -2.332 | -0.5917 | Yes |
| 73 | PHB |  |  | 5891 | -2.384 | -0.5916 | Yes |
| 74 | MRPL51 |  |  | 5910 | -2.421 | -0.5890 | Yes |
| 75 | NT5M |  |  | 5931 | -2.445 | -0.5866 | Yes |
| 76 | NDUFA2 |  |  | 5941 | -2.459 | -0.5826 | Yes |
| 77 | ATP5E |  |  | 6002 | -2.522 | -0.5858 | Yes |
| 78 | ACADM |  |  | 6051 | -2.581 | -0.5871 | Yes |
| 79 | ACAT1 |  |  | 6071 | -2.597 | -0.5843 | Yes |
| 80 | HINT2 |  |  | 6100 | -2.641 | -0.5826 | Yes |
| 81 | SLC25A4 |  |  | 6108 | -2.654 | -0.5779 | Yes |
| 82 | MTRF1 |  |  | 6119 | -2.670 | -0.5736 | Yes |
| 83 | SUPV3L1 |  |  | 6127 | -2.687 | -0.5688 | Yes |
| 84 | MAOB |  |  | 6130 | -2.689 | -0.5633 | Yes |
| 85 | ACADSB |  |  | 6233 | -2.869 | -0.5718 | Yes |
| 86 | ARG2 |  |  | 6281 | -2.952 | -0.5722 | Yes |
| 87 | FDXR |  |  | 6304 | -2.999 | -0.5689 | Yes |
| 88 | RHOT2 |  |  | 6381 | -3.140 | -0.5731 | Yes |
| 89 | SOD2 |  |  | 6412 | -3.219 | -0.5705 | Yes |
| 90 | BCKDK |  |  | 6425 | -3.258 | -0.5652 | Yes |
| 91 | TIMM50 |  |  | 6437 | -3.271 | -0.5597 | Yes |
| 92 | TSFM |  |  | 6463 | -3.338 | -0.5561 | Yes |
| 93 | GRPEL1 |  |  | 6487 | -3.405 | -0.5521 | Yes |
| 94 | MRPL32 |  |  | 6491 | -3.417 | -0.5452 | Yes |
| 95 | MTX2 |  |  | 6514 | -3.473 | -0.5409 | Yes |
| 96 | NDUFS2 |  |  | 6521 | -3.486 | -0.5342 | Yes |
| 97 | SUCLG1 |  |  | 6525 | -3.497 | -0.5271 | Yes |
| 98 | SCO1 |  |  | 6542 | -3.528 | -0.5218 | Yes |
| 99 | TFAM |  |  | 6543 | -3.529 | -0.5142 | Yes |
| 100 | NDUFA8 |  |  | 6547 | -3.536 | -0.5070 | Yes |
| 101 | GBAS |  |  | 6549 | -3.542 | -0.4995 | Yes |
| 102 | TIMM9 |  |  | 6556 | -3.553 | -0.4927 | Yes |
| 103 | SUCLA2 |  |  | 6566 | -3.576 | -0.4863 | Yes |
| 104 | SURF1 |  |  | 6573 | -3.596 | -0.4794 | Yes |
| 105 | SLC25A11 |  |  | 6582 | -3.619 | -0.4728 | Yes |
| 106 | UQCRC1 |  |  | 6607 | -3.702 | -0.4683 | Yes |
| 107 | TIMM8B |  |  | 6651 | -3.835 | -0.4662 | Yes |
| 108 | NDUFA11 |  |  | 6656 | -3.848 | -0.4585 | Yes |
| 109 | COX11 |  |  | 6659 | -3.864 | -0.4504 | Yes |
| 110 | PCCB |  |  | 6695 | -3.996 | -0.4469 | Yes |
| 111 | MRPS31 |  |  | 6719 | -4.041 | -0.4415 | Yes |
| 112 | MTHFD1 |  |  | 6729 | -4.081 | -0.4340 | Yes |
| 113 | CLPX |  |  | 6737 | -4.122 | -0.4261 | Yes |
| 114 | CYCS |  |  | 6766 | -4.280 | -0.4209 | Yes |
| 115 | IDH3B |  |  | 6776 | -4.331 | -0.4128 | Yes |
| 116 | NDUFA9 |  |  | 6784 | -4.356 | -0.4045 | Yes |
| 117 | MRPL12 |  |  | 6785 | -4.359 | -0.3951 | Yes |
| 118 | MRPS24 |  |  | 6789 | -4.377 | -0.3861 | Yes |
| 119 | ACN9 |  |  | 6793 | -4.400 | -0.3770 | Yes |
| 120 | MRPS15 |  |  | 6798 | -4.430 | -0.3680 | Yes |
| 121 | COX15 |  |  | 6801 | -4.443 | -0.3587 | Yes |
| 122 | PMPCA |  |  | 6817 | -4.514 | -0.3512 | Yes |
| 123 | SDHD |  |  | 6818 | -4.523 | -0.3414 | Yes |
| 124 | MSRB2 |  |  | 6829 | -4.558 | -0.3330 | Yes |
| 125 | OPA1 |  |  | 6841 | -4.614 | -0.3247 | Yes |
| 126 | PTS |  |  | 6849 | -4.692 | -0.3156 | Yes |
| 127 | BCKDHA |  |  | 6876 | -4.864 | -0.3088 | Yes |
| 128 | TIMM44 |  |  | 6877 | -4.881 | -0.2983 | Yes |
| 129 | CYC1 |  |  | 6879 | -4.925 | -0.2878 | Yes |
| 130 | COQ7 |  |  | 6896 | -5.009 | -0.2793 | Yes |
| 131 | ATP5B |  |  | 6904 | -5.072 | -0.2694 | Yes |
| 132 | ECHS1 |  |  | 6909 | -5.097 | -0.2590 | Yes |
| 133 | BBC3 |  |  | 6920 | -5.173 | -0.2493 | Yes |
| 134 | NDUFA4 |  |  | 6922 | -5.195 | -0.2382 | Yes |
| 135 | DLD |  |  | 6927 | -5.255 | -0.2275 | Yes |
| 136 | PTGES2 |  |  | 6933 | -5.319 | -0.2167 | Yes |
| 137 | NDUFS4 |  |  | 6939 | -5.391 | -0.2058 | Yes |
| 138 | NDUFA1 |  |  | 6941 | -5.397 | -0.1943 | Yes |
| 139 | POLRMT |  |  | 6954 | -5.547 | -0.1841 | Yes |
| 140 | NDUFAB1 |  |  | 6957 | -5.581 | -0.1724 | Yes |
| 141 | TIMM10 |  |  | 6965 | -5.684 | -0.1611 | Yes |
| 142 | ACO2 |  |  | 6981 | -5.918 | -0.1505 | Yes |
| 143 | BAK1 |  |  | 6983 | -6.041 | -0.1376 | Yes |
| 144 | AMACR |  |  | 6984 | -6.063 | -0.1246 | Yes |
| 145 | NDUFA6 |  |  | 6987 | -6.084 | -0.1117 | Yes |
| 146 | BPHL |  |  | 6990 | -6.135 | -0.0988 | Yes |
| 147 | CS |  |  | 6996 | -6.174 | -0.0862 | Yes |
| 148 | NDUFS1 |  |  | 6998 | -6.186 | -0.0730 | Yes |
| 149 | ALDH4A1 |  |  | 7025 | -6.784 | -0.0621 | Yes |
| 150 | ACP6 |  |  | 7033 | -7.001 | -0.0481 | Yes |
| 151 | MRPS35 |  |  | 7036 | -7.049 | -0.0332 | Yes |
| 152 | PDHA1 |  |  | 7065 | -7.949 | -0.0201 | Yes |
| 153 | PDK1 |  |  | 7085 | -10.972 | 0.0009 | Yes |
Table: GSEA details [plain text format]

  

Fig 2: MITOCHONDRION: Random ES distribution      
 Gene set null distribution of ES for **MITOCHONDRION**

  
