# Supplementary material for: Network-based integration of molecular and physiological data elucidates regulatory mechanisms underlying adaptation to high-fat diet
Source: Genes Nutr. 2015 May 28;10(4):22. doi: 10.1007/s12263-015-0470-6 (PMC4446272; doi:10.1007/s12263-015-0470-6)
Supplement: Supplementary file 4 — Supplementary material 4 (ZIP 6984 kb) [file 12263_2015_470_MOESM4_ESM.zip › HF LF 12 w GSEA result/ORGANIC_ACID_METABOLIC_PROCESS.html]

Details for gene set ORGANIC\_ACID\_METABOLIC\_PROCESS[GSEA]

|  || Dataset | HF LF 12w\_collapsed |
| Phenotype | NoPhenotypeAvailable |
| Upregulated in class | na\_neg |
| GeneSet | ORGANIC\_ACID\_METABOLIC\_PROCESS |
| Enrichment Score (ES) | -0.5030879 |
| Normalized Enrichment Score (NES) | -1.8902546 |
| Nominal p-value | 0.0 |
| FDR q-value | 0.005639903 |
| FWER p-Value | 0.147 |
Table: GSEA Results Summary

  

Fig 1: Enrichment plot: ORGANIC\_ACID\_METABOLIC\_PROCESS      
 Profile of the Running ES Score & Positions of GeneSet Members on the Rank Ordered List

  

| PROBE | GENE SYMBOL | GENE\_TITLE | RANK IN GENE LIST | RANK METRIC SCORE | RUNNING ES | CORE ENRICHMENT || 1 | SLC7A8 |  |  | 98 | 5.309 | 0.0187 | No |
| 2 | DDAH2 |  |  | 150 | 4.886 | 0.0415 | No |
| 3 | GSS |  |  | 432 | 3.406 | 0.0225 | No |
| 4 | CPT1A |  |  | 441 | 3.344 | 0.0419 | No |
| 5 | GATM |  |  | 808 | 2.348 | 0.0043 | No |
| 6 | SLC7A7 |  |  | 880 | 2.226 | 0.0079 | No |
| 7 | ARG1 |  |  | 1000 | 2.043 | 0.0035 | No |
| 8 | TNFRSF1A |  |  | 1146 | 1.835 | -0.0058 | No |
| 9 | MTHFR |  |  | 1434 | 1.498 | -0.0375 | No |
| 10 | SLC6A6 |  |  | 1974 | 0.886 | -0.1087 | No |
| 11 | ACOX3 |  |  | 2093 | 0.768 | -0.1208 | No |
| 12 | ABCD2 |  |  | 2117 | 0.753 | -0.1194 | No |
| 13 | ECH1 |  |  | 2274 | 0.620 | -0.1378 | No |
| 14 | PLOD1 |  |  | 2295 | 0.596 | -0.1370 | No |
| 15 | DEGS1 |  |  | 2370 | 0.530 | -0.1443 | No |
| 16 | NFS1 |  |  | 2605 | 0.314 | -0.1756 | No |
| 17 | ALDH18A1 |  |  | 2644 | 0.283 | -0.1793 | No |
| 18 | DDAH1 |  |  | 2817 | 0.147 | -0.2029 | No |
| 19 | MTHFD2 |  |  | 2836 | 0.129 | -0.2046 | No |
| 20 | ADIPOR2 |  |  | 2880 | 0.098 | -0.2101 | No |
| 21 | ALDH5A1 |  |  | 3496 | -0.341 | -0.2956 | No |
| 22 | GOT2 |  |  | 3500 | -0.346 | -0.2939 | No |
| 23 | FAH |  |  | 3555 | -0.377 | -0.2992 | No |
| 24 | DARS |  |  | 3600 | -0.411 | -0.3030 | No |
| 25 | ADIPOR1 |  |  | 3940 | -0.666 | -0.3471 | No |
| 26 | HDC |  |  | 3948 | -0.676 | -0.3439 | No |
| 27 | ASL |  |  | 4141 | -0.795 | -0.3664 | No |
| 28 | AS3MT |  |  | 4297 | -0.911 | -0.3828 | No |
| 29 | NPC1 |  |  | 4314 | -0.922 | -0.3794 | No |
| 30 | PTGS1 |  |  | 4502 | -1.067 | -0.3995 | No |
| 31 | ARPP19 |  |  | 4505 | -1.068 | -0.3932 | No |
| 32 | RARS |  |  | 4533 | -1.089 | -0.3903 | No |
| 33 | SARS2 |  |  | 4730 | -1.214 | -0.4108 | No |
| 34 | GSTZ1 |  |  | 4765 | -1.242 | -0.4080 | No |
| 35 | GGT1 |  |  | 4815 | -1.286 | -0.4070 | No |
| 36 | BBOX1 |  |  | 4972 | -1.409 | -0.4206 | No |
| 37 | HPGD |  |  | 5013 | -1.442 | -0.4174 | No |
| 38 | ALOX12 |  |  | 5178 | -1.576 | -0.4310 | No |
| 39 | ALDH6A1 |  |  | 5237 | -1.625 | -0.4293 | No |
| 40 | ACADS |  |  | 5464 | -1.864 | -0.4500 | No |
| 41 | GNPAT |  |  | 5672 | -2.100 | -0.4665 | No |
| 42 | SLC7A6 |  |  | 5708 | -2.164 | -0.4582 | No |
| 43 | CYP7B1 |  |  | 5915 | -2.422 | -0.4726 | No |
| 44 | ACADM |  |  | 6051 | -2.581 | -0.4759 | No |
| 45 | ACADSB |  |  | 6233 | -2.869 | -0.4840 | Yes |
| 46 | AARS |  |  | 6368 | -3.119 | -0.4839 | Yes |
| 47 | BCKDK |  |  | 6425 | -3.258 | -0.4718 | Yes |
| 48 | MLYCD |  |  | 6447 | -3.298 | -0.4545 | Yes |
| 49 | CROT |  |  | 6565 | -3.576 | -0.4492 | Yes |
| 50 | IDH1 |  |  | 6580 | -3.613 | -0.4289 | Yes |
| 51 | CDO1 |  |  | 6608 | -3.703 | -0.4100 | Yes |
| 52 | SCLY |  |  | 6709 | -4.031 | -0.3994 | Yes |
| 53 | IDH3B |  |  | 6776 | -4.331 | -0.3822 | Yes |
| 54 | ACN9 |  |  | 6793 | -4.400 | -0.3574 | Yes |
| 55 | ASPA |  |  | 6847 | -4.658 | -0.3362 | Yes |
| 56 | PTS |  |  | 6849 | -4.692 | -0.3075 | Yes |
| 57 | BCKDHA |  |  | 6876 | -4.864 | -0.2813 | Yes |
| 58 | GCLM |  |  | 6894 | -5.004 | -0.2529 | Yes |
| 59 | PPARGC1A |  |  | 6906 | -5.088 | -0.2232 | Yes |
| 60 | ECHS1 |  |  | 6909 | -5.097 | -0.1921 | Yes |
| 61 | TST |  |  | 6940 | -5.392 | -0.1632 | Yes |
| 62 | GCLC |  |  | 6977 | -5.860 | -0.1323 | Yes |
| 63 | ACO2 |  |  | 6981 | -5.918 | -0.0963 | Yes |
| 64 | ALDH4A1 |  |  | 7025 | -6.784 | -0.0607 | Yes |
| 65 | FADS1 |  |  | 7086 | -11.367 | 0.0007 | Yes |
Table: GSEA details [plain text format]

  

Fig 2: ORGANIC\_ACID\_METABOLIC\_PROCESS: Random ES distribution      
 Gene set null distribution of ES for **ORGANIC\_ACID\_METABOLIC\_PROCESS**

  
