# Supplementary material for: Network-based integration of molecular and physiological data elucidates regulatory mechanisms underlying adaptation to high-fat diet
Source: Genes Nutr. 2015 May 28;10(4):22. doi: 10.1007/s12263-015-0470-6 (PMC4446272; doi:10.1007/s12263-015-0470-6)
Supplement: Supplementary file 4 — Supplementary material 4 (ZIP 6984 kb) [file 12263_2015_470_MOESM4_ESM.zip › HF LF 12 w GSEA result/OXIDOREDUCTASE_ACTIVITY.html]

Details for gene set OXIDOREDUCTASE\_ACTIVITY[GSEA]

|  || Dataset | HF LF 12w\_collapsed |
| Phenotype | NoPhenotypeAvailable |
| Upregulated in class | na\_neg |
| GeneSet | OXIDOREDUCTASE\_ACTIVITY |
| Enrichment Score (ES) | -0.51081467 |
| Normalized Enrichment Score (NES) | -2.0690353 |
| Nominal p-value | 0.0 |
| FDR q-value | 5.339607E-4 |
| FWER p-Value | 0.011 |
Table: GSEA Results Summary

  

Fig 1: Enrichment plot: OXIDOREDUCTASE\_ACTIVITY      
 Profile of the Running ES Score & Positions of GeneSet Members on the Rank Ordered List

  

| PROBE | GENE SYMBOL | GENE\_TITLE | RANK IN GENE LIST | RANK METRIC SCORE | RUNNING ES | CORE ENRICHMENT || 1 | ALDH1A2 |  |  | 28 | 7.038 | 0.0169 | No |
| 2 | LOXL2 |  |  | 53 | 6.171 | 0.0317 | No |
| 3 | P4HA2 |  |  | 168 | 4.762 | 0.0295 | No |
| 4 | ALOX5 |  |  | 194 | 4.599 | 0.0396 | No |
| 5 | CYBA |  |  | 212 | 4.485 | 0.0504 | No |
| 6 | CYP27A1 |  |  | 362 | 3.653 | 0.0399 | No |
| 7 | HMOX1 |  |  | 438 | 3.366 | 0.0391 | No |
| 8 | P4HB |  |  | 534 | 3.008 | 0.0344 | No |
| 9 | ASPH |  |  | 550 | 2.963 | 0.0411 | No |
| 10 | PDIA5 |  |  | 630 | 2.742 | 0.0379 | No |
| 11 | AOC3 |  |  | 641 | 2.718 | 0.0445 | No |
| 12 | ALDH1A3 |  |  | 814 | 2.334 | 0.0268 | No |
| 13 | FMO2 |  |  | 848 | 2.274 | 0.0288 | No |
| 14 | GPX3 |  |  | 930 | 2.136 | 0.0235 | No |
| 15 | PLOD2 |  |  | 1028 | 2.002 | 0.0156 | No |
| 16 | CYP46A1 |  |  | 1127 | 1.869 | 0.0071 | No |
| 17 | LDHC |  |  | 1195 | 1.765 | 0.0027 | No |
| 18 | ADH4 |  |  | 1365 | 1.574 | -0.0168 | No |
| 19 | MTHFR |  |  | 1434 | 1.498 | -0.0221 | No |
| 20 | CYP17A1 |  |  | 1531 | 1.402 | -0.0317 | No |
| 21 | GPX4 |  |  | 1564 | 1.362 | -0.0323 | No |
| 22 | PLOD3 |  |  | 1862 | 1.011 | -0.0718 | No |
| 23 | VCL |  |  | 2003 | 0.855 | -0.0893 | No |
| 24 | ACOX3 |  |  | 2093 | 0.768 | -0.0998 | No |
| 25 | BLVRA |  |  | 2184 | 0.696 | -0.1106 | No |
| 26 | IMPDH1 |  |  | 2242 | 0.649 | -0.1169 | No |
| 27 | XDH |  |  | 2272 | 0.623 | -0.1192 | No |
| 28 | DEGS1 |  |  | 2370 | 0.530 | -0.1315 | No |
| 29 | MSRB3 |  |  | 2421 | 0.482 | -0.1373 | No |
| 30 | ALDH8A1 |  |  | 2703 | 0.245 | -0.1768 | No |
| 31 | MTHFD2 |  |  | 2836 | 0.129 | -0.1953 | No |
| 32 | ALOX12B |  |  | 3052 | -0.032 | -0.2260 | No |
| 33 | HMOX2 |  |  | 3222 | -0.149 | -0.2498 | No |
| 34 | ALDH1A1 |  |  | 3223 | -0.150 | -0.2494 | No |
| 35 | HSD17B4 |  |  | 3449 | -0.310 | -0.2807 | No |
| 36 | CYP1B1 |  |  | 3469 | -0.319 | -0.2824 | No |
| 37 | MGST3 |  |  | 3478 | -0.329 | -0.2826 | No |
| 38 | ALDH5A1 |  |  | 3496 | -0.341 | -0.2840 | No |
| 39 | DIO2 |  |  | 3634 | -0.440 | -0.3024 | No |
| 40 | IVD |  |  | 3877 | -0.613 | -0.3352 | No |
| 41 | TXNRD1 |  |  | 4158 | -0.806 | -0.3729 | No |
| 42 | NQO1 |  |  | 4161 | -0.808 | -0.3708 | No |
| 43 | ME2 |  |  | 4499 | -1.062 | -0.4160 | No |
| 44 | PTGS1 |  |  | 4502 | -1.067 | -0.4131 | No |
| 45 | GSTZ1 |  |  | 4765 | -1.242 | -0.4469 | No |
| 46 | BBOX1 |  |  | 4972 | -1.409 | -0.4723 | No |
| 47 | HPGD |  |  | 5013 | -1.442 | -0.4737 | No |
| 48 | CPOX |  |  | 5024 | -1.458 | -0.4708 | No |
| 49 | SUOX |  |  | 5042 | -1.471 | -0.4689 | No |
| 50 | ABP1 |  |  | 5047 | -1.475 | -0.4651 | No |
| 51 | PPOX |  |  | 5159 | -1.558 | -0.4764 | No |
| 52 | ALOX12 |  |  | 5178 | -1.576 | -0.4743 | No |
| 53 | AOX1 |  |  | 5181 | -1.579 | -0.4699 | No |
| 54 | ALDH6A1 |  |  | 5237 | -1.625 | -0.4730 | No |
| 55 | SPR |  |  | 5269 | -1.647 | -0.4725 | No |
| 56 | EGLN1 |  |  | 5310 | -1.690 | -0.4733 | No |
| 57 | DECR1 |  |  | 5429 | -1.824 | -0.4848 | No |
| 58 | PRODH |  |  | 5435 | -1.832 | -0.4800 | No |
| 59 | NOS3 |  |  | 5486 | -1.890 | -0.4816 | No |
| 60 | COX10 |  |  | 5500 | -1.902 | -0.4778 | No |
| 61 | WWOX |  |  | 5512 | -1.912 | -0.4737 | No |
| 62 | CBR3 |  |  | 5695 | -2.137 | -0.4935 | No |
| 63 | APEX1 |  |  | 5813 | -2.288 | -0.5034 | Yes |
| 64 | TXNL1 |  |  | 5829 | -2.308 | -0.4987 | Yes |
| 65 | COX7A1 |  |  | 5855 | -2.332 | -0.4954 | Yes |
| 66 | CYP7B1 |  |  | 5915 | -2.422 | -0.4967 | Yes |
| 67 | PHYH |  |  | 5957 | -2.475 | -0.4952 | Yes |
| 68 | ACADM |  |  | 6051 | -2.581 | -0.5009 | Yes |
| 69 | ALOX15 |  |  | 6081 | -2.614 | -0.4973 | Yes |
| 70 | MAOB |  |  | 6130 | -2.689 | -0.4962 | Yes |
| 71 | ACADSB |  |  | 6233 | -2.869 | -0.5023 | Yes |
| 72 | HSD17B7 |  |  | 6273 | -2.938 | -0.4992 | Yes |
| 73 | FDXR |  |  | 6304 | -2.999 | -0.4946 | Yes |
| 74 | SOD2 |  |  | 6412 | -3.219 | -0.5004 | Yes |
| 75 | DHCR24 |  |  | 6482 | -3.397 | -0.5002 | Yes |
| 76 | NDUFS2 |  |  | 6521 | -3.486 | -0.4953 | Yes |
| 77 | ACADL |  |  | 6546 | -3.535 | -0.4882 | Yes |
| 78 | SURF1 |  |  | 6573 | -3.596 | -0.4813 | Yes |
| 79 | IDH1 |  |  | 6580 | -3.613 | -0.4715 | Yes |
| 80 | ALDH2 |  |  | 6586 | -3.631 | -0.4614 | Yes |
| 81 | UQCRC1 |  |  | 6607 | -3.702 | -0.4533 | Yes |
| 82 | CDO1 |  |  | 6608 | -3.703 | -0.4423 | Yes |
| 83 | ALDH9A1 |  |  | 6622 | -3.751 | -0.4331 | Yes |
| 84 | COX11 |  |  | 6659 | -3.864 | -0.4268 | Yes |
| 85 | RDH10 |  |  | 6725 | -4.066 | -0.4240 | Yes |
| 86 | EHHADH |  |  | 6728 | -4.077 | -0.4122 | Yes |
| 87 | CYCS |  |  | 6766 | -4.280 | -0.4048 | Yes |
| 88 | IDH3B |  |  | 6776 | -4.331 | -0.3933 | Yes |
| 89 | NDUFA9 |  |  | 6784 | -4.356 | -0.3814 | Yes |
| 90 | HADHA |  |  | 6795 | -4.417 | -0.3697 | Yes |
| 91 | COX15 |  |  | 6801 | -4.443 | -0.3572 | Yes |
| 92 | FMO1 |  |  | 6805 | -4.457 | -0.3445 | Yes |
| 93 | SDHD |  |  | 6818 | -4.523 | -0.3328 | Yes |
| 94 | COX4I1 |  |  | 6819 | -4.529 | -0.3193 | Yes |
| 95 | MSRB2 |  |  | 6829 | -4.558 | -0.3071 | Yes |
| 96 | BCKDHA |  |  | 6876 | -4.864 | -0.2993 | Yes |
| 97 | CYC1 |  |  | 6879 | -4.925 | -0.2850 | Yes |
| 98 | MDH1 |  |  | 6882 | -4.948 | -0.2706 | Yes |
| 99 | DLD |  |  | 6927 | -5.255 | -0.2613 | Yes |
| 100 | IDH3G |  |  | 6935 | -5.359 | -0.2464 | Yes |
| 101 | NDUFS4 |  |  | 6939 | -5.391 | -0.2309 | Yes |
| 102 | TSTA3 |  |  | 6950 | -5.521 | -0.2159 | Yes |
| 103 | GPD1 |  |  | 6967 | -5.721 | -0.2012 | Yes |
| 104 | PRDX3 |  |  | 6994 | -6.158 | -0.1867 | Yes |
| 105 | NDUFS1 |  |  | 6998 | -6.186 | -0.1688 | Yes |
| 106 | COX7B |  |  | 7007 | -6.322 | -0.1512 | Yes |
| 107 | SC5DL |  |  | 7015 | -6.486 | -0.1330 | Yes |
| 108 | ALDH4A1 |  |  | 7025 | -6.784 | -0.1142 | Yes |
| 109 | COX5A |  |  | 7059 | -7.731 | -0.0960 | Yes |
| 110 | RDH11 |  |  | 7079 | -9.320 | -0.0710 | Yes |
| 111 | FADS1 |  |  | 7086 | -11.367 | -0.0382 | Yes |
| 112 | GPD2 |  |  | 7090 | -13.077 | 0.0001 | Yes |
Table: GSEA details [plain text format]

  

Fig 2: OXIDOREDUCTASE\_ACTIVITY: Random ES distribution      
 Gene set null distribution of ES for **OXIDOREDUCTASE\_ACTIVITY**

  
