# Supplementary material for: Network-based integration of molecular and physiological data elucidates regulatory mechanisms underlying adaptation to high-fat diet
Source: Genes Nutr. 2015 May 28;10(4):22. doi: 10.1007/s12263-015-0470-6 (PMC4446272; doi:10.1007/s12263-015-0470-6)
Supplement: Supplementary file 4 — Supplementary material 4 (ZIP 6984 kb) [file 12263_2015_470_MOESM4_ESM.zip › HF LF 12 w GSEA result/OXIDOREDUCTASE_ACTIVITY_ACTING_ON_CH_OH_GROUP_OF_DONORS.html]

Details for gene set OXIDOREDUCTASE\_ACTIVITY\_ACTING\_ON\_CH\_OH\_GROUP\_OF\_DONORS[GSEA]

|  || Dataset | HF LF 12w\_collapsed |
| Phenotype | NoPhenotypeAvailable |
| Upregulated in class | na\_neg |
| GeneSet | OXIDOREDUCTASE\_ACTIVITY\_ACTING\_ON\_CH\_OH\_GROUP\_OF\_DONORS |
| Enrichment Score (ES) | -0.75572443 |
| Normalized Enrichment Score (NES) | -2.161848 |
| Nominal p-value | 0.0 |
| FDR q-value | 1.2457072E-4 |
| FWER p-Value | 0.002 |
Table: GSEA Results Summary

  

Fig 1: Enrichment plot: OXIDOREDUCTASE\_ACTIVITY\_ACTING\_ON\_CH\_OH\_GROUP\_OF\_DONORS      
 Profile of the Running ES Score & Positions of GeneSet Members on the Rank Ordered List

  

| PROBE | GENE SYMBOL | GENE\_TITLE | RANK IN GENE LIST | RANK METRIC SCORE | RUNNING ES | CORE ENRICHMENT || 1 | LDHC |  |  | 1195 | 1.765 | -0.1463 | No |
| 2 | ADH4 |  |  | 1365 | 1.574 | -0.1501 | No |
| 3 | IMPDH1 |  |  | 2242 | 0.649 | -0.2656 | No |
| 4 | HSD17B4 |  |  | 3449 | -0.310 | -0.4322 | No |
| 5 | ME2 |  |  | 4499 | -1.062 | -0.5669 | No |
| 6 | HPGD |  |  | 5013 | -1.442 | -0.6209 | No |
| 7 | SPR |  |  | 5269 | -1.647 | -0.6359 | No |
| 8 | CBR3 |  |  | 5695 | -2.137 | -0.6686 | No |
| 9 | HSD17B7 |  |  | 6273 | -2.938 | -0.7125 | Yes |
| 10 | IDH1 |  |  | 6580 | -3.613 | -0.7094 | Yes |
| 11 | RDH10 |  |  | 6725 | -4.066 | -0.6776 | Yes |
| 12 | EHHADH |  |  | 6728 | -4.077 | -0.6256 | Yes |
| 13 | IDH3B |  |  | 6776 | -4.331 | -0.5767 | Yes |
| 14 | HADHA |  |  | 6795 | -4.417 | -0.5226 | Yes |
| 15 | MDH1 |  |  | 6882 | -4.948 | -0.4713 | Yes |
| 16 | IDH3G |  |  | 6935 | -5.359 | -0.4099 | Yes |
| 17 | TSTA3 |  |  | 6950 | -5.521 | -0.3411 | Yes |
| 18 | GPD1 |  |  | 6967 | -5.721 | -0.2700 | Yes |
| 19 | RDH11 |  |  | 7079 | -9.320 | -0.1662 | Yes |
| 20 | GPD2 |  |  | 7090 | -13.077 | 0.0001 | Yes |
Table: GSEA details [plain text format]

  

Fig 2: OXIDOREDUCTASE\_ACTIVITY\_ACTING\_ON\_CH\_OH\_GROUP\_OF\_DONORS: Random ES distribution      
 Gene set null distribution of ES for **OXIDOREDUCTASE\_ACTIVITY\_ACTING\_ON\_CH\_OH\_GROUP\_OF\_DONORS**

  
