# Supplementary material for: Network-based integration of molecular and physiological data elucidates regulatory mechanisms underlying adaptation to high-fat diet
Source: Genes Nutr. 2015 May 28;10(4):22. doi: 10.1007/s12263-015-0470-6 (PMC4446272; doi:10.1007/s12263-015-0470-6)
Supplement: Supplementary file 4 — Supplementary material 4 (ZIP 6984 kb) [file 12263_2015_470_MOESM4_ESM.zip › HF LF 12 w GSEA result/RECEPTOR_ACTIVITY.html]

Details for gene set RECEPTOR\_ACTIVITY[GSEA]

|  || Dataset | HF LF 12w\_collapsed |
| Phenotype | NoPhenotypeAvailable |
| Upregulated in class | na\_pos |
| GeneSet | RECEPTOR\_ACTIVITY |
| Enrichment Score (ES) | 0.44469687 |
| Normalized Enrichment Score (NES) | 2.141138 |
| Nominal p-value | 0.0 |
| FDR q-value | 0.001331537 |
| FWER p-Value | 0.011 |
Table: GSEA Results Summary

  

Fig 1: Enrichment plot: RECEPTOR\_ACTIVITY      
 Profile of the Running ES Score & Positions of GeneSet Members on the Rank Ordered List

  

| PROBE | GENE SYMBOL | GENE\_TITLE | RANK IN GENE LIST | RANK METRIC SCORE | RUNNING ES | CORE ENRICHMENT || 1 | ITGAX |  |  | 12 | 7.994 | 0.0270 | Yes |
| 2 | NR1D1 |  |  | 89 | 5.439 | 0.0356 | Yes |
| 3 | IL7R |  |  | 99 | 5.294 | 0.0533 | Yes |
| 4 | ACVR1B |  |  | 109 | 5.209 | 0.0707 | Yes |
| 5 | CLEC7A |  |  | 121 | 5.093 | 0.0874 | Yes |
| 6 | IL10RA |  |  | 174 | 4.693 | 0.0968 | Yes |
| 7 | LILRB4 |  |  | 236 | 4.332 | 0.1035 | Yes |
| 8 | IGSF6 |  |  | 240 | 4.319 | 0.1186 | Yes |
| 9 | LRRN2 |  |  | 244 | 4.293 | 0.1336 | Yes |
| 10 | PVR |  |  | 284 | 4.051 | 0.1425 | Yes |
| 11 | P2RX7 |  |  | 297 | 3.969 | 0.1551 | Yes |
| 12 | PTGER2 |  |  | 303 | 3.938 | 0.1685 | Yes |
| 13 | TNFRSF11B |  |  | 341 | 3.753 | 0.1766 | Yes |
| 14 | CHRM4 |  |  | 356 | 3.695 | 0.1879 | Yes |
| 15 | MERTK |  |  | 357 | 3.690 | 0.2011 | Yes |
| 16 | FZD4 |  |  | 384 | 3.566 | 0.2102 | Yes |
| 17 | IFNGR2 |  |  | 395 | 3.513 | 0.2214 | Yes |
| 18 | MST1R |  |  | 419 | 3.441 | 0.2304 | Yes |
| 19 | PROCR |  |  | 421 | 3.432 | 0.2426 | Yes |
| 20 | RRBP1 |  |  | 437 | 3.376 | 0.2526 | Yes |
| 21 | GPR133 |  |  | 440 | 3.349 | 0.2643 | Yes |
| 22 | C3AR1 |  |  | 443 | 3.340 | 0.2760 | Yes |
| 23 | STAB1 |  |  | 459 | 3.268 | 0.2856 | Yes |
| 24 | SMO |  |  | 476 | 3.200 | 0.2948 | Yes |
| 25 | ICAM1 |  |  | 488 | 3.156 | 0.3045 | Yes |
| 26 | CCR5 |  |  | 497 | 3.131 | 0.3146 | Yes |
| 27 | CSF1R |  |  | 519 | 3.047 | 0.3225 | Yes |
| 28 | P2RY1 |  |  | 527 | 3.024 | 0.3324 | Yes |
| 29 | AMHR2 |  |  | 540 | 2.991 | 0.3414 | Yes |
| 30 | IL6ST |  |  | 544 | 2.976 | 0.3516 | Yes |
| 31 | SCARA3 |  |  | 549 | 2.967 | 0.3617 | Yes |
| 32 | OSMR |  |  | 558 | 2.943 | 0.3711 | Yes |
| 33 | TLR2 |  |  | 580 | 2.875 | 0.3784 | Yes |
| 34 | LRP1 |  |  | 687 | 2.610 | 0.3726 | Yes |
| 35 | FCGRT |  |  | 698 | 2.580 | 0.3804 | Yes |
| 36 | PDGFRA |  |  | 746 | 2.463 | 0.3825 | Yes |
| 37 | SPN |  |  | 761 | 2.439 | 0.3892 | Yes |
| 38 | ADRB1 |  |  | 781 | 2.389 | 0.3951 | Yes |
| 39 | PTPRB |  |  | 827 | 2.305 | 0.3969 | Yes |
| 40 | ASGR2 |  |  | 850 | 2.270 | 0.4018 | Yes |
| 41 | NR1I3 |  |  | 863 | 2.245 | 0.4082 | Yes |
| 42 | IL27RA |  |  | 943 | 2.116 | 0.4044 | Yes |
| 43 | NRP1 |  |  | 979 | 2.076 | 0.4068 | Yes |
| 44 | CHRNB1 |  |  | 1038 | 1.988 | 0.4056 | Yes |
| 45 | GABBR1 |  |  | 1066 | 1.953 | 0.4087 | Yes |
| 46 | TGFBR2 |  |  | 1084 | 1.933 | 0.4132 | Yes |
| 47 | CXCL16 |  |  | 1111 | 1.893 | 0.4163 | Yes |
| 48 | XPR1 |  |  | 1132 | 1.858 | 0.4201 | Yes |
| 49 | TNFRSF1A |  |  | 1146 | 1.835 | 0.4248 | Yes |
| 50 | EGFR |  |  | 1225 | 1.728 | 0.4198 | Yes |
| 51 | GPR124 |  |  | 1252 | 1.694 | 0.4221 | Yes |
| 52 | MSR1 |  |  | 1260 | 1.684 | 0.4272 | Yes |
| 53 | RTN4RL1 |  |  | 1316 | 1.633 | 0.4251 | Yes |
| 54 | CD7 |  |  | 1335 | 1.604 | 0.4283 | Yes |
| 55 | GPR125 |  |  | 1432 | 1.498 | 0.4199 | Yes |
| 56 | CD14 |  |  | 1450 | 1.486 | 0.4228 | Yes |
| 57 | COLEC12 |  |  | 1451 | 1.484 | 0.4281 | Yes |
| 58 | TRAM1 |  |  | 1464 | 1.470 | 0.4316 | Yes |
| 59 | IFNGR1 |  |  | 1522 | 1.407 | 0.4285 | Yes |
| 60 | TNFRSF17 |  |  | 1523 | 1.406 | 0.4335 | Yes |
| 61 | IL31RA |  |  | 1545 | 1.384 | 0.4355 | Yes |
| 62 | PLXNA1 |  |  | 1570 | 1.356 | 0.4369 | Yes |
| 63 | P2RY2 |  |  | 1573 | 1.354 | 0.4415 | Yes |
| 64 | NR3C1 |  |  | 1631 | 1.286 | 0.4379 | Yes |
| 65 | HRH3 |  |  | 1638 | 1.276 | 0.4416 | Yes |
| 66 | PTPRK |  |  | 1649 | 1.262 | 0.4447 | Yes |
| 67 | IL1R1 |  |  | 1734 | 1.169 | 0.4368 | No |
| 68 | MARCO |  |  | 1752 | 1.153 | 0.4385 | No |
| 69 | MTNR1B |  |  | 1777 | 1.116 | 0.4391 | No |
| 70 | VIPR1 |  |  | 1990 | 0.868 | 0.4117 | No |
| 71 | PRKD3 |  |  | 2018 | 0.842 | 0.4108 | No |
| 72 | ABCA1 |  |  | 2045 | 0.815 | 0.4100 | No |
| 73 | LIFR |  |  | 2085 | 0.775 | 0.4072 | No |
| 74 | ELTD1 |  |  | 2240 | 0.650 | 0.3873 | No |
| 75 | OPRM1 |  |  | 2261 | 0.636 | 0.3868 | No |
| 76 | RTN4RL2 |  |  | 2312 | 0.576 | 0.3816 | No |
| 77 | GHR |  |  | 2319 | 0.573 | 0.3828 | No |
| 78 | FZD2 |  |  | 2339 | 0.559 | 0.3821 | No |
| 79 | SEC63 |  |  | 2440 | 0.460 | 0.3694 | No |
| 80 | FLT1 |  |  | 2449 | 0.451 | 0.3698 | No |
| 81 | IL2RB |  |  | 2530 | 0.381 | 0.3597 | No |
| 82 | PTGER4 |  |  | 2543 | 0.368 | 0.3593 | No |
| 83 | GPR56 |  |  | 2669 | 0.261 | 0.3422 | No |
| 84 | LY96 |  |  | 2742 | 0.214 | 0.3327 | No |
| 85 | PTK7 |  |  | 2770 | 0.193 | 0.3295 | No |
| 86 | NRP2 |  |  | 2797 | 0.165 | 0.3263 | No |
| 87 | RXRG |  |  | 2866 | 0.110 | 0.3169 | No |
| 88 | NPY |  |  | 2955 | 0.042 | 0.3044 | No |
| 89 | GABRR1 |  |  | 3013 | 0.001 | 0.2962 | No |
| 90 | LGTN |  |  | 3056 | -0.036 | 0.2903 | No |
| 91 | MED12 |  |  | 3060 | -0.040 | 0.2900 | No |
| 92 | RNF139 |  |  | 3077 | -0.058 | 0.2879 | No |
| 93 | PRKCH |  |  | 3104 | -0.071 | 0.2844 | No |
| 94 | SLC1A5 |  |  | 3112 | -0.076 | 0.2837 | No |
| 95 | PRKCZ |  |  | 3175 | -0.116 | 0.2752 | No |
| 96 | RYK |  |  | 3211 | -0.144 | 0.2707 | No |
| 97 | TSHR |  |  | 3290 | -0.193 | 0.2602 | No |
| 98 | FLT4 |  |  | 3391 | -0.263 | 0.2467 | No |
| 99 | LANCL1 |  |  | 3400 | -0.268 | 0.2465 | No |
| 100 | TBXA2R |  |  | 3408 | -0.278 | 0.2465 | No |
| 101 | RXRA |  |  | 3440 | -0.306 | 0.2432 | No |
| 102 | NR2C1 |  |  | 3473 | -0.324 | 0.2397 | No |
| 103 | TIE1 |  |  | 3482 | -0.333 | 0.2398 | No |
| 104 | GUCY1B3 |  |  | 3486 | -0.337 | 0.2405 | No |
| 105 | EPOR |  |  | 3533 | -0.367 | 0.2352 | No |
| 106 | ADCYAP1R1 |  |  | 3598 | -0.409 | 0.2275 | No |
| 107 | CLEC1A |  |  | 3708 | -0.489 | 0.2136 | No |
| 108 | ITPR3 |  |  | 3737 | -0.513 | 0.2114 | No |
| 109 | DRD4 |  |  | 3748 | -0.521 | 0.2118 | No |
| 110 | CUL5 |  |  | 3839 | -0.587 | 0.2010 | No |
| 111 | MMD |  |  | 3949 | -0.676 | 0.1877 | No |
| 112 | FZD7 |  |  | 4105 | -0.772 | 0.1682 | No |
| 113 | PLXNB1 |  |  | 4109 | -0.775 | 0.1705 | No |
| 114 | CXCR4 |  |  | 4118 | -0.780 | 0.1722 | No |
| 115 | FZD6 |  |  | 4125 | -0.783 | 0.1741 | No |
| 116 | F2R |  |  | 4331 | -0.934 | 0.1480 | No |
| 117 | TAS1R3 |  |  | 4349 | -0.950 | 0.1490 | No |
| 118 | LGALS3BP |  |  | 4377 | -0.971 | 0.1486 | No |
| 119 | PDGFRL |  |  | 4492 | -1.055 | 0.1360 | No |
| 120 | GRIK5 |  |  | 4565 | -1.107 | 0.1296 | No |
| 121 | IFNAR2 |  |  | 4675 | -1.183 | 0.1181 | No |
| 122 | HPGD |  |  | 5013 | -1.442 | 0.0748 | No |
| 123 | EFNA4 |  |  | 5243 | -1.630 | 0.0477 | No |
| 124 | IGF2R |  |  | 5330 | -1.708 | 0.0415 | No |
| 125 | LPHN1 |  |  | 5342 | -1.722 | 0.0461 | No |
| 126 | EBP |  |  | 5436 | -1.832 | 0.0393 | No |
| 127 | MET |  |  | 5442 | -1.838 | 0.0452 | No |
| 128 | THRA |  |  | 5496 | -1.900 | 0.0444 | No |
| 129 | KDR |  |  | 5671 | -2.097 | 0.0269 | No |
| 130 | PVRL1 |  |  | 5674 | -2.105 | 0.0341 | No |
| 131 | PTPRZ1 |  |  | 5750 | -2.210 | 0.0313 | No |
| 132 | AVPR1A |  |  | 5777 | -2.236 | 0.0356 | No |
| 133 | SLC20A2 |  |  | 5884 | -2.371 | 0.0288 | No |
| 134 | PVRL2 |  |  | 6155 | -2.724 | -0.0002 | No |
| 135 | FKBP3 |  |  | 6191 | -2.788 | 0.0048 | No |
| 136 | NR2F6 |  |  | 6527 | -3.499 | -0.0309 | No |
| 137 | PGRMC2 |  |  | 6821 | -4.537 | -0.0567 | No |
| 138 | PTGER3 |  |  | 6866 | -4.787 | -0.0459 | No |
| 139 | MED4 |  |  | 6945 | -5.448 | -0.0375 | No |
| 140 | LRPAP1 |  |  | 7048 | -7.319 | -0.0259 | No |
| 141 | MR1 |  |  | 7075 | -8.907 | 0.0023 | No |
Table: GSEA details [plain text format]

  

Fig 2: RECEPTOR\_ACTIVITY: Random ES distribution      
 Gene set null distribution of ES for **RECEPTOR\_ACTIVITY**

  
