# Supplementary material for: Network-based integration of molecular and physiological data elucidates regulatory mechanisms underlying adaptation to high-fat diet
Source: Genes Nutr. 2015 May 28;10(4):22. doi: 10.1007/s12263-015-0470-6 (PMC4446272; doi:10.1007/s12263-015-0470-6)
Supplement: Supplementary file 4 — Supplementary material 4 (ZIP 6984 kb) [file 12263_2015_470_MOESM4_ESM.zip › HF LF 12 w GSEA result/RESPONSE_TO_EXTERNAL_STIMULUS.html]

Details for gene set RESPONSE\_TO\_EXTERNAL\_STIMULUS[GSEA]

|  || Dataset | HF LF 12w\_collapsed |
| Phenotype | NoPhenotypeAvailable |
| Upregulated in class | na\_pos |
| GeneSet | RESPONSE\_TO\_EXTERNAL\_STIMULUS |
| Enrichment Score (ES) | 0.5113813 |
| Normalized Enrichment Score (NES) | 2.2937474 |
| Nominal p-value | 0.0 |
| FDR q-value | 4.7424197E-4 |
| FWER p-Value | 0.002 |
Table: GSEA Results Summary

  

Fig 1: Enrichment plot: RESPONSE\_TO\_EXTERNAL\_STIMULUS      
 Profile of the Running ES Score & Positions of GeneSet Members on the Rank Ordered List

  

| PROBE | GENE SYMBOL | GENE\_TITLE | RANK IN GENE LIST | RANK METRIC SCORE | RUNNING ES | CORE ENRICHMENT || 1 | LTB4R |  |  | 14 | 7.889 | 0.0293 | Yes |
| 2 | CCL7 |  |  | 17 | 7.530 | 0.0588 | Yes |
| 3 | CCR3 |  |  | 46 | 6.449 | 0.0804 | Yes |
| 4 | PROS1 |  |  | 62 | 5.993 | 0.1020 | Yes |
| 5 | ITGB2 |  |  | 86 | 5.478 | 0.1204 | Yes |
| 6 | C2 |  |  | 103 | 5.276 | 0.1391 | Yes |
| 7 | CCR2 |  |  | 112 | 5.179 | 0.1585 | Yes |
| 8 | PLAUR |  |  | 117 | 5.129 | 0.1782 | Yes |
| 9 | ALOX5AP |  |  | 139 | 4.956 | 0.1949 | Yes |
| 10 | MEFV |  |  | 143 | 4.917 | 0.2139 | Yes |
| 11 | CCL2 |  |  | 148 | 4.900 | 0.2328 | Yes |
| 12 | CCBP2 |  |  | 175 | 4.685 | 0.2476 | Yes |
| 13 | LBP |  |  | 176 | 4.677 | 0.2662 | Yes |
| 14 | F13A1 |  |  | 201 | 4.543 | 0.2808 | Yes |
| 15 | CCL11 |  |  | 222 | 4.439 | 0.2955 | Yes |
| 16 | CCL24 |  |  | 224 | 4.436 | 0.3129 | Yes |
| 17 | LEP |  |  | 227 | 4.387 | 0.3300 | Yes |
| 18 | DOCK2 |  |  | 228 | 4.377 | 0.3474 | Yes |
| 19 | FOS |  |  | 265 | 4.135 | 0.3586 | Yes |
| 20 | THBD |  |  | 275 | 4.110 | 0.3736 | Yes |
| 21 | ADM |  |  | 301 | 3.945 | 0.3857 | Yes |
| 22 | RASGRP4 |  |  | 331 | 3.801 | 0.3966 | Yes |
| 23 | FGF2 |  |  | 348 | 3.718 | 0.4091 | Yes |
| 24 | STC2 |  |  | 358 | 3.669 | 0.4223 | Yes |
| 25 | C3AR1 |  |  | 443 | 3.340 | 0.4236 | Yes |
| 26 | F10 |  |  | 460 | 3.264 | 0.4342 | Yes |
| 27 | CCL4 |  |  | 486 | 3.157 | 0.4432 | Yes |
| 28 | CCR5 |  |  | 497 | 3.131 | 0.4541 | Yes |
| 29 | WAS |  |  | 507 | 3.096 | 0.4651 | Yes |
| 30 | CCRL1 |  |  | 581 | 2.868 | 0.4661 | Yes |
| 31 | NFATC4 |  |  | 621 | 2.761 | 0.4714 | Yes |
| 32 | PF4 |  |  | 623 | 2.761 | 0.4822 | Yes |
| 33 | AOC3 |  |  | 641 | 2.718 | 0.4906 | Yes |
| 34 | CCL17 |  |  | 696 | 2.594 | 0.4931 | Yes |
| 35 | CCL5 |  |  | 752 | 2.451 | 0.4950 | Yes |
| 36 | SPN |  |  | 761 | 2.439 | 0.5035 | Yes |
| 37 | PLAT |  |  | 782 | 2.389 | 0.5101 | Yes |
| 38 | TRPV4 |  |  | 872 | 2.233 | 0.5062 | Yes |
| 39 | TFPI |  |  | 898 | 2.197 | 0.5114 | Yes |
| 40 | CTGF |  |  | 1033 | 1.994 | 0.5001 | No |
| 41 | TNFRSF1A |  |  | 1146 | 1.835 | 0.4914 | No |
| 42 | CXCR3 |  |  | 1226 | 1.728 | 0.4869 | No |
| 43 | RALBP1 |  |  | 1286 | 1.653 | 0.4850 | No |
| 44 | RTN4RL1 |  |  | 1316 | 1.633 | 0.4874 | No |
| 45 | AIF1 |  |  | 1336 | 1.603 | 0.4910 | No |
| 46 | S100A8 |  |  | 1340 | 1.600 | 0.4969 | No |
| 47 | VWF |  |  | 1356 | 1.583 | 0.5010 | No |
| 48 | CXCL9 |  |  | 1395 | 1.543 | 0.5017 | No |
| 49 | HDAC4 |  |  | 1596 | 1.330 | 0.4784 | No |
| 50 | PARP4 |  |  | 1628 | 1.290 | 0.4791 | No |
| 51 | CXCL12 |  |  | 1639 | 1.275 | 0.4827 | No |
| 52 | KLK8 |  |  | 1750 | 1.156 | 0.4715 | No |
| 53 | ABCF1 |  |  | 1787 | 1.105 | 0.4708 | No |
| 54 | TGFB1 |  |  | 2164 | 0.718 | 0.4198 | No |
| 55 | RAC1 |  |  | 2194 | 0.686 | 0.4184 | No |
| 56 | CCL8 |  |  | 2273 | 0.620 | 0.4097 | No |
| 57 | OGT |  |  | 2303 | 0.587 | 0.4079 | No |
| 58 | RTN4RL2 |  |  | 2312 | 0.576 | 0.4090 | No |
| 59 | INHA |  |  | 2493 | 0.417 | 0.3849 | No |
| 60 | GP9 |  |  | 2561 | 0.351 | 0.3767 | No |
| 61 | CD36 |  |  | 2659 | 0.269 | 0.3639 | No |
| 62 | INHBA |  |  | 2769 | 0.194 | 0.3491 | No |
| 63 | AHSG |  |  | 2834 | 0.131 | 0.3405 | No |
| 64 | GGCX |  |  | 2901 | 0.079 | 0.3314 | No |
| 65 | PLA2G2D |  |  | 2954 | 0.044 | 0.3241 | No |
| 66 | NPY |  |  | 2955 | 0.042 | 0.3243 | No |
| 67 | MAPK1 |  |  | 2984 | 0.019 | 0.3203 | No |
| 68 | NFATC3 |  |  | 3127 | -0.085 | 0.3004 | No |
| 69 | NFRKB |  |  | 3325 | -0.216 | 0.2730 | No |
| 70 | MMRN1 |  |  | 3472 | -0.324 | 0.2534 | No |
| 71 | TGFB2 |  |  | 3518 | -0.356 | 0.2484 | No |
| 72 | LMAN1 |  |  | 3664 | -0.459 | 0.2295 | No |
| 73 | CDKN1A |  |  | 3785 | -0.540 | 0.2145 | No |
| 74 | F8 |  |  | 3806 | -0.558 | 0.2138 | No |
| 75 | F11R |  |  | 4020 | -0.711 | 0.1862 | No |
| 76 | CDH13 |  |  | 4038 | -0.728 | 0.1866 | No |
| 77 | CXCR4 |  |  | 4118 | -0.780 | 0.1784 | No |
| 78 | F2R |  |  | 4331 | -0.934 | 0.1518 | No |
| 79 | CX3CL1 |  |  | 4357 | -0.956 | 0.1520 | No |
| 80 | PROC |  |  | 4374 | -0.968 | 0.1535 | No |
| 81 | F9 |  |  | 4473 | -1.037 | 0.1436 | No |
| 82 | EIF2AK1 |  |  | 4962 | -1.397 | 0.0794 | No |
| 83 | NFX1 |  |  | 5027 | -1.459 | 0.0760 | No |
| 84 | PLAU |  |  | 5056 | -1.483 | 0.0779 | No |
| 85 | ELF3 |  |  | 5164 | -1.567 | 0.0688 | No |
| 86 | AOX1 |  |  | 5181 | -1.579 | 0.0728 | No |
| 87 | RALA |  |  | 5456 | -1.854 | 0.0409 | No |
| 88 | CXCL13 |  |  | 5611 | -2.017 | 0.0269 | No |
| 89 | TRPC3 |  |  | 5627 | -2.039 | 0.0328 | No |
| 90 | CX3CR1 |  |  | 5971 | -2.485 | -0.0064 | No |
| 91 | CXCL14 |  |  | 5975 | -2.489 | 0.0031 | No |
| 92 | TMPRSS6 |  |  | 6047 | -2.576 | 0.0031 | No |
| 93 | ALOX15 |  |  | 6081 | -2.614 | 0.0088 | No |
| 94 | MAP2K1 |  |  | 6086 | -2.621 | 0.0186 | No |
| 95 | PIK3CB |  |  | 6111 | -2.656 | 0.0257 | No |
| 96 | CCL25 |  |  | 6223 | -2.843 | 0.0211 | No |
| 97 | ORM1 |  |  | 6398 | -3.186 | 0.0088 | No |
| 98 | ORM2 |  |  | 6469 | -3.363 | 0.0121 | No |
| 99 | CDO1 |  |  | 6608 | -3.703 | 0.0071 | No |
| 100 | PPARG |  |  | 6752 | -4.204 | 0.0033 | No |
| 101 | FADS1 |  |  | 7086 | -11.367 | 0.0007 | No |
Table: GSEA details [plain text format]

  

Fig 2: RESPONSE\_TO\_EXTERNAL\_STIMULUS: Random ES distribution      
 Gene set null distribution of ES for **RESPONSE\_TO\_EXTERNAL\_STIMULUS**

  
