# Supplementary material for: Network-based integration of molecular and physiological data elucidates regulatory mechanisms underlying adaptation to high-fat diet
Source: Genes Nutr. 2015 May 28;10(4):22. doi: 10.1007/s12263-015-0470-6 (PMC4446272; doi:10.1007/s12263-015-0470-6)
Supplement: Supplementary file 4 — Supplementary material 4 (ZIP 6984 kb) [file 12263_2015_470_MOESM4_ESM.zip › HF LF 12 w GSEA result/RESPONSE_TO_WOUNDING.html]

Details for gene set RESPONSE\_TO\_WOUNDING[GSEA]

|  || Dataset | HF LF 12w\_collapsed |
| Phenotype | NoPhenotypeAvailable |
| Upregulated in class | na\_pos |
| GeneSet | RESPONSE\_TO\_WOUNDING |
| Enrichment Score (ES) | 0.5576161 |
| Normalized Enrichment Score (NES) | 2.324352 |
| Nominal p-value | 0.0 |
| FDR q-value | 2.830375E-4 |
| FWER p-Value | 0.001 |
Table: GSEA Results Summary

  

Fig 1: Enrichment plot: RESPONSE\_TO\_WOUNDING      
 Profile of the Running ES Score & Positions of GeneSet Members on the Rank Ordered List

  

| PROBE | GENE SYMBOL | GENE\_TITLE | RANK IN GENE LIST | RANK METRIC SCORE | RUNNING ES | CORE ENRICHMENT || 1 | LTB4R |  |  | 14 | 7.889 | 0.0500 | Yes |
| 2 | CCR3 |  |  | 46 | 6.449 | 0.0881 | Yes |
| 3 | PROS1 |  |  | 62 | 5.993 | 0.1255 | Yes |
| 4 | C2 |  |  | 103 | 5.276 | 0.1546 | Yes |
| 5 | CCR2 |  |  | 112 | 5.179 | 0.1876 | Yes |
| 6 | ALOX5AP |  |  | 139 | 4.956 | 0.2166 | Yes |
| 7 | MEFV |  |  | 143 | 4.917 | 0.2486 | Yes |
| 8 | LBP |  |  | 176 | 4.677 | 0.2749 | Yes |
| 9 | F13A1 |  |  | 201 | 4.543 | 0.3014 | Yes |
| 10 | CCL11 |  |  | 222 | 4.439 | 0.3279 | Yes |
| 11 | CCL24 |  |  | 224 | 4.436 | 0.3570 | Yes |
| 12 | FOS |  |  | 265 | 4.135 | 0.3785 | Yes |
| 13 | THBD |  |  | 275 | 4.110 | 0.4044 | Yes |
| 14 | ADM |  |  | 301 | 3.945 | 0.4268 | Yes |
| 15 | C3AR1 |  |  | 443 | 3.340 | 0.4288 | Yes |
| 16 | F10 |  |  | 460 | 3.264 | 0.4480 | Yes |
| 17 | CCL4 |  |  | 486 | 3.157 | 0.4653 | Yes |
| 18 | CCR5 |  |  | 497 | 3.131 | 0.4845 | Yes |
| 19 | WAS |  |  | 507 | 3.096 | 0.5036 | Yes |
| 20 | NFATC4 |  |  | 621 | 2.761 | 0.5058 | Yes |
| 21 | PF4 |  |  | 623 | 2.761 | 0.5238 | Yes |
| 22 | AOC3 |  |  | 641 | 2.718 | 0.5393 | Yes |
| 23 | CCL5 |  |  | 752 | 2.451 | 0.5398 | Yes |
| 24 | PLAT |  |  | 782 | 2.389 | 0.5515 | Yes |
| 25 | TFPI |  |  | 898 | 2.197 | 0.5496 | Yes |
| 26 | CTGF |  |  | 1033 | 1.994 | 0.5437 | Yes |
| 27 | TNFRSF1A |  |  | 1146 | 1.835 | 0.5398 | Yes |
| 28 | RTN4RL1 |  |  | 1316 | 1.633 | 0.5266 | Yes |
| 29 | AIF1 |  |  | 1336 | 1.603 | 0.5344 | Yes |
| 30 | S100A8 |  |  | 1340 | 1.600 | 0.5445 | Yes |
| 31 | VWF |  |  | 1356 | 1.583 | 0.5528 | Yes |
| 32 | CXCL9 |  |  | 1395 | 1.543 | 0.5576 | Yes |
| 33 | HDAC4 |  |  | 1596 | 1.330 | 0.5379 | No |
| 34 | PARP4 |  |  | 1628 | 1.290 | 0.5420 | No |
| 35 | KLK8 |  |  | 1750 | 1.156 | 0.5324 | No |
| 36 | ABCF1 |  |  | 1787 | 1.105 | 0.5346 | No |
| 37 | TGFB1 |  |  | 2164 | 0.718 | 0.4858 | No |
| 38 | RAC1 |  |  | 2194 | 0.686 | 0.4862 | No |
| 39 | RTN4RL2 |  |  | 2312 | 0.576 | 0.4734 | No |
| 40 | GP9 |  |  | 2561 | 0.351 | 0.4404 | No |
| 41 | CD36 |  |  | 2659 | 0.269 | 0.4283 | No |
| 42 | AHSG |  |  | 2834 | 0.131 | 0.4045 | No |
| 43 | GGCX |  |  | 2901 | 0.079 | 0.3956 | No |
| 44 | PLA2G2D |  |  | 2954 | 0.044 | 0.3885 | No |
| 45 | NFATC3 |  |  | 3127 | -0.085 | 0.3646 | No |
| 46 | NFRKB |  |  | 3325 | -0.216 | 0.3379 | No |
| 47 | MMRN1 |  |  | 3472 | -0.324 | 0.3193 | No |
| 48 | TGFB2 |  |  | 3518 | -0.356 | 0.3152 | No |
| 49 | LMAN1 |  |  | 3664 | -0.459 | 0.2976 | No |
| 50 | F8 |  |  | 3806 | -0.558 | 0.2813 | No |
| 51 | F11R |  |  | 4020 | -0.711 | 0.2556 | No |
| 52 | CXCR4 |  |  | 4118 | -0.780 | 0.2470 | No |
| 53 | F2R |  |  | 4331 | -0.934 | 0.2230 | No |
| 54 | CX3CL1 |  |  | 4357 | -0.956 | 0.2257 | No |
| 55 | PROC |  |  | 4374 | -0.968 | 0.2298 | No |
| 56 | F9 |  |  | 4473 | -1.037 | 0.2227 | No |
| 57 | NFX1 |  |  | 5027 | -1.459 | 0.1536 | No |
| 58 | ELF3 |  |  | 5164 | -1.567 | 0.1446 | No |
| 59 | AOX1 |  |  | 5181 | -1.579 | 0.1527 | No |
| 60 | CX3CR1 |  |  | 5971 | -2.485 | 0.0568 | No |
| 61 | TMPRSS6 |  |  | 6047 | -2.576 | 0.0632 | No |
| 62 | ALOX15 |  |  | 6081 | -2.614 | 0.0757 | No |
| 63 | ORM1 |  |  | 6398 | -3.186 | 0.0517 | No |
| 64 | ORM2 |  |  | 6469 | -3.363 | 0.0640 | No |
| 65 | CDO1 |  |  | 6608 | -3.703 | 0.0687 | No |
Table: GSEA details [plain text format]

  

Fig 2: RESPONSE\_TO\_WOUNDING: Random ES distribution      
 Gene set null distribution of ES for **RESPONSE\_TO\_WOUNDING**

  
