# Supplementary material for: Network-based integration of molecular and physiological data elucidates regulatory mechanisms underlying adaptation to high-fat diet
Source: Genes Nutr. 2015 May 28;10(4):22. doi: 10.1007/s12263-015-0470-6 (PMC4446272; doi:10.1007/s12263-015-0470-6)
Supplement: Supplementary file 4 — Supplementary material 4 (ZIP 6984 kb) [file 12263_2015_470_MOESM4_ESM.zip › HF LF 12 w GSEA result/T_CELL_ACTIVATION.html]

Details for gene set T\_CELL\_ACTIVATION[GSEA]

|  || Dataset | HF LF 12w\_collapsed |
| Phenotype | NoPhenotypeAvailable |
| Upregulated in class | na\_pos |
| GeneSet | T\_CELL\_ACTIVATION |
| Enrichment Score (ES) | 0.7349468 |
| Normalized Enrichment Score (NES) | 2.067709 |
| Nominal p-value | 0.0 |
| FDR q-value | 0.0026065542 |
| FWER p-Value | 0.031 |
Table: GSEA Results Summary

  

Fig 1: Enrichment plot: T\_CELL\_ACTIVATION      
 Profile of the Running ES Score & Positions of GeneSet Members on the Rank Ordered List

  

| PROBE | GENE SYMBOL | GENE\_TITLE | RANK IN GENE LIST | RANK METRIC SCORE | RUNNING ES | CORE ENRICHMENT || 1 | CLEC7A |  |  | 121 | 5.093 | 0.1555 | Yes |
| 2 | CD2 |  |  | 451 | 3.299 | 0.2209 | Yes |
| 3 | ICOSLG |  |  | 546 | 2.975 | 0.3084 | Yes |
| 4 | EBI3 |  |  | 654 | 2.682 | 0.3842 | Yes |
| 5 | IL27 |  |  | 684 | 2.615 | 0.4687 | Yes |
| 6 | CD3D |  |  | 686 | 2.612 | 0.5571 | Yes |
| 7 | SIT1 |  |  | 811 | 2.338 | 0.6188 | Yes |
| 8 | SLA2 |  |  | 849 | 2.272 | 0.6906 | Yes |
| 9 | ELF4 |  |  | 1233 | 1.722 | 0.6948 | Yes |
| 10 | CD7 |  |  | 1335 | 1.604 | 0.7349 | Yes |
| 11 | LAT |  |  | 1798 | 1.084 | 0.7064 | No |
| 12 | JAG2 |  |  | 3166 | -0.108 | 0.5169 | No |
| 13 | SOCS5 |  |  | 3209 | -0.142 | 0.5158 | No |
| 14 | CD47 |  |  | 3284 | -0.189 | 0.5118 | No |
| 15 | CD24 |  |  | 4094 | -0.768 | 0.4235 | No |
Table: GSEA details [plain text format]

  

Fig 2: T\_CELL\_ACTIVATION: Random ES distribution      
 Gene set null distribution of ES for **T\_CELL\_ACTIVATION**

  
