# Supplementary material for: Network-based integration of molecular and physiological data elucidates regulatory mechanisms underlying adaptation to high-fat diet
Source: Genes Nutr. 2015 May 28;10(4):22. doi: 10.1007/s12263-015-0470-6 (PMC4446272; doi:10.1007/s12263-015-0470-6)
Supplement: Supplementary file 4 — Supplementary material 4 (ZIP 6984 kb) [file 12263_2015_470_MOESM4_ESM.zip › HF LF 12 w GSEA result/TRANSFERASE_ACTIVITY_TRANSFERRING_ALKYL_OR_ARYLOTHER_THAN_METHYLGROUPS.html]

Details for gene set TRANSFERASE\_ACTIVITY\_TRANSFERRING\_ALKYL\_OR\_ARYLOTHER\_THAN\_METHYLGROUPS[GSEA]

|  || Dataset | HF LF 12w\_collapsed |
| Phenotype | NoPhenotypeAvailable |
| Upregulated in class | na\_neg |
| GeneSet | TRANSFERASE\_ACTIVITY\_TRANSFERRING\_ALKYL\_OR\_ARYLOTHER\_THAN\_METHYLGROUPS |
| Enrichment Score (ES) | -0.6752678 |
| Normalized Enrichment Score (NES) | -1.8663179 |
| Nominal p-value | 0.0 |
| FDR q-value | 0.0072864206 |
| FWER p-Value | 0.193 |
Table: GSEA Results Summary

  

Fig 1: Enrichment plot: TRANSFERASE\_ACTIVITY\_TRANSFERRING\_ALKYL\_OR\_ARYLOTHER\_THAN\_METHYLGROUPS      
 Profile of the Running ES Score & Positions of GeneSet Members on the Rank Ordered List

  

| PROBE | GENE SYMBOL | GENE\_TITLE | RANK IN GENE LIST | RANK METRIC SCORE | RUNNING ES | CORE ENRICHMENT || 1 | PGGT1B |  |  | 2415 | 0.485 | -0.3284 | No |
| 2 | FNTB |  |  | 2574 | 0.337 | -0.3417 | No |
| 3 | SRM |  |  | 2774 | 0.187 | -0.3649 | No |
| 4 | GGPS1 |  |  | 2882 | 0.094 | -0.3775 | No |
| 5 | MGST3 |  |  | 3478 | -0.329 | -0.4528 | No |
| 6 | MGST1 |  |  | 3947 | -0.674 | -0.5010 | No |
| 7 | MAT2A |  |  | 4361 | -0.958 | -0.5338 | No |
| 8 | GSTT2 |  |  | 4621 | -1.147 | -0.5398 | No |
| 9 | GSTZ1 |  |  | 4765 | -1.242 | -0.5269 | No |
| 10 | GSTM5 |  |  | 4848 | -1.313 | -0.5035 | No |
| 11 | GSTM4 |  |  | 5264 | -1.643 | -0.5184 | No |
| 12 | RABGGTA |  |  | 5597 | -1.998 | -0.5120 | No |
| 13 | GSTM2 |  |  | 6753 | -4.204 | -0.5632 | Yes |
| 14 | GSTA3 |  |  | 7044 | -7.242 | -0.4111 | Yes |
| 15 | MGST2 |  |  | 7050 | -7.351 | -0.2159 | Yes |
| 16 | GSTA4 |  |  | 7071 | -8.312 | 0.0028 | Yes |
Table: GSEA details [plain text format]

  

Fig 2: TRANSFERASE\_ACTIVITY\_TRANSFERRING\_ALKYL\_OR\_ARYLOTHER\_THAN\_METHYLGROUPS: Random ES distribution      
 Gene set null distribution of ES for **TRANSFERASE\_ACTIVITY\_TRANSFERRING\_ALKYL\_OR\_ARYLOTHER\_THAN\_METHYLGROUPS**

  
