# Supplementary material for: Network-based integration of molecular and physiological data elucidates regulatory mechanisms underlying adaptation to high-fat diet
Source: Genes Nutr. 2015 May 28;10(4):22. doi: 10.1007/s12263-015-0470-6 (PMC4446272; doi:10.1007/s12263-015-0470-6)
Supplement: Supplementary file 4 — Supplementary material 4 (ZIP 6984 kb) [file 12263_2015_470_MOESM4_ESM.zip › HF LF 5 d GSEA result/AMINE_TRANSPORT.html]

Details for gene set AMINE\_TRANSPORT[GSEA]

|  || Dataset | comp\_HF5d-LF5d\_collapsed |
| Phenotype | NoPhenotypeAvailable |
| Upregulated in class | na\_pos |
| GeneSet | AMINE\_TRANSPORT |
| Enrichment Score (ES) | 0.6514795 |
| Normalized Enrichment Score (NES) | 1.9804045 |
| Nominal p-value | 0.0022371365 |
| FDR q-value | 0.013094396 |
| FWER p-Value | 0.099 |
Table: GSEA Results Summary

  

Fig 1: Enrichment plot: AMINE\_TRANSPORT      
 Profile of the Running ES Score & Positions of GeneSet Members on the Rank Ordered List

  

| PROBE | GENE SYMBOL | GENE\_TITLE | RANK IN GENE LIST | RANK METRIC SCORE | RUNNING ES | CORE ENRICHMENT || 1 | SLC7A8 |  |  | 129 | 2.442 | 0.1504 | Yes |
| 2 | SLC3A2 |  |  | 287 | 2.050 | 0.2698 | Yes |
| 3 | SLC25A12 |  |  | 578 | 1.620 | 0.3406 | Yes |
| 4 | SLC7A10 |  |  | 950 | 1.271 | 0.3760 | Yes |
| 5 | SLC16A10 |  |  | 1016 | 1.219 | 0.4510 | Yes |
| 6 | SLC18A2 |  |  | 1069 | 1.179 | 0.5251 | Yes |
| 7 | SLC1A3 |  |  | 1187 | 1.100 | 0.5845 | Yes |
| 8 | SLC22A3 |  |  | 1541 | 0.884 | 0.5956 | Yes |
| 9 | SLC1A1 |  |  | 1886 | 0.697 | 0.5952 | Yes |
| 10 | SLC25A13 |  |  | 1947 | 0.666 | 0.6327 | Yes |
| 11 | SLC38A3 |  |  | 2097 | 0.577 | 0.6515 | Yes |
| 12 | PRAF2 |  |  | 2715 | 0.294 | 0.5846 | No |
| 13 | CTNS |  |  | 2949 | 0.191 | 0.5648 | No |
| 14 | SLC1A5 |  |  | 3471 | -0.045 | 0.4943 | No |
| 15 | ARL6IP5 |  |  | 3865 | -0.247 | 0.4558 | No |
Table: GSEA details [plain text format]

  

Fig 2: AMINE\_TRANSPORT: Random ES distribution      
 Gene set null distribution of ES for **AMINE\_TRANSPORT**

  
