# Supplementary material for: Network-based integration of molecular and physiological data elucidates regulatory mechanisms underlying adaptation to high-fat diet
Source: Genes Nutr. 2015 May 28;10(4):22. doi: 10.1007/s12263-015-0470-6 (PMC4446272; doi:10.1007/s12263-015-0470-6)
Supplement: Supplementary file 4 — Supplementary material 4 (ZIP 6984 kb) [file 12263_2015_470_MOESM4_ESM.zip › HF LF 5 d GSEA result/BEHAVIOR.html]

Details for gene set BEHAVIOR[GSEA]

|  || Dataset | comp\_HF5d-LF5d\_collapsed |
| Phenotype | NoPhenotypeAvailable |
| Upregulated in class | na\_pos |
| GeneSet | BEHAVIOR |
| Enrichment Score (ES) | 0.49903402 |
| Normalized Enrichment Score (NES) | 1.9339297 |
| Nominal p-value | 0.0 |
| FDR q-value | 0.017694559 |
| FWER p-Value | 0.16 |
Table: GSEA Results Summary

  

Fig 1: Enrichment plot: BEHAVIOR      
 Profile of the Running ES Score & Positions of GeneSet Members on the Rank Ordered List

  

| PROBE | GENE SYMBOL | GENE\_TITLE | RANK IN GENE LIST | RANK METRIC SCORE | RUNNING ES | CORE ENRICHMENT || 1 | CCL8 |  |  | 56 | 2.814 | 0.0492 | Yes |
| 2 | LEP |  |  | 74 | 2.711 | 0.1019 | Yes |
| 3 | APOE |  |  | 86 | 2.652 | 0.1542 | Yes |
| 4 | CCL7 |  |  | 128 | 2.452 | 0.1982 | Yes |
| 5 | CCR2 |  |  | 219 | 2.188 | 0.2299 | Yes |
| 6 | CCL5 |  |  | 241 | 2.144 | 0.2705 | Yes |
| 7 | CCR5 |  |  | 244 | 2.139 | 0.3137 | Yes |
| 8 | PF4 |  |  | 290 | 2.044 | 0.3488 | Yes |
| 9 | SPN |  |  | 342 | 1.962 | 0.3814 | Yes |
| 10 | CXCL14 |  |  | 428 | 1.821 | 0.4064 | Yes |
| 11 | CCBP2 |  |  | 498 | 1.707 | 0.4313 | Yes |
| 12 | CXCR3 |  |  | 527 | 1.680 | 0.4614 | Yes |
| 13 | TGFB2 |  |  | 572 | 1.624 | 0.4882 | Yes |
| 14 | CCL2 |  |  | 875 | 1.330 | 0.4723 | Yes |
| 15 | CCL17 |  |  | 986 | 1.243 | 0.4820 | Yes |
| 16 | CCR3 |  |  | 1095 | 1.159 | 0.4902 | Yes |
| 17 | DOCK2 |  |  | 1191 | 1.098 | 0.4990 | Yes |
| 18 | CX3CL1 |  |  | 1442 | 0.942 | 0.4827 | No |
| 19 | ADCY8 |  |  | 1599 | 0.856 | 0.4780 | No |
| 20 | ITGB2 |  |  | 1794 | 0.753 | 0.4657 | No |
| 21 | RALBP1 |  |  | 1893 | 0.694 | 0.4659 | No |
| 22 | PLAU |  |  | 1938 | 0.671 | 0.4733 | No |
| 23 | CXCR4 |  |  | 2015 | 0.626 | 0.4753 | No |
| 24 | CXCL13 |  |  | 2207 | 0.528 | 0.4589 | No |
| 25 | PLAUR |  |  | 2391 | 0.447 | 0.4420 | No |
| 26 | CXCL9 |  |  | 2452 | 0.418 | 0.4420 | No |
| 27 | CCL11 |  |  | 2459 | 0.413 | 0.4495 | No |
| 28 | PPYR1 |  |  | 2545 | 0.372 | 0.4450 | No |
| 29 | CX3CR1 |  |  | 2643 | 0.326 | 0.4379 | No |
| 30 | CXCL12 |  |  | 2746 | 0.280 | 0.4291 | No |
| 31 | CDH13 |  |  | 2816 | 0.246 | 0.4243 | No |
| 32 | CCL24 |  |  | 2825 | 0.243 | 0.4281 | No |
| 33 | CCRL1 |  |  | 2863 | 0.234 | 0.4276 | No |
| 34 | NOVA1 |  |  | 3126 | 0.106 | 0.3926 | No |
| 35 | HPRT1 |  |  | 3192 | 0.074 | 0.3848 | No |
| 36 | OPRM1 |  |  | 3889 | -0.255 | 0.2913 | No |
| 37 | CCL25 |  |  | 4155 | -0.381 | 0.2614 | No |
| 38 | ZIC1 |  |  | 4215 | -0.414 | 0.2614 | No |
| 39 | MAPK1 |  |  | 4676 | -0.606 | 0.2085 | No |
| 40 | FGF2 |  |  | 4723 | -0.629 | 0.2147 | No |
| 41 | RALA |  |  | 5411 | -0.982 | 0.1372 | No |
| 42 | NPY |  |  | 5639 | -1.111 | 0.1275 | No |
| 43 | PIK3CB |  |  | 5683 | -1.143 | 0.1447 | No |
| 44 | MAP2K1 |  |  | 5962 | -1.304 | 0.1317 | No |
| 45 | KLK8 |  |  | 6075 | -1.396 | 0.1442 | No |
Table: GSEA details [plain text format]

  

Fig 2: BEHAVIOR: Random ES distribution      
 Gene set null distribution of ES for **BEHAVIOR**

  
