# Supplementary material for: Network-based integration of molecular and physiological data elucidates regulatory mechanisms underlying adaptation to high-fat diet
Source: Genes Nutr. 2015 May 28;10(4):22. doi: 10.1007/s12263-015-0470-6 (PMC4446272; doi:10.1007/s12263-015-0470-6)
Supplement: Supplementary file 4 — Supplementary material 4 (ZIP 6984 kb) [file 12263_2015_470_MOESM4_ESM.zip › HF LF 5 d GSEA result/CELL_SURFACE_RECEPTOR_LINKED_SIGNAL_TRANSDUCTION_GO_0007166.html]

Details for gene set CELL\_SURFACE\_RECEPTOR\_LINKED\_SIGNAL\_TRANSDUCTION\_GO\_0007166[GSEA]

|  || Dataset | comp\_HF5d-LF5d\_collapsed |
| Phenotype | NoPhenotypeAvailable |
| Upregulated in class | na\_pos |
| GeneSet | CELL\_SURFACE\_RECEPTOR\_LINKED\_SIGNAL\_TRANSDUCTION\_GO\_0007166 |
| Enrichment Score (ES) | 0.36782622 |
| Normalized Enrichment Score (NES) | 1.9148526 |
| Nominal p-value | 0.0 |
| FDR q-value | 0.019894985 |
| FWER p-Value | 0.19 |
Table: GSEA Results Summary

  

Fig 1: Enrichment plot: CELL\_SURFACE\_RECEPTOR\_LINKED\_SIGNAL\_TRANSDUCTION\_GO\_0007166      
 Profile of the Running ES Score & Positions of GeneSet Members on the Rank Ordered List

  

| PROBE | GENE SYMBOL | GENE\_TITLE | RANK IN GENE LIST | RANK METRIC SCORE | RUNNING ES | CORE ENRICHMENT || 1 | PYCARD |  |  | 28 | 3.173 | 0.0126 | Yes |
| 2 | ACVR1B |  |  | 75 | 2.707 | 0.0202 | Yes |
| 3 | APOE |  |  | 86 | 2.652 | 0.0327 | Yes |
| 4 | EPS15 |  |  | 87 | 2.645 | 0.0466 | Yes |
| 5 | BMPR1A |  |  | 117 | 2.499 | 0.0555 | Yes |
| 6 | IGSF6 |  |  | 118 | 2.497 | 0.0687 | Yes |
| 7 | RGS14 |  |  | 119 | 2.488 | 0.0818 | Yes |
| 8 | OSMR |  |  | 123 | 2.465 | 0.0943 | Yes |
| 9 | ADCY7 |  |  | 132 | 2.426 | 0.1059 | Yes |
| 10 | IL6ST |  |  | 179 | 2.286 | 0.1112 | Yes |
| 11 | AKAP12 |  |  | 192 | 2.251 | 0.1213 | Yes |
| 12 | PLCE1 |  |  | 195 | 2.243 | 0.1328 | Yes |
| 13 | CCR2 |  |  | 219 | 2.188 | 0.1410 | Yes |
| 14 | EGFR |  |  | 222 | 2.185 | 0.1522 | Yes |
| 15 | MDFIC |  |  | 226 | 2.178 | 0.1632 | Yes |
| 16 | CCR5 |  |  | 244 | 2.139 | 0.1720 | Yes |
| 17 | PF4 |  |  | 290 | 2.044 | 0.1763 | Yes |
| 18 | SMAD7 |  |  | 291 | 2.043 | 0.1870 | Yes |
| 19 | DOK2 |  |  | 322 | 1.992 | 0.1931 | Yes |
| 20 | LTB4R |  |  | 336 | 1.973 | 0.2016 | Yes |
| 21 | ADAMTS1 |  |  | 379 | 1.905 | 0.2056 | Yes |
| 22 | AVPR1A |  |  | 380 | 1.903 | 0.2156 | Yes |
| 23 | GAB1 |  |  | 388 | 1.891 | 0.2245 | Yes |
| 24 | IFNGR2 |  |  | 399 | 1.878 | 0.2329 | Yes |
| 25 | IRAK3 |  |  | 408 | 1.859 | 0.2415 | Yes |
| 26 | P2RY6 |  |  | 424 | 1.828 | 0.2490 | Yes |
| 27 | SOCS1 |  |  | 450 | 1.783 | 0.2547 | Yes |
| 28 | DOK1 |  |  | 455 | 1.778 | 0.2635 | Yes |
| 29 | FRS2 |  |  | 457 | 1.778 | 0.2727 | Yes |
| 30 | TRPV4 |  |  | 472 | 1.756 | 0.2799 | Yes |
| 31 | XPR1 |  |  | 488 | 1.714 | 0.2868 | Yes |
| 32 | CCBP2 |  |  | 498 | 1.707 | 0.2944 | Yes |
| 33 | CALM3 |  |  | 518 | 1.690 | 0.3006 | Yes |
| 34 | IL13RA1 |  |  | 534 | 1.670 | 0.3072 | Yes |
| 35 | GRB2 |  |  | 538 | 1.663 | 0.3155 | Yes |
| 36 | P2RY1 |  |  | 595 | 1.594 | 0.3158 | Yes |
| 37 | CALCA |  |  | 607 | 1.582 | 0.3225 | Yes |
| 38 | MARCO |  |  | 643 | 1.544 | 0.3255 | Yes |
| 39 | PIK3R1 |  |  | 666 | 1.525 | 0.3304 | Yes |
| 40 | LIFR |  |  | 692 | 1.505 | 0.3347 | Yes |
| 41 | CD24 |  |  | 804 | 1.398 | 0.3260 | Yes |
| 42 | FMOD |  |  | 841 | 1.362 | 0.3279 | Yes |
| 43 | SMAD1 |  |  | 844 | 1.357 | 0.3348 | Yes |
| 44 | PTPN6 |  |  | 859 | 1.343 | 0.3398 | Yes |
| 45 | CCL2 |  |  | 875 | 1.330 | 0.3446 | Yes |
| 46 | TNFRSF1A |  |  | 973 | 1.253 | 0.3372 | Yes |
| 47 | CCL17 |  |  | 986 | 1.243 | 0.3420 | Yes |
| 48 | HPGD |  |  | 1013 | 1.222 | 0.3446 | Yes |
| 49 | P2RY2 |  |  | 1037 | 1.204 | 0.3476 | Yes |
| 50 | GPSM2 |  |  | 1040 | 1.203 | 0.3537 | Yes |
| 51 | LEFTY1 |  |  | 1048 | 1.200 | 0.3590 | Yes |
| 52 | CCR3 |  |  | 1095 | 1.159 | 0.3584 | Yes |
| 53 | TGFA |  |  | 1152 | 1.124 | 0.3562 | Yes |
| 54 | IL7R |  |  | 1201 | 1.095 | 0.3550 | Yes |
| 55 | GPR65 |  |  | 1219 | 1.086 | 0.3583 | Yes |
| 56 | GNAI3 |  |  | 1299 | 1.027 | 0.3522 | Yes |
| 57 | TBXA2R |  |  | 1333 | 1.004 | 0.3527 | Yes |
| 58 | WASF2 |  |  | 1372 | 0.985 | 0.3524 | Yes |
| 59 | ADAM11 |  |  | 1382 | 0.977 | 0.3563 | Yes |
| 60 | GDF10 |  |  | 1392 | 0.971 | 0.3601 | Yes |
| 61 | SPHK1 |  |  | 1398 | 0.968 | 0.3644 | Yes |
| 62 | CX3CL1 |  |  | 1442 | 0.942 | 0.3632 | Yes |
| 63 | DTX1 |  |  | 1445 | 0.941 | 0.3678 | Yes |
| 64 | RGS5 |  |  | 1515 | 0.896 | 0.3626 | No |
| 65 | MPZL1 |  |  | 1563 | 0.874 | 0.3604 | No |
| 66 | BIRC2 |  |  | 1573 | 0.868 | 0.3636 | No |
| 67 | MBIP |  |  | 1617 | 0.847 | 0.3618 | No |
| 68 | INHBA |  |  | 1623 | 0.843 | 0.3656 | No |
| 69 | LY96 |  |  | 1707 | 0.800 | 0.3577 | No |
| 70 | GDF9 |  |  | 1808 | 0.746 | 0.3472 | No |
| 71 | ASGR2 |  |  | 1907 | 0.685 | 0.3366 | No |
| 72 | PXN |  |  | 1930 | 0.676 | 0.3370 | No |
| 73 | CD14 |  |  | 1931 | 0.676 | 0.3406 | No |
| 74 | CD47 |  |  | 1935 | 0.672 | 0.3437 | No |
| 75 | GPRC5C |  |  | 1982 | 0.645 | 0.3404 | No |
| 76 | DRD4 |  |  | 2012 | 0.627 | 0.3395 | No |
| 77 | CXCR4 |  |  | 2015 | 0.626 | 0.3425 | No |
| 78 | CD274 |  |  | 2025 | 0.619 | 0.3444 | No |
| 79 | KHDRBS1 |  |  | 2052 | 0.605 | 0.3439 | No |
| 80 | LAT |  |  | 2123 | 0.567 | 0.3367 | No |
| 81 | STC2 |  |  | 2271 | 0.498 | 0.3181 | No |
| 82 | GRB7 |  |  | 2278 | 0.495 | 0.3198 | No |
| 83 | GRIA3 |  |  | 2323 | 0.478 | 0.3160 | No |
| 84 | DOCK1 |  |  | 2376 | 0.454 | 0.3108 | No |
| 85 | CXCL9 |  |  | 2452 | 0.418 | 0.3022 | No |
| 86 | MERTK |  |  | 2496 | 0.399 | 0.2980 | No |
| 87 | PTHLH |  |  | 2519 | 0.387 | 0.2969 | No |
| 88 | PPYR1 |  |  | 2545 | 0.372 | 0.2952 | No |
| 89 | IL2RB |  |  | 2618 | 0.337 | 0.2866 | No |
| 90 | OPN3 |  |  | 2623 | 0.335 | 0.2878 | No |
| 91 | CX3CR1 |  |  | 2643 | 0.326 | 0.2867 | No |
| 92 | RGS19 |  |  | 2723 | 0.289 | 0.2768 | No |
| 93 | CXCL12 |  |  | 2746 | 0.280 | 0.2751 | No |
| 94 | RAMP1 |  |  | 2752 | 0.277 | 0.2758 | No |
| 95 | EDNRA |  |  | 2756 | 0.274 | 0.2769 | No |
| 96 | GABBR1 |  |  | 2763 | 0.269 | 0.2774 | No |
| 97 | CDH13 |  |  | 2816 | 0.246 | 0.2712 | No |
| 98 | CCRL1 |  |  | 2863 | 0.234 | 0.2657 | No |
| 99 | APOA1 |  |  | 2876 | 0.230 | 0.2652 | No |
| 100 | AGTR1 |  |  | 2926 | 0.205 | 0.2592 | No |
| 101 | IL27RA |  |  | 3019 | 0.156 | 0.2467 | No |
| 102 | ADRB1 |  |  | 3052 | 0.137 | 0.2428 | No |
| 103 | LANCL1 |  |  | 3109 | 0.111 | 0.2353 | No |
| 104 | VIPR1 |  |  | 3183 | 0.078 | 0.2251 | No |
| 105 | ABI1 |  |  | 3202 | 0.070 | 0.2229 | No |
| 106 | CAP2 |  |  | 3212 | 0.067 | 0.2220 | No |
| 107 | PDGFRA |  |  | 3217 | 0.064 | 0.2217 | No |
| 108 | CBL |  |  | 3249 | 0.050 | 0.2175 | No |
| 109 | PIK3CG |  |  | 3264 | 0.044 | 0.2157 | No |
| 110 | CD2 |  |  | 3313 | 0.021 | 0.2089 | No |
| 111 | FLT4 |  |  | 3317 | 0.020 | 0.2085 | No |
| 112 | CLEC1A |  |  | 3364 | -0.005 | 0.2019 | No |
| 113 | C3AR1 |  |  | 3413 | -0.022 | 0.1951 | No |
| 114 | GRK4 |  |  | 3701 | -0.159 | 0.1544 | No |
| 115 | TRAF6 |  |  | 3749 | -0.180 | 0.1486 | No |
| 116 | PRKD3 |  |  | 3814 | -0.217 | 0.1405 | No |
| 117 | OPRM1 |  |  | 3889 | -0.255 | 0.1311 | No |
| 118 | C3 |  |  | 3952 | -0.285 | 0.1236 | No |
| 119 | IFNAR2 |  |  | 3964 | -0.291 | 0.1236 | No |
| 120 | SOCS5 |  |  | 3979 | -0.299 | 0.1231 | No |
| 121 | PTK2 |  |  | 4045 | -0.327 | 0.1154 | No |
| 122 | EDNRB |  |  | 4095 | -0.352 | 0.1102 | No |
| 123 | HOMER2 |  |  | 4140 | -0.374 | 0.1058 | No |
| 124 | CCL25 |  |  | 4155 | -0.381 | 0.1058 | No |
| 125 | PSEN1 |  |  | 4159 | -0.382 | 0.1073 | No |
| 126 | ZIC1 |  |  | 4215 | -0.414 | 0.1016 | No |
| 127 | IKBKG |  |  | 4303 | -0.449 | 0.0913 | No |
| 128 | ABCA1 |  |  | 4350 | -0.467 | 0.0871 | No |
| 129 | EGF |  |  | 4373 | -0.476 | 0.0864 | No |
| 130 | EPGN |  |  | 4398 | -0.488 | 0.0855 | No |
| 131 | PTK2B |  |  | 4407 | -0.494 | 0.0870 | No |
| 132 | MTSS1 |  |  | 4411 | -0.495 | 0.0892 | No |
| 133 | CHRM4 |  |  | 4566 | -0.559 | 0.0698 | No |
| 134 | BSG |  |  | 4576 | -0.564 | 0.0715 | No |
| 135 | RGMB |  |  | 4615 | -0.578 | 0.0690 | No |
| 136 | TSHR |  |  | 4617 | -0.578 | 0.0719 | No |
| 137 | INHA |  |  | 4699 | -0.622 | 0.0635 | No |
| 138 | RGS16 |  |  | 4706 | -0.624 | 0.0659 | No |
| 139 | BIRC3 |  |  | 4726 | -0.631 | 0.0664 | No |
| 140 | NCSTN |  |  | 4772 | -0.655 | 0.0634 | No |
| 141 | GRK5 |  |  | 4775 | -0.655 | 0.0665 | No |
| 142 | GNB1 |  |  | 4785 | -0.660 | 0.0687 | No |
| 143 | GUCA1B |  |  | 4822 | -0.678 | 0.0671 | No |
| 144 | ERBB2 |  |  | 4844 | -0.690 | 0.0677 | No |
| 145 | MAP3K7 |  |  | 4858 | -0.695 | 0.0694 | No |
| 146 | STUB1 |  |  | 4954 | -0.743 | 0.0596 | No |
| 147 | GUCY2C |  |  | 4982 | -0.755 | 0.0597 | No |
| 148 | MTNR1B |  |  | 5039 | -0.780 | 0.0557 | No |
| 149 | SLA2 |  |  | 5132 | -0.822 | 0.0467 | No |
| 150 | PARD3 |  |  | 5135 | -0.822 | 0.0507 | No |
| 151 | FIBP |  |  | 5266 | -0.896 | 0.0366 | No |
| 152 | TGFB1 |  |  | 5288 | -0.910 | 0.0383 | No |
| 153 | GRK6 |  |  | 5314 | -0.924 | 0.0396 | No |
| 154 | GNA15 |  |  | 5439 | -1.001 | 0.0269 | No |
| 155 | KDR |  |  | 5455 | -1.012 | 0.0301 | No |
| 156 | TBL3 |  |  | 5465 | -1.018 | 0.0341 | No |
| 157 | GPR4 |  |  | 5583 | -1.081 | 0.0229 | No |
| 158 | RASD1 |  |  | 5618 | -1.099 | 0.0237 | No |
| 159 | NPY |  |  | 5639 | -1.111 | 0.0267 | No |
| 160 | PIK3CB |  |  | 5683 | -1.143 | 0.0265 | No |
| 161 | FOXC2 |  |  | 5726 | -1.167 | 0.0265 | No |
| 162 | ACR |  |  | 5897 | -1.263 | 0.0086 | No |
| 163 | PTGER3 |  |  | 5977 | -1.313 | 0.0040 | No |
| 164 | IL31RA |  |  | 6036 | -1.355 | 0.0028 | No |
| 165 | IRS1 |  |  | 6154 | -1.464 | -0.0064 | No |
| 166 | GABRR1 |  |  | 6170 | -1.471 | -0.0009 | No |
| 167 | PTGER2 |  |  | 6209 | -1.518 | 0.0016 | No |
| 168 | PRLR |  |  | 6258 | -1.558 | 0.0028 | No |
| 169 | CDKN1C |  |  | 6285 | -1.586 | 0.0074 | No |
| 170 | NPFF |  |  | 6380 | -1.682 | 0.0027 | No |
| 171 | HRH3 |  |  | 6383 | -1.684 | 0.0112 | No |
| 172 | RRAGA |  |  | 6424 | -1.732 | 0.0145 | No |
| 173 | HOMER3 |  |  | 6475 | -1.795 | 0.0168 | No |
| 174 | UBE2N |  |  | 6548 | -1.884 | 0.0162 | No |
| 175 | PTGER4 |  |  | 6589 | -1.946 | 0.0207 | No |
| 176 | SMURF1 |  |  | 6592 | -1.952 | 0.0307 | No |
| 177 | MKNK2 |  |  | 6599 | -1.971 | 0.0402 | No |
| 178 | RGS2 |  |  | 6917 | -2.731 | 0.0087 | No |
| 179 | CIDEA |  |  | 6981 | -3.114 | 0.0159 | No |
Table: GSEA details [plain text format]

  

Fig 2: CELL\_SURFACE\_RECEPTOR\_LINKED\_SIGNAL\_TRANSDUCTION\_GO\_0007166: Random ES distribution      
 Gene set null distribution of ES for **CELL\_SURFACE\_RECEPTOR\_LINKED\_SIGNAL\_TRANSDUCTION\_GO\_0007166**

  
