# Supplementary material for: Network-based integration of molecular and physiological data elucidates regulatory mechanisms underlying adaptation to high-fat diet
Source: Genes Nutr. 2015 May 28;10(4):22. doi: 10.1007/s12263-015-0470-6 (PMC4446272; doi:10.1007/s12263-015-0470-6)
Supplement: Supplementary file 4 — Supplementary material 4 (ZIP 6984 kb) [file 12263_2015_470_MOESM4_ESM.zip › HF LF 5 d GSEA result/CELLULAR_CATION_HOMEOSTASIS.html]

Details for gene set CELLULAR\_CATION\_HOMEOSTASIS[GSEA]

|  || Dataset | comp\_HF5d-LF5d\_collapsed |
| Phenotype | NoPhenotypeAvailable |
| Upregulated in class | na\_pos |
| GeneSet | CELLULAR\_CATION\_HOMEOSTASIS |
| Enrichment Score (ES) | 0.5816161 |
| Normalized Enrichment Score (NES) | 2.2040818 |
| Nominal p-value | 0.0 |
| FDR q-value | 0.003235561 |
| FWER p-Value | 0.006 |
Table: GSEA Results Summary

  

Fig 1: Enrichment plot: CELLULAR\_CATION\_HOMEOSTASIS      
 Profile of the Running ES Score & Positions of GeneSet Members on the Rank Ordered List

  

| PROBE | GENE SYMBOL | GENE\_TITLE | RANK IN GENE LIST | RANK METRIC SCORE | RUNNING ES | CORE ENRICHMENT || 1 | MT2A |  |  | 29 | 3.154 | 0.0712 | Yes |
| 2 | CCL7 |  |  | 128 | 2.452 | 0.1159 | Yes |
| 3 | PLCE1 |  |  | 195 | 2.243 | 0.1601 | Yes |
| 4 | CCR2 |  |  | 219 | 2.188 | 0.2091 | Yes |
| 5 | CCL5 |  |  | 241 | 2.144 | 0.2573 | Yes |
| 6 | CCR5 |  |  | 244 | 2.139 | 0.3081 | Yes |
| 7 | BCL2 |  |  | 296 | 2.039 | 0.3496 | Yes |
| 8 | AVPR1A |  |  | 380 | 1.903 | 0.3833 | Yes |
| 9 | TRPV4 |  |  | 472 | 1.756 | 0.4123 | Yes |
| 10 | SRI |  |  | 509 | 1.698 | 0.4478 | Yes |
| 11 | CXCR3 |  |  | 527 | 1.680 | 0.4855 | Yes |
| 12 | CALCA |  |  | 607 | 1.582 | 0.5121 | Yes |
| 13 | CD24 |  |  | 804 | 1.398 | 0.5177 | Yes |
| 14 | ATP7A |  |  | 847 | 1.355 | 0.5441 | Yes |
| 15 | CCL2 |  |  | 875 | 1.330 | 0.5721 | Yes |
| 16 | CCR3 |  |  | 1095 | 1.159 | 0.5687 | Yes |
| 17 | ATP1A1 |  |  | 1190 | 1.098 | 0.5816 | Yes |
| 18 | CXCR4 |  |  | 2015 | 0.626 | 0.4798 | No |
| 19 | CXCL13 |  |  | 2207 | 0.528 | 0.4653 | No |
| 20 | MYC |  |  | 2260 | 0.503 | 0.4700 | No |
| 21 | CCL11 |  |  | 2459 | 0.413 | 0.4518 | No |
| 22 | CLN5 |  |  | 2503 | 0.396 | 0.4552 | No |
| 23 | CXCL12 |  |  | 2746 | 0.280 | 0.4275 | No |
| 24 | EDNRA |  |  | 2756 | 0.274 | 0.4328 | No |
| 25 | CD52 |  |  | 2866 | 0.233 | 0.4229 | No |
| 26 | AGTR1 |  |  | 2926 | 0.205 | 0.4194 | No |
| 27 | C3AR1 |  |  | 3413 | -0.022 | 0.3511 | No |
| 28 | RGN |  |  | 4362 | -0.471 | 0.2280 | No |
| 29 | CALR |  |  | 4460 | -0.514 | 0.2266 | No |
| 30 | GCM2 |  |  | 4966 | -0.747 | 0.1728 | No |
| 31 | FTH1 |  |  | 5166 | -0.838 | 0.1647 | No |
| 32 | ATP1A2 |  |  | 5207 | -0.864 | 0.1796 | No |
| 33 | GNA15 |  |  | 5439 | -1.001 | 0.1708 | No |
| 34 | SLC40A1 |  |  | 5855 | -1.235 | 0.1415 | No |
| 35 | CLN6 |  |  | 6085 | -1.403 | 0.1426 | No |
Table: GSEA details [plain text format]

  

Fig 2: CELLULAR\_CATION\_HOMEOSTASIS: Random ES distribution      
 Gene set null distribution of ES for **CELLULAR\_CATION\_HOMEOSTASIS**

  
