# Supplementary material for: Network-based integration of molecular and physiological data elucidates regulatory mechanisms underlying adaptation to high-fat diet
Source: Genes Nutr. 2015 May 28;10(4):22. doi: 10.1007/s12263-015-0470-6 (PMC4446272; doi:10.1007/s12263-015-0470-6)
Supplement: Supplementary file 4 — Supplementary material 4 (ZIP 6984 kb) [file 12263_2015_470_MOESM4_ESM.zip › HF LF 5 d GSEA result/CELLULAR_DEFENSE_RESPONSE.html]

Details for gene set CELLULAR\_DEFENSE\_RESPONSE[GSEA]

|  || Dataset | comp\_HF5d-LF5d\_collapsed |
| Phenotype | NoPhenotypeAvailable |
| Upregulated in class | na\_pos |
| GeneSet | CELLULAR\_DEFENSE\_RESPONSE |
| Enrichment Score (ES) | 0.6793996 |
| Normalized Enrichment Score (NES) | 2.1436307 |
| Nominal p-value | 0.0 |
| FDR q-value | 0.0045275497 |
| FWER p-Value | 0.014 |
Table: GSEA Results Summary

  

Fig 1: Enrichment plot: CELLULAR\_DEFENSE\_RESPONSE      
 Profile of the Running ES Score & Positions of GeneSet Members on the Rank Ordered List

  

| PROBE | GENE SYMBOL | GENE\_TITLE | RANK IN GENE LIST | RANK METRIC SCORE | RUNNING ES | CORE ENRICHMENT || 1 | LBP |  |  | 43 | 2.939 | 0.1355 | Yes |
| 2 | CCR2 |  |  | 219 | 2.188 | 0.2161 | Yes |
| 3 | CCL5 |  |  | 241 | 2.144 | 0.3163 | Yes |
| 4 | CCR5 |  |  | 244 | 2.139 | 0.4191 | Yes |
| 5 | TYROBP |  |  | 263 | 2.103 | 0.5178 | Yes |
| 6 | SPN |  |  | 342 | 1.962 | 0.6012 | Yes |
| 7 | ZNF148 |  |  | 542 | 1.658 | 0.6529 | Yes |
| 8 | CCR3 |  |  | 1095 | 1.159 | 0.6307 | Yes |
| 9 | VEZF1 |  |  | 1136 | 1.129 | 0.6794 | Yes |
| 10 | LY96 |  |  | 1707 | 0.800 | 0.6373 | No |
| 11 | TCIRG1 |  |  | 2296 | 0.488 | 0.5777 | No |
| 12 | CXCL9 |  |  | 2452 | 0.418 | 0.5759 | No |
| 13 | CX3CR1 |  |  | 2643 | 0.326 | 0.5647 | No |
| 14 | CD5L |  |  | 3055 | 0.136 | 0.5132 | No |
| 15 | ITGB1 |  |  | 3061 | 0.134 | 0.5190 | No |
| 16 | LGALS3BP |  |  | 3288 | 0.032 | 0.4885 | No |
| 17 | FAIM3 |  |  | 3610 | -0.116 | 0.4487 | No |
| 18 | BECN1 |  |  | 4003 | -0.308 | 0.4082 | No |
| 19 | LYST |  |  | 4635 | -0.587 | 0.3472 | No |
Table: GSEA details [plain text format]

  

Fig 2: CELLULAR\_DEFENSE\_RESPONSE: Random ES distribution      
 Gene set null distribution of ES for **CELLULAR\_DEFENSE\_RESPONSE**

  
