# Supplementary material for: Network-based integration of molecular and physiological data elucidates regulatory mechanisms underlying adaptation to high-fat diet
Source: Genes Nutr. 2015 May 28;10(4):22. doi: 10.1007/s12263-015-0470-6 (PMC4446272; doi:10.1007/s12263-015-0470-6)
Supplement: Supplementary file 4 — Supplementary material 4 (ZIP 6984 kb) [file 12263_2015_470_MOESM4_ESM.zip › HF LF 5 d GSEA result/CHROMATIN_MODIFICATION.html]

Details for gene set CHROMATIN\_MODIFICATION[GSEA]

|  || Dataset | comp\_HF5d-LF5d\_collapsed |
| Phenotype | NoPhenotypeAvailable |
| Upregulated in class | na\_neg |
| GeneSet | CHROMATIN\_MODIFICATION |
| Enrichment Score (ES) | -0.6145132 |
| Normalized Enrichment Score (NES) | -1.755965 |
| Nominal p-value | 0.009819968 |
| FDR q-value | 0.035704974 |
| FWER p-Value | 0.667 |
Table: GSEA Results Summary

  

Fig 1: Enrichment plot: CHROMATIN\_MODIFICATION      
 Profile of the Running ES Score & Positions of GeneSet Members on the Rank Ordered List

  

| PROBE | GENE SYMBOL | GENE\_TITLE | RANK IN GENE LIST | RANK METRIC SCORE | RUNNING ES | CORE ENRICHMENT || 1 | HDAC4 |  |  | 1621 | 0.844 | -0.1870 | No |
| 2 | NSD1 |  |  | 3051 | 0.138 | -0.3821 | No |
| 3 | RBBP4 |  |  | 4019 | -0.316 | -0.5030 | No |
| 4 | HDAC10 |  |  | 4282 | -0.440 | -0.5182 | No |
| 5 | RBM14 |  |  | 4558 | -0.556 | -0.5293 | No |
| 6 | HMGA1 |  |  | 4852 | -0.694 | -0.5362 | No |
| 7 | SIRT4 |  |  | 4853 | -0.694 | -0.5016 | No |
| 8 | SIRT2 |  |  | 5653 | -1.122 | -0.5586 | Yes |
| 9 | PPARGC1A |  |  | 5735 | -1.171 | -0.5117 | Yes |
| 10 | PHB |  |  | 5961 | -1.303 | -0.4786 | Yes |
| 11 | TNP1 |  |  | 6052 | -1.368 | -0.4232 | Yes |
| 12 | ACTL6A |  |  | 6359 | -1.654 | -0.3841 | Yes |
| 13 | HDAC2 |  |  | 6366 | -1.659 | -0.3022 | Yes |
| 14 | UBE2N |  |  | 6548 | -1.884 | -0.2340 | Yes |
| 15 | SYCP3 |  |  | 6923 | -2.753 | -0.1497 | Yes |
| 16 | SIRT5 |  |  | 7016 | -3.479 | 0.0106 | Yes |
Table: GSEA details [plain text format]

  

Fig 2: CHROMATIN\_MODIFICATION: Random ES distribution      
 Gene set null distribution of ES for **CHROMATIN\_MODIFICATION**

  
