# Supplementary material for: Network-based integration of molecular and physiological data elucidates regulatory mechanisms underlying adaptation to high-fat diet
Source: Genes Nutr. 2015 May 28;10(4):22. doi: 10.1007/s12263-015-0470-6 (PMC4446272; doi:10.1007/s12263-015-0470-6)
Supplement: Supplementary file 4 — Supplementary material 4 (ZIP 6984 kb) [file 12263_2015_470_MOESM4_ESM.zip › HF LF 5 d GSEA result/COFACTOR_METABOLIC_PROCESS.html]

Details for gene set COFACTOR\_METABOLIC\_PROCESS[GSEA]

|  || Dataset | comp\_HF5d-LF5d\_collapsed |
| Phenotype | NoPhenotypeAvailable |
| Upregulated in class | na\_neg |
| GeneSet | COFACTOR\_METABOLIC\_PROCESS |
| Enrichment Score (ES) | -0.64710397 |
| Normalized Enrichment Score (NES) | -1.9156325 |
| Nominal p-value | 0.0033557047 |
| FDR q-value | 0.0061898716 |
| FWER p-Value | 0.159 |
Table: GSEA Results Summary

  

Fig 1: Enrichment plot: COFACTOR\_METABOLIC\_PROCESS      
 Profile of the Running ES Score & Positions of GeneSet Members on the Rank Ordered List

  

| PROBE | GENE SYMBOL | GENE\_TITLE | RANK IN GENE LIST | RANK METRIC SCORE | RUNNING ES | CORE ENRICHMENT || 1 | GPX1 |  |  | 1729 | 0.790 | -0.2133 | No |
| 2 | CTNS |  |  | 2949 | 0.191 | -0.3781 | No |
| 3 | BLVRA |  |  | 3150 | 0.093 | -0.4027 | No |
| 4 | CPOX |  |  | 3666 | -0.145 | -0.4698 | No |
| 5 | MTHFD2 |  |  | 4595 | -0.571 | -0.5785 | No |
| 6 | NFS1 |  |  | 4999 | -0.762 | -0.6055 | No |
| 7 | MLYCD |  |  | 5042 | -0.781 | -0.5807 | No |
| 8 | PPOX |  |  | 5513 | -1.046 | -0.6059 | Yes |
| 9 | COX10 |  |  | 5667 | -1.133 | -0.5829 | Yes |
| 10 | GCLC |  |  | 5823 | -1.219 | -0.5568 | Yes |
| 11 | COX15 |  |  | 5952 | -1.296 | -0.5239 | Yes |
| 12 | MOCS2 |  |  | 6142 | -1.454 | -0.4934 | Yes |
| 13 | SDHD |  |  | 6419 | -1.723 | -0.4645 | Yes |
| 14 | ALAS2 |  |  | 6802 | -2.335 | -0.4266 | Yes |
| 15 | COQ7 |  |  | 6860 | -2.500 | -0.3362 | Yes |
| 16 | GCLM |  |  | 6960 | -2.955 | -0.2339 | Yes |
| 17 | ACO2 |  |  | 6983 | -3.130 | -0.1137 | Yes |
| 18 | MOCOS |  |  | 6999 | -3.272 | 0.0130 | Yes |
Table: GSEA details [plain text format]

  

Fig 2: COFACTOR\_METABOLIC\_PROCESS: Random ES distribution      
 Gene set null distribution of ES for **COFACTOR\_METABOLIC\_PROCESS**

  
