# Supplementary material for: Network-based integration of molecular and physiological data elucidates regulatory mechanisms underlying adaptation to high-fat diet
Source: Genes Nutr. 2015 May 28;10(4):22. doi: 10.1007/s12263-015-0470-6 (PMC4446272; doi:10.1007/s12263-015-0470-6)
Supplement: Supplementary file 4 — Supplementary material 4 (ZIP 6984 kb) [file 12263_2015_470_MOESM4_ESM.zip › HF LF 5 d GSEA result/DEFENSE_RESPONSE.html]

Details for gene set DEFENSE\_RESPONSE[GSEA]

|  || Dataset | comp\_HF5d-LF5d\_collapsed |
| Phenotype | NoPhenotypeAvailable |
| Upregulated in class | na\_pos |
| GeneSet | DEFENSE\_RESPONSE |
| Enrichment Score (ES) | 0.44827512 |
| Normalized Enrichment Score (NES) | 2.015309 |
| Nominal p-value | 0.0 |
| FDR q-value | 0.012106775 |
| FWER p-Value | 0.071 |
Table: GSEA Results Summary

  

Fig 1: Enrichment plot: DEFENSE\_RESPONSE      
 Profile of the Running ES Score & Positions of GeneSet Members on the Rank Ordered List

  

| PROBE | GENE SYMBOL | GENE\_TITLE | RANK IN GENE LIST | RANK METRIC SCORE | RUNNING ES | CORE ENRICHMENT || 1 | LBP |  |  | 43 | 2.939 | 0.0359 | Yes |
| 2 | FOS |  |  | 207 | 2.218 | 0.0443 | Yes |
| 3 | CCR2 |  |  | 219 | 2.188 | 0.0740 | Yes |
| 4 | CCL5 |  |  | 241 | 2.144 | 0.1016 | Yes |
| 5 | CCR5 |  |  | 244 | 2.139 | 0.1319 | Yes |
| 6 | TYROBP |  |  | 263 | 2.103 | 0.1594 | Yes |
| 7 | ALOX5AP |  |  | 273 | 2.083 | 0.1878 | Yes |
| 8 | BCL2 |  |  | 296 | 2.039 | 0.2138 | Yes |
| 9 | LTB4R |  |  | 336 | 1.973 | 0.2365 | Yes |
| 10 | C2 |  |  | 340 | 1.966 | 0.2641 | Yes |
| 11 | SPN |  |  | 342 | 1.962 | 0.2920 | Yes |
| 12 | STAB1 |  |  | 395 | 1.881 | 0.3115 | Yes |
| 13 | AIF1 |  |  | 528 | 1.679 | 0.3166 | Yes |
| 14 | ZNF148 |  |  | 542 | 1.658 | 0.3385 | Yes |
| 15 | KCNN4 |  |  | 565 | 1.633 | 0.3586 | Yes |
| 16 | TLR6 |  |  | 679 | 1.511 | 0.3641 | Yes |
| 17 | S100A8 |  |  | 817 | 1.384 | 0.3644 | Yes |
| 18 | TNFRSF1A |  |  | 973 | 1.253 | 0.3602 | Yes |
| 19 | CCR3 |  |  | 1095 | 1.159 | 0.3595 | Yes |
| 20 | VEZF1 |  |  | 1136 | 1.129 | 0.3699 | Yes |
| 21 | PTPRCAP |  |  | 1224 | 1.082 | 0.3730 | Yes |
| 22 | CD81 |  |  | 1230 | 1.078 | 0.3877 | Yes |
| 23 | CX3CL1 |  |  | 1442 | 0.942 | 0.3711 | Yes |
| 24 | PLA2G2D |  |  | 1474 | 0.920 | 0.3798 | Yes |
| 25 | MEFV |  |  | 1521 | 0.893 | 0.3860 | Yes |
| 26 | HDAC4 |  |  | 1621 | 0.844 | 0.3840 | Yes |
| 27 | INHBA |  |  | 1623 | 0.843 | 0.3959 | Yes |
| 28 | AOC3 |  |  | 1640 | 0.835 | 0.4055 | Yes |
| 29 | IL28RA |  |  | 1660 | 0.824 | 0.4146 | Yes |
| 30 | COLEC12 |  |  | 1694 | 0.806 | 0.4214 | Yes |
| 31 | CCL4 |  |  | 1696 | 0.805 | 0.4327 | Yes |
| 32 | LY96 |  |  | 1707 | 0.800 | 0.4427 | Yes |
| 33 | BCL10 |  |  | 1824 | 0.734 | 0.4367 | Yes |
| 34 | RSAD2 |  |  | 1876 | 0.702 | 0.4394 | Yes |
| 35 | AHSG |  |  | 1908 | 0.685 | 0.4448 | Yes |
| 36 | F11R |  |  | 1952 | 0.663 | 0.4482 | Yes |
| 37 | CXCR4 |  |  | 2015 | 0.626 | 0.4483 | Yes |
| 38 | WAS |  |  | 2103 | 0.575 | 0.4441 | No |
| 39 | CEBPG |  |  | 2231 | 0.512 | 0.4333 | No |
| 40 | CD83 |  |  | 2243 | 0.507 | 0.4390 | No |
| 41 | ELF3 |  |  | 2261 | 0.503 | 0.4438 | No |
| 42 | TCIRG1 |  |  | 2296 | 0.488 | 0.4459 | No |
| 43 | NFRKB |  |  | 2330 | 0.476 | 0.4480 | No |
| 44 | CXCL9 |  |  | 2452 | 0.418 | 0.4367 | No |
| 45 | CCL11 |  |  | 2459 | 0.413 | 0.4418 | No |
| 46 | CX3CR1 |  |  | 2643 | 0.326 | 0.4203 | No |
| 47 | AOX1 |  |  | 2693 | 0.304 | 0.4177 | No |
| 48 | CCL24 |  |  | 2825 | 0.243 | 0.4025 | No |
| 49 | CD5L |  |  | 3055 | 0.136 | 0.3719 | No |
| 50 | ITGB1 |  |  | 3061 | 0.134 | 0.3731 | No |
| 51 | TIAL1 |  |  | 3064 | 0.134 | 0.3747 | No |
| 52 | NFATC4 |  |  | 3166 | 0.084 | 0.3615 | No |
| 53 | RAC1 |  |  | 3275 | 0.037 | 0.3466 | No |
| 54 | LGALS3BP |  |  | 3288 | 0.032 | 0.3454 | No |
| 55 | RNASE6 |  |  | 3342 | 0.009 | 0.3380 | No |
| 56 | CLEC1A |  |  | 3364 | -0.005 | 0.3351 | No |
| 57 | C3AR1 |  |  | 3413 | -0.022 | 0.3285 | No |
| 58 | MST1R |  |  | 3566 | -0.092 | 0.3082 | No |
| 59 | FAIM3 |  |  | 3610 | -0.116 | 0.3037 | No |
| 60 | PARP4 |  |  | 3757 | -0.185 | 0.2856 | No |
| 61 | BECN1 |  |  | 4003 | -0.308 | 0.2551 | No |
| 62 | ALOX15 |  |  | 4011 | -0.312 | 0.2585 | No |
| 63 | CAMLG |  |  | 4270 | -0.434 | 0.2280 | No |
| 64 | WFDC12 |  |  | 4450 | -0.510 | 0.2098 | No |
| 65 | ABCF1 |  |  | 4489 | -0.528 | 0.2119 | No |
| 66 | NFATC3 |  |  | 4518 | -0.541 | 0.2156 | No |
| 67 | LYST |  |  | 4635 | -0.587 | 0.2075 | No |
| 68 | NFX1 |  |  | 4712 | -0.625 | 0.2056 | No |
| 69 | MX2 |  |  | 5211 | -0.867 | 0.1470 | No |
| 70 | TGFB1 |  |  | 5288 | -0.910 | 0.1492 | No |
| 71 | ORM2 |  |  | 6678 | -2.093 | -0.0188 | No |
| 72 | ORM1 |  |  | 6710 | -2.152 | 0.0075 | No |
| 73 | CDO1 |  |  | 6998 | -3.263 | 0.0132 | No |
Table: GSEA details [plain text format]

  

Fig 2: DEFENSE\_RESPONSE: Random ES distribution      
 Gene set null distribution of ES for **DEFENSE\_RESPONSE**

  
