# Supplementary material for: Network-based integration of molecular and physiological data elucidates regulatory mechanisms underlying adaptation to high-fat diet
Source: Genes Nutr. 2015 May 28;10(4):22. doi: 10.1007/s12263-015-0470-6 (PMC4446272; doi:10.1007/s12263-015-0470-6)
Supplement: Supplementary file 4 — Supplementary material 4 (ZIP 6984 kb) [file 12263_2015_470_MOESM4_ESM.zip › HF LF 5 d GSEA result/ELECTRON_CARRIER_ACTIVITY.html]

Details for gene set ELECTRON\_CARRIER\_ACTIVITY[GSEA]

|  || Dataset | comp\_HF5d-LF5d\_collapsed |
| Phenotype | NoPhenotypeAvailable |
| Upregulated in class | na\_neg |
| GeneSet | ELECTRON\_CARRIER\_ACTIVITY |
| Enrichment Score (ES) | -0.5973271 |
| Normalized Enrichment Score (NES) | -1.936742 |
| Nominal p-value | 0.0033112583 |
| FDR q-value | 0.0055094534 |
| FWER p-Value | 0.126 |
Table: GSEA Results Summary

  

Fig 1: Enrichment plot: ELECTRON\_CARRIER\_ACTIVITY      
 Profile of the Running ES Score & Positions of GeneSet Members on the Rank Ordered List

  

| PROBE | GENE SYMBOL | GENE\_TITLE | RANK IN GENE LIST | RANK METRIC SCORE | RUNNING ES | CORE ENRICHMENT || 1 | P4HA2 |  |  | 7 | 4.223 | 0.0793 | No |
| 2 | CYBA |  |  | 103 | 2.556 | 0.1145 | No |
| 3 | ASPH |  |  | 441 | 1.799 | 0.1010 | No |
| 4 | LOXL2 |  |  | 475 | 1.742 | 0.1295 | No |
| 5 | TXNRD1 |  |  | 573 | 1.623 | 0.1466 | No |
| 6 | ALDH1A2 |  |  | 822 | 1.377 | 0.1377 | No |
| 7 | MAOB |  |  | 2556 | 0.367 | -0.1006 | No |
| 8 | DEGS1 |  |  | 2735 | 0.283 | -0.1204 | No |
| 9 | HMOX2 |  |  | 3518 | -0.073 | -0.2297 | No |
| 10 | NQO1 |  |  | 3537 | -0.079 | -0.2307 | No |
| 11 | ME2 |  |  | 3778 | -0.199 | -0.2609 | No |
| 12 | PHYH |  |  | 4017 | -0.315 | -0.2886 | No |
| 13 | FDXR |  |  | 4308 | -0.451 | -0.3211 | No |
| 14 | TSTA3 |  |  | 5736 | -1.172 | -0.5008 | No |
| 15 | SDHD |  |  | 6419 | -1.723 | -0.5645 | Yes |
| 16 | NDUFA9 |  |  | 6519 | -1.848 | -0.5434 | Yes |
| 17 | COX11 |  |  | 6569 | -1.914 | -0.5139 | Yes |
| 18 | NDUFS2 |  |  | 6777 | -2.265 | -0.5001 | Yes |
| 19 | NDUFS4 |  |  | 6792 | -2.295 | -0.4585 | Yes |
| 20 | CYCS |  |  | 6844 | -2.437 | -0.4193 | Yes |
| 21 | ALDH2 |  |  | 6865 | -2.528 | -0.3741 | Yes |
| 22 | IDH3B |  |  | 6930 | -2.804 | -0.3298 | Yes |
| 23 | CYC1 |  |  | 6975 | -3.040 | -0.2782 | Yes |
| 24 | DLD |  |  | 6996 | -3.249 | -0.2192 | Yes |
| 25 | COX5A |  |  | 7038 | -3.698 | -0.1547 | Yes |
| 26 | ALDH4A1 |  |  | 7056 | -4.141 | -0.0783 | Yes |
| 27 | NDUFS1 |  |  | 7068 | -4.370 | 0.0033 | Yes |
Table: GSEA details [plain text format]

  

Fig 2: ELECTRON\_CARRIER\_ACTIVITY: Random ES distribution      
 Gene set null distribution of ES for **ELECTRON\_CARRIER\_ACTIVITY**
