# Supplementary material for: Network-based integration of molecular and physiological data elucidates regulatory mechanisms underlying adaptation to high-fat diet
Source: Genes Nutr. 2015 May 28;10(4):22. doi: 10.1007/s12263-015-0470-6 (PMC4446272; doi:10.1007/s12263-015-0470-6)
Supplement: Supplementary file 4 — Supplementary material 4 (ZIP 6984 kb) [file 12263_2015_470_MOESM4_ESM.zip › HF LF 5 d GSEA result/ENVELOPE.html]

Details for gene set ENVELOPE[GSEA]

|  || Dataset | comp\_HF5d-LF5d\_collapsed |
| Phenotype | NoPhenotypeAvailable |
| Upregulated in class | na\_neg |
| GeneSet | ENVELOPE |
| Enrichment Score (ES) | -0.5325305 |
| Normalized Enrichment Score (NES) | -2.1480882 |
| Nominal p-value | 0.0 |
| FDR q-value | 5.201839E-4 |
| FWER p-Value | 0.008 |
Table: GSEA Results Summary

  

Fig 1: Enrichment plot: ENVELOPE      
 Profile of the Running ES Score & Positions of GeneSet Members on the Rank Ordered List

  

| PROBE | GENE SYMBOL | GENE\_TITLE | RANK IN GENE LIST | RANK METRIC SCORE | RUNNING ES | CORE ENRICHMENT || 1 | MCL1 |  |  | 17 | 3.532 | 0.0326 | No |
| 2 | BCL2 |  |  | 296 | 2.039 | 0.0132 | No |
| 3 | RAB11FIP5 |  |  | 502 | 1.705 | 0.0008 | No |
| 4 | SCRN1 |  |  | 736 | 1.466 | -0.0178 | No |
| 5 | NUP160 |  |  | 750 | 1.450 | -0.0053 | No |
| 6 | NUP153 |  |  | 1025 | 1.216 | -0.0323 | No |
| 7 | LBR |  |  | 1127 | 1.134 | -0.0355 | No |
| 8 | NUP98 |  |  | 1197 | 1.097 | -0.0345 | No |
| 9 | XPO1 |  |  | 1327 | 1.006 | -0.0429 | No |
| 10 | POLA1 |  |  | 1700 | 0.804 | -0.0879 | No |
| 11 | KPNB1 |  |  | 2011 | 0.628 | -0.1259 | No |
| 12 | EPC1 |  |  | 2042 | 0.608 | -0.1241 | No |
| 13 | GATM |  |  | 2393 | 0.447 | -0.1696 | No |
| 14 | MAOB |  |  | 2556 | 0.367 | -0.1891 | No |
| 15 | RANBP2 |  |  | 2943 | 0.194 | -0.2421 | No |
| 16 | IPO7 |  |  | 3101 | 0.114 | -0.2634 | No |
| 17 | NUTF2 |  |  | 3187 | 0.076 | -0.2747 | No |
| 18 | MPV17 |  |  | 3219 | 0.064 | -0.2785 | No |
| 19 | DDX19B |  |  | 3588 | -0.107 | -0.3299 | No |
| 20 | NUP133 |  |  | 3750 | -0.180 | -0.3511 | No |
| 21 | NPC1 |  |  | 3839 | -0.231 | -0.3613 | No |
| 22 | CASP7 |  |  | 3857 | -0.242 | -0.3614 | No |
| 23 | COX6B2 |  |  | 3909 | -0.267 | -0.3660 | No |
| 24 | NRM |  |  | 3993 | -0.305 | -0.3748 | No |
| 25 | HTATIP2 |  |  | 4020 | -0.317 | -0.3754 | No |
| 26 | PSEN1 |  |  | 4159 | -0.382 | -0.3912 | No |
| 27 | KPNA3 |  |  | 4248 | -0.427 | -0.3995 | No |
| 28 | OXA1L |  |  | 4256 | -0.429 | -0.3963 | No |
| 29 | RHOT2 |  |  | 4358 | -0.470 | -0.4060 | No |
| 30 | MATR3 |  |  | 4499 | -0.533 | -0.4207 | No |
| 31 | TIMM17A |  |  | 4569 | -0.561 | -0.4250 | No |
| 32 | MRPL19 |  |  | 4689 | -0.611 | -0.4359 | No |
| 33 | NUP50 |  |  | 5085 | -0.802 | -0.4842 | No |
| 34 | ATP5E |  |  | 5386 | -0.967 | -0.5174 | No |
| 35 | RANGAP1 |  |  | 5476 | -1.024 | -0.5199 | No |
| 36 | PPOX |  |  | 5513 | -1.046 | -0.5147 | No |
| 37 | ABCB6 |  |  | 5523 | -1.049 | -0.5056 | No |
| 38 | AAAS |  |  | 5713 | -1.159 | -0.5210 | Yes |
| 39 | SLC25A11 |  |  | 5729 | -1.168 | -0.5116 | Yes |
| 40 | NDUFA1 |  |  | 5747 | -1.178 | -0.5024 | Yes |
| 41 | KPNA1 |  |  | 5762 | -1.186 | -0.4926 | Yes |
| 42 | SURF1 |  |  | 5786 | -1.195 | -0.4841 | Yes |
| 43 | ABCF2 |  |  | 5811 | -1.211 | -0.4755 | Yes |
| 44 | TIMM8B |  |  | 5951 | -1.296 | -0.4824 | Yes |
| 45 | COX15 |  |  | 5952 | -1.296 | -0.4696 | Yes |
| 46 | PHB |  |  | 5961 | -1.303 | -0.4578 | Yes |
| 47 | PTGER3 |  |  | 5977 | -1.313 | -0.4470 | Yes |
| 48 | UCP3 |  |  | 5978 | -1.313 | -0.4340 | Yes |
| 49 | LMNB1 |  |  | 6039 | -1.357 | -0.4291 | Yes |
| 50 | NDUFA2 |  |  | 6084 | -1.402 | -0.4214 | Yes |
| 51 | MTX2 |  |  | 6229 | -1.535 | -0.4268 | Yes |
| 52 | TIMM9 |  |  | 6309 | -1.610 | -0.4221 | Yes |
| 53 | SDHD |  |  | 6419 | -1.723 | -0.4205 | Yes |
| 54 | TIMM10 |  |  | 6492 | -1.809 | -0.4129 | Yes |
| 55 | MFN2 |  |  | 6501 | -1.822 | -0.3960 | Yes |
| 56 | NDUFA9 |  |  | 6519 | -1.848 | -0.3801 | Yes |
| 57 | UQCRC1 |  |  | 6576 | -1.924 | -0.3690 | Yes |
| 58 | NDUFAB1 |  |  | 6622 | -1.998 | -0.3556 | Yes |
| 59 | MRPL32 |  |  | 6687 | -2.106 | -0.3439 | Yes |
| 60 | RAE1 |  |  | 6715 | -2.163 | -0.3263 | Yes |
| 61 | NDUFA6 |  |  | 6716 | -2.167 | -0.3048 | Yes |
| 62 | NUP54 |  |  | 6752 | -2.232 | -0.2877 | Yes |
| 63 | NDUFS2 |  |  | 6777 | -2.265 | -0.2687 | Yes |
| 64 | PARP1 |  |  | 6778 | -2.267 | -0.2462 | Yes |
| 65 | ABCB7 |  |  | 6790 | -2.286 | -0.2252 | Yes |
| 66 | NDUFS4 |  |  | 6792 | -2.295 | -0.2026 | Yes |
| 67 | PMPCA |  |  | 6795 | -2.314 | -0.1799 | Yes |
| 68 | ALAS2 |  |  | 6802 | -2.335 | -0.1576 | Yes |
| 69 | ATP5B |  |  | 6814 | -2.361 | -0.1358 | Yes |
| 70 | CYCS |  |  | 6844 | -2.437 | -0.1158 | Yes |
| 71 | ACN9 |  |  | 6898 | -2.650 | -0.0971 | Yes |
| 72 | RTN4 |  |  | 6909 | -2.681 | -0.0720 | Yes |
| 73 | OPA1 |  |  | 6912 | -2.702 | -0.0455 | Yes |
| 74 | TIMM50 |  |  | 6924 | -2.767 | -0.0196 | Yes |
| 75 | NDUFS1 |  |  | 7068 | -4.370 | 0.0033 | Yes |
Table: GSEA details [plain text format]

  

Fig 2: ENVELOPE: Random ES distribution      
 Gene set null distribution of ES for **ENVELOPE**
