# Supplementary material for: Network-based integration of molecular and physiological data elucidates regulatory mechanisms underlying adaptation to high-fat diet
Source: Genes Nutr. 2015 May 28;10(4):22. doi: 10.1007/s12263-015-0470-6 (PMC4446272; doi:10.1007/s12263-015-0470-6)
Supplement: Supplementary file 4 — Supplementary material 4 (ZIP 6984 kb) [file 12263_2015_470_MOESM4_ESM.zip › HF LF 5 d GSEA result/EXTRACELLULAR_MATRIX_PART.html]

Details for gene set EXTRACELLULAR\_MATRIX\_PART[GSEA]

|  || Dataset | comp\_HF5d-LF5d\_collapsed |
| Phenotype | NoPhenotypeAvailable |
| Upregulated in class | na\_pos |
| GeneSet | EXTRACELLULAR\_MATRIX\_PART |
| Enrichment Score (ES) | 0.62685376 |
| Normalized Enrichment Score (NES) | 2.1362 |
| Nominal p-value | 0.0 |
| FDR q-value | 0.0043387883 |
| FWER p-Value | 0.016 |
Table: GSEA Results Summary

  

Fig 1: Enrichment plot: EXTRACELLULAR\_MATRIX\_PART      
 Profile of the Running ES Score & Positions of GeneSet Members on the Rank Ordered List

  

| PROBE | GENE SYMBOL | GENE\_TITLE | RANK IN GENE LIST | RANK METRIC SCORE | RUNNING ES | CORE ENRICHMENT || 1 | EFEMP2 |  |  | 6 | 4.373 | 0.1354 | Yes |
| 2 | COL1A2 |  |  | 52 | 2.854 | 0.2179 | Yes |
| 3 | DST |  |  | 58 | 2.797 | 0.3043 | Yes |
| 4 | COL6A3 |  |  | 100 | 2.565 | 0.3784 | Yes |
| 5 | LUM |  |  | 208 | 2.217 | 0.4324 | Yes |
| 6 | COL3A1 |  |  | 221 | 2.186 | 0.4988 | Yes |
| 7 | SGCD |  |  | 524 | 1.686 | 0.5086 | Yes |
| 8 | FBN1 |  |  | 540 | 1.661 | 0.5582 | Yes |
| 9 | COL5A1 |  |  | 566 | 1.630 | 0.6054 | Yes |
| 10 | SNTB2 |  |  | 873 | 1.333 | 0.6037 | Yes |
| 11 | LAMB2 |  |  | 984 | 1.243 | 0.6269 | Yes |
| 12 | COL8A1 |  |  | 1546 | 0.881 | 0.5749 | No |
| 13 | COL4A3 |  |  | 1585 | 0.862 | 0.5964 | No |
| 14 | DMD |  |  | 2317 | 0.479 | 0.5080 | No |
| 15 | TINAG |  |  | 2691 | 0.305 | 0.4647 | No |
| 16 | COL4A2 |  |  | 3247 | 0.051 | 0.3878 | No |
| 17 | NID2 |  |  | 3369 | -0.007 | 0.3709 | No |
| 18 | LAMA4 |  |  | 4050 | -0.329 | 0.2849 | No |
| 19 | COL15A1 |  |  | 4545 | -0.552 | 0.2323 | No |
| 20 | COL5A3 |  |  | 5473 | -1.023 | 0.1330 | No |
| 21 | SNTB1 |  |  | 5613 | -1.097 | 0.1475 | No |
| 22 | MAGEE1 |  |  | 6598 | -1.971 | 0.0697 | No |
Table: GSEA details [plain text format]

  

Fig 2: EXTRACELLULAR\_MATRIX\_PART: Random ES distribution      
 Gene set null distribution of ES for **EXTRACELLULAR\_MATRIX\_PART**
