# Supplementary material for: Network-based integration of molecular and physiological data elucidates regulatory mechanisms underlying adaptation to high-fat diet
Source: Genes Nutr. 2015 May 28;10(4):22. doi: 10.1007/s12263-015-0470-6 (PMC4446272; doi:10.1007/s12263-015-0470-6)
Supplement: Supplementary file 4 — Supplementary material 4 (ZIP 6984 kb) [file 12263_2015_470_MOESM4_ESM.zip › HF LF 5 d GSEA result/EXTRACELLULAR_REGION.html]

Details for gene set EXTRACELLULAR\_REGION[GSEA]

|  || Dataset | comp\_HF5d-LF5d\_collapsed |
| Phenotype | NoPhenotypeAvailable |
| Upregulated in class | na\_pos |
| GeneSet | EXTRACELLULAR\_REGION |
| Enrichment Score (ES) | 0.38222796 |
| Normalized Enrichment Score (NES) | 1.899564 |
| Nominal p-value | 0.0 |
| FDR q-value | 0.020303175 |
| FWER p-Value | 0.21 |
Table: GSEA Results Summary

  

Fig 1: Enrichment plot: EXTRACELLULAR\_REGION      
 Profile of the Running ES Score & Positions of GeneSet Members on the Rank Ordered List

  

| PROBE | GENE SYMBOL | GENE\_TITLE | RANK IN GENE LIST | RANK METRIC SCORE | RUNNING ES | CORE ENRICHMENT || 1 | EFEMP2 |  |  | 6 | 4.373 | 0.0249 | Yes |
| 2 | CD248 |  |  | 15 | 3.586 | 0.0448 | Yes |
| 3 | LBP |  |  | 43 | 2.939 | 0.0582 | Yes |
| 4 | COL1A2 |  |  | 52 | 2.854 | 0.0738 | Yes |
| 5 | DST |  |  | 58 | 2.797 | 0.0896 | Yes |
| 6 | LEP |  |  | 74 | 2.711 | 0.1034 | Yes |
| 7 | ANGPT2 |  |  | 77 | 2.697 | 0.1189 | Yes |
| 8 | APOE |  |  | 86 | 2.652 | 0.1334 | Yes |
| 9 | COL6A3 |  |  | 100 | 2.565 | 0.1466 | Yes |
| 10 | CCL7 |  |  | 128 | 2.452 | 0.1571 | Yes |
| 11 | LOXL1 |  |  | 136 | 2.419 | 0.1704 | Yes |
| 12 | PCSK5 |  |  | 154 | 2.356 | 0.1818 | Yes |
| 13 | GPC1 |  |  | 169 | 2.314 | 0.1934 | Yes |
| 14 | LUM |  |  | 208 | 2.217 | 0.2009 | Yes |
| 15 | COL3A1 |  |  | 221 | 2.186 | 0.2121 | Yes |
| 16 | SFRP4 |  |  | 223 | 2.185 | 0.2248 | Yes |
| 17 | ECM1 |  |  | 288 | 2.049 | 0.2276 | Yes |
| 18 | EMILIN2 |  |  | 297 | 2.038 | 0.2384 | Yes |
| 19 | IL16 |  |  | 316 | 2.005 | 0.2476 | Yes |
| 20 | C2 |  |  | 340 | 1.966 | 0.2559 | Yes |
| 21 | SPN |  |  | 342 | 1.962 | 0.2673 | Yes |
| 22 | SERPINF1 |  |  | 344 | 1.961 | 0.2787 | Yes |
| 23 | LOXL2 |  |  | 475 | 1.742 | 0.2702 | Yes |
| 24 | C1QB |  |  | 495 | 1.710 | 0.2775 | Yes |
| 25 | C1QA |  |  | 521 | 1.688 | 0.2839 | Yes |
| 26 | SGCD |  |  | 524 | 1.686 | 0.2935 | Yes |
| 27 | FBN1 |  |  | 540 | 1.661 | 0.3011 | Yes |
| 28 | COL5A1 |  |  | 566 | 1.630 | 0.3071 | Yes |
| 29 | TGFB2 |  |  | 572 | 1.624 | 0.3159 | Yes |
| 30 | FBLN1 |  |  | 587 | 1.604 | 0.3233 | Yes |
| 31 | TGFBI |  |  | 594 | 1.595 | 0.3319 | Yes |
| 32 | CALCA |  |  | 607 | 1.582 | 0.3394 | Yes |
| 33 | FBLN2 |  |  | 614 | 1.574 | 0.3478 | Yes |
| 34 | FXYD6 |  |  | 711 | 1.484 | 0.3428 | Yes |
| 35 | FMOD |  |  | 841 | 1.362 | 0.3322 | Yes |
| 36 | SNTB2 |  |  | 873 | 1.333 | 0.3356 | Yes |
| 37 | CCL2 |  |  | 875 | 1.330 | 0.3432 | Yes |
| 38 | SLIT3 |  |  | 887 | 1.321 | 0.3494 | Yes |
| 39 | KL |  |  | 940 | 1.278 | 0.3495 | Yes |
| 40 | LAMB2 |  |  | 984 | 1.243 | 0.3506 | Yes |
| 41 | EBI3 |  |  | 1004 | 1.226 | 0.3551 | Yes |
| 42 | PLA2G4B |  |  | 1027 | 1.215 | 0.3590 | Yes |
| 43 | MMP7 |  |  | 1030 | 1.211 | 0.3659 | Yes |
| 44 | CTGF |  |  | 1033 | 1.207 | 0.3727 | Yes |
| 45 | MMP2 |  |  | 1160 | 1.118 | 0.3611 | Yes |
| 46 | SECTM1 |  |  | 1174 | 1.111 | 0.3658 | Yes |
| 47 | F13A1 |  |  | 1175 | 1.110 | 0.3723 | Yes |
| 48 | IFI30 |  |  | 1189 | 1.098 | 0.3769 | Yes |
| 49 | MGP |  |  | 1234 | 1.077 | 0.3769 | Yes |
| 50 | RAB35 |  |  | 1258 | 1.062 | 0.3798 | Yes |
| 51 | CDA |  |  | 1285 | 1.042 | 0.3822 | Yes |
| 52 | CX3CL1 |  |  | 1442 | 0.942 | 0.3653 | No |
| 53 | IGFBP1 |  |  | 1453 | 0.936 | 0.3694 | No |
| 54 | SPINT1 |  |  | 1473 | 0.920 | 0.3721 | No |
| 55 | PLA2G2D |  |  | 1474 | 0.920 | 0.3775 | No |
| 56 | COL8A1 |  |  | 1546 | 0.881 | 0.3724 | No |
| 57 | COL4A3 |  |  | 1585 | 0.862 | 0.3720 | No |
| 58 | HYAL1 |  |  | 1586 | 0.862 | 0.3771 | No |
| 59 | TNFRSF11B |  |  | 1608 | 0.853 | 0.3791 | No |
| 60 | INHBA |  |  | 1623 | 0.843 | 0.3820 | No |
| 61 | HBEGF |  |  | 1689 | 0.808 | 0.3774 | No |
| 62 | CCL4 |  |  | 1696 | 0.805 | 0.3813 | No |
| 63 | FGL2 |  |  | 1832 | 0.730 | 0.3662 | No |
| 64 | TNFAIP2 |  |  | 1862 | 0.711 | 0.3662 | No |
| 65 | ADM |  |  | 1975 | 0.649 | 0.3539 | No |
| 66 | DKKL1 |  |  | 2046 | 0.606 | 0.3474 | No |
| 67 | CLEC11A |  |  | 2205 | 0.529 | 0.3277 | No |
| 68 | COMP |  |  | 2208 | 0.527 | 0.3305 | No |
| 69 | IK |  |  | 2213 | 0.525 | 0.3330 | No |
| 70 | FGG |  |  | 2242 | 0.507 | 0.3320 | No |
| 71 | ANGPTL4 |  |  | 2275 | 0.496 | 0.3303 | No |
| 72 | FGB |  |  | 2313 | 0.480 | 0.3278 | No |
| 73 | DMD |  |  | 2317 | 0.479 | 0.3302 | No |
| 74 | SDF2 |  |  | 2436 | 0.427 | 0.3157 | No |
| 75 | CXCL9 |  |  | 2452 | 0.418 | 0.3160 | No |
| 76 | PTHLH |  |  | 2519 | 0.387 | 0.3088 | No |
| 77 | VWF |  |  | 2528 | 0.381 | 0.3099 | No |
| 78 | PVR |  |  | 2536 | 0.378 | 0.3111 | No |
| 79 | CFH |  |  | 2633 | 0.330 | 0.2992 | No |
| 80 | RNH1 |  |  | 2663 | 0.316 | 0.2969 | No |
| 81 | TINAG |  |  | 2691 | 0.305 | 0.2948 | No |
| 82 | EDN1 |  |  | 2710 | 0.296 | 0.2940 | No |
| 83 | ANGPTL1 |  |  | 2713 | 0.295 | 0.2954 | No |
| 84 | PRG2 |  |  | 2775 | 0.262 | 0.2882 | No |
| 85 | NUCB2 |  |  | 2788 | 0.257 | 0.2880 | No |
| 86 | CDH13 |  |  | 2816 | 0.246 | 0.2855 | No |
| 87 | APOA1 |  |  | 2876 | 0.230 | 0.2784 | No |
| 88 | IL15 |  |  | 2879 | 0.229 | 0.2794 | No |
| 89 | LTBP4 |  |  | 2951 | 0.190 | 0.2703 | No |
| 90 | ANGPTL3 |  |  | 2990 | 0.171 | 0.2659 | No |
| 91 | CD5L |  |  | 3055 | 0.136 | 0.2575 | No |
| 92 | EFNA5 |  |  | 3056 | 0.136 | 0.2583 | No |
| 93 | CRISP1 |  |  | 3236 | 0.056 | 0.2328 | No |
| 94 | COL4A2 |  |  | 3247 | 0.051 | 0.2317 | No |
| 95 | CHAD |  |  | 3254 | 0.047 | 0.2311 | No |
| 96 | LGALS3BP |  |  | 3288 | 0.032 | 0.2265 | No |
| 97 | RNASE6 |  |  | 3342 | 0.009 | 0.2190 | No |
| 98 | CPB2 |  |  | 3357 | 0.001 | 0.2170 | No |
| 99 | NID2 |  |  | 3369 | -0.007 | 0.2154 | No |
| 100 | RTN3 |  |  | 3503 | -0.062 | 0.1966 | No |
| 101 | LGALS7 |  |  | 3523 | -0.074 | 0.1943 | No |
| 102 | IFNAR2 |  |  | 3964 | -0.291 | 0.1327 | No |
| 103 | LAMA4 |  |  | 4050 | -0.329 | 0.1224 | No |
| 104 | VTN |  |  | 4146 | -0.377 | 0.1110 | No |
| 105 | FGF10 |  |  | 4218 | -0.417 | 0.1032 | No |
| 106 | IGFALS |  |  | 4264 | -0.432 | 0.0992 | No |
| 107 | PCSK2 |  |  | 4278 | -0.439 | 0.1000 | No |
| 108 | PNLIPRP1 |  |  | 4410 | -0.495 | 0.0840 | No |
| 109 | MYOC |  |  | 4446 | -0.509 | 0.0820 | No |
| 110 | WFDC12 |  |  | 4450 | -0.510 | 0.0845 | No |
| 111 | COL15A1 |  |  | 4545 | -0.552 | 0.0743 | No |
| 112 | INHA |  |  | 4699 | -0.622 | 0.0559 | No |
| 113 | FGF2 |  |  | 4723 | -0.629 | 0.0563 | No |
| 114 | ERBB2 |  |  | 4844 | -0.690 | 0.0431 | No |
| 115 | FLT1 |  |  | 4943 | -0.739 | 0.0333 | No |
| 116 | ADAMTS9 |  |  | 4985 | -0.756 | 0.0318 | No |
| 117 | TGFB1 |  |  | 5288 | -0.910 | -0.0063 | No |
| 118 | NUCB1 |  |  | 5299 | -0.916 | -0.0023 | No |
| 119 | KLK10 |  |  | 5359 | -0.954 | -0.0052 | No |
| 120 | LPL |  |  | 5401 | -0.976 | -0.0054 | No |
| 121 | MMP9 |  |  | 5409 | -0.981 | -0.0006 | No |
| 122 | COL5A3 |  |  | 5473 | -1.023 | -0.0037 | No |
| 123 | PCSK1N |  |  | 5514 | -1.046 | -0.0033 | No |
| 124 | RNPEP |  |  | 5554 | -1.066 | -0.0026 | No |
| 125 | NOG |  |  | 5580 | -1.079 | 0.0001 | No |
| 126 | HDGF |  |  | 5582 | -1.080 | 0.0063 | No |
| 127 | SNTB1 |  |  | 5613 | -1.097 | 0.0085 | No |
| 128 | NPY |  |  | 5639 | -1.111 | 0.0114 | No |
| 129 | COPA |  |  | 5705 | -1.156 | 0.0089 | No |
| 130 | GPX3 |  |  | 5836 | -1.226 | -0.0026 | No |
| 131 | IL27 |  |  | 5890 | -1.256 | -0.0029 | No |
| 132 | LIPE |  |  | 5906 | -1.268 | 0.0024 | No |
| 133 | KLK8 |  |  | 6075 | -1.396 | -0.0136 | No |
| 134 | CTRL |  |  | 6182 | -1.485 | -0.0201 | No |
| 135 | FIGF |  |  | 6302 | -1.600 | -0.0278 | No |
| 136 | MAGEE1 |  |  | 6598 | -1.971 | -0.0587 | No |
| 137 | PSAP |  |  | 6661 | -2.062 | -0.0555 | No |
| 138 | ORM2 |  |  | 6678 | -2.093 | -0.0455 | No |
| 139 | TFRC |  |  | 6709 | -2.149 | -0.0371 | No |
| 140 | ORM1 |  |  | 6710 | -2.152 | -0.0245 | No |
| 141 | APOA1BP |  |  | 6750 | -2.229 | -0.0170 | No |
| 142 | IL18BP |  |  | 6798 | -2.318 | -0.0101 | No |
| 143 | CTF1 |  |  | 6799 | -2.323 | 0.0036 | No |
| 144 | CABP4 |  |  | 6919 | -2.745 | 0.0026 | No |
| 145 | IDE |  |  | 7042 | -3.748 | 0.0071 | No |
Table: GSEA details [plain text format]

  

Fig 2: EXTRACELLULAR\_REGION: Random ES distribution      
 Gene set null distribution of ES for **EXTRACELLULAR\_REGION**
