# Supplementary material for: Network-based integration of molecular and physiological data elucidates regulatory mechanisms underlying adaptation to high-fat diet
Source: Genes Nutr. 2015 May 28;10(4):22. doi: 10.1007/s12263-015-0470-6 (PMC4446272; doi:10.1007/s12263-015-0470-6)
Supplement: Supplementary file 4 — Supplementary material 4 (ZIP 6984 kb) [file 12263_2015_470_MOESM4_ESM.zip › HF LF 5 d GSEA result/EXTRACELLULAR_REGION_PART.html]

Details for gene set EXTRACELLULAR\_REGION\_PART[GSEA]

|  || Dataset | comp\_HF5d-LF5d\_collapsed |
| Phenotype | NoPhenotypeAvailable |
| Upregulated in class | na\_pos |
| GeneSet | EXTRACELLULAR\_REGION\_PART |
| Enrichment Score (ES) | 0.42604512 |
| Normalized Enrichment Score (NES) | 2.0554872 |
| Nominal p-value | 0.0 |
| FDR q-value | 0.00906517 |
| FWER p-Value | 0.048 |
Table: GSEA Results Summary

  

Fig 1: Enrichment plot: EXTRACELLULAR\_REGION\_PART      
 Profile of the Running ES Score & Positions of GeneSet Members on the Rank Ordered List

  

| PROBE | GENE SYMBOL | GENE\_TITLE | RANK IN GENE LIST | RANK METRIC SCORE | RUNNING ES | CORE ENRICHMENT || 1 | EFEMP2 |  |  | 6 | 4.373 | 0.0319 | Yes |
| 2 | CD248 |  |  | 15 | 3.586 | 0.0577 | Yes |
| 3 | LBP |  |  | 43 | 2.939 | 0.0759 | Yes |
| 4 | COL1A2 |  |  | 52 | 2.854 | 0.0961 | Yes |
| 5 | DST |  |  | 58 | 2.797 | 0.1164 | Yes |
| 6 | LEP |  |  | 74 | 2.711 | 0.1346 | Yes |
| 7 | ANGPT2 |  |  | 77 | 2.697 | 0.1545 | Yes |
| 8 | COL6A3 |  |  | 100 | 2.565 | 0.1706 | Yes |
| 9 | CCL7 |  |  | 128 | 2.452 | 0.1851 | Yes |
| 10 | PCSK5 |  |  | 154 | 2.356 | 0.1992 | Yes |
| 11 | GPC1 |  |  | 169 | 2.314 | 0.2146 | Yes |
| 12 | LUM |  |  | 208 | 2.217 | 0.2258 | Yes |
| 13 | COL3A1 |  |  | 221 | 2.186 | 0.2405 | Yes |
| 14 | SFRP4 |  |  | 223 | 2.185 | 0.2567 | Yes |
| 15 | ECM1 |  |  | 288 | 2.049 | 0.2629 | Yes |
| 16 | IL16 |  |  | 316 | 2.005 | 0.2741 | Yes |
| 17 | C2 |  |  | 340 | 1.966 | 0.2855 | Yes |
| 18 | SPN |  |  | 342 | 1.962 | 0.3001 | Yes |
| 19 | LOXL2 |  |  | 475 | 1.742 | 0.2942 | Yes |
| 20 | C1QB |  |  | 495 | 1.710 | 0.3043 | Yes |
| 21 | C1QA |  |  | 521 | 1.688 | 0.3134 | Yes |
| 22 | SGCD |  |  | 524 | 1.686 | 0.3258 | Yes |
| 23 | FBN1 |  |  | 540 | 1.661 | 0.3361 | Yes |
| 24 | COL5A1 |  |  | 566 | 1.630 | 0.3447 | Yes |
| 25 | FBLN1 |  |  | 587 | 1.604 | 0.3539 | Yes |
| 26 | TGFBI |  |  | 594 | 1.595 | 0.3650 | Yes |
| 27 | CALCA |  |  | 607 | 1.582 | 0.3752 | Yes |
| 28 | FBLN2 |  |  | 614 | 1.574 | 0.3861 | Yes |
| 29 | FXYD6 |  |  | 711 | 1.484 | 0.3835 | Yes |
| 30 | FMOD |  |  | 841 | 1.362 | 0.3752 | Yes |
| 31 | SNTB2 |  |  | 873 | 1.333 | 0.3808 | Yes |
| 32 | CCL2 |  |  | 875 | 1.330 | 0.3906 | Yes |
| 33 | SLIT3 |  |  | 887 | 1.321 | 0.3989 | Yes |
| 34 | KL |  |  | 940 | 1.278 | 0.4011 | Yes |
| 35 | LAMB2 |  |  | 984 | 1.243 | 0.4042 | Yes |
| 36 | EBI3 |  |  | 1004 | 1.226 | 0.4107 | Yes |
| 37 | MMP7 |  |  | 1030 | 1.211 | 0.4162 | Yes |
| 38 | CTGF |  |  | 1033 | 1.207 | 0.4250 | Yes |
| 39 | MMP2 |  |  | 1160 | 1.118 | 0.4153 | Yes |
| 40 | SECTM1 |  |  | 1174 | 1.111 | 0.4218 | Yes |
| 41 | MGP |  |  | 1234 | 1.077 | 0.4214 | Yes |
| 42 | RAB35 |  |  | 1258 | 1.062 | 0.4260 | Yes |
| 43 | IGFBP1 |  |  | 1453 | 0.936 | 0.4053 | No |
| 44 | COL8A1 |  |  | 1546 | 0.881 | 0.3987 | No |
| 45 | COL4A3 |  |  | 1585 | 0.862 | 0.3997 | No |
| 46 | HYAL1 |  |  | 1586 | 0.862 | 0.4062 | No |
| 47 | INHBA |  |  | 1623 | 0.843 | 0.4073 | No |
| 48 | HBEGF |  |  | 1689 | 0.808 | 0.4041 | No |
| 49 | CCL4 |  |  | 1696 | 0.805 | 0.4092 | No |
| 50 | FGL2 |  |  | 1832 | 0.730 | 0.3954 | No |
| 51 | TNFAIP2 |  |  | 1862 | 0.711 | 0.3965 | No |
| 52 | ADM |  |  | 1975 | 0.649 | 0.3854 | No |
| 53 | DKKL1 |  |  | 2046 | 0.606 | 0.3799 | No |
| 54 | COMP |  |  | 2208 | 0.527 | 0.3607 | No |
| 55 | IK |  |  | 2213 | 0.525 | 0.3641 | No |
| 56 | FGG |  |  | 2242 | 0.507 | 0.3639 | No |
| 57 | FGB |  |  | 2313 | 0.480 | 0.3575 | No |
| 58 | DMD |  |  | 2317 | 0.479 | 0.3606 | No |
| 59 | SDF2 |  |  | 2436 | 0.427 | 0.3469 | No |
| 60 | CXCL9 |  |  | 2452 | 0.418 | 0.3479 | No |
| 61 | PTHLH |  |  | 2519 | 0.387 | 0.3413 | No |
| 62 | PVR |  |  | 2536 | 0.378 | 0.3419 | No |
| 63 | CFH |  |  | 2633 | 0.330 | 0.3306 | No |
| 64 | RNH1 |  |  | 2663 | 0.316 | 0.3288 | No |
| 65 | TINAG |  |  | 2691 | 0.305 | 0.3272 | No |
| 66 | EDN1 |  |  | 2710 | 0.296 | 0.3269 | No |
| 67 | ANGPTL1 |  |  | 2713 | 0.295 | 0.3288 | No |
| 68 | NUCB2 |  |  | 2788 | 0.257 | 0.3201 | No |
| 69 | CDH13 |  |  | 2816 | 0.246 | 0.3181 | No |
| 70 | APOA1 |  |  | 2876 | 0.230 | 0.3114 | No |
| 71 | IL15 |  |  | 2879 | 0.229 | 0.3128 | No |
| 72 | LTBP4 |  |  | 2951 | 0.190 | 0.3040 | No |
| 73 | ANGPTL3 |  |  | 2990 | 0.171 | 0.2999 | No |
| 74 | CD5L |  |  | 3055 | 0.136 | 0.2917 | No |
| 75 | EFNA5 |  |  | 3056 | 0.136 | 0.2927 | No |
| 76 | CRISP1 |  |  | 3236 | 0.056 | 0.2675 | No |
| 77 | COL4A2 |  |  | 3247 | 0.051 | 0.2665 | No |
| 78 | CHAD |  |  | 3254 | 0.047 | 0.2660 | No |
| 79 | LGALS3BP |  |  | 3288 | 0.032 | 0.2615 | No |
| 80 | CPB2 |  |  | 3357 | 0.001 | 0.2517 | No |
| 81 | NID2 |  |  | 3369 | -0.007 | 0.2502 | No |
| 82 | RTN3 |  |  | 3503 | -0.062 | 0.2316 | No |
| 83 | LGALS7 |  |  | 3523 | -0.074 | 0.2294 | No |
| 84 | LAMA4 |  |  | 4050 | -0.329 | 0.1565 | No |
| 85 | VTN |  |  | 4146 | -0.377 | 0.1457 | No |
| 86 | FGF10 |  |  | 4218 | -0.417 | 0.1387 | No |
| 87 | IGFALS |  |  | 4264 | -0.432 | 0.1354 | No |
| 88 | PCSK2 |  |  | 4278 | -0.439 | 0.1369 | No |
| 89 | MYOC |  |  | 4446 | -0.509 | 0.1167 | No |
| 90 | WFDC12 |  |  | 4450 | -0.510 | 0.1201 | No |
| 91 | COL15A1 |  |  | 4545 | -0.552 | 0.1108 | No |
| 92 | INHA |  |  | 4699 | -0.622 | 0.0935 | No |
| 93 | FGF2 |  |  | 4723 | -0.629 | 0.0950 | No |
| 94 | FLT1 |  |  | 4943 | -0.739 | 0.0691 | No |
| 95 | ADAMTS9 |  |  | 4985 | -0.756 | 0.0689 | No |
| 96 | TGFB1 |  |  | 5288 | -0.910 | 0.0324 | No |
| 97 | NUCB1 |  |  | 5299 | -0.916 | 0.0379 | No |
| 98 | MMP9 |  |  | 5409 | -0.981 | 0.0296 | No |
| 99 | COL5A3 |  |  | 5473 | -1.023 | 0.0283 | No |
| 100 | PCSK1N |  |  | 5514 | -1.046 | 0.0304 | No |
| 101 | NOG |  |  | 5580 | -1.079 | 0.0292 | No |
| 102 | HDGF |  |  | 5582 | -1.080 | 0.0371 | No |
| 103 | SNTB1 |  |  | 5613 | -1.097 | 0.0410 | No |
| 104 | NPY |  |  | 5639 | -1.111 | 0.0458 | No |
| 105 | COPA |  |  | 5705 | -1.156 | 0.0451 | No |
| 106 | GPX3 |  |  | 5836 | -1.226 | 0.0357 | No |
| 107 | IL27 |  |  | 5890 | -1.256 | 0.0375 | No |
| 108 | LIPE |  |  | 5906 | -1.268 | 0.0449 | No |
| 109 | KLK8 |  |  | 6075 | -1.396 | 0.0313 | No |
| 110 | CTRL |  |  | 6182 | -1.485 | 0.0272 | No |
| 111 | FIGF |  |  | 6302 | -1.600 | 0.0222 | No |
| 112 | MAGEE1 |  |  | 6598 | -1.971 | -0.0053 | No |
| 113 | PSAP |  |  | 6661 | -2.062 | 0.0013 | No |
| 114 | ORM2 |  |  | 6678 | -2.093 | 0.0147 | No |
| 115 | ORM1 |  |  | 6710 | -2.152 | 0.0264 | No |
| 116 | IDE |  |  | 7042 | -3.748 | 0.0070 | No |
Table: GSEA details [plain text format]

  

Fig 2: EXTRACELLULAR\_REGION\_PART: Random ES distribution      
 Gene set null distribution of ES for **EXTRACELLULAR\_REGION\_PART**
