# Supplementary material for: Network-based integration of molecular and physiological data elucidates regulatory mechanisms underlying adaptation to high-fat diet
Source: Genes Nutr. 2015 May 28;10(4):22. doi: 10.1007/s12263-015-0470-6 (PMC4446272; doi:10.1007/s12263-015-0470-6)
Supplement: Supplementary file 4 — Supplementary material 4 (ZIP 6984 kb) [file 12263_2015_470_MOESM4_ESM.zip › HF LF 5 d GSEA result/G_PROTEIN_COUPLED_RECEPTOR_BINDING.html]

Details for gene set G\_PROTEIN\_COUPLED\_RECEPTOR\_BINDING[GSEA]

|  || Dataset | comp\_HF5d-LF5d\_collapsed |
| Phenotype | NoPhenotypeAvailable |
| Upregulated in class | na\_pos |
| GeneSet | G\_PROTEIN\_COUPLED\_RECEPTOR\_BINDING |
| Enrichment Score (ES) | 0.60512483 |
| Normalized Enrichment Score (NES) | 1.9582822 |
| Nominal p-value | 0.0025316456 |
| FDR q-value | 0.015564197 |
| FWER p-Value | 0.126 |
Table: GSEA Results Summary

  

Fig 1: Enrichment plot: G\_PROTEIN\_COUPLED\_RECEPTOR\_BINDING      
 Profile of the Running ES Score & Positions of GeneSet Members on the Rank Ordered List

  

| PROBE | GENE SYMBOL | GENE\_TITLE | RANK IN GENE LIST | RANK METRIC SCORE | RUNNING ES | CORE ENRICHMENT || 1 | CCL8 |  |  | 56 | 2.814 | 0.0974 | Yes |
| 2 | CCL7 |  |  | 128 | 2.452 | 0.1791 | Yes |
| 3 | CXCL16 |  |  | 193 | 2.250 | 0.2542 | Yes |
| 4 | CCR2 |  |  | 219 | 2.188 | 0.3325 | Yes |
| 5 | CCL5 |  |  | 241 | 2.144 | 0.4097 | Yes |
| 6 | PF4 |  |  | 290 | 2.044 | 0.4794 | Yes |
| 7 | CXCL14 |  |  | 428 | 1.821 | 0.5282 | Yes |
| 8 | CALCA |  |  | 607 | 1.582 | 0.5622 | Yes |
| 9 | CCL2 |  |  | 875 | 1.330 | 0.5742 | Yes |
| 10 | CCL17 |  |  | 986 | 1.243 | 0.6051 | Yes |
| 11 | CX3CL1 |  |  | 1442 | 0.942 | 0.5760 | No |
| 12 | CCL4 |  |  | 1696 | 0.805 | 0.5704 | No |
| 13 | SLC9A3R1 |  |  | 1937 | 0.671 | 0.5616 | No |
| 14 | CXCL13 |  |  | 2207 | 0.528 | 0.5433 | No |
| 15 | CXCL9 |  |  | 2452 | 0.418 | 0.5244 | No |
| 16 | CCL11 |  |  | 2459 | 0.413 | 0.5390 | No |
| 17 | CXCL12 |  |  | 2746 | 0.280 | 0.5090 | No |
| 18 | CCL24 |  |  | 2825 | 0.243 | 0.5071 | No |
| 19 | CCL25 |  |  | 4155 | -0.381 | 0.3334 | No |
| 20 | PSMC5 |  |  | 6722 | -2.180 | 0.0522 | No |
Table: GSEA details [plain text format]

  

Fig 2: G\_PROTEIN\_COUPLED\_RECEPTOR\_BINDING: Random ES distribution      
 Gene set null distribution of ES for **G\_PROTEIN\_COUPLED\_RECEPTOR\_BINDING**
