# Supplementary material for: Network-based integration of molecular and physiological data elucidates regulatory mechanisms underlying adaptation to high-fat diet
Source: Genes Nutr. 2015 May 28;10(4):22. doi: 10.1007/s12263-015-0470-6 (PMC4446272; doi:10.1007/s12263-015-0470-6)
Supplement: Supplementary file 4 — Supplementary material 4 (ZIP 6984 kb) [file 12263_2015_470_MOESM4_ESM.zip › HF LF 5 d GSEA result/gsea_report_for_na_neg_1365164922288.html]

Report for na\_neg 1365164922288 [GSEA]

| GS  follow link to MSigDB | GS DETAILS | SIZE | ES | NES | NOM p-val | FDR q-val | FWER p-val | RANK AT MAX | LEADING EDGE || 1 | MITOCHONDRIAL\_PART | Details ... | 67 | -0.65 | -2.55 | 0.000 | 0.000 | 0.000 | 1580 | tags=72%, list=22%, signal=91% |
| 2 | MITOCHONDRION | Details ... | 153 | -0.57 | -2.55 | 0.000 | 0.000 | 0.000 | 1580 | tags=56%, list=22%, signal=70% |
| 3 | MITOCHONDRIAL\_INNER\_MEMBRANE | Details ... | 31 | -0.75 | -2.52 | 0.000 | 0.000 | 0.000 | 1364 | tags=81%, list=19%, signal=99% |
| 4 | ORGANELLE\_INNER\_MEMBRANE | Details ... | 35 | -0.71 | -2.44 | 0.000 | 0.000 | 0.000 | 1364 | tags=74%, list=19%, signal=92% |
| 5 | MITOCHONDRIAL\_MATRIX | Details ... | 24 | -0.73 | -2.30 | 0.000 | 0.000 | 0.000 | 471 | tags=54%, list=7%, signal=58% |
| 6 | MITOCHONDRIAL\_MEMBRANE\_PART | Details ... | 24 | -0.72 | -2.29 | 0.000 | 0.000 | 0.000 | 1346 | tags=75%, list=19%, signal=92% |
| 7 | MITOCHONDRIAL\_LUMEN | Details ... | 24 | -0.73 | -2.29 | 0.000 | 0.000 | 0.000 | 471 | tags=54%, list=7%, signal=58% |
| 8 | MITOCHONDRIAL\_ENVELOPE | Details ... | 44 | -0.63 | -2.27 | 0.000 | 0.000 | 0.000 | 1364 | tags=68%, list=19%, signal=84% |
| 9 | MITOCHONDRIAL\_MEMBRANE | Details ... | 38 | -0.63 | -2.24 | 0.000 | 0.000 | 0.001 | 1364 | tags=71%, list=19%, signal=88% |
| 10 | ENVELOPE | Details ... | 75 | -0.53 | -2.15 | 0.000 | 0.001 | 0.008 | 1380 | tags=51%, list=19%, signal=62% |
| 11 | OXIDOREDUCTASE\_ACTIVITY | Details ... | 112 | -0.49 | -2.15 | 0.000 | 0.000 | 0.008 | 1051 | tags=38%, list=15%, signal=44% |
| 12 | ORGANELLE\_ENVELOPE | Details ... | 75 | -0.53 | -2.11 | 0.000 | 0.001 | 0.014 | 1380 | tags=51%, list=19%, signal=62% |
| 13 | PEROXISOME | Details ... | 25 | -0.65 | -2.08 | 0.000 | 0.001 | 0.022 | 761 | tags=44%, list=11%, signal=49% |
| 14 | MICROBODY | Details ... | 25 | -0.65 | -2.07 | 0.000 | 0.001 | 0.022 | 761 | tags=44%, list=11%, signal=49% |
| 15 | OXIDOREDUCTASE\_ACTIVITY\_ACTING\_ON\_CH\_OH\_GROUP\_OF\_DONORS | Details ... | 20 | -0.67 | -1.99 | 0.002 | 0.003 | 0.064 | 558 | tags=45%, list=8%, signal=49% |
| 16 | ELECTRON\_CARRIER\_ACTIVITY | Details ... | 27 | -0.60 | -1.94 | 0.003 | 0.006 | 0.126 | 674 | tags=48%, list=10%, signal=53% |
| 17 | ORGANELLE\_MEMBRANE | Details ... | 133 | -0.44 | -1.92 | 0.000 | 0.006 | 0.148 | 1732 | tags=45%, list=24%, signal=59% |
| 18 | COFACTOR\_METABOLIC\_PROCESS | Details ... | 18 | -0.65 | -1.92 | 0.003 | 0.006 | 0.159 | 1580 | tags=61%, list=22%, signal=78% |
| 19 | OXIDOREDUCTASE\_ACTIVITY\_GO\_0016616 | Details ... | 18 | -0.62 | -1.79 | 0.005 | 0.025 | 0.518 | 1813 | tags=72%, list=26%, signal=97% |
| 20 | CHROMATIN\_MODIFICATION | Details ... | 16 | -0.61 | -1.76 | 0.010 | 0.036 | 0.667 | 1440 | tags=56%, list=20%, signal=70% |
| 21 | TRANSFERASE\_ACTIVITY\_TRANSFERRING\_ALKYL\_OR\_ARYLOTHER\_THAN\_METHYLGROUPS |  | 16 | -0.60 | -1.71 | 0.013 | 0.057 | 0.831 | 2131 | tags=69%, list=30%, signal=98% |
| 22 | INORGANIC\_CATION\_TRANSMEMBRANE\_TRANSPORTER\_ACTIVITY |  | 21 | -0.57 | -1.70 | 0.007 | 0.056 | 0.836 | 1426 | tags=52%, list=20%, signal=65% |
| 23 | MITOCHONDRION\_ORGANIZATION\_AND\_BIOGENESIS |  | 21 | -0.55 | -1.69 | 0.011 | 0.063 | 0.880 | 864 | tags=38%, list=12%, signal=43% |
| 24 | MACROMOLECULE\_CATABOLIC\_PROCESS |  | 53 | -0.44 | -1.66 | 0.006 | 0.079 | 0.936 | 1417 | tags=36%, list=20%, signal=44% |
| 25 | MEMBRANE\_ENCLOSED\_LUMEN |  | 155 | -0.37 | -1.65 | 0.001 | 0.076 | 0.936 | 1811 | tags=40%, list=26%, signal=53% |
| 26 | ORGANELLE\_LUMEN |  | 155 | -0.37 | -1.65 | 0.000 | 0.074 | 0.938 | 1811 | tags=40%, list=26%, signal=53% |
| 27 | CATABOLIC\_PROCESS |  | 87 | -0.40 | -1.64 | 0.001 | 0.082 | 0.966 | 1417 | tags=34%, list=20%, signal=43% |
| 28 | BIOPOLYMER\_CATABOLIC\_PROCESS |  | 48 | -0.45 | -1.64 | 0.012 | 0.080 | 0.966 | 1417 | tags=35%, list=20%, signal=44% |
| 29 | INTRINSIC\_TO\_ORGANELLE\_MEMBRANE |  | 25 | -0.51 | -1.61 | 0.018 | 0.099 | 0.983 | 783 | tags=28%, list=11%, signal=31% |
| 30 | GENERATION\_OF\_PRECURSOR\_METABOLITES\_AND\_ENERGY |  | 47 | -0.44 | -1.59 | 0.012 | 0.112 | 0.992 | 1580 | tags=45%, list=22%, signal=57% |
| 31 | CELLULAR\_CATABOLIC\_PROCESS |  | 84 | -0.39 | -1.59 | 0.004 | 0.110 | 0.993 | 1417 | tags=35%, list=20%, signal=43% |
| 32 | ORGANELLE\_PART |  | 418 | -0.32 | -1.58 | 0.000 | 0.109 | 0.993 | 1781 | tags=35%, list=25%, signal=44% |
| 33 | INTRACELLULAR\_ORGANELLE\_PART |  | 415 | -0.31 | -1.58 | 0.000 | 0.109 | 0.994 | 1781 | tags=35%, list=25%, signal=44% |
| 34 | LIGASE\_ACTIVITY |  | 48 | -0.43 | -1.57 | 0.018 | 0.110 | 0.996 | 1284 | tags=35%, list=18%, signal=43% |
| 35 | METALLOPEPTIDASE\_ACTIVITY |  | 18 | -0.52 | -1.57 | 0.027 | 0.107 | 0.996 | 937 | tags=39%, list=13%, signal=45% |
| 36 | TRANSFERASE\_ACTIVITY\_TRANSFERRING\_ACYL\_GROUPS |  | 21 | -0.52 | -1.56 | 0.023 | 0.114 | 0.998 | 882 | tags=33%, list=12%, signal=38% |
| 37 | INTEGRAL\_TO\_ORGANELLE\_MEMBRANE |  | 23 | -0.51 | -1.55 | 0.018 | 0.119 | 0.998 | 783 | tags=26%, list=11%, signal=29% |
| 38 | STRUCTURE\_SPECIFIC\_DNA\_BINDING |  | 18 | -0.52 | -1.55 | 0.033 | 0.121 | 0.999 | 2857 | tags=72%, list=40%, signal=121% |
| 39 | PROTEIN\_TARGETING |  | 43 | -0.44 | -1.54 | 0.011 | 0.127 | 0.999 | 1331 | tags=35%, list=19%, signal=43% |
| 40 | CARBOHYDRATE\_METABOLIC\_PROCESS |  | 53 | -0.40 | -1.53 | 0.023 | 0.129 | 0.999 | 430 | tags=23%, list=6%, signal=24% |
| 41 | CELLULAR\_COMPONENT\_DISASSEMBLY |  | 15 | -0.52 | -1.47 | 0.060 | 0.198 | 1.000 | 1041 | tags=40%, list=15%, signal=47% |
| 42 | TRANSFERASE\_ACTIVITY\_TRANSFERRING\_GROUPS\_OTHER\_THAN\_AMINO\_ACYL\_GROUPS |  | 17 | -0.51 | -1.46 | 0.071 | 0.207 | 1.000 | 882 | tags=35%, list=12%, signal=40% |
| 43 | ION\_TRANSMEMBRANE\_TRANSPORTER\_ACTIVITY |  | 70 | -0.37 | -1.46 | 0.036 | 0.211 | 1.000 | 1426 | tags=39%, list=20%, signal=48% |
| 44 | RIBOSOME |  | 16 | -0.52 | -1.45 | 0.039 | 0.210 | 1.000 | 2064 | tags=63%, list=29%, signal=88% |
| 45 | PROTEIN\_FOLDING |  | 21 | -0.47 | -1.44 | 0.077 | 0.223 | 1.000 | 2139 | tags=52%, list=30%, signal=75% |
| 46 | PROTEIN\_IMPORT |  | 23 | -0.46 | -1.43 | 0.085 | 0.229 | 1.000 | 1331 | tags=39%, list=19%, signal=48% |
| 47 | ORGANIC\_ACID\_METABOLIC\_PROCESS |  | 65 | -0.36 | -1.43 | 0.032 | 0.227 | 1.000 | 1868 | tags=43%, list=26%, signal=58% |
| 48 | INTRACELLULAR\_PROTEIN\_TRANSPORT |  | 58 | -0.37 | -1.42 | 0.048 | 0.244 | 1.000 | 1176 | tags=29%, list=17%, signal=35% |
| 49 | PROTEIN\_CATABOLIC\_PROCESS |  | 28 | -0.43 | -1.40 | 0.067 | 0.268 | 1.000 | 1700 | tags=36%, list=24%, signal=47% |
| 50 | CARBOXYLIC\_ACID\_METABOLIC\_PROCESS |  | 64 | -0.36 | -1.40 | 0.031 | 0.267 | 1.000 | 1868 | tags=42%, list=26%, signal=57% |
| 51 | INTRACELLULAR\_TRANSPORT |  | 116 | -0.32 | -1.39 | 0.025 | 0.268 | 1.000 | 1183 | tags=26%, list=17%, signal=31% |
| 52 | LYTIC\_VACUOLE |  | 21 | -0.46 | -1.39 | 0.101 | 0.266 | 1.000 | 1386 | tags=38%, list=20%, signal=47% |
| 53 | CATION\_TRANSMEMBRANE\_TRANSPORTER\_ACTIVITY |  | 53 | -0.37 | -1.39 | 0.050 | 0.262 | 1.000 | 1426 | tags=40%, list=20%, signal=49% |
| 54 | LYSOSOME |  | 21 | -0.46 | -1.39 | 0.085 | 0.264 | 1.000 | 1386 | tags=38%, list=20%, signal=47% |
| 55 | STRUCTURAL\_CONSTITUENT\_OF\_RIBOSOME |  | 16 | -0.49 | -1.36 | 0.126 | 0.307 | 1.000 | 2064 | tags=50%, list=29%, signal=70% |
| 56 | AROMATIC\_COMPOUND\_METABOLIC\_PROCESS |  | 15 | -0.48 | -1.36 | 0.135 | 0.309 | 1.000 | 1868 | tags=60%, list=26%, signal=81% |
| 57 | CELLULAR\_BIOSYNTHETIC\_PROCESS |  | 112 | -0.30 | -1.33 | 0.046 | 0.362 | 1.000 | 1585 | tags=32%, list=22%, signal=41% |
| 58 | LYASE\_ACTIVITY |  | 27 | -0.40 | -1.31 | 0.128 | 0.392 | 1.000 | 2111 | tags=63%, list=30%, signal=89% |
| 59 | NUCLEOBASENUCLEOSIDE\_AND\_NUCLEOTIDE\_METABOLIC\_PROCESS |  | 22 | -0.43 | -1.31 | 0.136 | 0.386 | 1.000 | 486 | tags=18%, list=7%, signal=19% |
| 60 | CELLULAR\_CARBOHYDRATE\_METABOLIC\_PROCESS |  | 38 | -0.38 | -1.31 | 0.133 | 0.382 | 1.000 | 1384 | tags=37%, list=20%, signal=46% |
| 61 | GOLGI\_APPARATUS\_PART |  | 39 | -0.37 | -1.31 | 0.125 | 0.378 | 1.000 | 1697 | tags=38%, list=24%, signal=50% |
| 62 | SUBSTRATE\_SPECIFIC\_TRANSMEMBRANE\_TRANSPORTER\_ACTIVITY |  | 96 | -0.31 | -1.30 | 0.095 | 0.402 | 1.000 | 1432 | tags=31%, list=20%, signal=39% |
| 63 | RESPONSE\_TO\_OXIDATIVE\_STRESS |  | 17 | -0.44 | -1.29 | 0.153 | 0.401 | 1.000 | 1355 | tags=47%, list=19%, signal=58% |
| 64 | PROTEIN\_TRANSPORT |  | 62 | -0.33 | -1.29 | 0.102 | 0.401 | 1.000 | 1176 | tags=27%, list=17%, signal=33% |
| 65 | CELLULAR\_PROTEIN\_CATABOLIC\_PROCESS |  | 26 | -0.40 | -1.29 | 0.143 | 0.405 | 1.000 | 2300 | tags=46%, list=32%, signal=68% |
| 66 | ACID\_AMINO\_ACID\_LIGASE\_ACTIVITY |  | 29 | -0.38 | -1.27 | 0.180 | 0.442 | 1.000 | 1270 | tags=28%, list=18%, signal=33% |
| 67 | AMINO\_ACID\_METABOLIC\_PROCESS |  | 32 | -0.37 | -1.26 | 0.157 | 0.455 | 1.000 | 1868 | tags=47%, list=26%, signal=63% |
| 68 | CELLULAR\_MACROMOLECULE\_CATABOLIC\_PROCESS |  | 41 | -0.35 | -1.26 | 0.172 | 0.459 | 1.000 | 1700 | tags=34%, list=24%, signal=45% |
| 69 | VACUOLE |  | 25 | -0.39 | -1.25 | 0.161 | 0.461 | 1.000 | 1386 | tags=32%, list=20%, signal=40% |
| 70 | LIPID\_METABOLIC\_PROCESS |  | 97 | -0.30 | -1.25 | 0.110 | 0.459 | 1.000 | 1115 | tags=26%, list=16%, signal=30% |
| 71 | PROTEIN\_COMPLEX |  | 291 | -0.26 | -1.25 | 0.051 | 0.466 | 1.000 | 1707 | tags=31%, list=24%, signal=39% |
| 72 | TRANSFERASE\_ACTIVITY\_TRANSFERRING\_HEXOSYL\_GROUPS |  | 27 | -0.38 | -1.24 | 0.186 | 0.465 | 1.000 | 741 | tags=19%, list=10%, signal=21% |
| 73 | GOLGI\_VESICLE\_TRANSPORT |  | 22 | -0.41 | -1.24 | 0.174 | 0.463 | 1.000 | 1646 | tags=41%, list=23%, signal=53% |
| 74 | TRANSMEMBRANE\_TRANSPORTER\_ACTIVITY |  | 106 | -0.29 | -1.23 | 0.120 | 0.480 | 1.000 | 1432 | tags=30%, list=20%, signal=37% |
| 75 | MACROMOLECULAR\_COMPLEX |  | 331 | -0.25 | -1.23 | 0.062 | 0.475 | 1.000 | 1707 | tags=30%, list=24%, signal=38% |
| 76 | NUCLEAR\_BODY |  | 15 | -0.43 | -1.23 | 0.216 | 0.475 | 1.000 | 1085 | tags=33%, list=15%, signal=39% |
| 77 | ESTABLISHMENT\_OF\_CELLULAR\_LOCALIZATION |  | 142 | -0.27 | -1.23 | 0.112 | 0.475 | 1.000 | 1183 | tags=24%, list=17%, signal=28% |
| 78 | RNA\_BINDING |  | 80 | -0.30 | -1.22 | 0.160 | 0.481 | 1.000 | 2107 | tags=36%, list=30%, signal=51% |
| 79 | LIGASE\_ACTIVITY\_FORMING\_CARBON\_NITROGEN\_BONDS |  | 36 | -0.35 | -1.22 | 0.192 | 0.492 | 1.000 | 1284 | tags=28%, list=18%, signal=34% |
| 80 | RNA\_PROCESSING |  | 62 | -0.31 | -1.21 | 0.170 | 0.495 | 1.000 | 1924 | tags=32%, list=27%, signal=44% |
| 81 | TRANSFERASE\_ACTIVITY\_TRANSFERRING\_GLYCOSYL\_GROUPS |  | 41 | -0.34 | -1.21 | 0.200 | 0.502 | 1.000 | 483 | tags=17%, list=7%, signal=18% |
| 82 | ESTABLISHMENT\_AND\_OR\_MAINTENANCE\_OF\_CHROMATIN\_ARCHITECTURE |  | 27 | -0.37 | -1.21 | 0.231 | 0.499 | 1.000 | 1516 | tags=37%, list=21%, signal=47% |
| 83 | RIBONUCLEOPROTEIN\_COMPLEX |  | 48 | -0.33 | -1.19 | 0.208 | 0.528 | 1.000 | 2064 | tags=40%, list=29%, signal=55% |
| 84 | MRNA\_PROCESSING\_GO\_0006397 |  | 27 | -0.37 | -1.18 | 0.250 | 0.543 | 1.000 | 1924 | tags=37%, list=27%, signal=51% |
| 85 | AMINO\_ACID\_AND\_DERIVATIVE\_METABOLIC\_PROCESS |  | 43 | -0.33 | -1.18 | 0.203 | 0.544 | 1.000 | 1868 | tags=42%, list=26%, signal=56% |
| 86 | BIOSYNTHETIC\_PROCESS |  | 151 | -0.27 | -1.18 | 0.147 | 0.539 | 1.000 | 1661 | tags=30%, list=23%, signal=38% |
| 87 | DNA\_REPAIR |  | 58 | -0.31 | -1.18 | 0.224 | 0.535 | 1.000 | 2281 | tags=45%, list=32%, signal=66% |
| 88 | ESTABLISHMENT\_OF\_PROTEIN\_LOCALIZATION |  | 70 | -0.29 | -1.18 | 0.214 | 0.532 | 1.000 | 1176 | tags=26%, list=17%, signal=31% |
| 89 | LIPID\_BINDING |  | 30 | -0.35 | -1.18 | 0.238 | 0.529 | 1.000 | 1190 | tags=33%, list=17%, signal=40% |
| 90 | PERINUCLEAR\_REGION\_OF\_CYTOPLASM |  | 21 | -0.38 | -1.15 | 0.275 | 0.585 | 1.000 | 690 | tags=19%, list=10%, signal=21% |
| 91 | SUBSTRATE\_SPECIFIC\_TRANSPORTER\_ACTIVITY |  | 116 | -0.27 | -1.15 | 0.187 | 0.587 | 1.000 | 1482 | tags=30%, list=21%, signal=38% |
| 92 | DNA\_METABOLIC\_PROCESS |  | 107 | -0.27 | -1.15 | 0.218 | 0.581 | 1.000 | 2033 | tags=38%, list=29%, signal=53% |
| 93 | CELLULAR\_LOCALIZATION |  | 147 | -0.25 | -1.15 | 0.196 | 0.584 | 1.000 | 1183 | tags=23%, list=17%, signal=27% |
| 94 | NUCLEOTIDE\_METABOLIC\_PROCESS |  | 18 | -0.39 | -1.14 | 0.299 | 0.610 | 1.000 | 239 | tags=11%, list=3%, signal=11% |
| 95 | ALCOHOL\_METABOLIC\_PROCESS |  | 35 | -0.33 | -1.13 | 0.288 | 0.628 | 1.000 | 1614 | tags=34%, list=23%, signal=44% |
| 96 | PROTEOLYSIS |  | 78 | -0.27 | -1.12 | 0.294 | 0.658 | 1.000 | 1087 | tags=22%, list=15%, signal=25% |
| 97 | PORE\_COMPLEX |  | 17 | -0.39 | -1.11 | 0.324 | 0.675 | 1.000 | 1617 | tags=35%, list=23%, signal=46% |
| 98 | PEPTIDASE\_ACTIVITY |  | 58 | -0.29 | -1.10 | 0.305 | 0.678 | 1.000 | 1018 | tags=21%, list=14%, signal=24% |
| 99 | GAMETE\_GENERATION |  | 30 | -0.33 | -1.10 | 0.352 | 0.675 | 1.000 | 1885 | tags=43%, list=27%, signal=59% |
| 100 | CHROMOSOME\_ORGANIZATION\_AND\_BIOGENESIS |  | 45 | -0.30 | -1.09 | 0.312 | 0.707 | 1.000 | 1516 | tags=31%, list=21%, signal=39% |
| 101 | ENDOMEMBRANE\_SYSTEM |  | 95 | -0.26 | -1.07 | 0.347 | 0.761 | 1.000 | 2008 | tags=34%, list=28%, signal=46% |
| 102 | PROTEIN\_OLIGOMERIZATION |  | 15 | -0.38 | -1.07 | 0.385 | 0.769 | 1.000 | 1257 | tags=40%, list=18%, signal=49% |
| 103 | PROTEIN\_LOCALIZATION |  | 76 | -0.27 | -1.06 | 0.352 | 0.775 | 1.000 | 1355 | tags=26%, list=19%, signal=32% |
| 104 | NUCLEOSIDE\_TRIPHOSPHATASE\_ACTIVITY |  | 88 | -0.26 | -1.06 | 0.345 | 0.768 | 1.000 | 2030 | tags=36%, list=29%, signal=50% |
| 105 | SENSORY\_PERCEPTION |  | 36 | -0.30 | -1.06 | 0.379 | 0.763 | 1.000 | 1265 | tags=31%, list=18%, signal=37% |
| 106 | NUCLEAR\_ENVELOPE\_ENDOPLASMIC\_RETICULUM\_NETWORK |  | 39 | -0.30 | -1.06 | 0.374 | 0.757 | 1.000 | 986 | tags=21%, list=14%, signal=24% |
| 107 | MACROMOLECULE\_LOCALIZATION |  | 84 | -0.25 | -1.06 | 0.370 | 0.766 | 1.000 | 1355 | tags=25%, list=19%, signal=31% |
| 108 | RIBONUCLEOPROTEIN\_COMPLEX\_BIOGENESIS\_AND\_ASSEMBLY |  | 21 | -0.34 | -1.05 | 0.374 | 0.767 | 1.000 | 1395 | tags=29%, list=20%, signal=35% |
| 109 | REGULATION\_OF\_DNA\_METABOLIC\_PROCESS |  | 18 | -0.36 | -1.05 | 0.361 | 0.760 | 1.000 | 2230 | tags=56%, list=31%, signal=81% |
| 110 | RESPONSE\_TO\_ENDOGENOUS\_STIMULUS |  | 84 | -0.26 | -1.05 | 0.357 | 0.753 | 1.000 | 1905 | tags=36%, list=27%, signal=48% |
| 111 | DNA\_RECOMBINATION |  | 17 | -0.36 | -1.05 | 0.394 | 0.763 | 1.000 | 1805 | tags=47%, list=25%, signal=63% |
| 112 | MRNA\_METABOLIC\_PROCESS |  | 32 | -0.31 | -1.05 | 0.391 | 0.762 | 1.000 | 1674 | tags=31%, list=24%, signal=41% |
| 113 | REGULATION\_OF\_BINDING |  | 20 | -0.35 | -1.05 | 0.425 | 0.755 | 1.000 | 610 | tags=25%, list=9%, signal=27% |
| 114 | ORGANELLE\_ORGANIZATION\_AND\_BIOGENESIS |  | 168 | -0.23 | -1.04 | 0.369 | 0.754 | 1.000 | 1548 | tags=26%, list=22%, signal=32% |
| 115 | TRANSPORT |  | 274 | -0.22 | -1.04 | 0.376 | 0.758 | 1.000 | 1586 | tags=26%, list=22%, signal=32% |
| 116 | PYROPHOSPHATASE\_ACTIVITY |  | 92 | -0.25 | -1.04 | 0.390 | 0.760 | 1.000 | 2030 | tags=36%, list=29%, signal=50% |
| 117 | HYDROLASE\_ACTIVITY\_ACTING\_ON\_ACID\_ANHYDRIDES |  | 92 | -0.25 | -1.04 | 0.400 | 0.756 | 1.000 | 2030 | tags=36%, list=29%, signal=50% |
| 118 | CELL\_CYCLE\_CHECKPOINT\_GO\_0000075 |  | 18 | -0.36 | -1.03 | 0.426 | 0.758 | 1.000 | 1894 | tags=44%, list=27%, signal=60% |
| 119 | ENZYME\_BINDING |  | 65 | -0.27 | -1.03 | 0.403 | 0.760 | 1.000 | 1063 | tags=25%, list=15%, signal=29% |
| 120 | CYTOSOL |  | 73 | -0.26 | -1.03 | 0.424 | 0.761 | 1.000 | 1776 | tags=32%, list=25%, signal=42% |
| 121 | SEXUAL\_REPRODUCTION |  | 35 | -0.30 | -1.03 | 0.444 | 0.757 | 1.000 | 1885 | tags=40%, list=27%, signal=54% |
| 122 | RESPONSE\_TO\_DNA\_DAMAGE\_STIMULUS |  | 70 | -0.26 | -1.02 | 0.437 | 0.766 | 1.000 | 1905 | tags=34%, list=27%, signal=46% |
| 123 | REGULATION\_OF\_TRANSCRIPTION\_FACTOR\_ACTIVITY |  | 15 | -0.37 | -1.02 | 0.448 | 0.774 | 1.000 | 610 | tags=27%, list=9%, signal=29% |
| 124 | NUCLEAR\_PART |  | 186 | -0.22 | -1.02 | 0.398 | 0.770 | 1.000 | 1811 | tags=30%, list=26%, signal=39% |
| 125 | DNA\_DIRECTED\_RNA\_POLYMERASEII\_HOLOENZYME |  | 17 | -0.35 | -1.01 | 0.418 | 0.777 | 1.000 | 2078 | tags=41%, list=29%, signal=58% |
| 126 | DEPHOSPHORYLATION |  | 22 | -0.33 | -1.01 | 0.465 | 0.771 | 1.000 | 758 | tags=23%, list=11%, signal=25% |
| 127 | ELECTRON\_TRANSPORT\_GO\_0006118 |  | 22 | -0.33 | -1.01 | 0.449 | 0.778 | 1.000 | 1778 | tags=45%, list=25%, signal=60% |
| 128 | PROTEIN\_RNA\_COMPLEX\_ASSEMBLY |  | 16 | -0.35 | -1.00 | 0.450 | 0.785 | 1.000 | 1395 | tags=31%, list=20%, signal=39% |
| 129 | REGULATION\_OF\_DNA\_BINDING |  | 17 | -0.34 | -1.00 | 0.455 | 0.794 | 1.000 | 610 | tags=24%, list=9%, signal=26% |
| 130 | REGULATION\_OF\_TRANSPORT |  | 19 | -0.34 | -1.00 | 0.458 | 0.793 | 1.000 | 227 | tags=11%, list=3%, signal=11% |
| 131 | UNFOLDED\_PROTEIN\_BINDING |  | 16 | -0.34 | -0.99 | 0.450 | 0.789 | 1.000 | 641 | tags=19%, list=9%, signal=21% |
| 132 | CELL\_CYCLE\_ARREST\_GO\_0007050 |  | 32 | -0.29 | -0.99 | 0.484 | 0.788 | 1.000 | 1145 | tags=25%, list=16%, signal=30% |
| 133 | LIPID\_BIOSYNTHETIC\_PROCESS |  | 25 | -0.31 | -0.99 | 0.478 | 0.786 | 1.000 | 1027 | tags=24%, list=14%, signal=28% |
| 134 | COATED\_VESICLE |  | 20 | -0.33 | -0.99 | 0.462 | 0.791 | 1.000 | 1697 | tags=35%, list=24%, signal=46% |
| 135 | TRANSLATION\_FACTOR\_ACTIVITY\_NUCLEIC\_ACID\_BINDING |  | 19 | -0.33 | -0.98 | 0.474 | 0.794 | 1.000 | 2604 | tags=53%, list=37%, signal=83% |
| 136 | ENDOPLASMIC\_RETICULUM\_MEMBRANE |  | 36 | -0.28 | -0.98 | 0.490 | 0.793 | 1.000 | 986 | tags=19%, list=14%, signal=22% |
| 137 | CELLULAR\_LIPID\_METABOLIC\_PROCESS |  | 78 | -0.24 | -0.98 | 0.494 | 0.800 | 1.000 | 1063 | tags=23%, list=15%, signal=27% |
| 138 | APOPTOTIC\_PROGRAM |  | 25 | -0.31 | -0.98 | 0.477 | 0.795 | 1.000 | 823 | tags=20%, list=12%, signal=23% |
| 139 | ESTABLISHMENT\_OF\_LOCALIZATION |  | 294 | -0.20 | -0.97 | 0.533 | 0.806 | 1.000 | 1586 | tags=24%, list=22%, signal=30% |
| 140 | SYNAPTIC\_TRANSMISSION |  | 38 | -0.28 | -0.97 | 0.485 | 0.802 | 1.000 | 1018 | tags=21%, list=14%, signal=24% |
| 141 | ENDOPLASMIC\_RETICULUM\_PART |  | 40 | -0.27 | -0.97 | 0.520 | 0.801 | 1.000 | 1008 | tags=20%, list=14%, signal=23% |
| 142 | NUCLEOLUS |  | 35 | -0.28 | -0.97 | 0.491 | 0.797 | 1.000 | 2302 | tags=43%, list=32%, signal=63% |
| 143 | NEUROLOGICAL\_SYSTEM\_PROCESS |  | 80 | -0.24 | -0.97 | 0.513 | 0.792 | 1.000 | 1265 | tags=24%, list=18%, signal=29% |
| 144 | ATPASE\_ACTIVITY |  | 46 | -0.27 | -0.97 | 0.505 | 0.787 | 1.000 | 1548 | tags=26%, list=22%, signal=33% |
| 145 | AMINE\_METABOLIC\_PROCESS |  | 58 | -0.25 | -0.97 | 0.537 | 0.787 | 1.000 | 460 | tags=16%, list=6%, signal=16% |
| 146 | CELLULAR\_PROTEIN\_METABOLIC\_PROCESS |  | 395 | -0.19 | -0.96 | 0.579 | 0.797 | 1.000 | 1440 | tags=22%, list=20%, signal=25% |
| 147 | MONOCARBOXYLIC\_ACID\_METABOLIC\_PROCESS |  | 27 | -0.29 | -0.96 | 0.521 | 0.801 | 1.000 | 995 | tags=22%, list=14%, signal=26% |
| 148 | PROTEIN\_N\_TERMINUS\_BINDING |  | 17 | -0.33 | -0.95 | 0.519 | 0.800 | 1.000 | 2230 | tags=53%, list=31%, signal=77% |
| 149 | TRANSLATION |  | 54 | -0.25 | -0.95 | 0.555 | 0.801 | 1.000 | 1395 | tags=26%, list=20%, signal=32% |
| 150 | CELLULAR\_MACROMOLECULE\_METABOLIC\_PROCESS |  | 398 | -0.19 | -0.95 | 0.614 | 0.796 | 1.000 | 1440 | tags=22%, list=20%, signal=26% |
| 151 | TRANSLATION\_REGULATOR\_ACTIVITY |  | 20 | -0.31 | -0.95 | 0.551 | 0.803 | 1.000 | 2604 | tags=50%, list=37%, signal=79% |
| 152 | FATTY\_ACID\_METABOLIC\_PROCESS |  | 21 | -0.31 | -0.94 | 0.541 | 0.805 | 1.000 | 2051 | tags=43%, list=29%, signal=60% |
| 153 | NITROGEN\_COMPOUND\_METABOLIC\_PROCESS |  | 63 | -0.24 | -0.94 | 0.538 | 0.804 | 1.000 | 537 | tags=16%, list=8%, signal=17% |
| 154 | NUCLEOTIDYLTRANSFERASE\_ACTIVITY |  | 21 | -0.30 | -0.94 | 0.529 | 0.804 | 1.000 | 2017 | tags=48%, list=28%, signal=66% |
| 155 | SMALL\_PROTEIN\_CONJUGATING\_ENZYME\_ACTIVITY |  | 25 | -0.29 | -0.94 | 0.547 | 0.801 | 1.000 | 1263 | tags=24%, list=18%, signal=29% |
| 156 | NUCLEAR\_LUMEN |  | 122 | -0.22 | -0.93 | 0.591 | 0.814 | 1.000 | 1857 | tags=32%, list=26%, signal=43% |
| 157 | TRANSITION\_METAL\_ION\_BINDING |  | 36 | -0.26 | -0.92 | 0.559 | 0.830 | 1.000 | 1684 | tags=31%, list=24%, signal=40% |
| 158 | NUCLEOBASENUCLEOSIDENUCLEOTIDE\_AND\_NUCLEIC\_ACID\_METABOLIC\_PROCESS |  | 447 | -0.18 | -0.92 | 0.725 | 0.833 | 1.000 | 1385 | tags=21%, list=20%, signal=24% |
| 159 | NUCLEAR\_PORE |  | 16 | -0.33 | -0.92 | 0.567 | 0.828 | 1.000 | 2008 | tags=38%, list=28%, signal=52% |
| 160 | HELICASE\_ACTIVITY |  | 19 | -0.31 | -0.92 | 0.590 | 0.827 | 1.000 | 1355 | tags=26%, list=19%, signal=32% |
| 161 | KINASE\_BINDING |  | 24 | -0.29 | -0.92 | 0.580 | 0.826 | 1.000 | 1189 | tags=29%, list=17%, signal=35% |
| 162 | UBIQUITIN\_PROTEIN\_LIGASE\_ACTIVITY |  | 23 | -0.29 | -0.91 | 0.596 | 0.829 | 1.000 | 1263 | tags=22%, list=18%, signal=26% |
| 163 | NUCLEAR\_ENVELOPE |  | 32 | -0.27 | -0.91 | 0.606 | 0.836 | 1.000 | 1380 | tags=25%, list=19%, signal=31% |
| 164 | GLYCOPROTEIN\_BIOSYNTHETIC\_PROCESS |  | 22 | -0.29 | -0.91 | 0.589 | 0.838 | 1.000 | 483 | tags=14%, list=7%, signal=15% |
| 165 | SECRETORY\_PATHWAY |  | 34 | -0.26 | -0.90 | 0.614 | 0.844 | 1.000 | 1646 | tags=35%, list=23%, signal=46% |
| 166 | ENDOPEPTIDASE\_ACTIVITY |  | 36 | -0.26 | -0.90 | 0.617 | 0.846 | 1.000 | 1196 | tags=22%, list=17%, signal=27% |
| 167 | MACROMOLECULE\_BIOSYNTHETIC\_PROCESS |  | 97 | -0.22 | -0.90 | 0.686 | 0.841 | 1.000 | 1395 | tags=23%, list=20%, signal=28% |
| 168 | PROTEIN\_METABOLIC\_PROCESS |  | 442 | -0.18 | -0.89 | 0.802 | 0.864 | 1.000 | 1440 | tags=21%, list=20%, signal=24% |
| 169 | SMALL\_CONJUGATING\_PROTEIN\_LIGASE\_ACTIVITY |  | 25 | -0.27 | -0.88 | 0.610 | 0.864 | 1.000 | 1263 | tags=20%, list=18%, signal=24% |
| 170 | GTPASE\_ACTIVITY |  | 41 | -0.24 | -0.88 | 0.644 | 0.872 | 1.000 | 2113 | tags=44%, list=30%, signal=62% |
| 171 | NUCLEASE\_ACTIVITY |  | 17 | -0.31 | -0.88 | 0.627 | 0.874 | 1.000 | 941 | tags=24%, list=13%, signal=27% |
| 172 | DNA\_PACKAGING |  | 18 | -0.30 | -0.87 | 0.631 | 0.872 | 1.000 | 1763 | tags=39%, list=25%, signal=52% |
| 173 | NUCLEAR\_MEMBRANE\_PART |  | 20 | -0.29 | -0.87 | 0.620 | 0.869 | 1.000 | 1617 | tags=30%, list=23%, signal=39% |
| 174 | GOLGI\_APPARATUS |  | 86 | -0.21 | -0.87 | 0.732 | 0.882 | 1.000 | 1697 | tags=29%, list=24%, signal=38% |
| 175 | MEMBRANE\_ORGANIZATION\_AND\_BIOGENESIS |  | 50 | -0.23 | -0.86 | 0.685 | 0.887 | 1.000 | 1323 | tags=24%, list=19%, signal=29% |
| 176 | NUCLEAR\_TRANSPORT |  | 35 | -0.25 | -0.86 | 0.679 | 0.885 | 1.000 | 920 | tags=17%, list=13%, signal=20% |
| 177 | NEGATIVE\_REGULATION\_OF\_RNA\_METABOLIC\_PROCESS |  | 41 | -0.24 | -0.86 | 0.695 | 0.885 | 1.000 | 1107 | tags=20%, list=16%, signal=23% |
| 178 | NUCLEOCYTOPLASMIC\_TRANSPORT |  | 35 | -0.25 | -0.86 | 0.699 | 0.882 | 1.000 | 920 | tags=17%, list=13%, signal=20% |
| 179 | MONOVALENT\_INORGANIC\_CATION\_TRANSPORT |  | 17 | -0.29 | -0.84 | 0.683 | 0.902 | 1.000 | 1785 | tags=35%, list=25%, signal=47% |
| 180 | NUCLEOPLASM |  | 91 | -0.20 | -0.84 | 0.775 | 0.898 | 1.000 | 1763 | tags=30%, list=25%, signal=39% |
| 181 | NEGATIVE\_REGULATION\_OF\_TRANSCRIPTION\_DNA\_DEPENDENT |  | 41 | -0.24 | -0.84 | 0.734 | 0.904 | 1.000 | 1107 | tags=20%, list=16%, signal=23% |
| 182 | HYDROLASE\_ACTIVITY\_ACTING\_ON\_CARBON\_NITROGEN\_NOT\_PEPTIDEBONDS |  | 20 | -0.28 | -0.84 | 0.712 | 0.902 | 1.000 | 2200 | tags=50%, list=31%, signal=72% |
| 183 | CHROMOSOME |  | 41 | -0.23 | -0.83 | 0.762 | 0.920 | 1.000 | 1596 | tags=24%, list=23%, signal=31% |
| 184 | REPRODUCTION |  | 69 | -0.21 | -0.82 | 0.791 | 0.919 | 1.000 | 1885 | tags=33%, list=27%, signal=45% |
| 185 | SECRETION\_BY\_CELL |  | 41 | -0.23 | -0.82 | 0.759 | 0.918 | 1.000 | 1646 | tags=32%, list=23%, signal=41% |
| 186 | ZINC\_ION\_BINDING |  | 30 | -0.25 | -0.82 | 0.727 | 0.913 | 1.000 | 1684 | tags=33%, list=24%, signal=44% |
| 187 | POSITIVE\_REGULATION\_OF\_PROTEIN\_METABOLIC\_PROCESS |  | 24 | -0.26 | -0.81 | 0.758 | 0.935 | 1.000 | 1395 | tags=33%, list=20%, signal=41% |
| 188 | SUBSTRATE\_SPECIFIC\_CHANNEL\_ACTIVITY |  | 25 | -0.25 | -0.81 | 0.731 | 0.931 | 1.000 | 1423 | tags=32%, list=20%, signal=40% |
| 189 | RNA\_SPLICING |  | 34 | -0.23 | -0.81 | 0.765 | 0.927 | 1.000 | 1689 | tags=26%, list=24%, signal=35% |
| 190 | RHODOPSIN\_LIKE\_RECEPTOR\_ACTIVITY |  | 17 | -0.28 | -0.81 | 0.747 | 0.923 | 1.000 | 1116 | tags=24%, list=16%, signal=28% |
| 191 | ION\_CHANNEL\_ACTIVITY |  | 24 | -0.26 | -0.80 | 0.751 | 0.923 | 1.000 | 1423 | tags=33%, list=20%, signal=42% |
| 192 | NUCLEOPLASM\_PART |  | 69 | -0.20 | -0.77 | 0.846 | 0.963 | 1.000 | 1763 | tags=29%, list=25%, signal=38% |
| 193 | TRANSMISSION\_OF\_NERVE\_IMPULSE |  | 42 | -0.21 | -0.77 | 0.830 | 0.963 | 1.000 | 1018 | tags=19%, list=14%, signal=22% |
| 194 | TRANSFERASE\_ACTIVITY\_TRANSFERRING\_PHOSPHORUS\_CONTAINING\_GROUPS |  | 176 | -0.17 | -0.77 | 0.919 | 0.959 | 1.000 | 1669 | tags=22%, list=24%, signal=28% |
| 195 | INTERPHASE |  | 28 | -0.24 | -0.77 | 0.789 | 0.955 | 1.000 | 1145 | tags=21%, list=16%, signal=25% |
| 196 | PROTEIN\_COMPLEX\_ASSEMBLY |  | 71 | -0.19 | -0.77 | 0.862 | 0.957 | 1.000 | 1778 | tags=30%, list=25%, signal=39% |
| 197 | NEGATIVE\_REGULATION\_OF\_CELL\_CYCLE |  | 36 | -0.21 | -0.75 | 0.838 | 0.966 | 1.000 | 1145 | tags=22%, list=16%, signal=26% |
| 198 | INTRACELLULAR\_NON\_MEMBRANE\_BOUND\_ORGANELLE |  | 197 | -0.16 | -0.75 | 0.959 | 0.965 | 1.000 | 499 | tags=9%, list=7%, signal=10% |
| 199 | NON\_MEMBRANE\_BOUND\_ORGANELLE |  | 197 | -0.16 | -0.75 | 0.976 | 0.963 | 1.000 | 499 | tags=9%, list=7%, signal=10% |
| 200 | DNA\_REPLICATION |  | 39 | -0.21 | -0.74 | 0.871 | 0.974 | 1.000 | 1192 | tags=21%, list=17%, signal=25% |
| 201 | DNA\_DEPENDENT\_DNA\_REPLICATION |  | 20 | -0.25 | -0.74 | 0.838 | 0.971 | 1.000 | 1085 | tags=20%, list=15%, signal=24% |
| 202 | PROTEIN\_IMPORT\_INTO\_NUCLEUS |  | 18 | -0.25 | -0.73 | 0.825 | 0.973 | 1.000 | 812 | tags=17%, list=11%, signal=19% |
| 203 | GUANYL\_NUCLEOTIDE\_BINDING |  | 19 | -0.24 | -0.72 | 0.828 | 0.974 | 1.000 | 1992 | tags=42%, list=28%, signal=58% |
| 204 | GTP\_BINDING |  | 19 | -0.24 | -0.72 | 0.859 | 0.972 | 1.000 | 1992 | tags=42%, list=28%, signal=58% |
| 205 | NUCLEAR\_IMPORT |  | 19 | -0.24 | -0.72 | 0.820 | 0.968 | 1.000 | 812 | tags=16%, list=11%, signal=18% |
| 206 | SECRETION |  | 56 | -0.19 | -0.72 | 0.910 | 0.969 | 1.000 | 1500 | tags=25%, list=21%, signal=31% |
| 207 | CHROMOSOMAL\_PART |  | 36 | -0.20 | -0.71 | 0.884 | 0.974 | 1.000 | 1596 | tags=22%, list=23%, signal=29% |
| 208 | KINASE\_ACTIVITY |  | 151 | -0.16 | -0.70 | 0.979 | 0.971 | 1.000 | 1669 | tags=21%, list=24%, signal=26% |
| 209 | ENDOPLASMIC\_RETICULUM |  | 107 | -0.16 | -0.70 | 0.963 | 0.969 | 1.000 | 1027 | tags=12%, list=14%, signal=14% |
| 210 | REGULATION\_OF\_CELLULAR\_PROTEIN\_METABOLIC\_PROCESS |  | 52 | -0.19 | -0.69 | 0.931 | 0.971 | 1.000 | 1395 | tags=23%, list=20%, signal=29% |
| 211 | MICROTUBULE\_BASED\_PROCESS |  | 21 | -0.22 | -0.69 | 0.884 | 0.970 | 1.000 | 770 | tags=14%, list=11%, signal=16% |
| 212 | ATPASE\_ACTIVITY\_COUPLED |  | 37 | -0.20 | -0.68 | 0.902 | 0.972 | 1.000 | 1886 | tags=27%, list=27%, signal=37% |
| 213 | SPLICEOSOME |  | 16 | -0.24 | -0.67 | 0.872 | 0.978 | 1.000 | 1155 | tags=19%, list=16%, signal=22% |
| 214 | GLYCOPROTEIN\_METABOLIC\_PROCESS |  | 29 | -0.20 | -0.66 | 0.932 | 0.981 | 1.000 | 483 | tags=10%, list=7%, signal=11% |
| 215 | NEGATIVE\_REGULATION\_OF\_TRANSCRIPTION\_FROM\_RNA\_POLYMERASE\_II\_PROMOTER |  | 25 | -0.20 | -0.65 | 0.929 | 0.980 | 1.000 | 1107 | tags=16%, list=16%, signal=19% |
| 216 | REGULATION\_OF\_PROTEIN\_MODIFICATION\_PROCESS |  | 17 | -0.23 | -0.65 | 0.910 | 0.976 | 1.000 | 1358 | tags=29%, list=19%, signal=36% |
| 217 | GOLGI\_MEMBRANE |  | 18 | -0.22 | -0.63 | 0.913 | 0.979 | 1.000 | 1697 | tags=28%, list=24%, signal=36% |
| 218 | REGULATION\_OF\_TRANSLATION |  | 27 | -0.19 | -0.62 | 0.951 | 0.982 | 1.000 | 1395 | tags=22%, list=20%, signal=28% |
| 219 | SEQUENCE\_SPECIFIC\_DNA\_BINDING |  | 17 | -0.21 | -0.60 | 0.950 | 0.985 | 1.000 | 2579 | tags=53%, list=36%, signal=83% |
| 220 | CYSTEINE\_TYPE\_PEPTIDASE\_ACTIVITY |  | 16 | -0.18 | -0.53 | 0.977 | 0.996 | 1.000 | 1417 | tags=19%, list=20%, signal=23% |
| 221 | TRANSMEMBRANE\_RECEPTOR\_PROTEIN\_TYROSINE\_KINASE\_ACTIVITY |  | 17 | -0.17 | -0.50 | 0.988 | 0.995 | 1.000 | 2150 | tags=29%, list=30%, signal=42% |
Table: Gene sets enriched in phenotype **na **[plain text format]****
