# Supplementary material for: Network-based integration of molecular and physiological data elucidates regulatory mechanisms underlying adaptation to high-fat diet
Source: Genes Nutr. 2015 May 28;10(4):22. doi: 10.1007/s12263-015-0470-6 (PMC4446272; doi:10.1007/s12263-015-0470-6)
Supplement: Supplementary file 4 — Supplementary material 4 (ZIP 6984 kb) [file 12263_2015_470_MOESM4_ESM.zip › HF LF 5 d GSEA result/gsea_report_for_na_pos_1365164922288.html]

Report for na\_pos 1365164922288 [GSEA]

| GS  follow link to MSigDB | GS DETAILS | SIZE | ES | NES | NOM p-val | FDR q-val | FWER p-val | RANK AT MAX | LEADING EDGE || 1 | EXTRACELLULAR\_MATRIX | Details ... | 34 | 0.62 | 2.30 | 0.000 | 0.002 | 0.001 | 1033 | tags=50%, list=15%, signal=58% |
| 2 | PROTEINACEOUS\_EXTRACELLULAR\_MATRIX | Details ... | 34 | 0.62 | 2.27 | 0.000 | 0.002 | 0.002 | 1033 | tags=50%, list=15%, signal=58% |
| 3 | CELLULAR\_CATION\_HOMEOSTASIS | Details ... | 35 | 0.58 | 2.20 | 0.000 | 0.003 | 0.006 | 1190 | tags=49%, list=17%, signal=58% |
| 4 | CATION\_HOMEOSTASIS | Details ... | 35 | 0.58 | 2.18 | 0.000 | 0.003 | 0.008 | 1190 | tags=49%, list=17%, signal=58% |
| 5 | CELLULAR\_DEFENSE\_RESPONSE | Details ... | 19 | 0.68 | 2.14 | 0.000 | 0.005 | 0.014 | 1136 | tags=47%, list=16%, signal=56% |
| 6 | EXTRACELLULAR\_MATRIX\_PART | Details ... | 22 | 0.63 | 2.14 | 0.000 | 0.004 | 0.016 | 984 | tags=50%, list=14%, signal=58% |
| 7 | REGULATION\_OF\_MAP\_KINASE\_ACTIVITY | Details ... | 28 | 0.60 | 2.13 | 0.000 | 0.004 | 0.017 | 1237 | tags=54%, list=17%, signal=65% |
| 8 | CHEMOKINE\_RECEPTOR\_BINDING | Details ... | 17 | 0.68 | 2.11 | 0.000 | 0.004 | 0.018 | 986 | tags=53%, list=14%, signal=61% |
| 9 | EXTRACELLULAR\_REGION\_PART | Details ... | 116 | 0.43 | 2.06 | 0.000 | 0.009 | 0.048 | 1258 | tags=36%, list=18%, signal=43% |
| 10 | DEFENSE\_RESPONSE | Details ... | 73 | 0.45 | 2.02 | 0.000 | 0.012 | 0.071 | 2015 | tags=51%, list=28%, signal=70% |
| 11 | CHEMOKINE\_ACTIVITY | Details ... | 16 | 0.66 | 2.01 | 0.000 | 0.012 | 0.077 | 986 | tags=50%, list=14%, signal=58% |
| 12 | LOCOMOTORY\_BEHAVIOR | Details ... | 36 | 0.53 | 1.99 | 0.000 | 0.013 | 0.090 | 1191 | tags=42%, list=17%, signal=50% |
| 13 | AMINE\_TRANSPORT | Details ... | 15 | 0.65 | 1.98 | 0.002 | 0.013 | 0.099 | 2097 | tags=73%, list=30%, signal=104% |
| 14 | G\_PROTEIN\_COUPLED\_RECEPTOR\_BINDING | Details ... | 20 | 0.61 | 1.96 | 0.003 | 0.016 | 0.126 | 986 | tags=50%, list=14%, signal=58% |
| 15 | POSITIVE\_REGULATION\_OF\_MAP\_KINASE\_ACTIVITY | Details ... | 22 | 0.57 | 1.94 | 0.003 | 0.018 | 0.156 | 1237 | tags=50%, list=17%, signal=60% |
| 16 | BEHAVIOR | Details ... | 45 | 0.50 | 1.93 | 0.000 | 0.018 | 0.160 | 1191 | tags=38%, list=17%, signal=45% |
| 17 | CELL\_SURFACE\_RECEPTOR\_LINKED\_SIGNAL\_TRANSDUCTION\_GO\_0007166 | Details ... | 179 | 0.37 | 1.91 | 0.000 | 0.020 | 0.190 | 1445 | tags=35%, list=20%, signal=43% |
| 18 | PROTEIN\_KINASE\_CASCADE | Details ... | 108 | 0.40 | 1.91 | 0.000 | 0.019 | 0.191 | 1354 | tags=39%, list=19%, signal=47% |
| 19 | EXTRACELLULAR\_REGION | Details ... | 145 | 0.38 | 1.90 | 0.000 | 0.020 | 0.210 | 1285 | tags=35%, list=18%, signal=42% |
| 20 | RESPONSE\_TO\_EXTERNAL\_STIMULUS | Details ... | 101 | 0.40 | 1.90 | 0.000 | 0.020 | 0.220 | 2015 | tags=51%, list=28%, signal=71% |
| 21 | CELL\_PROJECTION |  | 38 | 0.50 | 1.89 | 0.003 | 0.021 | 0.237 | 1500 | tags=42%, list=21%, signal=53% |
| 22 | RESPONSE\_TO\_WOUNDING |  | 65 | 0.44 | 1.88 | 0.003 | 0.021 | 0.244 | 1696 | tags=46%, list=24%, signal=60% |
| 23 | MAPKKK\_CASCADE\_GO\_0000165 |  | 35 | 0.50 | 1.87 | 0.000 | 0.021 | 0.261 | 1237 | tags=46%, list=17%, signal=55% |
| 24 | RESPONSE\_TO\_OTHER\_ORGANISM |  | 27 | 0.52 | 1.86 | 0.005 | 0.023 | 0.287 | 863 | tags=44%, list=12%, signal=50% |
| 25 | IMMUNE\_SYSTEM\_PROCESS |  | 127 | 0.38 | 1.85 | 0.000 | 0.026 | 0.323 | 2243 | tags=55%, list=32%, signal=79% |
| 26 | RECEPTOR\_COMPLEX |  | 15 | 0.62 | 1.83 | 0.005 | 0.028 | 0.360 | 1824 | tags=67%, list=26%, signal=90% |
| 27 | PLASMA\_MEMBRANE\_PART |  | 322 | 0.32 | 1.82 | 0.000 | 0.032 | 0.409 | 1708 | tags=38%, list=24%, signal=47% |
| 28 | ANATOMICAL\_STRUCTURE\_FORMATION |  | 24 | 0.53 | 1.80 | 0.002 | 0.034 | 0.440 | 2275 | tags=63%, list=32%, signal=92% |
| 29 | ANGIOGENESIS |  | 23 | 0.52 | 1.80 | 0.003 | 0.034 | 0.445 | 2275 | tags=61%, list=32%, signal=89% |
| 30 | CELL\_CELL\_ADHESION |  | 28 | 0.49 | 1.79 | 0.005 | 0.037 | 0.492 | 1935 | tags=54%, list=27%, signal=73% |
| 31 | RESPONSE\_TO\_BIOTIC\_STIMULUS |  | 40 | 0.45 | 1.77 | 0.000 | 0.039 | 0.524 | 863 | tags=35%, list=12%, signal=40% |
| 32 | MULTICELLULAR\_ORGANISMAL\_DEVELOPMENT |  | 287 | 0.32 | 1.77 | 0.000 | 0.039 | 0.538 | 1690 | tags=36%, list=24%, signal=45% |
| 33 | STRESS\_ACTIVATED\_PROTEIN\_KINASE\_SIGNALING\_PATHWAY |  | 15 | 0.58 | 1.76 | 0.018 | 0.039 | 0.547 | 1112 | tags=47%, list=16%, signal=55% |
| 34 | COAGULATION |  | 19 | 0.54 | 1.75 | 0.014 | 0.041 | 0.573 | 1477 | tags=47%, list=21%, signal=60% |
| 35 | ORGAN\_MORPHOGENESIS |  | 50 | 0.43 | 1.75 | 0.003 | 0.040 | 0.574 | 2275 | tags=54%, list=32%, signal=79% |
| 36 | GROWTH |  | 26 | 0.49 | 1.75 | 0.005 | 0.040 | 0.589 | 1623 | tags=50%, list=23%, signal=65% |
| 37 | CYTOKINE\_ACTIVITY |  | 29 | 0.49 | 1.74 | 0.007 | 0.042 | 0.613 | 1696 | tags=55%, list=24%, signal=72% |
| 38 | INTRACELLULAR\_SIGNALING\_CASCADE |  | 240 | 0.32 | 1.74 | 0.000 | 0.041 | 0.613 | 1398 | tags=33%, list=20%, signal=40% |
| 39 | BLOOD\_COAGULATION |  | 19 | 0.54 | 1.74 | 0.013 | 0.040 | 0.620 | 1477 | tags=47%, list=21%, signal=60% |
| 40 | HEMOSTASIS |  | 20 | 0.55 | 1.74 | 0.010 | 0.039 | 0.621 | 1477 | tags=50%, list=21%, signal=63% |
| 41 | INTEGRAL\_TO\_PLASMA\_MEMBRANE |  | 258 | 0.32 | 1.74 | 0.000 | 0.039 | 0.624 | 1666 | tags=38%, list=23%, signal=47% |
| 42 | ANATOMICAL\_STRUCTURE\_MORPHOGENESIS |  | 119 | 0.36 | 1.74 | 0.000 | 0.039 | 0.633 | 2275 | tags=49%, list=32%, signal=71% |
| 43 | INTRINSIC\_TO\_PLASMA\_MEMBRANE |  | 266 | 0.32 | 1.73 | 0.000 | 0.039 | 0.640 | 1666 | tags=38%, list=23%, signal=47% |
| 44 | PLASMA\_MEMBRANE |  | 407 | 0.31 | 1.73 | 0.000 | 0.039 | 0.655 | 1708 | tags=37%, list=24%, signal=46% |
| 45 | WOUND\_HEALING |  | 23 | 0.50 | 1.71 | 0.007 | 0.046 | 0.716 | 1571 | tags=48%, list=22%, signal=61% |
| 46 | JNK\_CASCADE |  | 15 | 0.58 | 1.70 | 0.014 | 0.048 | 0.737 | 1112 | tags=47%, list=16%, signal=55% |
| 47 | REGULATION\_OF\_PROTEIN\_KINASE\_ACTIVITY |  | 61 | 0.39 | 1.70 | 0.006 | 0.048 | 0.750 | 1237 | tags=34%, list=17%, signal=41% |
| 48 | CELLULAR\_HOMEOSTASIS |  | 46 | 0.42 | 1.69 | 0.003 | 0.050 | 0.772 | 1190 | tags=41%, list=17%, signal=49% |
| 49 | CELL\_PROLIFERATION\_GO\_0008283 |  | 168 | 0.33 | 1.69 | 0.000 | 0.050 | 0.782 | 1729 | tags=38%, list=24%, signal=49% |
| 50 | CYTOSKELETON\_ORGANIZATION\_AND\_BIOGENESIS |  | 66 | 0.39 | 1.69 | 0.009 | 0.050 | 0.783 | 1891 | tags=47%, list=27%, signal=63% |
| 51 | ION\_HOMEOSTASIS |  | 42 | 0.43 | 1.68 | 0.003 | 0.052 | 0.809 | 1190 | tags=43%, list=17%, signal=51% |
| 52 | PROTEIN\_DNA\_COMPLEX\_ASSEMBLY |  | 20 | 0.52 | 1.68 | 0.010 | 0.052 | 0.819 | 855 | tags=30%, list=12%, signal=34% |
| 53 | REGULATION\_OF\_CATALYTIC\_ACTIVITY |  | 104 | 0.36 | 1.67 | 0.004 | 0.052 | 0.830 | 1389 | tags=34%, list=20%, signal=41% |
| 54 | REGULATION\_OF\_BIOLOGICAL\_QUALITY |  | 152 | 0.33 | 1.67 | 0.000 | 0.054 | 0.842 | 1477 | tags=38%, list=21%, signal=47% |
| 55 | REGULATION\_OF\_CELL\_GROWTH |  | 17 | 0.54 | 1.66 | 0.021 | 0.056 | 0.857 | 1623 | tags=59%, list=23%, signal=76% |
| 56 | ACTIN\_CYTOSKELETON |  | 48 | 0.41 | 1.66 | 0.005 | 0.057 | 0.863 | 2007 | tags=50%, list=28%, signal=69% |
| 57 | G\_PROTEIN\_COUPLED\_RECEPTOR\_PROTEIN\_SIGNALING\_PATHWAY |  | 82 | 0.36 | 1.66 | 0.014 | 0.056 | 0.863 | 1398 | tags=34%, list=20%, signal=42% |
| 58 | REGULATION\_OF\_TRANSFERASE\_ACTIVITY |  | 63 | 0.39 | 1.66 | 0.003 | 0.056 | 0.866 | 1237 | tags=33%, list=17%, signal=40% |
| 59 | ACTIVATION\_OF\_MAPK\_ACTIVITY |  | 16 | 0.55 | 1.66 | 0.017 | 0.055 | 0.866 | 281 | tags=31%, list=4%, signal=32% |
| 60 | CYTOKINE\_PRODUCTION |  | 23 | 0.48 | 1.64 | 0.015 | 0.059 | 0.888 | 1260 | tags=48%, list=18%, signal=58% |
| 61 | REGULATION\_OF\_KINASE\_ACTIVITY |  | 63 | 0.39 | 1.64 | 0.003 | 0.061 | 0.899 | 1237 | tags=33%, list=17%, signal=40% |
| 62 | RHO\_PROTEIN\_SIGNAL\_TRANSDUCTION |  | 20 | 0.50 | 1.63 | 0.017 | 0.061 | 0.904 | 1378 | tags=40%, list=19%, signal=50% |
| 63 | RECEPTOR\_ACTIVITY |  | 141 | 0.33 | 1.63 | 0.000 | 0.061 | 0.910 | 1707 | tags=38%, list=24%, signal=49% |
| 64 | ACTIN\_FILAMENT\_BASED\_PROCESS |  | 37 | 0.43 | 1.63 | 0.005 | 0.062 | 0.916 | 1891 | tags=43%, list=27%, signal=59% |
| 65 | RECEPTOR\_BINDING |  | 116 | 0.34 | 1.63 | 0.000 | 0.062 | 0.918 | 1739 | tags=41%, list=25%, signal=53% |
| 66 | REGULATION\_OF\_GROWTH |  | 20 | 0.50 | 1.63 | 0.021 | 0.061 | 0.920 | 1623 | tags=55%, list=23%, signal=71% |
| 67 | CELL\_SURFACE |  | 25 | 0.46 | 1.62 | 0.020 | 0.063 | 0.928 | 1640 | tags=60%, list=23%, signal=78% |
| 68 | CELL\_MIGRATION |  | 28 | 0.46 | 1.62 | 0.018 | 0.063 | 0.929 | 1442 | tags=54%, list=20%, signal=67% |
| 69 | INFLAMMATORY\_RESPONSE |  | 39 | 0.42 | 1.62 | 0.005 | 0.063 | 0.933 | 2015 | tags=54%, list=28%, signal=75% |
| 70 | RAS\_PROTEIN\_SIGNAL\_TRANSDUCTION |  | 34 | 0.44 | 1.61 | 0.010 | 0.065 | 0.939 | 1378 | tags=38%, list=19%, signal=47% |
| 71 | POSITIVE\_REGULATION\_OF\_MULTICELLULAR\_ORGANISMAL\_PROCESS |  | 19 | 0.51 | 1.61 | 0.021 | 0.065 | 0.943 | 1398 | tags=53%, list=20%, signal=65% |
| 72 | TRANSMEMBRANE\_RECEPTOR\_PROTEIN\_TYROSINE\_KINASE\_SIGNALING\_PATHWAY |  | 26 | 0.46 | 1.60 | 0.021 | 0.066 | 0.949 | 666 | tags=31%, list=9%, signal=34% |
| 73 | REGULATION\_OF\_CELL\_DIFFERENTIATION |  | 24 | 0.46 | 1.60 | 0.021 | 0.068 | 0.957 | 974 | tags=38%, list=14%, signal=43% |
| 74 | ORGAN\_DEVELOPMENT |  | 169 | 0.31 | 1.60 | 0.000 | 0.068 | 0.960 | 1690 | tags=34%, list=24%, signal=44% |
| 75 | MOLECULAR\_ADAPTOR\_ACTIVITY |  | 18 | 0.50 | 1.60 | 0.032 | 0.068 | 0.961 | 2278 | tags=67%, list=32%, signal=98% |
| 76 | ANATOMICAL\_STRUCTURE\_DEVELOPMENT |  | 280 | 0.30 | 1.59 | 0.000 | 0.067 | 0.961 | 1696 | tags=33%, list=24%, signal=42% |
| 77 | VASCULATURE\_DEVELOPMENT |  | 25 | 0.46 | 1.59 | 0.017 | 0.069 | 0.965 | 2392 | tags=60%, list=34%, signal=90% |
| 78 | ACTIN\_BINDING |  | 33 | 0.43 | 1.58 | 0.018 | 0.071 | 0.970 | 2015 | tags=55%, list=28%, signal=76% |
| 79 | CYTOSKELETAL\_PROTEIN\_BINDING |  | 60 | 0.38 | 1.58 | 0.009 | 0.072 | 0.972 | 2053 | tags=53%, list=29%, signal=74% |
| 80 | ACTIN\_CYTOSKELETON\_ORGANIZATION\_AND\_BIOGENESIS |  | 33 | 0.42 | 1.58 | 0.024 | 0.072 | 0.974 | 688 | tags=24%, list=10%, signal=27% |
| 81 | POSITIVE\_REGULATION\_OF\_TRANSFERASE\_ACTIVITY |  | 37 | 0.41 | 1.58 | 0.015 | 0.072 | 0.974 | 1237 | tags=35%, list=17%, signal=42% |
| 82 | PROTEIN\_COMPLEX\_BINDING |  | 22 | 0.47 | 1.58 | 0.038 | 0.071 | 0.974 | 1585 | tags=45%, list=22%, signal=58% |
| 83 | REGULATION\_OF\_MOLECULAR\_FUNCTION |  | 122 | 0.32 | 1.57 | 0.004 | 0.073 | 0.980 | 1389 | tags=33%, list=20%, signal=40% |
| 84 | EXTRACELLULAR\_SPACE |  | 83 | 0.34 | 1.56 | 0.006 | 0.080 | 0.986 | 1174 | tags=30%, list=17%, signal=36% |
| 85 | IMMUNE\_RESPONSE |  | 87 | 0.34 | 1.55 | 0.012 | 0.080 | 0.986 | 2243 | tags=52%, list=32%, signal=75% |
| 86 | RESPONSE\_TO\_VIRUS |  | 20 | 0.48 | 1.55 | 0.028 | 0.081 | 0.989 | 632 | tags=35%, list=9%, signal=38% |
| 87 | POSITIVE\_REGULATION\_OF\_SIGNAL\_TRANSDUCTION |  | 51 | 0.37 | 1.55 | 0.019 | 0.082 | 0.989 | 1573 | tags=37%, list=22%, signal=48% |
| 88 | SYSTEM\_DEVELOPMENT |  | 237 | 0.29 | 1.54 | 0.000 | 0.084 | 0.990 | 1690 | tags=33%, list=24%, signal=42% |
| 89 | REGULATION\_OF\_MULTICELLULAR\_ORGANISMAL\_PROCESS |  | 41 | 0.38 | 1.54 | 0.008 | 0.085 | 0.991 | 1935 | tags=61%, list=27%, signal=83% |
| 90 | HEMOPOIESIS |  | 35 | 0.41 | 1.54 | 0.022 | 0.085 | 0.991 | 1654 | tags=46%, list=23%, signal=59% |
| 91 | NEGATIVE\_REGULATION\_OF\_CELL\_PROLIFERATION |  | 47 | 0.38 | 1.53 | 0.025 | 0.086 | 0.991 | 1869 | tags=47%, list=26%, signal=63% |
| 92 | MULTI\_ORGANISM\_PROCESS |  | 45 | 0.38 | 1.53 | 0.008 | 0.087 | 0.993 | 2015 | tags=56%, list=28%, signal=77% |
| 93 | TRANSMEMBRANE\_RECEPTOR\_ACTIVITY |  | 86 | 0.34 | 1.52 | 0.006 | 0.090 | 0.994 | 1401 | tags=31%, list=20%, signal=39% |
| 94 | LEADING\_EDGE |  | 20 | 0.48 | 1.52 | 0.032 | 0.089 | 0.994 | 1372 | tags=30%, list=19%, signal=37% |
| 95 | PROTEIN\_TYROSINE\_PHOSPHATASE\_ACTIVITY |  | 19 | 0.47 | 1.52 | 0.035 | 0.089 | 0.995 | 1367 | tags=42%, list=19%, signal=52% |
| 96 | REGULATION\_OF\_CELL\_PROLIFERATION |  | 99 | 0.33 | 1.52 | 0.013 | 0.088 | 0.995 | 1782 | tags=39%, list=25%, signal=52% |
| 97 | RECEPTOR\_SIGNALING\_PROTEIN\_ACTIVITY |  | 33 | 0.41 | 1.52 | 0.033 | 0.089 | 0.996 | 457 | tags=27%, list=6%, signal=29% |
| 98 | POSITIVE\_REGULATION\_OF\_BIOLOGICAL\_PROCESS |  | 241 | 0.28 | 1.52 | 0.000 | 0.088 | 0.996 | 1260 | tags=25%, list=18%, signal=30% |
| 99 | IMMUNE\_SYSTEM\_DEVELOPMENT |  | 37 | 0.40 | 1.52 | 0.030 | 0.088 | 0.996 | 1654 | tags=43%, list=23%, signal=56% |
| 100 | REGULATION\_OF\_SIGNAL\_TRANSDUCTION |  | 82 | 0.33 | 1.51 | 0.009 | 0.089 | 0.996 | 1623 | tags=39%, list=23%, signal=50% |
| 101 | CHEMICAL\_HOMEOSTASIS |  | 55 | 0.35 | 1.51 | 0.020 | 0.090 | 0.998 | 1190 | tags=38%, list=17%, signal=46% |
| 102 | ENZYME\_LINKED\_RECEPTOR\_PROTEIN\_SIGNALING\_PATHWAY |  | 45 | 0.38 | 1.51 | 0.017 | 0.091 | 0.998 | 1152 | tags=36%, list=16%, signal=42% |
| 103 | HEMOPOIETIC\_OR\_LYMPHOID\_ORGAN\_DEVELOPMENT |  | 36 | 0.40 | 1.50 | 0.018 | 0.093 | 0.999 | 1654 | tags=44%, list=23%, signal=58% |
| 104 | SECOND\_MESSENGER\_MEDIATED\_SIGNALING |  | 40 | 0.39 | 1.50 | 0.044 | 0.094 | 0.999 | 1398 | tags=38%, list=20%, signal=46% |
| 105 | REGULATION\_OF\_I\_KAPPAB\_KINASE\_NF\_KAPPAB\_CASCADE |  | 38 | 0.39 | 1.50 | 0.043 | 0.094 | 0.999 | 1573 | tags=39%, list=22%, signal=50% |
| 106 | PEPTIDE\_BINDING |  | 24 | 0.43 | 1.49 | 0.039 | 0.096 | 0.999 | 918 | tags=33%, list=13%, signal=38% |
| 107 | INTRINSIC\_TO\_MEMBRANE |  | 388 | 0.26 | 1.49 | 0.006 | 0.097 | 0.999 | 1571 | tags=31%, list=22%, signal=38% |
| 108 | POSITIVE\_REGULATION\_OF\_I\_KAPPAB\_KINASE\_NF\_KAPPAB\_CASCADE |  | 38 | 0.39 | 1.49 | 0.027 | 0.096 | 0.999 | 1573 | tags=39%, list=22%, signal=50% |
| 109 | INTEGRAL\_TO\_MEMBRANE |  | 378 | 0.27 | 1.49 | 0.000 | 0.096 | 0.999 | 1565 | tags=31%, list=22%, signal=38% |
| 110 | POSITIVE\_REGULATION\_OF\_CATALYTIC\_ACTIVITY |  | 64 | 0.34 | 1.49 | 0.008 | 0.096 | 0.999 | 1389 | tags=33%, list=20%, signal=40% |
| 111 | ADENYL\_RIBONUCLEOTIDE\_BINDING |  | 61 | 0.35 | 1.48 | 0.012 | 0.096 | 0.999 | 1044 | tags=30%, list=15%, signal=34% |
| 112 | POSITIVE\_REGULATION\_OF\_CELLULAR\_PROCESS |  | 230 | 0.28 | 1.48 | 0.000 | 0.098 | 0.999 | 1239 | tags=25%, list=17%, signal=29% |
| 113 | PROTEIN\_PROCESSING |  | 17 | 0.46 | 1.47 | 0.073 | 0.101 | 0.999 | 1237 | tags=29%, list=17%, signal=36% |
| 114 | NEGATIVE\_REGULATION\_OF\_BIOLOGICAL\_PROCESS |  | 234 | 0.27 | 1.46 | 0.000 | 0.106 | 1.000 | 1518 | tags=30%, list=21%, signal=37% |
| 115 | ADENYL\_NUCLEOTIDE\_BINDING |  | 62 | 0.34 | 1.46 | 0.023 | 0.106 | 1.000 | 1044 | tags=29%, list=15%, signal=34% |
| 116 | PROTEIN\_DOMAIN\_SPECIFIC\_BINDING |  | 21 | 0.44 | 1.46 | 0.054 | 0.110 | 1.000 | 1260 | tags=38%, list=18%, signal=46% |
| 117 | HOMEOSTATIC\_PROCESS |  | 74 | 0.33 | 1.45 | 0.016 | 0.114 | 1.000 | 1190 | tags=34%, list=17%, signal=40% |
| 118 | TRANSCRIPTION\_COREPRESSOR\_ACTIVITY |  | 39 | 0.37 | 1.44 | 0.022 | 0.118 | 1.000 | 1257 | tags=36%, list=18%, signal=43% |
| 119 | SH3\_SH2\_ADAPTOR\_ACTIVITY |  | 17 | 0.48 | 1.43 | 0.067 | 0.125 | 1.000 | 2278 | tags=65%, list=32%, signal=95% |
| 120 | NEGATIVE\_REGULATION\_OF\_CATALYTIC\_ACTIVITY |  | 20 | 0.45 | 1.41 | 0.074 | 0.146 | 1.000 | 1891 | tags=55%, list=27%, signal=75% |
| 121 | NEGATIVE\_REGULATION\_OF\_CELLULAR\_PROCESS |  | 222 | 0.26 | 1.41 | 0.004 | 0.146 | 1.000 | 1518 | tags=30%, list=21%, signal=37% |
| 122 | SMALL\_GTPASE\_REGULATOR\_ACTIVITY |  | 23 | 0.42 | 1.39 | 0.103 | 0.157 | 1.000 | 2112 | tags=57%, list=30%, signal=80% |
| 123 | POSITIVE\_REGULATION\_OF\_DEVELOPMENTAL\_PROCESS |  | 80 | 0.31 | 1.39 | 0.035 | 0.161 | 1.000 | 1228 | tags=29%, list=17%, signal=34% |
| 124 | PROTEIN\_DIMERIZATION\_ACTIVITY |  | 67 | 0.32 | 1.38 | 0.049 | 0.163 | 1.000 | 1356 | tags=31%, list=19%, signal=38% |
| 125 | ATP\_BINDING |  | 58 | 0.32 | 1.38 | 0.035 | 0.163 | 1.000 | 1044 | tags=28%, list=15%, signal=32% |
| 126 | RESPONSE\_TO\_CHEMICAL\_STIMULUS |  | 105 | 0.29 | 1.37 | 0.030 | 0.170 | 1.000 | 1463 | tags=33%, list=21%, signal=41% |
| 127 | IDENTICAL\_PROTEIN\_BINDING |  | 123 | 0.28 | 1.36 | 0.032 | 0.179 | 1.000 | 1640 | tags=32%, list=23%, signal=41% |
| 128 | PROTEIN\_HOMODIMERIZATION\_ACTIVITY |  | 46 | 0.34 | 1.36 | 0.081 | 0.179 | 1.000 | 1459 | tags=35%, list=21%, signal=44% |
| 129 | CYTOSKELETAL\_PART |  | 60 | 0.32 | 1.35 | 0.067 | 0.188 | 1.000 | 2007 | tags=48%, list=28%, signal=67% |
| 130 | REGULATION\_OF\_BODY\_FLUID\_LEVELS |  | 26 | 0.39 | 1.35 | 0.078 | 0.190 | 1.000 | 1477 | tags=38%, list=21%, signal=48% |
| 131 | PHOSPHOPROTEIN\_PHOSPHATASE\_ACTIVITY |  | 28 | 0.37 | 1.34 | 0.086 | 0.194 | 1.000 | 1491 | tags=43%, list=21%, signal=54% |
| 132 | G\_PROTEIN\_SIGNALING\_COUPLED\_TO\_CAMP\_NUCLEOTIDE\_SECOND\_MESSENGER |  | 15 | 0.44 | 1.34 | 0.140 | 0.193 | 1.000 | 1372 | tags=40%, list=19%, signal=49% |
| 133 | PROTEIN\_AMINO\_ACID\_PHOSPHORYLATION |  | 100 | 0.29 | 1.34 | 0.052 | 0.196 | 1.000 | 1238 | tags=25%, list=17%, signal=30% |
| 134 | MITOTIC\_CELL\_CYCLE |  | 59 | 0.31 | 1.33 | 0.051 | 0.200 | 1.000 | 2229 | tags=49%, list=31%, signal=71% |
| 135 | POSITIVE\_REGULATION\_OF\_TRANSCRIPTION |  | 44 | 0.34 | 1.33 | 0.075 | 0.200 | 1.000 | 974 | tags=25%, list=14%, signal=29% |
| 136 | PEPTIDYL\_AMINO\_ACID\_MODIFICATION |  | 19 | 0.41 | 1.33 | 0.110 | 0.200 | 1.000 | 1794 | tags=47%, list=25%, signal=63% |
| 137 | CELL\_ACTIVATION |  | 24 | 0.40 | 1.33 | 0.101 | 0.199 | 1.000 | 2287 | tags=58%, list=32%, signal=86% |
| 138 | PURINE\_RIBONUCLEOTIDE\_BINDING |  | 78 | 0.30 | 1.33 | 0.036 | 0.198 | 1.000 | 1059 | tags=27%, list=15%, signal=31% |
| 139 | LIPASE\_ACTIVITY |  | 15 | 0.44 | 1.33 | 0.121 | 0.202 | 1.000 | 1474 | tags=47%, list=21%, signal=59% |
| 140 | POSITIVE\_REGULATION\_OF\_CELLULAR\_METABOLIC\_PROCESS |  | 70 | 0.30 | 1.32 | 0.089 | 0.203 | 1.000 | 1230 | tags=29%, list=17%, signal=34% |
| 141 | TRANSCRIPTION\_FACTOR\_ACTIVITY |  | 90 | 0.28 | 1.31 | 0.060 | 0.218 | 1.000 | 1509 | tags=31%, list=21%, signal=39% |
| 142 | POSITIVE\_REGULATION\_OF\_METABOLIC\_PROCESS |  | 73 | 0.30 | 1.30 | 0.064 | 0.222 | 1.000 | 1004 | tags=26%, list=14%, signal=30% |
| 143 | GTPASE\_REGULATOR\_ACTIVITY |  | 46 | 0.33 | 1.30 | 0.104 | 0.222 | 1.000 | 1378 | tags=35%, list=19%, signal=43% |
| 144 | PURINE\_NUCLEOTIDE\_BINDING |  | 79 | 0.29 | 1.30 | 0.097 | 0.222 | 1.000 | 1059 | tags=27%, list=15%, signal=31% |
| 145 | CELL\_CELL\_SIGNALING |  | 95 | 0.27 | 1.29 | 0.049 | 0.238 | 1.000 | 812 | tags=21%, list=11%, signal=23% |
| 146 | PROTEIN\_HETERODIMERIZATION\_ACTIVITY |  | 30 | 0.35 | 1.28 | 0.127 | 0.241 | 1.000 | 572 | tags=23%, list=8%, signal=25% |
| 147 | ENZYME\_REGULATOR\_ACTIVITY |  | 122 | 0.26 | 1.28 | 0.064 | 0.244 | 1.000 | 2142 | tags=45%, list=30%, signal=63% |
| 148 | REGULATION\_OF\_DEVELOPMENTAL\_PROCESS |  | 163 | 0.25 | 1.27 | 0.044 | 0.250 | 1.000 | 983 | tags=21%, list=14%, signal=24% |
| 149 | MEMBRANE\_FRACTION |  | 110 | 0.27 | 1.27 | 0.069 | 0.251 | 1.000 | 1668 | tags=36%, list=24%, signal=47% |
| 150 | LEUKOCYTE\_ACTIVATION |  | 22 | 0.39 | 1.27 | 0.148 | 0.253 | 1.000 | 2287 | tags=59%, list=32%, signal=87% |
| 151 | MYELOID\_CELL\_DIFFERENTIATION |  | 21 | 0.38 | 1.27 | 0.160 | 0.256 | 1.000 | 1954 | tags=52%, list=28%, signal=72% |
| 152 | M\_PHASE\_OF\_MITOTIC\_CELL\_CYCLE |  | 32 | 0.34 | 1.26 | 0.130 | 0.264 | 1.000 | 2308 | tags=50%, list=33%, signal=74% |
| 153 | PHOSPHORIC\_ESTER\_HYDROLASE\_ACTIVITY |  | 53 | 0.30 | 1.26 | 0.129 | 0.265 | 1.000 | 1491 | tags=34%, list=21%, signal=43% |
| 154 | CYTOSKELETON |  | 113 | 0.26 | 1.26 | 0.063 | 0.265 | 1.000 | 1953 | tags=40%, list=28%, signal=54% |
| 155 | MAGNESIUM\_ION\_BINDING |  | 20 | 0.39 | 1.25 | 0.161 | 0.267 | 1.000 | 696 | tags=20%, list=10%, signal=22% |
| 156 | NUCLEOTIDE\_BINDING |  | 87 | 0.27 | 1.25 | 0.108 | 0.265 | 1.000 | 1059 | tags=25%, list=15%, signal=29% |
| 157 | NEGATIVE\_REGULATION\_OF\_GROWTH |  | 15 | 0.42 | 1.25 | 0.189 | 0.268 | 1.000 | 1623 | tags=47%, list=23%, signal=60% |
| 158 | POSITIVE\_REGULATION\_OF\_CELL\_PROLIFERATION |  | 50 | 0.30 | 1.25 | 0.126 | 0.270 | 1.000 | 1230 | tags=26%, list=17%, signal=31% |
| 159 | CARBOHYDRATE\_BINDING |  | 17 | 0.41 | 1.24 | 0.174 | 0.273 | 1.000 | 773 | tags=29%, list=11%, signal=33% |
| 160 | TRANSCRIPTION\_COFACTOR\_ACTIVITY |  | 75 | 0.28 | 1.24 | 0.098 | 0.272 | 1.000 | 1138 | tags=27%, list=16%, signal=31% |
| 161 | SYSTEM\_PROCESS |  | 137 | 0.25 | 1.24 | 0.075 | 0.277 | 1.000 | 1458 | tags=30%, list=21%, signal=37% |
| 162 | RESPONSE\_TO\_ABIOTIC\_STIMULUS |  | 26 | 0.35 | 1.23 | 0.170 | 0.288 | 1.000 | 1746 | tags=54%, list=25%, signal=71% |
| 163 | PROTEIN\_BINDING\_BRIDGING |  | 23 | 0.36 | 1.23 | 0.185 | 0.287 | 1.000 | 2487 | tags=65%, list=35%, signal=100% |
| 164 | KINASE\_REGULATOR\_ACTIVITY |  | 16 | 0.40 | 1.23 | 0.196 | 0.287 | 1.000 | 1824 | tags=44%, list=26%, signal=59% |
| 165 | TRANSCRIPTION\_REPRESSOR\_ACTIVITY |  | 58 | 0.29 | 1.23 | 0.120 | 0.287 | 1.000 | 1077 | tags=26%, list=15%, signal=30% |
| 166 | LYMPHOCYTE\_ACTIVATION |  | 21 | 0.37 | 1.22 | 0.202 | 0.300 | 1.000 | 2287 | tags=57%, list=32%, signal=84% |
| 167 | PHOSPHORYLATION |  | 115 | 0.26 | 1.22 | 0.104 | 0.298 | 1.000 | 1238 | tags=24%, list=17%, signal=29% |
| 168 | POSITIVE\_REGULATION\_OF\_TRANSCRIPTION\_FROM\_RNA\_POLYMERASE\_II\_PROMOTER |  | 21 | 0.38 | 1.22 | 0.192 | 0.296 | 1.000 | 1749 | tags=43%, list=25%, signal=57% |
| 169 | MITOSIS |  | 31 | 0.33 | 1.21 | 0.174 | 0.305 | 1.000 | 2308 | tags=48%, list=33%, signal=71% |
| 170 | T\_CELL\_ACTIVATION |  | 15 | 0.41 | 1.21 | 0.251 | 0.304 | 1.000 | 2287 | tags=60%, list=32%, signal=88% |
| 171 | TRANSCRIPTION\_FACTOR\_BINDING |  | 103 | 0.26 | 1.21 | 0.102 | 0.308 | 1.000 | 1269 | tags=27%, list=18%, signal=33% |
| 172 | TRANSCRIPTION\_ACTIVATOR\_ACTIVITY |  | 51 | 0.30 | 1.21 | 0.162 | 0.308 | 1.000 | 1138 | tags=25%, list=16%, signal=30% |
| 173 | POSITIVE\_REGULATION\_OF\_NUCLEOBASENUCLEOSIDENUCLEOTIDE\_AND\_NUCLEIC\_ACID\_METABOLIC\_PROCESS |  | 48 | 0.29 | 1.20 | 0.142 | 0.315 | 1.000 | 974 | tags=23%, list=14%, signal=26% |
| 174 | REGULATION\_OF\_HYDROLASE\_ACTIVITY |  | 30 | 0.33 | 1.20 | 0.200 | 0.321 | 1.000 | 1893 | tags=43%, list=27%, signal=59% |
| 175 | CELL\_FRACTION |  | 157 | 0.23 | 1.19 | 0.080 | 0.329 | 1.000 | 1190 | tags=25%, list=17%, signal=29% |
| 176 | ENZYME\_INHIBITOR\_ACTIVITY |  | 38 | 0.30 | 1.19 | 0.204 | 0.334 | 1.000 | 1729 | tags=42%, list=24%, signal=55% |
| 177 | NERVOUS\_SYSTEM\_DEVELOPMENT |  | 90 | 0.25 | 1.18 | 0.126 | 0.339 | 1.000 | 1478 | tags=29%, list=21%, signal=36% |
| 178 | STRUCTURAL\_MOLECULE\_ACTIVITY |  | 58 | 0.28 | 1.18 | 0.184 | 0.341 | 1.000 | 1801 | tags=38%, list=25%, signal=50% |
| 179 | I\_KAPPAB\_KINASE\_NF\_KAPPAB\_CASCADE |  | 44 | 0.30 | 1.17 | 0.169 | 0.349 | 1.000 | 1573 | tags=34%, list=22%, signal=44% |
| 180 | TRANSCRIPTION |  | 252 | 0.22 | 1.17 | 0.067 | 0.349 | 1.000 | 1273 | tags=23%, list=18%, signal=27% |
| 181 | OXIDOREDUCTASE\_ACTIVITY\_GO\_0016705 |  | 15 | 0.40 | 1.17 | 0.255 | 0.356 | 1.000 | 1054 | tags=47%, list=15%, signal=55% |
| 182 | LEUKOCYTE\_DIFFERENTIATION |  | 16 | 0.37 | 1.16 | 0.240 | 0.369 | 1.000 | 1626 | tags=44%, list=23%, signal=57% |
| 183 | NEGATIVE\_REGULATION\_OF\_DEVELOPMENTAL\_PROCESS |  | 77 | 0.26 | 1.16 | 0.166 | 0.369 | 1.000 | 1738 | tags=34%, list=25%, signal=44% |
| 184 | RECEPTOR\_SIGNALING\_PROTEIN\_SERINE\_THREONINE\_KINASE\_ACTIVITY |  | 16 | 0.38 | 1.15 | 0.266 | 0.373 | 1.000 | 281 | tags=19%, list=4%, signal=19% |
| 185 | SMALL\_GTPASE\_MEDIATED\_SIGNAL\_TRANSDUCTION |  | 42 | 0.29 | 1.15 | 0.247 | 0.385 | 1.000 | 858 | tags=24%, list=12%, signal=27% |
| 186 | HYDROLASE\_ACTIVITY\_ACTING\_ON\_ESTER\_BONDS |  | 86 | 0.26 | 1.15 | 0.198 | 0.384 | 1.000 | 1538 | tags=33%, list=22%, signal=41% |
| 187 | ENDOSOME |  | 29 | 0.32 | 1.13 | 0.255 | 0.402 | 1.000 | 948 | tags=28%, list=13%, signal=32% |
| 188 | CALCIUM\_ION\_BINDING |  | 33 | 0.31 | 1.13 | 0.264 | 0.405 | 1.000 | 1472 | tags=42%, list=21%, signal=53% |
| 189 | ION\_BINDING |  | 89 | 0.24 | 1.13 | 0.219 | 0.411 | 1.000 | 1472 | tags=28%, list=21%, signal=35% |
| 190 | REGULATION\_OF\_PHOSPHORYLATION |  | 21 | 0.35 | 1.13 | 0.301 | 0.413 | 1.000 | 1824 | tags=48%, list=26%, signal=64% |
| 191 | POST\_TRANSLATIONAL\_PROTEIN\_MODIFICATION |  | 174 | 0.22 | 1.12 | 0.171 | 0.414 | 1.000 | 1532 | tags=28%, list=22%, signal=34% |
| 192 | POSITIVE\_REGULATION\_OF\_HYDROLASE\_ACTIVITY |  | 20 | 0.35 | 1.12 | 0.312 | 0.415 | 1.000 | 1893 | tags=45%, list=27%, signal=61% |
| 193 | REGULATION\_OF\_TRANSCRIPTION |  | 177 | 0.22 | 1.12 | 0.179 | 0.420 | 1.000 | 1273 | tags=24%, list=18%, signal=28% |
| 194 | TISSUE\_DEVELOPMENT |  | 31 | 0.31 | 1.12 | 0.288 | 0.421 | 1.000 | 2103 | tags=52%, list=30%, signal=73% |
| 195 | RESPONSE\_TO\_STRESS |  | 196 | 0.21 | 1.11 | 0.198 | 0.440 | 1.000 | 817 | tags=18%, list=12%, signal=20% |
| 196 | REGULATION\_OF\_APOPTOSIS |  | 125 | 0.22 | 1.11 | 0.232 | 0.440 | 1.000 | 1469 | tags=26%, list=21%, signal=32% |
| 197 | STRUCTURAL\_CONSTITUENT\_OF\_CYTOSKELETON |  | 15 | 0.37 | 1.10 | 0.326 | 0.441 | 1.000 | 1801 | tags=47%, list=25%, signal=62% |
| 198 | REGULATION\_OF\_PROGRAMMED\_CELL\_DEATH |  | 125 | 0.22 | 1.10 | 0.238 | 0.448 | 1.000 | 1469 | tags=26%, list=21%, signal=32% |
| 199 | TRANSCRIPTION\_DNA\_DEPENDENT |  | 216 | 0.21 | 1.10 | 0.214 | 0.454 | 1.000 | 1273 | tags=22%, list=18%, signal=26% |
| 200 | REGULATION\_OF\_GENE\_EXPRESSION |  | 207 | 0.20 | 1.09 | 0.224 | 0.469 | 1.000 | 1004 | tags=19%, list=14%, signal=21% |
| 201 | RNA\_BIOSYNTHETIC\_PROCESS |  | 217 | 0.20 | 1.08 | 0.230 | 0.475 | 1.000 | 1273 | tags=22%, list=18%, signal=26% |
| 202 | TRANSMEMBRANE\_RECEPTOR\_PROTEIN\_SERINE\_THREONINE\_KINASE\_SIGNALING\_PATHWAY |  | 17 | 0.36 | 1.08 | 0.391 | 0.473 | 1.000 | 1048 | tags=41%, list=15%, signal=48% |
| 203 | CAMP\_MEDIATED\_SIGNALING |  | 16 | 0.36 | 1.08 | 0.329 | 0.477 | 1.000 | 1372 | tags=38%, list=19%, signal=46% |
| 204 | SOLUBLE\_FRACTION |  | 57 | 0.25 | 1.08 | 0.311 | 0.478 | 1.000 | 940 | tags=21%, list=13%, signal=24% |
| 205 | PHOSPHORIC\_MONOESTER\_HYDROLASE\_ACTIVITY |  | 41 | 0.28 | 1.08 | 0.322 | 0.476 | 1.000 | 1491 | tags=37%, list=21%, signal=46% |
| 206 | BIOPOLYMER\_MODIFICATION |  | 236 | 0.20 | 1.07 | 0.215 | 0.486 | 1.000 | 1827 | tags=31%, list=26%, signal=41% |
| 207 | REGULATION\_OF\_NUCLEOBASENUCLEOSIDENUCLEOTIDE\_AND\_NUCLEIC\_ACID\_METABOLIC\_PROCESS |  | 196 | 0.21 | 1.07 | 0.275 | 0.489 | 1.000 | 1285 | tags=23%, list=18%, signal=27% |
| 208 | GROWTH\_FACTOR\_ACTIVITY |  | 19 | 0.33 | 1.07 | 0.372 | 0.493 | 1.000 | 2205 | tags=53%, list=31%, signal=76% |
| 209 | PROTEIN\_SERINE\_THREONINE\_KINASE\_ACTIVITY |  | 82 | 0.23 | 1.07 | 0.341 | 0.496 | 1.000 | 1239 | tags=22%, list=17%, signal=26% |
| 210 | APOPTOSIS\_GO |  | 157 | 0.21 | 1.06 | 0.305 | 0.511 | 1.000 | 1469 | tags=25%, list=21%, signal=31% |
| 211 | MUSCLE\_DEVELOPMENT |  | 27 | 0.30 | 1.05 | 0.400 | 0.520 | 1.000 | 2007 | tags=48%, list=28%, signal=67% |
| 212 | PROTEIN\_MODIFICATION\_PROCESS |  | 229 | 0.20 | 1.05 | 0.289 | 0.518 | 1.000 | 1827 | tags=31%, list=26%, signal=41% |
| 213 | TRANSCRIPTION\_FROM\_RNA\_POLYMERASE\_II\_PROMOTER |  | 155 | 0.21 | 1.05 | 0.281 | 0.516 | 1.000 | 1509 | tags=25%, list=21%, signal=31% |
| 214 | CYTOPLASMIC\_MEMBRANE\_BOUND\_VESICLE |  | 43 | 0.26 | 1.05 | 0.341 | 0.514 | 1.000 | 847 | tags=19%, list=12%, signal=21% |
| 215 | PROGRAMMED\_CELL\_DEATH |  | 157 | 0.21 | 1.05 | 0.303 | 0.516 | 1.000 | 1469 | tags=25%, list=21%, signal=31% |
| 216 | POSITIVE\_REGULATION\_OF\_RNA\_METABOLIC\_PROCESS |  | 38 | 0.27 | 1.05 | 0.327 | 0.514 | 1.000 | 1824 | tags=34%, list=26%, signal=46% |
| 217 | POSITIVE\_REGULATION\_OF\_TRANSCRIPTIONDNA\_DEPENDENT |  | 38 | 0.27 | 1.05 | 0.367 | 0.518 | 1.000 | 1824 | tags=34%, list=26%, signal=46% |
| 218 | CYTOPLASMIC\_VESICLE |  | 43 | 0.26 | 1.04 | 0.408 | 0.541 | 1.000 | 847 | tags=19%, list=12%, signal=21% |
| 219 | REGULATION\_OF\_MITOSIS |  | 18 | 0.33 | 1.04 | 0.406 | 0.540 | 1.000 | 1827 | tags=39%, list=26%, signal=52% |
| 220 | G\_PROTEIN\_SIGNALING\_COUPLED\_TO\_CYCLIC\_NUCLEOTIDE\_SECOND\_MESSENGER |  | 23 | 0.30 | 1.03 | 0.413 | 0.547 | 1.000 | 1372 | tags=30%, list=19%, signal=38% |
| 221 | CELL\_JUNCTION |  | 27 | 0.29 | 1.03 | 0.421 | 0.549 | 1.000 | 2268 | tags=41%, list=32%, signal=60% |
| 222 | GTPASE\_ACTIVATOR\_ACTIVITY |  | 19 | 0.32 | 1.03 | 0.392 | 0.549 | 1.000 | 1893 | tags=47%, list=27%, signal=64% |
| 223 | SKELETAL\_DEVELOPMENT |  | 28 | 0.29 | 1.02 | 0.403 | 0.557 | 1.000 | 1908 | tags=39%, list=27%, signal=54% |
| 224 | GUANYL\_NUCLEOTIDE\_EXCHANGE\_FACTOR\_ACTIVITY |  | 18 | 0.33 | 1.02 | 0.410 | 0.558 | 1.000 | 1308 | tags=33%, list=18%, signal=41% |
| 225 | NEURON\_DEVELOPMENT |  | 15 | 0.34 | 1.02 | 0.423 | 0.558 | 1.000 | 572 | tags=27%, list=8%, signal=29% |
| 226 | CELL\_DEVELOPMENT |  | 200 | 0.19 | 1.01 | 0.404 | 0.577 | 1.000 | 1500 | tags=25%, list=21%, signal=31% |
| 227 | MACROMOLECULAR\_COMPLEX\_ASSEMBLY |  | 104 | 0.21 | 1.01 | 0.431 | 0.580 | 1.000 | 326 | tags=10%, list=5%, signal=10% |
| 228 | REGULATION\_OF\_CELLULAR\_METABOLIC\_PROCESS |  | 251 | 0.19 | 1.01 | 0.420 | 0.577 | 1.000 | 1308 | tags=23%, list=18%, signal=27% |
| 229 | REGULATION\_OF\_METABOLIC\_PROCESS |  | 256 | 0.19 | 1.01 | 0.424 | 0.576 | 1.000 | 1308 | tags=23%, list=18%, signal=27% |
| 230 | ANTI\_APOPTOSIS |  | 47 | 0.24 | 0.99 | 0.430 | 0.618 | 1.000 | 875 | tags=19%, list=12%, signal=22% |
| 231 | REGULATION\_OF\_CELL\_CYCLE |  | 78 | 0.22 | 0.99 | 0.455 | 0.628 | 1.000 | 1188 | tags=24%, list=17%, signal=29% |
| 232 | PROTEIN\_AMINO\_ACID\_DEPHOSPHORYLATION |  | 21 | 0.30 | 0.99 | 0.467 | 0.628 | 1.000 | 1367 | tags=38%, list=19%, signal=47% |
| 233 | GATED\_CHANNEL\_ACTIVITY |  | 21 | 0.30 | 0.99 | 0.484 | 0.625 | 1.000 | 960 | tags=29%, list=14%, signal=33% |
| 234 | GENERATION\_OF\_NEURONS |  | 20 | 0.31 | 0.99 | 0.479 | 0.623 | 1.000 | 572 | tags=25%, list=8%, signal=27% |
| 235 | REGULATION\_OF\_TRANSCRIPTIONDNA\_DEPENDENT |  | 145 | 0.19 | 0.98 | 0.522 | 0.627 | 1.000 | 1273 | tags=21%, list=18%, signal=26% |
| 236 | CATION\_BINDING |  | 69 | 0.22 | 0.98 | 0.473 | 0.634 | 1.000 | 1472 | tags=29%, list=21%, signal=36% |
| 237 | REGULATION\_OF\_CELLULAR\_COMPONENT\_ORGANIZATION\_AND\_BIOGENESIS |  | 43 | 0.24 | 0.97 | 0.484 | 0.658 | 1.000 | 885 | tags=21%, list=12%, signal=24% |
| 238 | ENZYME\_ACTIVATOR\_ACTIVITY |  | 47 | 0.24 | 0.97 | 0.487 | 0.656 | 1.000 | 1515 | tags=32%, list=21%, signal=40% |
| 239 | PHOSPHOLIPID\_METABOLIC\_PROCESS |  | 23 | 0.29 | 0.96 | 0.509 | 0.668 | 1.000 | 1474 | tags=43%, list=21%, signal=55% |
| 240 | CELLULAR\_COMPONENT\_ASSEMBLY |  | 109 | 0.20 | 0.95 | 0.529 | 0.685 | 1.000 | 493 | tags=11%, list=7%, signal=12% |
| 241 | REGULATION\_OF\_RNA\_METABOLIC\_PROCESS |  | 148 | 0.19 | 0.94 | 0.648 | 0.710 | 1.000 | 1273 | tags=21%, list=18%, signal=25% |
| 242 | METAL\_ION\_TRANSPORT |  | 23 | 0.29 | 0.94 | 0.524 | 0.719 | 1.000 | 960 | tags=26%, list=14%, signal=30% |
| 243 | RNA\_POLYMERASE\_II\_TRANSCRIPTION\_FACTOR\_ACTIVITY |  | 54 | 0.23 | 0.93 | 0.607 | 0.727 | 1.000 | 1359 | tags=24%, list=19%, signal=30% |
| 244 | TRANSCRIPTION\_FACTOR\_COMPLEX |  | 23 | 0.28 | 0.93 | 0.583 | 0.731 | 1.000 | 966 | tags=22%, list=14%, signal=25% |
| 245 | TRANSMEMBRANE\_RECEPTOR\_PROTEIN\_KINASE\_ACTIVITY |  | 19 | 0.29 | 0.93 | 0.559 | 0.732 | 1.000 | 222 | tags=11%, list=3%, signal=11% |
| 246 | CATION\_TRANSPORT |  | 36 | 0.24 | 0.93 | 0.591 | 0.732 | 1.000 | 960 | tags=22%, list=14%, signal=26% |
| 247 | CARBOHYDRATE\_BIOSYNTHETIC\_PROCESS |  | 15 | 0.31 | 0.93 | 0.551 | 0.733 | 1.000 | 730 | tags=27%, list=10%, signal=30% |
| 248 | PROTEIN\_KINASE\_BINDING |  | 19 | 0.29 | 0.93 | 0.521 | 0.733 | 1.000 | 923 | tags=37%, list=13%, signal=42% |
| 249 | MICROTUBULE\_CYTOSKELETON |  | 45 | 0.23 | 0.92 | 0.602 | 0.731 | 1.000 | 1196 | tags=27%, list=17%, signal=32% |
| 250 | CELL\_CYCLE\_PHASE |  | 60 | 0.22 | 0.92 | 0.611 | 0.730 | 1.000 | 2229 | tags=45%, list=31%, signal=65% |
| 251 | CELL\_CYCLE\_PROCESS |  | 67 | 0.21 | 0.92 | 0.614 | 0.732 | 1.000 | 2229 | tags=43%, list=31%, signal=63% |
| 252 | MEMBRANE\_BOUND\_VESICLE |  | 44 | 0.23 | 0.92 | 0.565 | 0.731 | 1.000 | 847 | tags=18%, list=12%, signal=21% |
| 253 | M\_PHASE |  | 35 | 0.25 | 0.92 | 0.614 | 0.731 | 1.000 | 1897 | tags=37%, list=27%, signal=50% |
| 254 | INTERPHASE\_OF\_MITOTIC\_CELL\_CYCLE |  | 27 | 0.26 | 0.92 | 0.581 | 0.728 | 1.000 | 2229 | tags=48%, list=31%, signal=70% |
| 255 | VESICLE |  | 45 | 0.23 | 0.91 | 0.599 | 0.739 | 1.000 | 847 | tags=18%, list=12%, signal=20% |
| 256 | MICROTUBULE\_ORGANIZING\_CENTER |  | 19 | 0.28 | 0.91 | 0.577 | 0.739 | 1.000 | 1014 | tags=26%, list=14%, signal=31% |
| 257 | NUCLEUS |  | 485 | 0.16 | 0.91 | 0.864 | 0.737 | 1.000 | 1359 | tags=21%, list=19%, signal=24% |
| 258 | REGULATION\_OF\_CYCLIN\_DEPENDENT\_PROTEIN\_KINASE\_ACTIVITY |  | 18 | 0.29 | 0.91 | 0.592 | 0.738 | 1.000 | 967 | tags=22%, list=14%, signal=26% |
| 259 | ION\_TRANSPORT |  | 46 | 0.23 | 0.90 | 0.621 | 0.757 | 1.000 | 1622 | tags=33%, list=23%, signal=42% |
| 260 | METAL\_ION\_TRANSMEMBRANE\_TRANSPORTER\_ACTIVITY |  | 30 | 0.25 | 0.90 | 0.588 | 0.762 | 1.000 | 1188 | tags=30%, list=17%, signal=36% |
| 261 | NEGATIVE\_REGULATION\_OF\_TRANSCRIPTION |  | 60 | 0.21 | 0.90 | 0.696 | 0.761 | 1.000 | 607 | tags=17%, list=9%, signal=18% |
| 262 | ACTIVE\_TRANSMEMBRANE\_TRANSPORTER\_ACTIVITY |  | 39 | 0.23 | 0.89 | 0.632 | 0.772 | 1.000 | 1341 | tags=28%, list=19%, signal=35% |
| 263 | EPIDERMIS\_DEVELOPMENT |  | 16 | 0.29 | 0.88 | 0.619 | 0.785 | 1.000 | 1436 | tags=31%, list=20%, signal=39% |
| 264 | REGULATION\_OF\_RESPONSE\_TO\_STIMULUS |  | 19 | 0.28 | 0.88 | 0.630 | 0.789 | 1.000 | 1908 | tags=37%, list=27%, signal=50% |
| 265 | NEUROGENESIS |  | 24 | 0.26 | 0.88 | 0.634 | 0.795 | 1.000 | 572 | tags=21%, list=8%, signal=23% |
| 266 | POSITIVE\_REGULATION\_OF\_CELLULAR\_PROTEIN\_METABOLIC\_PROCESS |  | 23 | 0.26 | 0.88 | 0.643 | 0.793 | 1.000 | 1230 | tags=35%, list=17%, signal=42% |
| 267 | CATION\_CHANNEL\_ACTIVITY |  | 22 | 0.26 | 0.87 | 0.644 | 0.800 | 1.000 | 1188 | tags=32%, list=17%, signal=38% |
| 268 | STEROID\_METABOLIC\_PROCESS |  | 20 | 0.27 | 0.87 | 0.639 | 0.802 | 1.000 | 988 | tags=20%, list=14%, signal=23% |
| 269 | PROTEIN\_KINASE\_ACTIVITY |  | 117 | 0.18 | 0.87 | 0.777 | 0.802 | 1.000 | 1746 | tags=27%, list=25%, signal=36% |
| 270 | CYCLIC\_NUCLEOTIDE\_MEDIATED\_SIGNALING |  | 24 | 0.26 | 0.87 | 0.680 | 0.800 | 1.000 | 1372 | tags=29%, list=19%, signal=36% |
| 271 | VESICLE\_MEDIATED\_TRANSPORT |  | 78 | 0.19 | 0.86 | 0.731 | 0.802 | 1.000 | 1931 | tags=33%, list=27%, signal=45% |
| 272 | HYDROLASE\_ACTIVITY\_ACTING\_ON\_GLYCOSYL\_BONDS |  | 20 | 0.27 | 0.86 | 0.657 | 0.802 | 1.000 | 1165 | tags=25%, list=16%, signal=30% |
| 273 | REGULATION\_OF\_IMMUNE\_SYSTEM\_PROCESS |  | 19 | 0.27 | 0.86 | 0.652 | 0.802 | 1.000 | 1004 | tags=26%, list=14%, signal=31% |
| 274 | CELL\_CYCLE\_GO\_0007049 |  | 114 | 0.18 | 0.85 | 0.790 | 0.818 | 1.000 | 1235 | tags=20%, list=17%, signal=24% |
| 275 | MEMBRANE\_LIPID\_METABOLIC\_PROCESS |  | 33 | 0.23 | 0.85 | 0.705 | 0.821 | 1.000 | 1474 | tags=36%, list=21%, signal=46% |
| 276 | PROTEIN\_C\_TERMINUS\_BINDING |  | 29 | 0.23 | 0.84 | 0.701 | 0.826 | 1.000 | 370 | tags=14%, list=5%, signal=14% |
| 277 | NEGATIVE\_REGULATION\_OF\_APOPTOSIS |  | 55 | 0.20 | 0.84 | 0.764 | 0.827 | 1.000 | 1469 | tags=25%, list=21%, signal=32% |
| 278 | REGULATION\_OF\_TRANSCRIPTION\_FROM\_RNA\_POLYMERASE\_II\_PROMOTER |  | 89 | 0.18 | 0.84 | 0.827 | 0.826 | 1.000 | 1359 | tags=21%, list=19%, signal=26% |
| 279 | NEGATIVE\_REGULATION\_OF\_PROGRAMMED\_CELL\_DEATH |  | 55 | 0.20 | 0.84 | 0.789 | 0.827 | 1.000 | 1469 | tags=25%, list=21%, signal=32% |
| 280 | NEGATIVE\_REGULATION\_OF\_CELLULAR\_METABOLIC\_PROCESS |  | 80 | 0.18 | 0.84 | 0.824 | 0.824 | 1.000 | 2138 | tags=38%, list=30%, signal=53% |
| 281 | PHOSPHOTRANSFERASE\_ACTIVITY\_ALCOHOL\_GROUP\_AS\_ACCEPTOR |  | 140 | 0.17 | 0.84 | 0.870 | 0.826 | 1.000 | 1239 | tags=20%, list=17%, signal=24% |
| 282 | REGULATION\_OF\_PROTEIN\_METABOLIC\_PROCESS |  | 57 | 0.19 | 0.84 | 0.805 | 0.823 | 1.000 | 1824 | tags=35%, list=26%, signal=47% |
| 283 | NEURON\_DIFFERENTIATION |  | 19 | 0.26 | 0.83 | 0.695 | 0.829 | 1.000 | 572 | tags=21%, list=8%, signal=23% |
| 284 | NEGATIVE\_REGULATION\_OF\_NUCLEOBASENUCLEOSIDENUCLEOTIDE\_AND\_NUCLEIC\_ACID\_METABOLIC\_PROCESS |  | 67 | 0.19 | 0.82 | 0.851 | 0.843 | 1.000 | 607 | tags=15%, list=9%, signal=16% |
| 285 | TRANSCRIPTION\_COACTIVATOR\_ACTIVITY |  | 34 | 0.22 | 0.82 | 0.791 | 0.844 | 1.000 | 1138 | tags=21%, list=16%, signal=24% |
| 286 | UBIQUITIN\_CYCLE |  | 23 | 0.24 | 0.81 | 0.744 | 0.855 | 1.000 | 1911 | tags=48%, list=27%, signal=65% |
| 287 | CENTRAL\_NERVOUS\_SYSTEM\_DEVELOPMENT |  | 30 | 0.22 | 0.80 | 0.779 | 0.874 | 1.000 | 1308 | tags=27%, list=18%, signal=33% |
| 288 | REPRODUCTIVE\_PROCESS |  | 42 | 0.20 | 0.79 | 0.810 | 0.880 | 1.000 | 1696 | tags=33%, list=24%, signal=44% |
| 289 | GTPASE\_BINDING |  | 15 | 0.27 | 0.79 | 0.741 | 0.879 | 1.000 | 681 | tags=20%, list=10%, signal=22% |
| 290 | RNA\_METABOLIC\_PROCESS |  | 290 | 0.14 | 0.78 | 0.995 | 0.883 | 1.000 | 1273 | tags=18%, list=18%, signal=21% |
| 291 | SMALL\_GTPASE\_BINDING |  | 15 | 0.27 | 0.78 | 0.770 | 0.888 | 1.000 | 681 | tags=20%, list=10%, signal=22% |
| 292 | NEGATIVE\_REGULATION\_OF\_METABOLIC\_PROCESS |  | 82 | 0.17 | 0.78 | 0.919 | 0.886 | 1.000 | 607 | tags=13%, list=9%, signal=15% |
| 293 | LIPID\_CATABOLIC\_PROCESS |  | 15 | 0.26 | 0.78 | 0.773 | 0.885 | 1.000 | 1361 | tags=40%, list=19%, signal=49% |
| 294 | DNA\_BINDING |  | 175 | 0.15 | 0.76 | 0.979 | 0.901 | 1.000 | 1509 | tags=22%, list=21%, signal=28% |
| 295 | PRIMARY\_ACTIVE\_TRANSMEMBRANE\_TRANSPORTER\_ACTIVITY |  | 15 | 0.25 | 0.75 | 0.831 | 0.909 | 1.000 | 1190 | tags=27%, list=17%, signal=32% |
| 296 | HYDROLASE\_ACTIVITY\_ACTING\_ON\_ACID\_ANHYDRIDESCATALYZING\_TRANSMEMBRANE\_MOVEMENT\_OF\_SUBSTANCES |  | 15 | 0.25 | 0.74 | 0.820 | 0.917 | 1.000 | 1190 | tags=27%, list=17%, signal=32% |
| 297 | ECTODERM\_DEVELOPMENT |  | 17 | 0.23 | 0.72 | 0.836 | 0.932 | 1.000 | 1436 | tags=29%, list=20%, signal=37% |
| 298 | G\_PROTEIN\_COUPLED\_RECEPTOR\_ACTIVITY |  | 28 | 0.20 | 0.72 | 0.899 | 0.935 | 1.000 | 1333 | tags=21%, list=19%, signal=26% |
| 299 | PROTEIN\_UBIQUITINATION |  | 19 | 0.23 | 0.72 | 0.871 | 0.932 | 1.000 | 1911 | tags=47%, list=27%, signal=65% |
| 300 | NUCLEAR\_MEMBRANE |  | 24 | 0.21 | 0.71 | 0.891 | 0.932 | 1.000 | 1197 | tags=25%, list=17%, signal=30% |
| 301 | ATPASE\_ACTIVITY\_COUPLED\_TO\_MOVEMENT\_OF\_SUBSTANCES |  | 16 | 0.23 | 0.71 | 0.862 | 0.935 | 1.000 | 1190 | tags=25%, list=17%, signal=30% |
| 302 | PROTEIN\_MODIFICATION\_BY\_SMALL\_PROTEIN\_CONJUGATION |  | 20 | 0.21 | 0.69 | 0.897 | 0.946 | 1.000 | 1911 | tags=45%, list=27%, signal=61% |
| 303 | INTERCELLULAR\_JUNCTION |  | 20 | 0.20 | 0.67 | 0.911 | 0.955 | 1.000 | 2453 | tags=45%, list=35%, signal=69% |
| 304 | SERINE\_TYPE\_PEPTIDASE\_ACTIVITY |  | 15 | 0.20 | 0.60 | 0.950 | 0.985 | 1.000 | 1678 | tags=40%, list=24%, signal=52% |
| 305 | SERINE\_HYDROLASE\_ACTIVITY |  | 15 | 0.20 | 0.59 | 0.951 | 0.985 | 1.000 | 1678 | tags=40%, list=24%, signal=52% |
| 306 | PROTEIN\_TYROSINE\_KINASE\_ACTIVITY |  | 25 | 0.15 | 0.51 | 0.995 | 0.995 | 1.000 | 1696 | tags=20%, list=24%, signal=26% |
Table: Gene sets enriched in phenotype **na **[plain text format]****
