# Supplementary material for: Network-based integration of molecular and physiological data elucidates regulatory mechanisms underlying adaptation to high-fat diet
Source: Genes Nutr. 2015 May 28;10(4):22. doi: 10.1007/s12263-015-0470-6 (PMC4446272; doi:10.1007/s12263-015-0470-6)
Supplement: Supplementary file 4 — Supplementary material 4 (ZIP 6984 kb) [file 12263_2015_470_MOESM4_ESM.zip › HF LF 5 d GSEA result/index.html]

Index for xtools.gsea.GseaPreranked my\_analysis.GseaPreranked.1365164922288

### GSEA Report for Dataset comp\_HF5d-LF5d

#### Enrichment in phenotype: **na**

- 306 / 527 gene sets are upregulated in phenotype **na\_pos**- 147 gene sets are significant at FDR < 25%- 74 gene sets are significantly enriched at nominal pvalue < 1%- 125 gene sets are significantly enriched at nominal pvalue < 5%- Snapshot of enrichment results- Detailed enrichment results in html format- Detailed enrichment results in excel format (tab delimited text)- Guide to interpret results

#### Enrichment in phenotype: **na**

- 221 / 527 gene sets are upregulated in phenotype **na\_neg**- 48 gene sets are significantly enriched at FDR < 25%- 28 gene sets are significantly enriched at nominal pvalue < 1%- 47 gene sets are significantly enriched at nominal pvalue < 5%- Snapshot of enrichment results- Detailed enrichment results in html format- Detailed enrichment results in excel format (tab delimited text)- Guide to interpret results

#### Dataset details

- The dataset has 22690 native features- After collapsing features into gene symbols, there are: 7092 genes

#### Gene set details

- Gene set size filters (min=15, max=500) resulted in filtering out 927 / 1454 gene sets- The remaining 527 gene sets were used in the analysis- List of gene sets used and their sizes (restricted to features in the specified dataset)

#### Gene markers for the **na\_pos** *versus* **na\_neg** comparison

- The dataset has 7092 features (genes)- Detailed rank ordered gene list for all features in the dataset

#### Global statistics and plots

- Plot of p-values *vs.* NES- Global ES histogram

#### Other

- Parameters used for this analysis

#### Comments

- There were duplicate row identifiers in the specified ranked list. One id was arbitarilly choosen. Details are below.
  Generally, this is OK, but if you want to avoid this automagic, edit your ranked list so that all row ids are unique
  # of row ids in original dataset: 25150
  # of row UNIQUE ids in original dataset: 22690
  # The duplicates were
    

  ```
  A_51_P124535
  A_51_P124535
  A_51_P124535
  A_51_P124535
  A_51_P124535
  A_51_P124535
  A_51_P124535
  A_51_P124535
  A_51_P124535
  A_51_P124748
  A_51_P485421
  A_51_P485421
  A_51_P124748
  A_51_P108226
  A_51_P485421
  A_51_P485421
  A_51_P112734
  A_51_P124748
  A_51_P485421
  A_51_P112734
  A_51_P124748
  A_51_P112734
  A_51_P485421
  A_51_P485421
  A_51_P118132
  A_51_P485421
  A_51_P108226
  A_51_P108226
  A_51_P124748
  A_51_P112734
  A_51_P108226
  A_51_P108226
  A_51_P124748
  A_51_P108226
  A_51_P427674
  A_51_P485421
  A_51_P108226
  A_51_P126437
  A_51_P126437
  A_51_P108226
  A_51_P126437
  A_51_P126437
  A_51_P118132
  A_51_P112445
  A_51_P118132
  A_51_P112734
  A_51_P427674
  A_51_P124748
  A_51_P427674
  A_51_P108226
  A_52_P374882
  A_51_P124748
  A_51_P112734
  A_51_P126437
  A_51_P126437
  A_51_P126437
  A_51_P118132
  A_52_P374882
  A_51_P118132
  A_51_P126437
  A_51_P103650
  A_51_P103650
  A_51_P118132
  A_52_P374882
  A_52_P374882
  A_51_P121031
  A_51_P171999
  A_51_P103650
  A_51_P171999
  A_52_P374882
  A_51_P121031
  A_52_P374882
  A_51_P171999
  A_51_P121031
  A_52_P374882
  A_52_P262219
  A_51_P171999
  A_52_P262219
  A_52_P262219
  A_51_P171999
  A_51_P112734
  A_51_P103650
  A_52_P106259
  A_51_P112445
  A_52_P374882
  A_52_P262219
  A_51_P171999
  A_51_P126437
  A_51_P118132
  A_51_P171999
  A_51_P121031
  A_51_P119429
  A_51_P427674
  A_52_P262219
  A_52_P106259
  A_51_P103650
  A_52_P374882
  A_51_P105124
  A_51_P118132
  A_52_P262219
  A_52_P106259
  A_51_P119429
  A_52_P262219
  A_51_P171999
  A_51_P171999
  A_51_P130095
  A_51_P119429
  A_52_P106259
  A_51_P130095
  A_51_P105124
  A_51_P105124
  A_51_P103650
  A_51_P103650
  A_51_P118132
  A_51_P108459
  A_51_P112734
  A_51_P108459
  A_52_P106259
  A_51_P130095
  A_51_P121031
  A_52_P106259
  A_52_P262219
  A_51_P130095
  A_52_P106259
  A_51_P112734
  A_51_P121031
  A_51_P119429
  A_51_P105124
  A_52_P106259
  A_51_P427674
  A_51_P119429
  A_51_P108459
  A_51_P130095
  A_51_P105124
  A_51_P119429
  A_51_P124748
  A_51_P427674
  A_51_P108459
  A_51_P195506
  A_51_P195506
  A_52_P192426
  A_51_P116651
  A_51_P119429
  A_51_P121031
  A_51_P195506
  A_51_P119429
  A_51_P103650
  A_51_P119429
  A_52_P262219
  A_51_P121031
  A_51_P130095
  A_51_P130095
  A_51_P127681
  A_51_P121031
  A_52_P106259
  A_51_P115085
  A_51_P121891
  A_51_P115178
  A_51_P115085
  A_52_P192426
  A_51_P102789
  A_51_P112445
  A_51_P115085
  A_51_P102789
  A_51_P105124
  A_51_P127681
  A_51_P118603
  A_51_P122740
  A_51_P115085
  A_51_P108459
  A_51_P115085
  A_51_P122740
  A_51_P115178
  A_51_P103650
  A_51_P121891
  A_51_P118603
  A_51_P195506
  A_51_P121891
  A_51_P114878
  A_51_P105124
  A_51_P127681
  A_51_P452629
  A_51_P102789
  A_51_P102789
  A_51_P116651
  A_51_P114616
  A_51_P122582
  A_51_P112762
  A_51_P112445
  A_51_P427674
  A_51_P115085
  A_51_P114616
  A_51_P121891
  A_51_P118603
  A_51_P114878
  A_51_P115085
  A_51_P130095
  A_51_P195506
  A_51_P105124
  A_51_P108459
  A_51_P102789
  A_51_P130095
  A_52_P192426
  A_51_P286737
  A_51_P106144
  A_51_P102789
  A_51_P108459
  A_51_P116651
  A_51_P286737
  A_51_P105124
  A_52_P192426
  A_51_P286737
  A_51_P286737
  A_51_P125135
  A_51_P108252
  A_51_P106144
  A_51_P427674
  A_51_P108252
  A_51_P112445
  A_51_P452629
  A_51_P286737
  A_51_P121891
  A_51_P121891
  A_51_P114616
  A_51_P112762
  A_51_P112762
  A_51_P121891
  A_51_P452629
  A_51_P102789
  A_51_P116651
  A_51_P201480
  A_51_P452629
  A_52_P192426
  A_51_P201480
  A_51_P106144
  A_51_P114616
  A_51_P125135
  A_51_P128463
  A_51_P104125
  A_51_P195506
  A_51_P172231
  A_51_P123705
  A_51_P127681
  A_51_P207591
  A_51_P195506
  A_51_P112762
  A_51_P108252
  A_51_P195506
  A_51_P115085
  A_51_P115178
  A_51_P125135
  A_51_P107362
  A_51_P207591
  A_51_P128775
  A_51_P452629
  A_51_P112445
  A_51_P112762
  A_51_P195506
  A_51_P286737
  A_51_P452629
  A_51_P116651
  A_51_P128463
  A_51_P108252
  A_51_P116651
  A_51_P127681
  A_51_P128336
  A_51_P102789
  A_51_P118603
  A_51_P201480
  A_51_P207591
  A_51_P286737
  A_51_P115178
  A_51_P452629
  A_51_P116651
  A_51_P127681
  A_51_P207591
  A_52_P192426
  A_51_P505868
  A_51_P505868
  A_51_P172231
  A_51_P115178
  A_51_P128775
  A_51_P104125
  A_51_P172231
  A_51_P106144
  A_51_P112762
  A_51_P114878
  A_51_P126835
  A_51_P128463
  A_51_P121390
  A_51_P452629
  A_51_P201480
  A_51_P345649
  A_51_P286737
  A_51_P116651
  A_51_P207591
  A_51_P127681
  A_51_P128775
  A_51_P122740
  A_51_P108252
  A_51_P114878
  A_51_P122740
  A_51_P201480
  A_51_P127681
  A_51_P100787
  A_51_P172231
  A_51_P104125
  A_51_P116651
  A_51_P118763
  A_51_P128463
  A_52_P192426
  A_51_P104125
  A_51_P121891
  A_51_P128463
  A_51_P128775
  A_51_P172231
  A_51_P106144
  A_51_P207591
  A_51_P346445
  A_51_P128463
  A_51_P104897
  A_51_P112762
  A_51_P114616
  A_51_P117226
  A_51_P104125
  A_51_P108459
  A_51_P106144
  A_51_P201480
  A_51_P104897
  A_51_P104897
  A_51_P121390
  A_51_P118603
  A_51_P505868
  A_51_P123705
  A_51_P104897
  A_51_P108252
  A_51_P345649
  A_51_P122582
  A_51_P108252
  A_51_P121390
  A_51_P201480
  A_52_P192426
  A_51_P112445
  A_51_P111612
  A_51_P427674
  A_51_P111612
  A_51_P505868
  A_51_P127681
  A_51_P207591
  A_51_P108252
  A_51_P125135
  A_51_P100787
  A_51_P122582
  A_51_P118763
  A_51_P125691
  A_51_P172231
  A_51_P286737
  A_51_P399845
  A_51_P452629
  A_51_P114616
  A_51_P125135
  A_51_P121390
  A_51_P122582
  A_51_P108020
  A_51_P112762
  A_51_P505868
  A_51_P121891
  A_51_P114062
  A_51_P115178
  A_51_P113722
  A_51_P201480
  A_51_P106144
  A_51_P104897
  A_51_P108020
  A_51_P108020
  A_51_P505868
  A_51_P115178
  A_52_P192426
  A_51_P106144
  A_51_P114616
  A_51_P122582
  A_51_P125135
  A_51_P345649
  A_51_P112445
  A_51_P207591
  A_51_P207591
  A_51_P411297
  A_51_P111612
  A_51_P113722
  A_51_P115085
  A_51_P107362
  A_51_P104897
  A_51_P106952
  A_51_P110395
  A_51_P113722
  A_51_P104608
  A_51_P108252
  A_51_P505868
  A_51_P113003
  A_51_P122740
  A_51_P110759
  A_51_P117226
  A_51_P279552
  A_51_P104897
  A_51_P505868
  A_51_P111757
  A_51_P254855
  A_51_P104897
  A_51_P104897
  A_51_P104125
  A_51_P104608
  A_51_P128463
  A_51_P105927
  A_51_P254855
  A_51_P122582
  A_51_P108020
  A_51_P345649
  A_51_P109144
  A_51_P279552
  A_51_P345649
  A_51_P279552
  A_51_P254855
  A_51_P104125
  A_51_P115178
  A_51_P128463
  A_51_P254855
  A_51_P279552
  A_51_P254855
  A_51_P108020
  A_51_P172231
  A_51_P114616
  A_51_P279552
  A_52_P420504
  A_51_P346445
  A_51_P346445
  A_51_P102355
  A_52_P420504
  A_51_P452153
  A_52_P420504
  A_51_P122035
  A_51_P123676
  A_51_P122582
  A_51_P128775
  A_51_P123705
  A_51_P122740
  A_51_P128775
  A_51_P108459
  A_51_P279552
  A_51_P125135
  A_51_P123920
  A_51_P117581
  A_51_P254855
  A_51_P279552
  A_51_P345649
  A_51_P335969
  A_51_P122035
  A_51_P106952
  A_51_P335969
  A_51_P123676
  A_51_P106144
  A_51_P106952
  A_51_P128336
  A_51_P111757
  A_51_P201480
  A_51_P106397
  A_51_P114878
  A_51_P122740
  A_51_P452153
  A_51_P452153
  A_51_P335969
  A_51_P125691
  A_51_P102355
  A_52_P420504
  A_51_P279552
  A_52_P420504
  A_51_P114878
  A_51_P254855
  A_52_P420504
  A_51_P110471
  A_51_P108020
  A_51_P111612
  A_51_P335969
  A_51_P104608
  A_51_P110395
  A_51_P112762
  A_51_P106952
  A_51_P411297
  A_52_P420504
  A_51_P452153
  A_51_P108020
  A_51_P335969
  A_51_P346445
  A_51_P110759
  A_51_P481788
  A_51_P113003
  A_51_P102911
  A_51_P111757
  A_51_P107362
  A_52_P214630
  A_51_P163444
  A_51_P452153
  A_51_P172231
  A_51_P115178
  A_51_P109144
  A_52_P214630
  A_51_P107782
  A_51_P254855
  A_51_P128336
  A_51_P118603
  A_51_P111612
  A_51_P346445
  A_51_P122740
  A_51_P107362
  A_51_P110395
  A_51_P109171
  A_51_P128463
  A_51_P105927
  A_51_P126067
  A_51_P114878
  A_51_P114456
  A_51_P110395
  A_51_P101460
  A_51_P254855
  A_51_P279552
  A_51_P102911
  A_51_P117226
  A_51_P102355
  A_51_P105927
  A_51_P128336
  A_51_P110471
  A_52_P214630
  A_51_P112308
  A_51_P108489
  A_51_P100787
  A_52_P174915
  A_51_P112308
  A_51_P101460
  A_51_P114878
  A_51_P121390
  A_51_P105927
  A_51_P452153
  A_51_P111612
  A_52_P420504
  A_51_P211491
  A_51_P110759
  A_51_P102789
  A_51_P128336
  A_51_P118763
  A_51_P128775
  A_51_P109171
  A_51_P335969
  A_52_P174915
  A_51_P335969
  A_52_P214630
  A_51_P110471
  A_51_P122582
  A_51_P113722
  A_51_P107362
  A_51_P346445
  A_51_P116601
  A_51_P116601
  A_51_P101460
  A_52_P214630
  A_52_P214630
  A_51_P117226
  A_51_P110395
  A_51_P104891
  A_51_P198434
  A_51_P113403
  A_51_P101460
  A_51_P100787
  A_51_P108020
  A_51_P106259
  A_51_P125648
  A_51_P111757
  A_51_P399845
  A_51_P125135
  A_51_P109369
  A_51_P309854
  A_51_P309854
  A_52_P420504
  A_51_P101460
  A_51_P110471
  A_51_P110759
  A_51_P111612
  A_51_P346445
  A_51_P114062
  A_51_P126835
  A_51_P126067
  A_51_P116601
  A_51_P335969
  A_51_P115817
  A_51_P309854
  A_52_P174915
  A_51_P109144
  A_51_P104125
  A_51_P163444
  A_51_P107782
  A_51_P128336
  A_51_P100787
  A_51_P101460
  A_51_P113403
  A_51_P109144
  A_51_P102911
  A_51_P115005
  A_51_P105927
  A_51_P113403
  A_51_P118539
  A_51_P101075
  A_51_P110759
  A_51_P102911
  A_51_P111612
  A_51_P126067
  A_52_P214630
  A_51_P117581
  A_51_P211491
  A_51_P115817
  A_51_P114634
  A_51_P122649
  A_51_P107362
  A_51_P452153
  A_51_P104608
  A_51_P102911
  A_51_P100787
  A_51_P102911
  A_51_P346445
  A_51_P123920
  A_51_P112308
  A_51_P335969
  A_51_P309854
  A_51_P106259
  A_51_P452153
  A_52_P174915
  A_51_P110471
  A_51_P110471
  A_51_P126067
  A_51_P113722
  A_51_P125467
  A_51_P110759
  A_51_P109171
  A_52_P174915
  A_51_P117581
  A_51_P128336
  A_51_P102911
  A_51_P106952
  A_51_P101460
  A_52_P214630
  A_51_P128876
  A_51_P113722
  A_51_P110471
  A_51_P239673
  A_51_P309854
  A_51_P121390
  A_51_P116601
  A_51_P106259
  A_51_P104608
  A_51_P110395
  A_51_P115005
  A_51_P101460
  A_51_P345649
  A_51_P128987
  A_51_P121390
  A_51_P110471
  A_51_P211491
  A_51_P121390
  A_51_P101460
  A_52_P237077
  A_51_P108020
  A_51_P117581
  A_51_P105927
  A_51_P105927
  A_51_P110759
  A_51_P110759
  A_51_P116601
  A_51_P123705
  A_51_P109144
  A_51_P116601
  A_51_P112308
  A_51_P107782
  A_51_P108190
  A_51_P112308
  A_51_P109144
  A_52_P214630
  A_51_P309854
  A_51_P125691
  A_51_P116581
  A_51_P117226
  A_51_P116601
  A_51_P113722
  A_51_P118539
  A_51_P110471
  A_51_P114456
  A_51_P125691
  A_51_P113195
  A_51_P113722
  A_51_P505868
  A_51_P125135
  A_51_P128876
  A_51_P123705
  A_52_P237077
  A_51_P108489
  A_51_P122649
  A_51_P100174
  A_51_P113003
  A_51_P117581
  A_51_P126835
  A_51_P198434
  A_51_P118168
  A_51_P106259
  A_51_P105927
  A_51_P110759
  A_51_P116581
  A_51_P106397
  A_51_P121390
  A_51_P107362
  A_51_P116447
  A_51_P198434
  A_51_P107362
  A_51_P112445
  A_51_P116601
  A_51_P100174
  A_51_P122649
  A_51_P346445
  A_51_P452153
  A_51_P123676
  A_51_P239673
  A_51_P101075
  A_51_P118763
  A_51_P116581
  A_51_P106259
  A_51_P124254
  A_51_P137336
  A_51_P122740
  A_51_P114456
  A_51_P102911
  A_52_P174915
  A_51_P122649
  A_51_P128876
  A_52_P174915
  A_51_P126177
  A_51_P108489
  A_51_P128775
  A_51_P117581
  A_51_P309854
  A_51_P100174
  A_51_P118539
  A_51_P106952
  A_51_P137336
  A_51_P102911
  A_51_P211491
  A_51_P115005
  A_51_P125691
  A_51_P110395
  A_51_P118763
  A_51_P122035
  A_51_P116601
  A_51_P101075
  A_52_P369581
  A_51_P345649
  A_51_P104608
  A_51_P109171
  A_51_P126835
  A_51_P124254
  A_51_P108190
  A_51_P100174
  A_51_P125467
  A_51_P137336
  A_51_P122141
  A_51_P117581
  A_51_P104891
  A_51_P122649
  A_51_P107782
  A_51_P125467
  A_52_P174915
  A_51_P106952
  A_51_P106259
  A_51_P109171
  A_51_P109369
  A_51_P128987
  A_51_P137336
  A_51_P126835
  A_51_P239673
  A_51_P137336
  A_51_P163444
  A_51_P115005
  A_51_P100174
  A_51_P120615
  A_51_P118168
  A_51_P118763
  A_51_P101075
  A_51_P116581
  A_51_P109171
  A_51_P129317
  A_51_P117581
  A_51_P112308
  A_51_P112308
  A_51_P163444
  A_51_P137336
  A_51_P137336
  A_51_P128987
  A_51_P100174
  A_51_P116581
  A_51_P115005
  A_51_P239673
  A_52_P237077
  A_51_P110395
  A_51_P118763
  A_51_P114456
  A_51_P126177
  A_51_P113195
  A_51_P101075
  A_51_P137336
  A_51_P109144
  A_51_P198434
  A_51_P112308
  A_51_P114062
  A_51_P309854
  A_51_P122649
  A_51_P239673
  A_51_P126835
  A_51_P117226
  A_51_P107782
  A_51_P101075
  A_51_P114616
  A_51_P481788
  A_51_P100174
  A_51_P115005
  A_51_P122649
  A_51_P128876
  A_51_P118603
  A_51_P129317
  A_51_P117226
  A_51_P113003
  A_51_P122649
  A_51_P115817
  A_51_P113403
  A_51_P124254
  A_51_P100174
  A_51_P112355
  A_51_P102355
  A_51_P108489
  A_51_P126835
  A_51_P122582
  A_51_P128987
  A_51_P101075
  A_51_P116755
  A_51_P123705
  A_51_P113722
  A_51_P113403
  A_51_P101006
  A_51_P128987
  A_51_P101006
  A_51_P123705
  A_51_P114456
  A_51_P113403
  A_51_P106259
  A_51_P129317
  A_51_P113003
  A_52_P174915
  A_51_P110395
  A_51_P126626
  A_51_P198434
  A_52_P237077
  A_51_P163444
  A_51_P126835
  A_51_P100174
  A_52_P237077
  A_51_P112355
  A_51_P109369
  A_51_P123676
  A_51_P115817
  A_51_P117226
  A_51_P128336
  A_51_P107782
  A_51_P126626
  A_51_P117581
  A_51_P122845
  A_51_P118539
  A_51_P124606
  A_52_P237077
  A_51_P123705
  A_51_P104891
  A_51_P111544
  A_51_P105017
  A_51_P112817
  A_51_P122845
  A_51_P239673
  A_51_P122845
  A_51_P211491
  A_51_P122649
  A_51_P481788
  A_51_P108190
  A_51_P122035
  A_51_P108489
  A_51_P106259
  A_51_P115817
  A_51_P118168
  A_51_P110814
  A_51_P122035
  A_51_P124254
  A_51_P108190
  A_51_P106397
  A_51_P126067
  A_51_P116755
  A_51_P112308
  A_51_P122035
  A_51_P116755
  A_51_P125467
  A_52_P237077
  A_51_P106952
  A_51_P126626
  A_51_P117226
  A_51_P113195
  A_51_P124254
  A_51_P137336
  A_51_P128876
  A_51_P117130
  A_51_P125467
  A_51_P126626
  A_51_P113403
  A_51_P128876
  A_51_P118603
  A_51_P113003
  A_51_P105927
  A_51_P107362
  A_51_P122035
  A_51_P172231
  A_52_P237077
  A_51_P101006
  A_51_P106428
  A_51_P124254
  A_51_P128876
  A_51_P113195
  A_51_P101006
  A_51_P104608
  A_51_P110672
  A_51_P111544
  A_51_P112817
  A_51_P118539
  A_51_P211491
  A_51_P126626
  A_51_P124254
  A_51_P116581
  A_51_P239673
  A_51_P110672
  A_51_P112355
  A_51_P127756
  A_51_P101075
  A_51_P126626
  A_51_P115005
  A_51_P123676
  A_51_P111757
  A_51_P125467
  A_51_P109171
  A_51_P128336
  A_51_P127756
  A_51_P111952
  A_51_P124254
  A_51_P116755
  A_51_P163444
  A_51_P128987
  A_51_P118168
  A_51_P109369
  A_51_P106397
  A_51_P481788
  A_51_P112355
  A_51_P124254
  A_51_P124606
  A_51_P126177
  A_51_P128987
  A_51_P110814
  A_51_P109369
  A_51_P110672
  A_51_P106259
  A_51_P363947
  A_51_P104125
  A_51_P123676
  A_51_P112355
  A_51_P111757
  A_51_P115005
  A_51_P163444
  A_51_P126626
  A_51_P128876
  A_51_P104891
  A_51_P411297
  A_51_P128987
  A_51_P121275
  A_51_P163444
  A_51_P108489
  A_51_P211491
  A_51_P126177
  A_51_P118603
  A_52_P236448
  A_51_P126067
  A_51_P105017
  A_51_P468505
  A_51_P118539
  A_51_P113403
  A_51_P115817
  A_51_P111952
  A_51_P119597
  A_51_P126177
  A_51_P125691
  A_51_P116755
  A_51_P126626
  A_51_P128987
  A_51_P123920
  A_51_P106397
  A_51_P119597
  A_51_P101006
  A_51_P105709
  A_51_P115005
  A_51_P481788
  A_51_P129317
  A_51_P198434
  A_52_P237077
  A_51_P113195
  A_51_P123676
  A_51_P115817
  A_51_P107782
  A_51_P110301
  A_51_P117130
  A_51_P128147
  A_51_P110672
  A_51_P112677
  A_51_P101075
  A_51_P122845
  A_51_P363947
  A_51_P118539
  A_51_P111952
  A_51_P113195
  A_51_P126177
  A_51_P110672
  A_51_P126177
  A_51_P108190
  A_51_P122845
  A_51_P104608
  A_51_P122035
  A_51_P163444
  A_51_P123676
  A_51_P111612
  A_51_P102860
  A_52_P369581
  A_51_P107782
  A_51_P110672
  A_51_P123705
  A_51_P124606
  A_51_P128876
  A_51_P106428
  A_51_P117130
  A_51_P122246
  A_51_P100787
  A_51_P101660
  A_51_P113195
  A_51_P126067
  A_51_P125691
  A_51_P111544
  A_52_P42269
  A_51_P113403
  A_51_P111952
  A_51_P100787
  A_51_P109369
  A_51_P113195
  A_51_P111544
  A_51_P123676
  A_51_P211491
  A_51_P101006
  A_51_P464576
  A_51_P110814
  A_51_P125467
  A_51_P126177
  A_51_P408082
  A_51_P102355
  A_51_P112817
  A_51_P121275
  A_51_P102355
  A_51_P111544
  A_51_P111952
  A_51_P111952
  A_51_P112557
  A_51_P112817
  A_51_P239673
  A_51_P118539
  A_51_P112817
  A_51_P118763
  A_51_P111544
  A_51_P104891
  A_51_P112355
  A_51_P128147
  A_51_P118539
  A_51_P116616
  A_51_P105709
  A_51_P111757
  A_51_P123920
  A_51_P481788
  A_51_P124719
  A_51_P110301
  A_51_P129317
  A_51_P110814
  A_51_P110301
  A_51_P105709
  A_51_P122141
  A_51_P120615
  A_51_P129317
  A_51_P102355
  A_51_P118763
  A_51_P111544
  A_51_P126626
  A_51_P464576
  A_51_P363947
  A_51_P116581
  A_51_P119597
  A_51_P111952
  A_51_P125691
  A_52_P236448
  A_51_P107782
  A_51_P363947
  A_51_P110301
  A_51_P124568
  A_51_P123920
  A_51_P213099
  A_51_P108489
  A_51_P112557
  A_51_P116755
  A_51_P213099
  A_51_P118168
  A_51_P101006
  A_51_P117130
  A_51_P122845
  A_51_P109369
  A_51_P128147
  A_51_P115817
  A_51_P122246
  A_51_P117130
  A_51_P104891
  A_51_P108190
  A_51_P101006
  A_51_P115159
  A_51_P110672
  A_51_P110035
  A_51_P110814
  A_51_P111757
  A_51_P106428
  A_51_P116447
  A_51_P129317
  A_51_P122141
  A_51_P128775
  A_51_P116755
  A_51_P122845
  A_51_P198434
  A_51_P111544
  A_51_P128147
  A_51_P129317
  A_51_P122246
  A_51_P112355
  A_51_P363947
  A_51_P101228
  A_51_P464576
  A_51_P101006
  A_51_P115159
  A_51_P116581
  A_51_P110301
  A_51_P116616
  A_51_P109171
  A_51_P124719
  A_51_P114049
  A_51_P363947
  A_52_P369581
  A_51_P100573
  A_51_P116447
  A_51_P112355
  A_51_P483280
  A_51_P127756
  A_51_P106952
  A_51_P113003
  A_51_P110035
  A_51_P111952
  A_51_P213099
  A_51_P126835
  A_51_P110301
  A_51_P125467
  A_51_P108190
  A_51_P239673
  A_51_P101228
  A_52_P282762
  A_51_P107020
  A_51_P481788
  A_51_P112677
  A_51_P127756
  A_51_P109144
  A_51_P116755
  A_51_P114878
  A_51_P483280
  A_51_P108190
  A_51_P104891
  A_51_P345649
  A_51_P123920
  A_51_P125050
  A_51_P119597
  A_51_P127756
  A_51_P120461
  A_51_P483280
  A_51_P125050
  A_51_P112557
  A_51_P110035
  A_52_P236448
  A_51_P123920
  A_51_P116447
  A_51_P105017
  A_51_P198434
  A_51_P115817
  A_51_P122141
  A_52_P42269
  A_51_P399845
  A_51_P363947
  A_51_P483280
  A_51_P128147
  A_51_P363947
  A_51_P127756
  A_51_P109369
  A_51_P483280
  A_51_P111952
  A_51_P117130
  A_51_P481788
  A_51_P119597
  A_52_P369581
  A_51_P124568
  A_51_P112627
  A_51_P100573
  A_51_P399845
  A_51_P119597
  A_51_P115159
  A_51_P100573
  A_51_P123920
  A_51_P110672
  A_51_P109840
  A_51_P126067
  A_51_P113195
  A_51_P109144
  A_51_P106397
  A_51_P112557
  A_51_P110814
  A_51_P122246
  A_51_P104608
  A_51_P464576
  A_51_P112627
  A_51_P100573
  A_51_P110814
  A_52_P369581
  A_52_P42269
  A_51_P120461
  A_51_P211491
  A_51_P104891
  A_51_P110814
  A_51_P483280
  A_51_P118168
  A_51_P112627
  A_51_P127756
  A_51_P109369
  A_51_P109840
  A_51_P116616
  A_51_P116447
  A_51_P122246
  A_51_P107020
  A_51_P117130
  A_51_P104891
  A_51_P128147
  A_51_P363947
  A_51_P102355
  A_51_P124568
  A_51_P101228
  A_51_P123795
  A_52_P236448
  A_51_P107020
  A_52_P282762
  A_51_P464576
  A_51_P101660
  A_51_P110814
  A_51_P114456
  A_51_P122246
  A_51_P129317
  A_51_P122035
  A_52_P49250
  A_51_P399845
  A_51_P127756
  A_51_P107020
  A_51_P121275
  A_51_P411297
  A_51_P108489
  A_52_P42269
  A_51_P115159
  A_51_P118168
  A_51_P112627
  A_51_P109335
  A_51_P100787
  A_52_P49250
  A_51_P100573
  A_51_P121275
  A_51_P100573
  A_51_P109335
  A_51_P112677
  A_51_P121275
  A_52_P49250
  A_51_P125050
  A_51_P102860
  A_51_P127756
  A_51_P124568
  A_51_P106397
  A_51_P123795
  A_51_P112627
  A_51_P116616
  A_51_P114634
  A_51_P119401
  A_51_P110672
  A_52_P42269
  A_51_P125446
  A_52_P369581
  A_51_P116581
  A_52_P282762
  A_51_P117130
  A_51_P110301
  A_51_P116616
  A_51_P109840
  A_51_P112557
  A_51_P112627
  A_51_P127334
  A_51_P107020
  A_51_P111757
  A_51_P110301
  A_51_P124568
  A_51_P112627
  A_51_P105709
  A_51_P124568
  A_51_P124568
  A_51_P100573
  A_51_P105408
  A_51_P124568
  A_51_P127334
  A_51_P123795
  A_51_P122246
  A_51_P116616
  A_51_P100573
  A_52_P236448
  A_51_P125446
  A_51_P105709
  A_51_P105709
  A_51_P113003
  A_51_P191463
  A_51_P116447
  A_51_P123077
  A_51_P114854
  A_51_P112817
  A_51_P127334
  A_51_P114634
  A_51_P122845
  A_51_P116616
  A_51_P122246
  A_51_P119597
  A_51_P119597
  A_51_P124719
  A_51_P114634
  A_51_P123077
  A_51_P112677
  A_51_P100573
  A_52_P369581
  A_51_P112677
  A_51_P112355
  A_51_P114634
  A_51_P112817
  A_51_P109840
  A_51_P121275
  A_51_P108334
  A_51_P125446
  A_52_P38627
  A_51_P114634
  A_51_P120615
  A_51_P120093
  A_51_P198434
  A_51_P128147
  A_51_P121275
  A_51_P106428
  A_51_P100309
  A_51_P102860
  A_51_P109840
  A_51_P106428
  A_51_P109840
  A_51_P120717
  A_52_P42269
  A_51_P109421
  A_51_P103780
  A_51_P106428
  A_51_P114456
  A_51_P118168
  A_51_P106397
  A_51_P106397
  A_51_P107020
  A_51_P114456
  A_51_P101228
  A_51_P124568
  A_52_P38627
  A_51_P119401
  A_51_P112677
  A_52_P507214
  A_51_P105709
  A_51_P464576
  A_51_P124606
  A_51_P112677
  A_51_P112627
  A_51_P464576
  A_52_P507214
  A_52_P42269
  A_51_P109840
  A_51_P116326
  A_51_P109421
  A_51_P126177
  A_51_P122246
  A_51_P120093
  A_51_P128147
  A_51_P116616
  A_51_P483280
  A_52_P282762
  A_51_P100309
  A_51_P112627
  A_52_P38627
  A_52_P236448
  A_51_P101660
  A_51_P107020
  A_51_P316553
  A_51_P123795
  A_52_P38627
  A_52_P236448
  A_51_P101660
  A_51_P101660
  A_51_P106428
  A_51_P102355
  A_51_P114005
  A_51_P103780
  A_52_P38627
  A_51_P125050
  A_51_P106428
  A_51_P109840
  A_51_P191463
  A_51_P116326
  A_51_P112557
  A_51_P468505
  A_51_P110301
  A_51_P464576
  A_51_P119401
  A_51_P128499
  A_51_P114005
  A_52_P282762
  A_51_P411297
  A_51_P123795
  A_51_P123604
  A_51_P483280
  A_51_P115159
  A_51_P114005
  A_51_P127738
  A_51_P108334
  A_51_P123795
  A_51_P107020
  A_51_P107020
  A_51_P125050
  A_51_P101660
  A_51_P120717
  A_51_P123795
  A_51_P124719
  A_51_P123920
  A_51_P107090
  A_51_P399845
  A_51_P468505
  A_51_P121412
  A_51_P123017
  A_51_P116687
  A_51_P108190
  A_51_P123017
  A_51_P120461
  A_51_P114005
  A_52_P42269
  A_52_P369581
  A_51_P107090
  A_51_P123795
  A_51_P118650
  A_51_P108334
  A_51_P114634
  A_51_P119597
  A_51_P125446
  A_51_P123795
  A_51_P114005
  A_51_P124719
  A_51_P123017
  A_51_P211854
  A_51_P191463
  A_51_P120717
  A_51_P213099
  A_51_P399845
  A_51_P121818
  A_51_P114049
  A_52_P38627
  A_51_P125467
  A_51_P125050
  A_51_P102860
  A_51_P213099
  A_51_P128499
  A_52_P507214
  A_51_P109840
  A_51_P117130
  A_51_P102860
  A_51_P115159
  A_51_P464576
  A_51_P128147
  A_51_P399845
  A_51_P109335
  A_51_P120093
  A_51_P105709
  A_51_P105709
  A_52_P282762
  A_51_P112677
  A_52_P580582
  A_51_P114049
  A_51_P114005
  A_51_P125050
  A_51_P102518
  A_51_P103929
  A_51_P112817
  A_52_P49250
  A_52_P49250
  A_51_P109335
  A_51_P109171
  A_51_P120875
  A_52_P507214
  A_51_P213099
  A_51_P112817
  A_51_P117236
  A_51_P411297
  A_51_P316553
  A_51_P123017
  A_51_P114005
  A_52_P42269
  A_51_P483280
  A_51_P122141
  A_51_P115159
  A_51_P121818
  A_51_P123017
  A_51_P125446
  A_51_P118650
  A_51_P128499
  A_52_P507214
  A_51_P103929
  A_52_P282762
  A_51_P110035
  A_51_P114634
  A_51_P114049
  A_51_P316553
  A_51_P123077
  A_51_P116687
  A_51_P116421
  A_51_P123604
  A_52_P38627
  A_51_P213099
  A_51_P121275
  A_52_P236448
  A_51_P123017
  A_52_P38627
  A_51_P112677
  A_51_P123077
  A_51_P114634
  A_51_P123077
  A_51_P105408
  A_51_P116687
  A_51_P105408
  A_51_P121252
  A_51_P114049
  A_51_P105408
  A_52_P369581
  A_51_P115159
  A_51_P125691
  A_52_P49250
  A_51_P114005
  A_51_P108334
  A_51_P191669
  A_51_P124798
  A_51_P116687
  A_51_P116421
  A_51_P123017
  A_52_P38627
  A_51_P110888
  A_51_P109421
  A_51_P120461
  A_51_P316553
  A_51_P121412
  A_51_P191463
  A_51_P101228
  A_51_P191669
  A_51_P127334
  A_51_P191463
  A_51_P316553
  A_51_P116421
  A_52_P49250
  A_51_P109335
  A_51_P123077
  A_51_P128499
  A_51_P121275
  A_52_P507214
  A_51_P114966
  A_51_P118650
  A_51_P127915
  A_51_P114854
  A_51_P127915
  A_51_P116421
  A_51_P101196
  A_51_P114854
  A_51_P316553
  A_51_P124719
  A_51_P124719
  A_51_P102518
  A_51_P116687
  A_51_P116447
  A_51_P117604
  A_52_P507214
  A_51_P124798
  A_51_P115626
  A_52_P282762
  A_51_P101196
  A_51_P103780
  A_51_P124719
  A_51_P108334
  A_51_P112237
  A_51_P390715
  A_51_P115159
  A_51_P116616
  A_51_P122845
  A_51_P316553
  A_51_P411297
  A_51_P118650
  A_51_P117236
  A_51_P105408
  A_51_P120717
  A_51_P114456
  A_51_P105408
  A_51_P124606
  A_51_P306017
  A_51_P114854
  A_51_P121818
  A_51_P117236
  A_51_P316553
  A_51_P120066
  A_51_P124798
  A_51_P102860
  A_51_P390715
  A_51_P468505
  A_51_P123604
  A_51_P112557
  A_51_P108489
  A_51_P191669
  A_51_P125050
  A_51_P108862
  A_51_P123077
  A_51_P118650
  A_51_P127915
  A_51_P125446
  A_51_P120461
  A_51_P116687
  A_51_P105017
  A_51_P390715
  A_51_P481788
  A_51_P411297
  A_51_P127915
  A_51_P112557
  A_51_P114966
  A_51_P129100
  A_52_P580582
  A_51_P120461
  A_51_P390715
  A_51_P128499
  A_51_P120461
  A_51_P191463
  A_51_P123017
  A_51_P468505
  A_51_P111544
  A_51_P125446
  A_51_P122141
  A_51_P121252
  A_51_P103929
  A_51_P120093
  A_51_P128075
  A_51_P105408
  A_51_P108334
  A_51_P117739
  A_51_P128075
  A_52_P49250
  A_51_P120066
  A_51_P103929
  A_51_P121252
  A_51_P108862
  A_51_P123604
  A_51_P306017
  A_51_P127334
  A_51_P128075
  A_51_P118650
  A_51_P468505
  A_51_P125446
  A_51_P191669
  A_51_P127915
  A_51_P128075
  A_51_P114049
  A_51_P109421
  A_51_P120875
  A_51_P127915
  A_51_P123077
  A_51_P120717
  A_51_P112557
  A_51_P120461
  A_51_P102860
  A_51_P108334
  A_51_P114966
  A_51_P123604
  A_51_P101660
  A_51_P124798
  A_51_P123604
  A_51_P105408
  A_51_P120093
  A_51_P114966
  A_51_P116447
  A_51_P100327
  A_52_P49250
  A_51_P129100
  A_51_P114049
  A_51_P108334
  A_51_P100327
  A_51_P128075
  A_51_P105017
  A_51_P112237
  A_51_P128499
  A_51_P114966
  A_51_P114854
  A_51_P116326
  A_51_P108862
  A_51_P306017
  A_51_P121412
  A_51_P191463
  A_52_P580582
  A_51_P116687
  A_51_P108334
  A_51_P306017
  A_51_P211854
  A_51_P115626
  A_51_P123017
  A_51_P105017
  A_51_P109335
  A_51_P117236
  A_51_P125446
  A_51_P105408
  A_51_P119401
  A_51_P116421
  A_51_P116447
  A_51_P128075
  A_51_P100327
  A_51_P120875
  A_51_P114005
  A_51_P103780
  A_51_P103780
  A_51_P120875
  A_51_P120875
  A_51_P120066
  A_51_P120615
  A_51_P122141
  A_51_P109335
  A_51_P108862
  A_51_P118885
  A_51_P127738
  A_51_P124798
  A_51_P106428
  A_51_P120717
  A_51_P124606
  A_51_P116421
  A_51_P103780
  A_51_P117604
  A_51_P128075
  A_51_P120093
  A_51_P127334
  A_51_P127915
  A_51_P121302
  A_51_P411297
  A_51_P103718
  A_51_P121252
  A_51_P119401
  A_51_P390715
  A_51_P191463
  A_51_P108659
  A_51_P120093
  A_51_P121412
  A_51_P120093
  A_51_P120615
  A_51_P115626
  A_51_P100327
  A_51_P117604
  A_51_P107808
  A_51_P101196
  A_51_P127915
  A_51_P107090
  A_51_P120093
  A_51_P398235
  A_51_P103718
  A_51_P306017
  A_51_P107090
  A_51_P101196
  A_51_P390715
  A_51_P129100
  A_51_P390715
  A_51_P306017
  A_51_P115626
  A_51_P468505
  A_51_P102860
  A_51_P101228
  A_51_P116687
  A_51_P390715
  A_51_P114049
  A_51_P120717
  A_51_P117739
  A_51_P103718
  A_51_P117236
  A_51_P107090
  A_51_P191463
  A_51_P114966
  A_51_P117604
  A_51_P115626
  A_51_P109421
  A_51_P119401
  A_51_P115626
  A_51_P117604
  A_51_P119401
  A_51_P121302
  A_51_P120066
  A_51_P108581
  A_51_P128075
  A_51_P101228
  A_51_P118885
  A_51_P103780
  A_51_P109421
  A_51_P399845
  A_51_P114049
  A_51_P128075
  A_51_P101196
  A_51_P306017
  A_51_P127334
  A_51_P118168
  A_51_P115626
  A_51_P123604
  A_52_P236448
  A_51_P103718
  A_51_P108659
  A_51_P115626
  A_51_P127334
  A_51_P117236
  A_51_P114854
  A_51_P125050
  A_51_P108659
  A_51_P121302
  A_51_P121412
  A_51_P120875
  A_51_P390715
  A_52_P580582
  A_51_P108659
  A_51_P106799
  A_51_P119401
  A_51_P118650
  A_51_P103780
  A_51_P104430
  A_51_P119401
  A_51_P103718
  A_51_P211854
  A_51_P110035
  A_51_P116421
  A_51_P123604
  A_51_P103718
  A_51_P116326
  A_51_P121412
  A_51_P398235
  A_51_P120066
  A_51_P108659
  A_51_P103929
  A_51_P108659
  A_51_P114966
  A_51_P127320
  A_51_P121302
  A_51_P102860
  A_51_P117236
  A_52_P282762
  A_51_P123077
  A_51_P112237
  A_51_P120875
  A_51_P306017
  A_51_P120461
  A_52_P507214
  A_51_P120615
  A_51_P121252
  A_51_P116421
  A_51_P101228
  A_51_P101196
  A_51_P121412
  A_51_P117739
  A_52_P507214
  A_51_P468505
  A_51_P127841
  A_51_P101660
  A_51_P108659
  A_51_P101196
  A_51_P129100
  A_51_P211854
  A_51_P127320
  A_51_P127320
  A_51_P117604
  A_51_P108862
  A_51_P118650
  A_51_P124719
  A_51_P118650
  A_51_P121412
  A_51_P102518
  A_51_P124606
  A_51_P101660
  A_51_P108581
  A_51_P111462
  A_51_P316553
  A_51_P121818
  A_51_P100327
  A_51_P111462
  A_51_P114854
  A_52_P580582
  A_51_P127320
  A_51_P103757
  A_51_P127320
  A_51_P114966
  A_51_P117236
  A_51_P129100
  A_51_P124798
  A_51_P120875
  A_51_P103780
  A_51_P127320
  A_51_P120066
  A_51_P120066
  A_51_P139651
  A_51_P127915
  A_51_P112237
  A_51_P102507
  A_51_P125260
  A_51_P101228
  A_51_P100327
  A_51_P109421
  A_51_P116421
  A_51_P111462
  A_51_P139651
  A_51_P107090
  A_51_P120615
  A_51_P106799
  A_51_P119031
  A_51_P109421
  A_51_P121302
  A_51_P117236
  A_51_P119031
  A_51_P120615
  A_51_P211854
  A_51_P102507
  A_51_P103718
  A_51_P127320
  A_51_P119544
  A_51_P119544
  A_51_P127320
  A_51_P121252
  A_51_P121302
  A_51_P122085
  A_51_P211854
  A_51_P107090
  A_51_P102507
  A_51_P125260
  A_51_P100327
  A_51_P127320
  A_51_P213099
  A_51_P117739
  A_51_P102507
  A_51_P110035
  A_51_P125260
  A_51_P118885
  A_51_P114966
  A_51_P121818
  A_51_P109335
  A_51_P122085
  A_51_P125260
  A_51_P118885
  A_51_P109335
  A_51_P107090
  A_51_P100852
  A_51_P117604
  A_51_P100327
  A_51_P121302
  A_51_P119544
  A_51_P102507
  A_51_P121302
  A_51_P121252
  A_51_P129100
  A_51_P106799
  A_51_P113003
  A_51_P110035
  A_51_P106799
  A_51_P102503
  A_51_P122085
  A_51_P122085
  A_51_P114854
  A_51_P122085
  A_51_P398235
  A_51_P108659
  A_51_P211854
  A_51_P114854
  A_51_P120717
  A_51_P116088
  A_51_P108862
  A_51_P103757
  A_51_P121818
  A_51_P398235
  A_51_P116687
  A_51_P101196
  A_51_P139651
  A_51_P398235
  A_51_P119031
  A_51_P121818
  A_52_P580582
  A_51_P122085
  A_51_P139651
  A_51_P112237
  A_51_P115626
  A_51_P126525
  A_51_P127334
  A_51_P108862
  A_51_P108862
  A_51_P124798
  A_51_P112237
  A_51_P106799
  A_51_P103718
  A_51_P139651
  A_51_P121818
  A_51_P127841
  A_51_P127841
  A_51_P117739
  A_51_P103757
  A_51_P125260
  A_51_P107090
  A_51_P109421
  A_51_P122425
  A_51_P111462
  A_51_P102507
  A_51_P121412
  A_51_P129100
  A_51_P117604
  A_51_P103757
  A_51_P100852
  A_51_P103929
  A_51_P119544
  A_51_P241667
  A_51_P121302
  A_51_P100852
  A_51_P101196
  A_51_P108659
  A_51_P126525
  A_51_P103757
  A_51_P139651
  A_51_P121252
  A_51_P116088
  A_51_P139651
  A_51_P118885
  A_51_P107808
  A_51_P121818
  A_51_P105017
  A_51_P117739
  A_51_P103718
  A_51_P110035
  A_51_P100852
  A_51_P124606
  A_51_P125260
  A_51_P119544
  A_51_P100852
  A_51_P111462
  A_51_P107808
  A_51_P120066
  A_51_P120717
  A_51_P119544
  A_51_P111462
  A_51_P125260
  A_51_P119544
  A_51_P120875
  A_52_P228236
  A_51_P113784
  A_51_P119544
  A_51_P211854
  A_51_P116088
  A_51_P102507
  A_51_P105017
  A_51_P112237
  A_51_P117739
  A_51_P120066
  A_51_P122425
  A_51_P127841
  A_51_P116088
  A_51_P122085
  A_51_P118223
  A_51_P117604
  A_51_P432403
  A_51_P398235
  A_51_P124798
  A_51_P139651
  A_51_P106799
  A_51_P136888
  A_51_P103757
  A_51_P118223
  A_51_P104933
  A_51_P116088
  A_51_P139651
  A_51_P108581
  A_51_P104933
  A_51_P124798
  A_51_P117739
  A_51_P121252
  A_51_P127841
  A_51_P104430
  A_51_P118223
  A_51_P116088
  A_51_P126525
  A_51_P125260
  A_51_P119031
  A_51_P116088
  A_52_P580582
  A_51_P111462
  A_52_P580582
  A_51_P106799
  A_51_P122085
  A_51_P116088
  A_51_P114177
  A_51_P103757
  A_51_P104933
  A_51_P112237
  A_51_P127841
  A_51_P127841
  A_51_P111462
  A_51_P104933
  A_51_P122425
  A_51_P126525
  A_51_P100327
  A_51_P106799
  A_51_P126525
  A_51_P398235
  A_51_P104430
  A_51_P108862
  A_51_P122085
  A_51_P468505
  A_51_P117739
  A_51_P102507
  A_51_P103757
  A_51_P211854
  A_51_P113784
  A_51_P125260
  A_51_P213099
  A_51_P118223
  A_51_P398235
  A_51_P116326
  A_51_P104933
  A_51_P107808
  A_51_P100852
  A_51_P108581
  A_51_P119031
  A_51_P122238
  A_51_P102507
  A_51_P104933
  A_51_P113784
  A_51_P116007
  A_52_P580582
  A_51_P124606
  A_51_P104933
  A_51_P129100
  A_52_P228236
  A_51_P136888
  A_51_P114177
  A_51_P110035
  A_51_P306017
  A_51_P129100
  A_51_P105017
  A_51_P108581
  A_51_P102503
  A_51_P113784
  A_51_P118885
  A_52_P228236
  A_51_P106859
  A_51_P118885
  A_51_P104933
  A_51_P116088
  A_51_P108581
  A_51_P120615
  A_51_P104933
  A_51_P103757
  A_52_P228236
  A_51_P126525
  A_52_P228236
  A_52_P228236
  A_51_P118885
  A_51_P107808
  A_51_P108581
  A_51_P113784
  A_51_P127841
  A_51_P111462
  A_51_P110931
  A_51_P241667
  A_51_P398235
  A_51_P122425
  A_51_P102503
  A_51_P119544
  A_51_P113072
  A_51_P114177
  A_51_P114177
  A_51_P241667
  A_51_P110931
  A_51_P112237
  A_51_P126525
  A_51_P136888
  A_51_P123604
  A_51_P103929
  A_52_P228236
  A_51_P103929
  A_51_P106859
  A_51_P106799
  A_51_P114177
  A_51_P119031
  A_51_P100852
  A_51_P118885
  A_51_P126525
  A_51_P110931
  A_51_P113072
  A_51_P241667
  A_51_P116326
  A_51_P108853
  A_51_P110931
  A_51_P116326
  A_51_P118223
  A_51_P125183
  A_51_P106859
  A_51_P113072
  A_51_P122723
  A_51_P122425
  A_51_P113072
  A_51_P122723
  A_51_P108581
  A_51_P106859
  A_51_P136888
  A_51_P241667
  A_51_P103929
  A_51_P110931
  A_51_P136888
  A_51_P114177
  A_51_P104430
  A_51_P122238
  A_51_P127841
  A_51_P122238
  A_51_P126525
  A_51_P106859
  A_51_P107433
  A_51_P119031
  A_51_P106859
  A_51_P122425
  A_51_P118223
  A_51_P136888
  A_51_P107433
  A_51_P107808
  A_51_P100852
  A_51_P116326
  A_51_P125183
  A_52_P228236
  A_51_P122723
  A_51_P122723
  A_51_P106059
  A_51_P122723
  A_51_P113784
  A_51_P118223
  A_51_P113784
  A_51_P122425
  A_51_P107433
  A_51_P107808
  A_51_P119031
  A_51_P122238
  A_51_P122238
  A_51_P122425
  A_51_P125183
  A_51_P136888
  A_51_P241667
  A_51_P106859
  A_51_P122238
  A_52_P228236
  A_51_P125183
  A_51_P108853
  A_51_P113784
  A_51_P114177
  A_51_P113072
  A_51_P107808
  A_51_P108853
  A_51_P113784
  A_51_P108581
  A_51_P122723
  A_51_P125183
  A_51_P241667
  A_51_P106859
  A_51_P114177
  A_51_P122723
  A_51_P118223
  A_51_P125183
  A_51_P117995
  A_51_P106859
  A_51_P241667
  A_51_P122425
  A_51_P108853
  A_51_P136888
  A_51_P136888
  A_51_P108853
  A_51_P118223
  A_51_P102503
  A_51_P125183
  A_51_P122238
  A_51_P108853
  A_51_P122238
  A_51_P102503
  A_51_P114177
  A_51_P119031
  A_51_P117995
  A_51_P122238
  A_51_P122723
  A_51_P107433
  A_51_P241667
  A_51_P107433
  A_51_P106059
  A_51_P437327
  A_51_P122723
  A_51_P437327
  A_51_P113072
  A_51_P106059
  A_51_P113072
  A_51_P107433
  A_51_P110931
  A_51_P110931
  A_51_P110931
  A_51_P117995
  A_51_P107433
  A_51_P102503
  A_51_P108853
  A_51_P102503
  A_51_P117995
  A_51_P125183
  A_51_P108853
  A_51_P107433
  A_51_P108853
  A_51_P107808
  A_51_P117995
  A_51_P437327
  A_51_P106059
  A_51_P102503
  A_51_P113072
  A_51_P107433
  A_51_P117995
  A_51_P437327
  A_51_P127297
  A_51_P117995
  A_51_P113072
  A_51_P127297
  A_51_P127297
  A_51_P217498
  A_51_P106059
  A_51_P127297
  A_51_P106059
  A_51_P127297
  A_51_P127297
  A_51_P102503
  A_51_P128648
  A_51_P217498
  A_51_P127297
  A_51_P117995
  A_51_P125183
  A_51_P127297
  A_51_P106059
  A_51_P128648
  A_51_P437327
  A_51_P127297
  A_51_P110931
  A_51_P128648
  A_51_P106059
  A_51_P217498
  A_51_P128648
  A_51_P112223
  A_51_P217498
  A_51_P100828
  A_51_P128648
  A_51_P128648
  A_51_P217498
  A_51_P100828
  A_51_P437327
  A_51_P112223
  A_51_P117995
  A_51_P110841
  A_51_P100828
  A_51_P437327
  A_51_P112223
  A_51_P217498
  A_51_P100828
  A_51_P110841
  A_51_P128648
  A_51_P128648
  A_51_P456957
  A_51_P456957
  A_51_P456957
  A_51_P456957
  A_51_P100828
  A_51_P103975
  A_51_P100828
  A_51_P110841
  A_51_P437327
  A_51_P103975
  A_51_P128648
  A_51_P217498
  A_51_P103975
  A_51_P100828
  A_51_P456957
  A_51_P456957
  A_51_P112223
  A_51_P456957
  A_51_P112223
  A_51_P103975
  A_51_P217498
  A_51_P112223
  A_51_P217498
  A_51_P110841
  A_51_P100828
  A_51_P103975
  A_51_P103975
  A_51_P106059
  A_51_P456957
  A_51_P110841
  A_51_P110841
  A_51_P456957
  A_51_P103975
  A_51_P103975
  A_51_P103975
  A_51_P112223
  A_51_P110841
  A_51_P112223
  A_51_P110841
  A_51_P110841
  A_51_P100828
  A_51_P112223
  A_51_P114693
  A_51_P114693
  A_51_P114693
  A_51_P114693
  A_51_P114693
  A_51_P114693
  A_51_P114693
  A_51_P114693
  A_51_P114693
  ```

---

Report: my\_analysis.GseaPreranked.1365164922288.rpt   by user: DerousD

xtools.gsea.GseaPreranked [Fri, Apr 5, '13 2 PM 28]

Website: www.broadinstitute.org/GSEA
Questions & Suggestions: Email
