# Supplementary material for: Network-based integration of molecular and physiological data elucidates regulatory mechanisms underlying adaptation to high-fat diet
Source: Genes Nutr. 2015 May 28;10(4):22. doi: 10.1007/s12263-015-0470-6 (PMC4446272; doi:10.1007/s12263-015-0470-6)
Supplement: Supplementary file 4 — Supplementary material 4 (ZIP 6984 kb) [file 12263_2015_470_MOESM4_ESM.zip › HF LF 5 d GSEA result/LOCOMOTORY_BEHAVIOR.html]

Details for gene set LOCOMOTORY\_BEHAVIOR[GSEA]

|  || Dataset | comp\_HF5d-LF5d\_collapsed |
| Phenotype | NoPhenotypeAvailable |
| Upregulated in class | na\_pos |
| GeneSet | LOCOMOTORY\_BEHAVIOR |
| Enrichment Score (ES) | 0.52914727 |
| Normalized Enrichment Score (NES) | 1.9890254 |
| Nominal p-value | 0.0 |
| FDR q-value | 0.01296407 |
| FWER p-Value | 0.09 |
Table: GSEA Results Summary

  

Fig 1: Enrichment plot: LOCOMOTORY\_BEHAVIOR      
 Profile of the Running ES Score & Positions of GeneSet Members on the Rank Ordered List

  

| PROBE | GENE SYMBOL | GENE\_TITLE | RANK IN GENE LIST | RANK METRIC SCORE | RUNNING ES | CORE ENRICHMENT || 1 | CCL8 |  |  | 56 | 2.814 | 0.0635 | Yes |
| 2 | CCL7 |  |  | 128 | 2.452 | 0.1157 | Yes |
| 3 | CCR2 |  |  | 219 | 2.188 | 0.1586 | Yes |
| 4 | CCL5 |  |  | 241 | 2.144 | 0.2100 | Yes |
| 5 | CCR5 |  |  | 244 | 2.139 | 0.2641 | Yes |
| 6 | PF4 |  |  | 290 | 2.044 | 0.3096 | Yes |
| 7 | SPN |  |  | 342 | 1.962 | 0.3522 | Yes |
| 8 | CXCL14 |  |  | 428 | 1.821 | 0.3864 | Yes |
| 9 | CCBP2 |  |  | 498 | 1.707 | 0.4200 | Yes |
| 10 | CXCR3 |  |  | 527 | 1.680 | 0.4587 | Yes |
| 11 | TGFB2 |  |  | 572 | 1.624 | 0.4937 | Yes |
| 12 | CCL2 |  |  | 875 | 1.330 | 0.4846 | Yes |
| 13 | CCL17 |  |  | 986 | 1.243 | 0.5006 | Yes |
| 14 | CCR3 |  |  | 1095 | 1.159 | 0.5147 | Yes |
| 15 | DOCK2 |  |  | 1191 | 1.098 | 0.5291 | Yes |
| 16 | CX3CL1 |  |  | 1442 | 0.942 | 0.5176 | No |
| 17 | ITGB2 |  |  | 1794 | 0.753 | 0.4870 | No |
| 18 | RALBP1 |  |  | 1893 | 0.694 | 0.4908 | No |
| 19 | PLAU |  |  | 1938 | 0.671 | 0.5016 | No |
| 20 | CXCR4 |  |  | 2015 | 0.626 | 0.5067 | No |
| 21 | CXCL13 |  |  | 2207 | 0.528 | 0.4930 | No |
| 22 | PLAUR |  |  | 2391 | 0.447 | 0.4784 | No |
| 23 | CXCL9 |  |  | 2452 | 0.418 | 0.4806 | No |
| 24 | CCL11 |  |  | 2459 | 0.413 | 0.4902 | No |
| 25 | CX3CR1 |  |  | 2643 | 0.326 | 0.4725 | No |
| 26 | CXCL12 |  |  | 2746 | 0.280 | 0.4652 | No |
| 27 | CDH13 |  |  | 2816 | 0.246 | 0.4617 | No |
| 28 | CCL24 |  |  | 2825 | 0.243 | 0.4667 | No |
| 29 | CCRL1 |  |  | 2863 | 0.234 | 0.4674 | No |
| 30 | NOVA1 |  |  | 3126 | 0.106 | 0.4330 | No |
| 31 | CCL25 |  |  | 4155 | -0.381 | 0.2969 | No |
| 32 | MAPK1 |  |  | 4676 | -0.606 | 0.2386 | No |
| 33 | FGF2 |  |  | 4723 | -0.629 | 0.2481 | No |
| 34 | RALA |  |  | 5411 | -0.982 | 0.1757 | No |
| 35 | PIK3CB |  |  | 5683 | -1.143 | 0.1663 | No |
| 36 | MAP2K1 |  |  | 5962 | -1.304 | 0.1600 | No |
Table: GSEA details [plain text format]

  

Fig 2: LOCOMOTORY\_BEHAVIOR: Random ES distribution      
 Gene set null distribution of ES for **LOCOMOTORY\_BEHAVIOR**
