# Supplementary material for: Network-based integration of molecular and physiological data elucidates regulatory mechanisms underlying adaptation to high-fat diet
Source: Genes Nutr. 2015 May 28;10(4):22. doi: 10.1007/s12263-015-0470-6 (PMC4446272; doi:10.1007/s12263-015-0470-6)
Supplement: Supplementary file 4 — Supplementary material 4 (ZIP 6984 kb) [file 12263_2015_470_MOESM4_ESM.zip › HF LF 5 d GSEA result/MITOCHONDRIAL_ENVELOPE.html]

Details for gene set MITOCHONDRIAL\_ENVELOPE[GSEA]

|  || Dataset | comp\_HF5d-LF5d\_collapsed |
| Phenotype | NoPhenotypeAvailable |
| Upregulated in class | na\_neg |
| GeneSet | MITOCHONDRIAL\_ENVELOPE |
| Enrichment Score (ES) | -0.6274207 |
| Normalized Enrichment Score (NES) | -2.269719 |
| Nominal p-value | 0.0 |
| FDR q-value | 0.0 |
| FWER p-Value | 0.0 |
Table: GSEA Results Summary

  

Fig 1: Enrichment plot: MITOCHONDRIAL\_ENVELOPE      
 Profile of the Running ES Score & Positions of GeneSet Members on the Rank Ordered List

  

| PROBE | GENE SYMBOL | GENE\_TITLE | RANK IN GENE LIST | RANK METRIC SCORE | RUNNING ES | CORE ENRICHMENT || 1 | MCL1 |  |  | 17 | 3.532 | 0.0467 | No |
| 2 | BCL2 |  |  | 296 | 2.039 | 0.0357 | No |
| 3 | RAB11FIP5 |  |  | 502 | 1.705 | 0.0303 | No |
| 4 | GATM |  |  | 2393 | 0.447 | -0.2316 | No |
| 5 | MAOB |  |  | 2556 | 0.367 | -0.2495 | No |
| 6 | MPV17 |  |  | 3219 | 0.064 | -0.3426 | No |
| 7 | CASP7 |  |  | 3857 | -0.242 | -0.4296 | No |
| 8 | COX6B2 |  |  | 3909 | -0.267 | -0.4331 | No |
| 9 | OXA1L |  |  | 4256 | -0.429 | -0.4762 | No |
| 10 | RHOT2 |  |  | 4358 | -0.470 | -0.4840 | No |
| 11 | TIMM17A |  |  | 4569 | -0.561 | -0.5060 | No |
| 12 | ATP5E |  |  | 5386 | -0.967 | -0.6083 | No |
| 13 | PPOX |  |  | 5513 | -1.046 | -0.6117 | Yes |
| 14 | ABCB6 |  |  | 5523 | -1.049 | -0.5983 | Yes |
| 15 | SLC25A11 |  |  | 5729 | -1.168 | -0.6112 | Yes |
| 16 | NDUFA1 |  |  | 5747 | -1.178 | -0.5972 | Yes |
| 17 | SURF1 |  |  | 5786 | -1.195 | -0.5860 | Yes |
| 18 | ABCF2 |  |  | 5811 | -1.211 | -0.5725 | Yes |
| 19 | TIMM8B |  |  | 5951 | -1.296 | -0.5742 | Yes |
| 20 | COX15 |  |  | 5952 | -1.296 | -0.5562 | Yes |
| 21 | PHB |  |  | 5961 | -1.303 | -0.5392 | Yes |
| 22 | UCP3 |  |  | 5978 | -1.313 | -0.5232 | Yes |
| 23 | NDUFA2 |  |  | 6084 | -1.402 | -0.5186 | Yes |
| 24 | MTX2 |  |  | 6229 | -1.535 | -0.5176 | Yes |
| 25 | TIMM9 |  |  | 6309 | -1.610 | -0.5064 | Yes |
| 26 | SDHD |  |  | 6419 | -1.723 | -0.4979 | Yes |
| 27 | TIMM10 |  |  | 6492 | -1.809 | -0.4830 | Yes |
| 28 | MFN2 |  |  | 6501 | -1.822 | -0.4588 | Yes |
| 29 | NDUFA9 |  |  | 6519 | -1.848 | -0.4355 | Yes |
| 30 | UQCRC1 |  |  | 6576 | -1.924 | -0.4166 | Yes |
| 31 | NDUFAB1 |  |  | 6622 | -1.998 | -0.3952 | Yes |
| 32 | MRPL32 |  |  | 6687 | -2.106 | -0.3750 | Yes |
| 33 | NDUFA6 |  |  | 6716 | -2.167 | -0.3488 | Yes |
| 34 | NDUFS2 |  |  | 6777 | -2.265 | -0.3258 | Yes |
| 35 | ABCB7 |  |  | 6790 | -2.286 | -0.2957 | Yes |
| 36 | NDUFS4 |  |  | 6792 | -2.295 | -0.2639 | Yes |
| 37 | PMPCA |  |  | 6795 | -2.314 | -0.2320 | Yes |
| 38 | ALAS2 |  |  | 6802 | -2.335 | -0.2004 | Yes |
| 39 | ATP5B |  |  | 6814 | -2.361 | -0.1691 | Yes |
| 40 | CYCS |  |  | 6844 | -2.437 | -0.1393 | Yes |
| 41 | ACN9 |  |  | 6898 | -2.650 | -0.1099 | Yes |
| 42 | OPA1 |  |  | 6912 | -2.702 | -0.0742 | Yes |
| 43 | TIMM50 |  |  | 6924 | -2.767 | -0.0373 | Yes |
| 44 | NDUFS1 |  |  | 7068 | -4.370 | 0.0033 | Yes |
Table: GSEA details [plain text format]

  

Fig 2: MITOCHONDRIAL\_ENVELOPE: Random ES distribution      
 Gene set null distribution of ES for **MITOCHONDRIAL\_ENVELOPE**
