# Supplementary material for: Network-based integration of molecular and physiological data elucidates regulatory mechanisms underlying adaptation to high-fat diet
Source: Genes Nutr. 2015 May 28;10(4):22. doi: 10.1007/s12263-015-0470-6 (PMC4446272; doi:10.1007/s12263-015-0470-6)
Supplement: Supplementary file 4 — Supplementary material 4 (ZIP 6984 kb) [file 12263_2015_470_MOESM4_ESM.zip › HF LF 5 d GSEA result/MITOCHONDRIAL_INNER_MEMBRANE.html]

Details for gene set MITOCHONDRIAL\_INNER\_MEMBRANE[GSEA]

|  || Dataset | comp\_HF5d-LF5d\_collapsed |
| Phenotype | NoPhenotypeAvailable |
| Upregulated in class | na\_neg |
| GeneSet | MITOCHONDRIAL\_INNER\_MEMBRANE |
| Enrichment Score (ES) | -0.74685335 |
| Normalized Enrichment Score (NES) | -2.521824 |
| Nominal p-value | 0.0 |
| FDR q-value | 0.0 |
| FWER p-Value | 0.0 |
Table: GSEA Results Summary

  

Fig 1: Enrichment plot: MITOCHONDRIAL\_INNER\_MEMBRANE      
 Profile of the Running ES Score & Positions of GeneSet Members on the Rank Ordered List

  

| PROBE | GENE SYMBOL | GENE\_TITLE | RANK IN GENE LIST | RANK METRIC SCORE | RUNNING ES | CORE ENRICHMENT || 1 | MPV17 |  |  | 3219 | 0.064 | -0.4547 | No |
| 2 | COX6B2 |  |  | 3909 | -0.267 | -0.5472 | No |
| 3 | OXA1L |  |  | 4256 | -0.429 | -0.5880 | No |
| 4 | TIMM17A |  |  | 4569 | -0.561 | -0.6214 | No |
| 5 | ATP5E |  |  | 5386 | -0.967 | -0.7185 | No |
| 6 | PPOX |  |  | 5513 | -1.046 | -0.7164 | No |
| 7 | SLC25A11 |  |  | 5729 | -1.168 | -0.7246 | Yes |
| 8 | NDUFA1 |  |  | 5747 | -1.178 | -0.7045 | Yes |
| 9 | SURF1 |  |  | 5786 | -1.195 | -0.6870 | Yes |
| 10 | TIMM8B |  |  | 5951 | -1.296 | -0.6855 | Yes |
| 11 | COX15 |  |  | 5952 | -1.296 | -0.6608 | Yes |
| 12 | PHB |  |  | 5961 | -1.303 | -0.6370 | Yes |
| 13 | UCP3 |  |  | 5978 | -1.313 | -0.6142 | Yes |
| 14 | NDUFA2 |  |  | 6084 | -1.402 | -0.6023 | Yes |
| 15 | TIMM9 |  |  | 6309 | -1.610 | -0.6033 | Yes |
| 16 | SDHD |  |  | 6419 | -1.723 | -0.5858 | Yes |
| 17 | TIMM10 |  |  | 6492 | -1.809 | -0.5614 | Yes |
| 18 | NDUFA9 |  |  | 6519 | -1.848 | -0.5298 | Yes |
| 19 | UQCRC1 |  |  | 6576 | -1.924 | -0.5010 | Yes |
| 20 | NDUFAB1 |  |  | 6622 | -1.998 | -0.4692 | Yes |
| 21 | MRPL32 |  |  | 6687 | -2.106 | -0.4381 | Yes |
| 22 | NDUFA6 |  |  | 6716 | -2.167 | -0.4007 | Yes |
| 23 | NDUFS2 |  |  | 6777 | -2.265 | -0.3659 | Yes |
| 24 | ABCB7 |  |  | 6790 | -2.286 | -0.3240 | Yes |
| 25 | NDUFS4 |  |  | 6792 | -2.295 | -0.2803 | Yes |
| 26 | PMPCA |  |  | 6795 | -2.314 | -0.2364 | Yes |
| 27 | ALAS2 |  |  | 6802 | -2.335 | -0.1926 | Yes |
| 28 | ATP5B |  |  | 6814 | -2.361 | -0.1491 | Yes |
| 29 | OPA1 |  |  | 6912 | -2.702 | -0.1112 | Yes |
| 30 | TIMM50 |  |  | 6924 | -2.767 | -0.0600 | Yes |
| 31 | NDUFS1 |  |  | 7068 | -4.370 | 0.0033 | Yes |
Table: GSEA details [plain text format]

  

Fig 2: MITOCHONDRIAL\_INNER\_MEMBRANE: Random ES distribution      
 Gene set null distribution of ES for **MITOCHONDRIAL\_INNER\_MEMBRANE**
