# Supplementary material for: Network-based integration of molecular and physiological data elucidates regulatory mechanisms underlying adaptation to high-fat diet
Source: Genes Nutr. 2015 May 28;10(4):22. doi: 10.1007/s12263-015-0470-6 (PMC4446272; doi:10.1007/s12263-015-0470-6)
Supplement: Supplementary file 4 — Supplementary material 4 (ZIP 6984 kb) [file 12263_2015_470_MOESM4_ESM.zip › HF LF 5 d GSEA result/MITOCHONDRIAL_LUMEN.html]

Details for gene set MITOCHONDRIAL\_LUMEN[GSEA]

|  || Dataset | comp\_HF5d-LF5d\_collapsed |
| Phenotype | NoPhenotypeAvailable |
| Upregulated in class | na\_neg |
| GeneSet | MITOCHONDRIAL\_LUMEN |
| Enrichment Score (ES) | -0.7294044 |
| Normalized Enrichment Score (NES) | -2.2857478 |
| Nominal p-value | 0.0 |
| FDR q-value | 0.0 |
| FWER p-Value | 0.0 |
Table: GSEA Results Summary

  

Fig 1: Enrichment plot: MITOCHONDRIAL\_LUMEN      
 Profile of the Running ES Score & Positions of GeneSet Members on the Rank Ordered List

  

| PROBE | GENE SYMBOL | GENE\_TITLE | RANK IN GENE LIST | RANK METRIC SCORE | RUNNING ES | CORE ENRICHMENT || 1 | NR3C1 |  |  | 1358 | 0.992 | -0.1704 | No |
| 2 | MRPL52 |  |  | 3284 | 0.034 | -0.4420 | No |
| 3 | MRPS28 |  |  | 4086 | -0.350 | -0.5476 | No |
| 4 | MRPS12 |  |  | 4220 | -0.417 | -0.5573 | No |
| 5 | NFS1 |  |  | 4999 | -0.762 | -0.6506 | No |
| 6 | MRPL10 |  |  | 5157 | -0.835 | -0.6545 | No |
| 7 | MRPL51 |  |  | 5432 | -0.997 | -0.6714 | No |
| 8 | MRPS22 |  |  | 5635 | -1.108 | -0.6756 | No |
| 9 | MRPS11 |  |  | 5703 | -1.154 | -0.6598 | No |
| 10 | MRPS24 |  |  | 5988 | -1.316 | -0.6710 | No |
| 11 | ACADM |  |  | 6098 | -1.415 | -0.6554 | No |
| 12 | NDUFAB1 |  |  | 6622 | -1.998 | -0.6855 | Yes |
| 13 | DBT |  |  | 6642 | -2.025 | -0.6438 | Yes |
| 14 | TIMM44 |  |  | 6726 | -2.182 | -0.6076 | Yes |
| 15 | MRPS18A |  |  | 6734 | -2.201 | -0.5603 | Yes |
| 16 | MRPL12 |  |  | 6763 | -2.250 | -0.5148 | Yes |
| 17 | BCKDK |  |  | 6779 | -2.268 | -0.4672 | Yes |
| 18 | BCKDHA |  |  | 6809 | -2.351 | -0.4197 | Yes |
| 19 | GRPEL1 |  |  | 6857 | -2.479 | -0.3719 | Yes |
| 20 | SUPV3L1 |  |  | 6882 | -2.588 | -0.3185 | Yes |
| 21 | CS |  |  | 7027 | -3.552 | -0.2609 | Yes |
| 22 | MRPS15 |  |  | 7048 | -3.825 | -0.1797 | Yes |
| 23 | ALDH4A1 |  |  | 7056 | -4.141 | -0.0898 | Yes |
| 24 | MRPS35 |  |  | 7063 | -4.309 | 0.0040 | Yes |
Table: GSEA details [plain text format]

  

Fig 2: MITOCHONDRIAL\_LUMEN: Random ES distribution      
 Gene set null distribution of ES for **MITOCHONDRIAL\_LUMEN**
