# Supplementary material for: Network-based integration of molecular and physiological data elucidates regulatory mechanisms underlying adaptation to high-fat diet
Source: Genes Nutr. 2015 May 28;10(4):22. doi: 10.1007/s12263-015-0470-6 (PMC4446272; doi:10.1007/s12263-015-0470-6)
Supplement: Supplementary file 4 — Supplementary material 4 (ZIP 6984 kb) [file 12263_2015_470_MOESM4_ESM.zip › HF LF 5 d GSEA result/MITOCHONDRIAL_PART.html]

Details for gene set MITOCHONDRIAL\_PART[GSEA]

|  || Dataset | comp\_HF5d-LF5d\_collapsed |
| Phenotype | NoPhenotypeAvailable |
| Upregulated in class | na\_neg |
| GeneSet | MITOCHONDRIAL\_PART |
| Enrichment Score (ES) | -0.6479756 |
| Normalized Enrichment Score (NES) | -2.5528924 |
| Nominal p-value | 0.0 |
| FDR q-value | 0.0 |
| FWER p-Value | 0.0 |
Table: GSEA Results Summary

  

Fig 1: Enrichment plot: MITOCHONDRIAL\_PART      
 Profile of the Running ES Score & Positions of GeneSet Members on the Rank Ordered List

  

| PROBE | GENE SYMBOL | GENE\_TITLE | RANK IN GENE LIST | RANK METRIC SCORE | RUNNING ES | CORE ENRICHMENT || 1 | MCL1 |  |  | 17 | 3.532 | 0.0282 | No |
| 2 | BCL2 |  |  | 296 | 2.039 | 0.0063 | No |
| 3 | RAB11FIP5 |  |  | 502 | 1.705 | -0.0081 | No |
| 4 | NR3C1 |  |  | 1358 | 0.992 | -0.1212 | No |
| 5 | GATM |  |  | 2393 | 0.447 | -0.2646 | No |
| 6 | MAOB |  |  | 2556 | 0.367 | -0.2844 | No |
| 7 | MPV17 |  |  | 3219 | 0.064 | -0.3781 | No |
| 8 | MRPL52 |  |  | 3284 | 0.034 | -0.3869 | No |
| 9 | CASP7 |  |  | 3857 | -0.242 | -0.4663 | No |
| 10 | COX6B2 |  |  | 3909 | -0.267 | -0.4712 | No |
| 11 | MRPS28 |  |  | 4086 | -0.350 | -0.4932 | No |
| 12 | MRPS12 |  |  | 4220 | -0.417 | -0.5086 | No |
| 13 | OXA1L |  |  | 4256 | -0.429 | -0.5098 | No |
| 14 | RHOT2 |  |  | 4358 | -0.470 | -0.5201 | No |
| 15 | TIMM17A |  |  | 4569 | -0.561 | -0.5452 | No |
| 16 | NFS1 |  |  | 4999 | -0.762 | -0.5996 | No |
| 17 | MRPL10 |  |  | 5157 | -0.835 | -0.6147 | No |
| 18 | ATP5E |  |  | 5386 | -0.967 | -0.6388 | No |
| 19 | MRPL51 |  |  | 5432 | -0.997 | -0.6366 | No |
| 20 | PPOX |  |  | 5513 | -1.046 | -0.6389 | Yes |
| 21 | ABCB6 |  |  | 5523 | -1.049 | -0.6311 | Yes |
| 22 | MRPS22 |  |  | 5635 | -1.108 | -0.6373 | Yes |
| 23 | MRPS11 |  |  | 5703 | -1.154 | -0.6368 | Yes |
| 24 | SLC25A11 |  |  | 5729 | -1.168 | -0.6303 | Yes |
| 25 | NDUFA1 |  |  | 5747 | -1.178 | -0.6225 | Yes |
| 26 | SURF1 |  |  | 5786 | -1.195 | -0.6176 | Yes |
| 27 | ABCF2 |  |  | 5811 | -1.211 | -0.6105 | Yes |
| 28 | TIMM8B |  |  | 5951 | -1.296 | -0.6190 | Yes |
| 29 | COX15 |  |  | 5952 | -1.296 | -0.6078 | Yes |
| 30 | PHB |  |  | 5961 | -1.303 | -0.5977 | Yes |
| 31 | UCP3 |  |  | 5978 | -1.313 | -0.5886 | Yes |
| 32 | MRPS24 |  |  | 5988 | -1.316 | -0.5784 | Yes |
| 33 | NDUFA2 |  |  | 6084 | -1.402 | -0.5798 | Yes |
| 34 | ACADM |  |  | 6098 | -1.415 | -0.5694 | Yes |
| 35 | MTX2 |  |  | 6229 | -1.535 | -0.5746 | Yes |
| 36 | TIMM9 |  |  | 6309 | -1.610 | -0.5719 | Yes |
| 37 | SDHD |  |  | 6419 | -1.723 | -0.5725 | Yes |
| 38 | TIMM10 |  |  | 6492 | -1.809 | -0.5671 | Yes |
| 39 | MFN2 |  |  | 6501 | -1.822 | -0.5524 | Yes |
| 40 | NDUFA9 |  |  | 6519 | -1.848 | -0.5388 | Yes |
| 41 | UQCRC1 |  |  | 6576 | -1.924 | -0.5301 | Yes |
| 42 | NDUFAB1 |  |  | 6622 | -1.998 | -0.5192 | Yes |
| 43 | DBT |  |  | 6642 | -2.025 | -0.5044 | Yes |
| 44 | MRPL32 |  |  | 6687 | -2.106 | -0.4924 | Yes |
| 45 | NDUFA6 |  |  | 6716 | -2.167 | -0.4776 | Yes |
| 46 | TIMM44 |  |  | 6726 | -2.182 | -0.4600 | Yes |
| 47 | MRPS18A |  |  | 6734 | -2.201 | -0.4419 | Yes |
| 48 | MRPL12 |  |  | 6763 | -2.250 | -0.4264 | Yes |
| 49 | NDUFS2 |  |  | 6777 | -2.265 | -0.4087 | Yes |
| 50 | BCKDK |  |  | 6779 | -2.268 | -0.3891 | Yes |
| 51 | ABCB7 |  |  | 6790 | -2.286 | -0.3708 | Yes |
| 52 | NDUFS4 |  |  | 6792 | -2.295 | -0.3510 | Yes |
| 53 | PMPCA |  |  | 6795 | -2.314 | -0.3313 | Yes |
| 54 | ALAS2 |  |  | 6802 | -2.335 | -0.3119 | Yes |
| 55 | BCKDHA |  |  | 6809 | -2.351 | -0.2924 | Yes |
| 56 | ATP5B |  |  | 6814 | -2.361 | -0.2725 | Yes |
| 57 | CYCS |  |  | 6844 | -2.437 | -0.2555 | Yes |
| 58 | GRPEL1 |  |  | 6857 | -2.479 | -0.2357 | Yes |
| 59 | SUPV3L1 |  |  | 6882 | -2.588 | -0.2167 | Yes |
| 60 | ACN9 |  |  | 6898 | -2.650 | -0.1959 | Yes |
| 61 | OPA1 |  |  | 6912 | -2.702 | -0.1743 | Yes |
| 62 | TIMM50 |  |  | 6924 | -2.767 | -0.1519 | Yes |
| 63 | CS |  |  | 7027 | -3.552 | -0.1357 | Yes |
| 64 | MRPS15 |  |  | 7048 | -3.825 | -0.1054 | Yes |
| 65 | ALDH4A1 |  |  | 7056 | -4.141 | -0.0705 | Yes |
| 66 | MRPS35 |  |  | 7063 | -4.309 | -0.0340 | Yes |
| 67 | NDUFS1 |  |  | 7068 | -4.370 | 0.0033 | Yes |
Table: GSEA details [plain text format]

  

Fig 2: MITOCHONDRIAL\_PART: Random ES distribution      
 Gene set null distribution of ES for **MITOCHONDRIAL\_PART**
