# Supplementary material for: Network-based integration of molecular and physiological data elucidates regulatory mechanisms underlying adaptation to high-fat diet
Source: Genes Nutr. 2015 May 28;10(4):22. doi: 10.1007/s12263-015-0470-6 (PMC4446272; doi:10.1007/s12263-015-0470-6)
Supplement: Supplementary file 4 — Supplementary material 4 (ZIP 6984 kb) [file 12263_2015_470_MOESM4_ESM.zip › HF LF 5 d GSEA result/MITOCHONDRION.html]

Details for gene set MITOCHONDRION[GSEA]

|  || Dataset | comp\_HF5d-LF5d\_collapsed |
| Phenotype | NoPhenotypeAvailable |
| Upregulated in class | na\_neg |
| GeneSet | MITOCHONDRION |
| Enrichment Score (ES) | -0.56894755 |
| Normalized Enrichment Score (NES) | -2.5504165 |
| Nominal p-value | 0.0 |
| FDR q-value | 0.0 |
| FWER p-Value | 0.0 |
Table: GSEA Results Summary

  

Fig 1: Enrichment plot: MITOCHONDRION      
 Profile of the Running ES Score & Positions of GeneSet Members on the Rank Ordered List

  

| PROBE | GENE SYMBOL | GENE\_TITLE | RANK IN GENE LIST | RANK METRIC SCORE | RUNNING ES | CORE ENRICHMENT || 1 | MCL1 |  |  | 17 | 3.532 | 0.0127 | No |
| 2 | OAT |  |  | 109 | 2.527 | 0.0104 | No |
| 3 | BCL2 |  |  | 296 | 2.039 | -0.0077 | No |
| 4 | PTRF |  |  | 493 | 1.711 | -0.0286 | No |
| 5 | RAB11FIP5 |  |  | 502 | 1.705 | -0.0224 | No |
| 6 | SLC25A12 |  |  | 578 | 1.620 | -0.0263 | No |
| 7 | CPT1A |  |  | 830 | 1.369 | -0.0566 | No |
| 8 | ARG2 |  |  | 889 | 1.319 | -0.0593 | No |
| 9 | DUT |  |  | 911 | 1.299 | -0.0568 | No |
| 10 | PMAIP1 |  |  | 1076 | 1.171 | -0.0754 | No |
| 11 | ATPIF1 |  |  | 1115 | 1.138 | -0.0760 | No |
| 12 | NR3C1 |  |  | 1358 | 0.992 | -0.1066 | No |
| 13 | PDK4 |  |  | 1726 | 0.791 | -0.1561 | No |
| 14 | SLC25A13 |  |  | 1947 | 0.666 | -0.1850 | No |
| 15 | AGPAT5 |  |  | 1970 | 0.653 | -0.1854 | No |
| 16 | AASS |  |  | 2239 | 0.508 | -0.2218 | No |
| 17 | BCL2L1 |  |  | 2343 | 0.469 | -0.2346 | No |
| 18 | BID |  |  | 2359 | 0.460 | -0.2348 | No |
| 19 | GATM |  |  | 2393 | 0.447 | -0.2377 | No |
| 20 | NME4 |  |  | 2414 | 0.436 | -0.2387 | No |
| 21 | PDK3 |  |  | 2430 | 0.429 | -0.2390 | No |
| 22 | GSTZ1 |  |  | 2518 | 0.387 | -0.2499 | No |
| 23 | MAOB |  |  | 2556 | 0.367 | -0.2536 | No |
| 24 | ASAH2 |  |  | 2712 | 0.295 | -0.2747 | No |
| 25 | SUCLG1 |  |  | 3033 | 0.148 | -0.3202 | No |
| 26 | MPV17 |  |  | 3219 | 0.064 | -0.3466 | No |
| 27 | BCL2L10 |  |  | 3255 | 0.047 | -0.3514 | No |
| 28 | MRPL52 |  |  | 3284 | 0.034 | -0.3553 | No |
| 29 | CPOX |  |  | 3666 | -0.145 | -0.4096 | No |
| 30 | SHMT2 |  |  | 3685 | -0.153 | -0.4115 | No |
| 31 | BBC3 |  |  | 3779 | -0.199 | -0.4241 | No |
| 32 | CASP7 |  |  | 3857 | -0.242 | -0.4341 | No |
| 33 | TFAM |  |  | 3893 | -0.257 | -0.4381 | No |
| 34 | COX6B2 |  |  | 3909 | -0.267 | -0.4391 | No |
| 35 | MRPS28 |  |  | 4086 | -0.350 | -0.4630 | No |
| 36 | SLC25A27 |  |  | 4123 | -0.365 | -0.4666 | No |
| 37 | PSEN1 |  |  | 4159 | -0.382 | -0.4700 | No |
| 38 | MRPS12 |  |  | 4220 | -0.417 | -0.4769 | No |
| 39 | NT5M |  |  | 4224 | -0.419 | -0.4755 | No |
| 40 | OXA1L |  |  | 4256 | -0.429 | -0.4781 | No |
| 41 | COQ4 |  |  | 4262 | -0.431 | -0.4770 | No |
| 42 | FDXR |  |  | 4308 | -0.451 | -0.4816 | No |
| 43 | RHOT2 |  |  | 4358 | -0.470 | -0.4866 | No |
| 44 | MSRB3 |  |  | 4378 | -0.478 | -0.4873 | No |
| 45 | SLC9A6 |  |  | 4512 | -0.538 | -0.5042 | No |
| 46 | COX7A1 |  |  | 4542 | -0.552 | -0.5060 | No |
| 47 | TIMM17A |  |  | 4569 | -0.561 | -0.5073 | No |
| 48 | MTHFD2 |  |  | 4595 | -0.571 | -0.5085 | No |
| 49 | COX8C |  |  | 4684 | -0.608 | -0.5186 | No |
| 50 | GOT2 |  |  | 4715 | -0.627 | -0.5202 | No |
| 51 | CASP8 |  |  | 4731 | -0.633 | -0.5196 | No |
| 52 | DHRS4 |  |  | 4757 | -0.644 | -0.5205 | No |
| 53 | POLRMT |  |  | 4850 | -0.693 | -0.5308 | No |
| 54 | ABCE1 |  |  | 4869 | -0.701 | -0.5304 | No |
| 55 | SLC25A4 |  |  | 4892 | -0.714 | -0.5305 | No |
| 56 | DIABLO |  |  | 4983 | -0.755 | -0.5402 | No |
| 57 | NFS1 |  |  | 4999 | -0.762 | -0.5391 | No |
| 58 | MTHFD1 |  |  | 5106 | -0.812 | -0.5509 | No |
| 59 | MRPL10 |  |  | 5157 | -0.835 | -0.5545 | No |
| 60 | TRNT1 |  |  | 5192 | -0.854 | -0.5558 | No |
| 61 | ACADS |  |  | 5253 | -0.889 | -0.5606 | No |
| 62 | FIBP |  |  | 5266 | -0.896 | -0.5585 | No |
| 63 | AK3 |  |  | 5276 | -0.902 | -0.5559 | No |
| 64 | PTS |  |  | 5292 | -0.913 | -0.5542 | No |
| 65 | ALDH5A1 |  |  | 5315 | -0.925 | -0.5534 | No |
| 66 | SCO1 |  |  | 5329 | -0.934 | -0.5513 | No |
| 67 | ATP5E |  |  | 5386 | -0.967 | -0.5552 | No |
| 68 | MRPL51 |  |  | 5432 | -0.997 | -0.5574 | No |
| 69 | PPOX |  |  | 5513 | -1.046 | -0.5645 | Yes |
| 70 | ABCB6 |  |  | 5523 | -1.049 | -0.5613 | Yes |
| 71 | MSRB2 |  |  | 5525 | -1.050 | -0.5569 | Yes |
| 72 | CLPX |  |  | 5566 | -1.070 | -0.5581 | Yes |
| 73 | GBAS |  |  | 5627 | -1.105 | -0.5620 | Yes |
| 74 | MRPS22 |  |  | 5635 | -1.108 | -0.5583 | Yes |
| 75 | MRPS11 |  |  | 5703 | -1.154 | -0.5630 | Yes |
| 76 | SLC25A11 |  |  | 5729 | -1.168 | -0.5616 | Yes |
| 77 | NDUFA1 |  |  | 5747 | -1.178 | -0.5590 | Yes |
| 78 | GPX4 |  |  | 5750 | -1.178 | -0.5542 | Yes |
| 79 | POLG |  |  | 5759 | -1.184 | -0.5503 | Yes |
| 80 | SURF1 |  |  | 5786 | -1.195 | -0.5489 | Yes |
| 81 | ABCF2 |  |  | 5811 | -1.211 | -0.5472 | Yes |
| 82 | TSFM |  |  | 5922 | -1.277 | -0.5576 | Yes |
| 83 | TBRG4 |  |  | 5948 | -1.292 | -0.5557 | Yes |
| 84 | TIMM8B |  |  | 5951 | -1.296 | -0.5504 | Yes |
| 85 | COX15 |  |  | 5952 | -1.296 | -0.5448 | Yes |
| 86 | PHB |  |  | 5961 | -1.303 | -0.5404 | Yes |
| 87 | UCP3 |  |  | 5978 | -1.313 | -0.5371 | Yes |
| 88 | MRPS24 |  |  | 5988 | -1.316 | -0.5327 | Yes |
| 89 | SOD2 |  |  | 6042 | -1.359 | -0.5346 | Yes |
| 90 | NDUFA2 |  |  | 6084 | -1.402 | -0.5345 | Yes |
| 91 | ACADM |  |  | 6098 | -1.415 | -0.5303 | Yes |
| 92 | NDUFA4 |  |  | 6150 | -1.463 | -0.5314 | Yes |
| 93 | MTX2 |  |  | 6229 | -1.535 | -0.5360 | Yes |
| 94 | SARS2 |  |  | 6272 | -1.574 | -0.5353 | Yes |
| 95 | TIMM9 |  |  | 6309 | -1.610 | -0.5336 | Yes |
| 96 | ACAT1 |  |  | 6372 | -1.670 | -0.5354 | Yes |
| 97 | NAPG |  |  | 6410 | -1.710 | -0.5334 | Yes |
| 98 | ACADSB |  |  | 6412 | -1.713 | -0.5262 | Yes |
| 99 | SDHD |  |  | 6419 | -1.723 | -0.5197 | Yes |
| 100 | HINT2 |  |  | 6489 | -1.809 | -0.5219 | Yes |
| 101 | TIMM10 |  |  | 6492 | -1.809 | -0.5144 | Yes |
| 102 | MFN2 |  |  | 6501 | -1.822 | -0.5078 | Yes |
| 103 | DECR1 |  |  | 6507 | -1.831 | -0.5006 | Yes |
| 104 | NDUFA8 |  |  | 6514 | -1.839 | -0.4936 | Yes |
| 105 | NDUFA9 |  |  | 6519 | -1.848 | -0.4863 | Yes |
| 106 | AMACR |  |  | 6543 | -1.877 | -0.4816 | Yes |
| 107 | MRPS31 |  |  | 6550 | -1.888 | -0.4743 | Yes |
| 108 | SLC22A4 |  |  | 6560 | -1.902 | -0.4675 | Yes |
| 109 | COX11 |  |  | 6569 | -1.914 | -0.4604 | Yes |
| 110 | UQCRC1 |  |  | 6576 | -1.924 | -0.4531 | Yes |
| 111 | BPHL |  |  | 6605 | -1.978 | -0.4486 | Yes |
| 112 | NDUFAB1 |  |  | 6622 | -1.998 | -0.4424 | Yes |
| 113 | SUCLA2 |  |  | 6640 | -2.022 | -0.4362 | Yes |
| 114 | DBT |  |  | 6642 | -2.025 | -0.4276 | Yes |
| 115 | MRPL32 |  |  | 6687 | -2.106 | -0.4249 | Yes |
| 116 | PCCB |  |  | 6688 | -2.107 | -0.4159 | Yes |
| 117 | NDUFA6 |  |  | 6716 | -2.167 | -0.4105 | Yes |
| 118 | TIMM44 |  |  | 6726 | -2.182 | -0.4025 | Yes |
| 119 | GHITM |  |  | 6730 | -2.185 | -0.3935 | Yes |
| 120 | MRPS18A |  |  | 6734 | -2.201 | -0.3845 | Yes |
| 121 | MRPL12 |  |  | 6763 | -2.250 | -0.3789 | Yes |
| 122 | ECHS1 |  |  | 6769 | -2.258 | -0.3700 | Yes |
| 123 | NDUFS2 |  |  | 6777 | -2.265 | -0.3613 | Yes |
| 124 | BCKDK |  |  | 6779 | -2.268 | -0.3517 | Yes |
| 125 | BAK1 |  |  | 6786 | -2.281 | -0.3428 | Yes |
| 126 | ABCB7 |  |  | 6790 | -2.286 | -0.3335 | Yes |
| 127 | NDUFS4 |  |  | 6792 | -2.295 | -0.3238 | Yes |
| 128 | NDUFA11 |  |  | 6793 | -2.302 | -0.3139 | Yes |
| 129 | PMPCA |  |  | 6795 | -2.314 | -0.3041 | Yes |
| 130 | ALAS2 |  |  | 6802 | -2.335 | -0.2950 | Yes |
| 131 | BCKDHA |  |  | 6809 | -2.351 | -0.2858 | Yes |
| 132 | ATP5B |  |  | 6814 | -2.361 | -0.2762 | Yes |
| 133 | CYCS |  |  | 6844 | -2.437 | -0.2700 | Yes |
| 134 | GRPEL1 |  |  | 6857 | -2.479 | -0.2611 | Yes |
| 135 | COQ7 |  |  | 6860 | -2.500 | -0.2507 | Yes |
| 136 | SUPV3L1 |  |  | 6882 | -2.588 | -0.2426 | Yes |
| 137 | PTGES2 |  |  | 6886 | -2.611 | -0.2319 | Yes |
| 138 | MTRF1 |  |  | 6892 | -2.631 | -0.2213 | Yes |
| 139 | ACN9 |  |  | 6898 | -2.650 | -0.2107 | Yes |
| 140 | ACP6 |  |  | 6910 | -2.687 | -0.2007 | Yes |
| 141 | OPA1 |  |  | 6912 | -2.702 | -0.1893 | Yes |
| 142 | TIMM50 |  |  | 6924 | -2.767 | -0.1790 | Yes |
| 143 | IDH3B |  |  | 6930 | -2.804 | -0.1678 | Yes |
| 144 | CYC1 |  |  | 6975 | -3.040 | -0.1611 | Yes |
| 145 | ACO2 |  |  | 6983 | -3.130 | -0.1487 | Yes |
| 146 | DLD |  |  | 6996 | -3.249 | -0.1365 | Yes |
| 147 | CS |  |  | 7027 | -3.552 | -0.1256 | Yes |
| 148 | MRPS15 |  |  | 7048 | -3.825 | -0.1121 | Yes |
| 149 | ALDH4A1 |  |  | 7056 | -4.141 | -0.0953 | Yes |
| 150 | MRPS35 |  |  | 7063 | -4.309 | -0.0778 | Yes |
| 151 | NDUFS1 |  |  | 7068 | -4.370 | -0.0596 | Yes |
| 152 | PDHA1 |  |  | 7086 | -6.605 | -0.0338 | Yes |
| 153 | PDK1 |  |  | 7087 | -8.013 | 0.0006 | Yes |
Table: GSEA details [plain text format]

  

Fig 2: MITOCHONDRION: Random ES distribution      
 Gene set null distribution of ES for **MITOCHONDRION**
