# Supplementary material for: Network-based integration of molecular and physiological data elucidates regulatory mechanisms underlying adaptation to high-fat diet
Source: Genes Nutr. 2015 May 28;10(4):22. doi: 10.1007/s12263-015-0470-6 (PMC4446272; doi:10.1007/s12263-015-0470-6)
Supplement: Supplementary file 4 — Supplementary material 4 (ZIP 6984 kb) [file 12263_2015_470_MOESM4_ESM.zip › HF LF 5 d GSEA result/ORGANELLE_MEMBRANE.html]

Details for gene set ORGANELLE\_MEMBRANE[GSEA]

|  || Dataset | comp\_HF5d-LF5d\_collapsed |
| Phenotype | NoPhenotypeAvailable |
| Upregulated in class | na\_neg |
| GeneSet | ORGANELLE\_MEMBRANE |
| Enrichment Score (ES) | -0.43748075 |
| Normalized Enrichment Score (NES) | -1.9209462 |
| Nominal p-value | 0.0 |
| FDR q-value | 0.006139384 |
| FWER p-Value | 0.148 |
Table: GSEA Results Summary

  

Fig 1: Enrichment plot: ORGANELLE\_MEMBRANE      
 Profile of the Running ES Score & Positions of GeneSet Members on the Rank Ordered List

  

| PROBE | GENE SYMBOL | GENE\_TITLE | RANK IN GENE LIST | RANK METRIC SCORE | RUNNING ES | CORE ENRICHMENT || 1 | MCL1 |  |  | 17 | 3.532 | 0.0192 | No |
| 2 | HERPUD1 |  |  | 151 | 2.366 | 0.0145 | No |
| 3 | EGFR |  |  | 222 | 2.185 | 0.0178 | No |
| 4 | BCL2 |  |  | 296 | 2.039 | 0.0198 | No |
| 5 | LRMP |  |  | 321 | 1.993 | 0.0285 | No |
| 6 | ASPH |  |  | 441 | 1.799 | 0.0224 | No |
| 7 | RAB11FIP5 |  |  | 502 | 1.705 | 0.0242 | No |
| 8 | LAMP2 |  |  | 647 | 1.541 | 0.0129 | No |
| 9 | GOLGA3 |  |  | 716 | 1.479 | 0.0122 | No |
| 10 | SCRN1 |  |  | 736 | 1.466 | 0.0185 | No |
| 11 | NUP160 |  |  | 750 | 1.450 | 0.0255 | No |
| 12 | AADAC |  |  | 831 | 1.368 | 0.0223 | No |
| 13 | ATP7A |  |  | 847 | 1.355 | 0.0284 | No |
| 14 | RPN1 |  |  | 874 | 1.332 | 0.0329 | No |
| 15 | GLG1 |  |  | 1007 | 1.223 | 0.0214 | No |
| 16 | NUP153 |  |  | 1025 | 1.216 | 0.0264 | No |
| 17 | LBR |  |  | 1127 | 1.134 | 0.0188 | No |
| 18 | NUP98 |  |  | 1197 | 1.097 | 0.0156 | No |
| 19 | KRTCAP2 |  |  | 1304 | 1.026 | 0.0066 | No |
| 20 | EDEM1 |  |  | 1416 | 0.958 | -0.0035 | No |
| 21 | MRVI1 |  |  | 1430 | 0.952 | 0.0005 | No |
| 22 | PLP2 |  |  | 1754 | 0.777 | -0.0412 | No |
| 23 | VAMP4 |  |  | 1882 | 0.699 | -0.0552 | No |
| 24 | KPNB1 |  |  | 2011 | 0.628 | -0.0697 | No |
| 25 | EPC1 |  |  | 2042 | 0.608 | -0.0703 | No |
| 26 | MAP1LC3A |  |  | 2170 | 0.545 | -0.0852 | No |
| 27 | EXT1 |  |  | 2447 | 0.419 | -0.1223 | No |
| 28 | CLN5 |  |  | 2503 | 0.396 | -0.1278 | No |
| 29 | DERL2 |  |  | 2520 | 0.385 | -0.1278 | No |
| 30 | RER1 |  |  | 2586 | 0.350 | -0.1350 | No |
| 31 | SLC33A1 |  |  | 2604 | 0.343 | -0.1353 | No |
| 32 | PCSK7 |  |  | 2607 | 0.340 | -0.1335 | No |
| 33 | PIGF |  |  | 2612 | 0.339 | -0.1320 | No |
| 34 | COPB2 |  |  | 2929 | 0.203 | -0.1762 | No |
| 35 | RANBP2 |  |  | 2943 | 0.194 | -0.1769 | No |
| 36 | CTNS |  |  | 2949 | 0.191 | -0.1764 | No |
| 37 | PIGS |  |  | 2968 | 0.182 | -0.1779 | No |
| 38 | BET1 |  |  | 3013 | 0.159 | -0.1833 | No |
| 39 | LMAN1 |  |  | 3100 | 0.114 | -0.1949 | No |
| 40 | IPO7 |  |  | 3101 | 0.114 | -0.1942 | No |
| 41 | COPG |  |  | 3121 | 0.108 | -0.1963 | No |
| 42 | COPB1 |  |  | 3177 | 0.080 | -0.2037 | No |
| 43 | NUTF2 |  |  | 3187 | 0.076 | -0.2045 | No |
| 44 | MPV17 |  |  | 3219 | 0.064 | -0.2086 | No |
| 45 | ERN1 |  |  | 3327 | 0.017 | -0.2239 | No |
| 46 | ENTPD4 |  |  | 3538 | -0.079 | -0.2536 | No |
| 47 | DDX19B |  |  | 3588 | -0.107 | -0.2600 | No |
| 48 | AP1G2 |  |  | 3669 | -0.147 | -0.2706 | No |
| 49 | CUZD1 |  |  | 3735 | -0.174 | -0.2788 | No |
| 50 | NUP133 |  |  | 3750 | -0.180 | -0.2797 | No |
| 51 | CASP7 |  |  | 3857 | -0.242 | -0.2935 | No |
| 52 | MAP1LC3B |  |  | 3862 | -0.245 | -0.2926 | No |
| 53 | COX6B2 |  |  | 3909 | -0.267 | -0.2975 | No |
| 54 | VAPB |  |  | 4069 | -0.342 | -0.3183 | No |
| 55 | ALG3 |  |  | 4072 | -0.343 | -0.3165 | No |
| 56 | LDLRAP1 |  |  | 4076 | -0.344 | -0.3148 | No |
| 57 | SPCS1 |  |  | 4107 | -0.357 | -0.3170 | No |
| 58 | GPAA1 |  |  | 4108 | -0.357 | -0.3148 | No |
| 59 | PSEN1 |  |  | 4159 | -0.382 | -0.3196 | No |
| 60 | SRP9 |  |  | 4162 | -0.384 | -0.3176 | No |
| 61 | KPNA3 |  |  | 4248 | -0.427 | -0.3272 | No |
| 62 | OXA1L |  |  | 4256 | -0.429 | -0.3255 | No |
| 63 | RHOT2 |  |  | 4358 | -0.470 | -0.3372 | No |
| 64 | MATR3 |  |  | 4499 | -0.533 | -0.3540 | No |
| 65 | SLC9A6 |  |  | 4512 | -0.538 | -0.3525 | No |
| 66 | TIMM17A |  |  | 4569 | -0.561 | -0.3571 | No |
| 67 | MRPL19 |  |  | 4689 | -0.611 | -0.3705 | No |
| 68 | PIGK |  |  | 4720 | -0.628 | -0.3709 | No |
| 69 | PXMP4 |  |  | 4842 | -0.688 | -0.3841 | No |
| 70 | NUP50 |  |  | 5085 | -0.802 | -0.4140 | No |
| 71 | DERL3 |  |  | 5086 | -0.803 | -0.4091 | No |
| 72 | DAD1 |  |  | 5122 | -0.818 | -0.4091 | No |
| 73 | UBQLN4 |  |  | 5319 | -0.928 | -0.4316 | No |
| 74 | DPM2 |  |  | 5361 | -0.954 | -0.4316 | Yes |
| 75 | ATP5E |  |  | 5386 | -0.967 | -0.4292 | Yes |
| 76 | ICA1 |  |  | 5396 | -0.974 | -0.4245 | Yes |
| 77 | SRPR |  |  | 5475 | -1.024 | -0.4295 | Yes |
| 78 | RANGAP1 |  |  | 5476 | -1.024 | -0.4232 | Yes |
| 79 | PPOX |  |  | 5513 | -1.046 | -0.4220 | Yes |
| 80 | ABCD3 |  |  | 5545 | -1.062 | -0.4199 | Yes |
| 81 | COPE |  |  | 5593 | -1.086 | -0.4201 | Yes |
| 82 | COG2 |  |  | 5611 | -1.096 | -0.4158 | Yes |
| 83 | COPA |  |  | 5705 | -1.156 | -0.4221 | Yes |
| 84 | AAAS |  |  | 5713 | -1.159 | -0.4160 | Yes |
| 85 | SLC25A11 |  |  | 5729 | -1.168 | -0.4110 | Yes |
| 86 | NDUFA1 |  |  | 5747 | -1.178 | -0.4063 | Yes |
| 87 | KPNA1 |  |  | 5762 | -1.186 | -0.4010 | Yes |
| 88 | SURF1 |  |  | 5786 | -1.195 | -0.3970 | Yes |
| 89 | MAP4K2 |  |  | 5789 | -1.197 | -0.3900 | Yes |
| 90 | TRIM23 |  |  | 5830 | -1.224 | -0.3883 | Yes |
| 91 | ARFGEF2 |  |  | 5920 | -1.276 | -0.3933 | Yes |
| 92 | TIMM8B |  |  | 5951 | -1.296 | -0.3896 | Yes |
| 93 | COX15 |  |  | 5952 | -1.296 | -0.3817 | Yes |
| 94 | PHB |  |  | 5961 | -1.303 | -0.3749 | Yes |
| 95 | UCP3 |  |  | 5978 | -1.313 | -0.3692 | Yes |
| 96 | LMNB1 |  |  | 6039 | -1.357 | -0.3695 | Yes |
| 97 | NDUFA2 |  |  | 6084 | -1.402 | -0.3673 | Yes |
| 98 | DDOST |  |  | 6107 | -1.423 | -0.3617 | Yes |
| 99 | MTX2 |  |  | 6229 | -1.535 | -0.3697 | Yes |
| 100 | TIMM9 |  |  | 6309 | -1.610 | -0.3712 | Yes |
| 101 | DERL1 |  |  | 6310 | -1.610 | -0.3614 | Yes |
| 102 | SELP |  |  | 6316 | -1.613 | -0.3522 | Yes |
| 103 | PEX12 |  |  | 6332 | -1.629 | -0.3444 | Yes |
| 104 | SDHD |  |  | 6419 | -1.723 | -0.3463 | Yes |
| 105 | TIMM10 |  |  | 6492 | -1.809 | -0.3455 | Yes |
| 106 | MFN2 |  |  | 6501 | -1.822 | -0.3356 | Yes |
| 107 | NDUFA9 |  |  | 6519 | -1.848 | -0.3267 | Yes |
| 108 | ORMDL1 |  |  | 6520 | -1.849 | -0.3154 | Yes |
| 109 | UQCRC1 |  |  | 6576 | -1.924 | -0.3115 | Yes |
| 110 | NDUFAB1 |  |  | 6622 | -1.998 | -0.3058 | Yes |
| 111 | BSCL2 |  |  | 6643 | -2.027 | -0.2963 | Yes |
| 112 | MRPL32 |  |  | 6687 | -2.106 | -0.2896 | Yes |
| 113 | RAE1 |  |  | 6715 | -2.163 | -0.2802 | Yes |
| 114 | NDUFA6 |  |  | 6716 | -2.167 | -0.2670 | Yes |
| 115 | PEX19 |  |  | 6727 | -2.183 | -0.2551 | Yes |
| 116 | NUP54 |  |  | 6752 | -2.232 | -0.2449 | Yes |
| 117 | NDUFS2 |  |  | 6777 | -2.265 | -0.2345 | Yes |
| 118 | COPG2 |  |  | 6787 | -2.283 | -0.2218 | Yes |
| 119 | ABCB7 |  |  | 6790 | -2.286 | -0.2081 | Yes |
| 120 | NDUFS4 |  |  | 6792 | -2.295 | -0.1942 | Yes |
| 121 | PMPCA |  |  | 6795 | -2.314 | -0.1804 | Yes |
| 122 | ALAS2 |  |  | 6802 | -2.335 | -0.1670 | Yes |
| 123 | ATP5B |  |  | 6814 | -2.361 | -0.1541 | Yes |
| 124 | PEX14 |  |  | 6856 | -2.478 | -0.1449 | Yes |
| 125 | ABCA2 |  |  | 6866 | -2.534 | -0.1307 | Yes |
| 126 | RTN4 |  |  | 6909 | -2.681 | -0.1203 | Yes |
| 127 | OPA1 |  |  | 6912 | -2.702 | -0.1041 | Yes |
| 128 | TIMM50 |  |  | 6924 | -2.767 | -0.0887 | Yes |
| 129 | POMT1 |  |  | 6984 | -3.149 | -0.0780 | Yes |
| 130 | PEX16 |  |  | 6993 | -3.210 | -0.0595 | Yes |
| 131 | SLC2A4 |  |  | 7041 | -3.730 | -0.0434 | Yes |
| 132 | PEX11A |  |  | 7049 | -3.865 | -0.0208 | Yes |
| 133 | NDUFS1 |  |  | 7068 | -4.370 | 0.0033 | Yes |
Table: GSEA details [plain text format]

  

Fig 2: ORGANELLE\_MEMBRANE: Random ES distribution      
 Gene set null distribution of ES for **ORGANELLE\_MEMBRANE**
