# Supplementary material for: Network-based integration of molecular and physiological data elucidates regulatory mechanisms underlying adaptation to high-fat diet
Source: Genes Nutr. 2015 May 28;10(4):22. doi: 10.1007/s12263-015-0470-6 (PMC4446272; doi:10.1007/s12263-015-0470-6)
Supplement: Supplementary file 4 — Supplementary material 4 (ZIP 6984 kb) [file 12263_2015_470_MOESM4_ESM.zip › HF LF 5 d GSEA result/OXIDOREDUCTASE_ACTIVITY.html]

Details for gene set OXIDOREDUCTASE\_ACTIVITY[GSEA]

|  || Dataset | comp\_HF5d-LF5d\_collapsed |
| Phenotype | NoPhenotypeAvailable |
| Upregulated in class | na\_neg |
| GeneSet | OXIDOREDUCTASE\_ACTIVITY |
| Enrichment Score (ES) | -0.4940274 |
| Normalized Enrichment Score (NES) | -2.1480258 |
| Nominal p-value | 0.0 |
| FDR q-value | 4.7289443E-4 |
| FWER p-Value | 0.008 |
Table: GSEA Results Summary

  

Fig 1: Enrichment plot: OXIDOREDUCTASE\_ACTIVITY      
 Profile of the Running ES Score & Positions of GeneSet Members on the Rank Ordered List

  

| PROBE | GENE SYMBOL | GENE\_TITLE | RANK IN GENE LIST | RANK METRIC SCORE | RUNNING ES | CORE ENRICHMENT || 1 | P4HA2 |  |  | 7 | 4.223 | 0.0234 | No |
| 2 | CYBA |  |  | 103 | 2.556 | 0.0246 | No |
| 3 | ALOX5 |  |  | 157 | 2.349 | 0.0306 | No |
| 4 | MTHFR |  |  | 405 | 1.866 | 0.0060 | No |
| 5 | ASPH |  |  | 441 | 1.799 | 0.0114 | No |
| 6 | LOXL2 |  |  | 475 | 1.742 | 0.0168 | No |
| 7 | FMO2 |  |  | 501 | 1.707 | 0.0231 | No |
| 8 | TXNRD1 |  |  | 573 | 1.623 | 0.0223 | No |
| 9 | HMOX1 |  |  | 653 | 1.538 | 0.0199 | No |
| 10 | PLOD2 |  |  | 656 | 1.533 | 0.0284 | No |
| 11 | ALDH1A3 |  |  | 770 | 1.434 | 0.0205 | No |
| 12 | ALDH1A2 |  |  | 822 | 1.377 | 0.0212 | No |
| 13 | ABP1 |  |  | 907 | 1.303 | 0.0167 | No |
| 14 | CYP7B1 |  |  | 988 | 1.239 | 0.0124 | No |
| 15 | HPGD |  |  | 1013 | 1.222 | 0.0161 | No |
| 16 | BBOX1 |  |  | 1047 | 1.200 | 0.0183 | No |
| 17 | PTGS1 |  |  | 1054 | 1.195 | 0.0243 | No |
| 18 | ALDH1A1 |  |  | 1060 | 1.189 | 0.0305 | No |
| 19 | ALOX12 |  |  | 1117 | 1.138 | 0.0291 | No |
| 20 | AOC3 |  |  | 1640 | 0.835 | -0.0409 | No |
| 21 | PRODH |  |  | 1751 | 0.779 | -0.0522 | No |
| 22 | CBR3 |  |  | 2013 | 0.627 | -0.0859 | No |
| 23 | CYP1B1 |  |  | 2355 | 0.462 | -0.1321 | No |
| 24 | GSTZ1 |  |  | 2518 | 0.387 | -0.1531 | No |
| 25 | MAOB |  |  | 2556 | 0.367 | -0.1562 | No |
| 26 | AOX1 |  |  | 2693 | 0.304 | -0.1740 | No |
| 27 | DEGS1 |  |  | 2735 | 0.283 | -0.1782 | No |
| 28 | SUOX |  |  | 2842 | 0.239 | -0.1920 | No |
| 29 | BLVRA |  |  | 3150 | 0.093 | -0.2355 | No |
| 30 | HMOX2 |  |  | 3518 | -0.073 | -0.2876 | No |
| 31 | VCL |  |  | 3525 | -0.075 | -0.2880 | No |
| 32 | NQO1 |  |  | 3537 | -0.079 | -0.2892 | No |
| 33 | PLOD3 |  |  | 3564 | -0.092 | -0.2924 | No |
| 34 | CPOX |  |  | 3666 | -0.145 | -0.3060 | No |
| 35 | P4HB |  |  | 3736 | -0.174 | -0.3149 | No |
| 36 | ME2 |  |  | 3778 | -0.199 | -0.3196 | No |
| 37 | XDH |  |  | 3849 | -0.238 | -0.3282 | No |
| 38 | IVD |  |  | 3933 | -0.276 | -0.3385 | No |
| 39 | ALOX15 |  |  | 4011 | -0.312 | -0.3478 | No |
| 40 | PHYH |  |  | 4017 | -0.315 | -0.3467 | No |
| 41 | CYP46A1 |  |  | 4145 | -0.377 | -0.3627 | No |
| 42 | CYP27A1 |  |  | 4208 | -0.409 | -0.3692 | No |
| 43 | FDXR |  |  | 4308 | -0.451 | -0.3808 | No |
| 44 | MSRB3 |  |  | 4378 | -0.478 | -0.3879 | No |
| 45 | APEX1 |  |  | 4413 | -0.497 | -0.3899 | No |
| 46 | WWOX |  |  | 4442 | -0.508 | -0.3910 | No |
| 47 | ACOX3 |  |  | 4494 | -0.530 | -0.3952 | No |
| 48 | COX7A1 |  |  | 4542 | -0.552 | -0.3987 | No |
| 49 | MTHFD2 |  |  | 4595 | -0.571 | -0.4029 | No |
| 50 | ADH4 |  |  | 4666 | -0.601 | -0.4094 | No |
| 51 | ALOX12B |  |  | 4817 | -0.673 | -0.4270 | No |
| 52 | MGST3 |  |  | 4962 | -0.747 | -0.4433 | No |
| 53 | LDHC |  |  | 5040 | -0.781 | -0.4499 | No |
| 54 | DIO2 |  |  | 5048 | -0.785 | -0.4463 | No |
| 55 | HSD17B4 |  |  | 5280 | -0.905 | -0.4742 | No |
| 56 | CYP17A1 |  |  | 5311 | -0.923 | -0.4731 | No |
| 57 | ALDH5A1 |  |  | 5315 | -0.925 | -0.4682 | No |
| 58 | PDIA5 |  |  | 5316 | -0.925 | -0.4629 | No |
| 59 | IDH1 |  |  | 5317 | -0.927 | -0.4575 | No |
| 60 | RDH10 |  |  | 5416 | -0.985 | -0.4658 | No |
| 61 | PPOX |  |  | 5513 | -1.046 | -0.4735 | No |
| 62 | MSRB2 |  |  | 5525 | -1.050 | -0.4690 | No |
| 63 | SPR |  |  | 5585 | -1.082 | -0.4712 | No |
| 64 | COX10 |  |  | 5667 | -1.133 | -0.4763 | No |
| 65 | TSTA3 |  |  | 5736 | -1.172 | -0.4792 | No |
| 66 | GPX4 |  |  | 5750 | -1.178 | -0.4743 | No |
| 67 | SURF1 |  |  | 5786 | -1.195 | -0.4724 | No |
| 68 | GPX3 |  |  | 5836 | -1.226 | -0.4723 | No |
| 69 | COX15 |  |  | 5952 | -1.296 | -0.4813 | No |
| 70 | SOD2 |  |  | 6042 | -1.359 | -0.4862 | Yes |
| 71 | TXNL1 |  |  | 6046 | -1.361 | -0.4787 | Yes |
| 72 | DHCR24 |  |  | 6066 | -1.383 | -0.4734 | Yes |
| 73 | ALDH8A1 |  |  | 6080 | -1.399 | -0.4672 | Yes |
| 74 | IMPDH1 |  |  | 6094 | -1.411 | -0.4609 | Yes |
| 75 | ACADM |  |  | 6098 | -1.415 | -0.4531 | Yes |
| 76 | NOS3 |  |  | 6134 | -1.447 | -0.4498 | Yes |
| 77 | ACADSB |  |  | 6412 | -1.713 | -0.4796 | Yes |
| 78 | SDHD |  |  | 6419 | -1.723 | -0.4705 | Yes |
| 79 | EGLN1 |  |  | 6483 | -1.803 | -0.4690 | Yes |
| 80 | DECR1 |  |  | 6507 | -1.831 | -0.4618 | Yes |
| 81 | NDUFA9 |  |  | 6519 | -1.848 | -0.4526 | Yes |
| 82 | IDH3G |  |  | 6535 | -1.872 | -0.4440 | Yes |
| 83 | COX11 |  |  | 6569 | -1.914 | -0.4376 | Yes |
| 84 | UQCRC1 |  |  | 6576 | -1.924 | -0.4273 | Yes |
| 85 | HSD17B7 |  |  | 6625 | -2.001 | -0.4226 | Yes |
| 86 | RDH11 |  |  | 6665 | -2.070 | -0.4162 | Yes |
| 87 | ACADL |  |  | 6689 | -2.107 | -0.4074 | Yes |
| 88 | HADHA |  |  | 6704 | -2.142 | -0.3970 | Yes |
| 89 | ALDH6A1 |  |  | 6728 | -2.184 | -0.3876 | Yes |
| 90 | EHHADH |  |  | 6736 | -2.204 | -0.3759 | Yes |
| 91 | NDUFS2 |  |  | 6777 | -2.265 | -0.3685 | Yes |
| 92 | NDUFS4 |  |  | 6792 | -2.295 | -0.3572 | Yes |
| 93 | BCKDHA |  |  | 6809 | -2.351 | -0.3459 | Yes |
| 94 | COX7B |  |  | 6833 | -2.401 | -0.3353 | Yes |
| 95 | CYCS |  |  | 6844 | -2.437 | -0.3226 | Yes |
| 96 | COX4I1 |  |  | 6847 | -2.443 | -0.3088 | Yes |
| 97 | ALDH2 |  |  | 6865 | -2.528 | -0.2966 | Yes |
| 98 | SC5DL |  |  | 6873 | -2.562 | -0.2828 | Yes |
| 99 | ALDH9A1 |  |  | 6903 | -2.662 | -0.2715 | Yes |
| 100 | MDH1 |  |  | 6929 | -2.798 | -0.2589 | Yes |
| 101 | IDH3B |  |  | 6930 | -2.804 | -0.2427 | Yes |
| 102 | FMO1 |  |  | 6968 | -2.997 | -0.2307 | Yes |
| 103 | CYC1 |  |  | 6975 | -3.040 | -0.2139 | Yes |
| 104 | DLD |  |  | 6996 | -3.249 | -0.1980 | Yes |
| 105 | CDO1 |  |  | 6998 | -3.263 | -0.1793 | Yes |
| 106 | FADS1 |  |  | 7018 | -3.490 | -0.1618 | Yes |
| 107 | COX5A |  |  | 7038 | -3.698 | -0.1431 | Yes |
| 108 | ALDH4A1 |  |  | 7056 | -4.141 | -0.1216 | Yes |
| 109 | NDUFS1 |  |  | 7068 | -4.370 | -0.0979 | Yes |
| 110 | PRDX3 |  |  | 7073 | -4.553 | -0.0721 | Yes |
| 111 | GPD1 |  |  | 7074 | -4.564 | -0.0457 | Yes |
| 112 | GPD2 |  |  | 7088 | -8.295 | 0.0004 | Yes |
Table: GSEA details [plain text format]

  

Fig 2: OXIDOREDUCTASE\_ACTIVITY: Random ES distribution      
 Gene set null distribution of ES for **OXIDOREDUCTASE\_ACTIVITY**
