# Supplementary material for: Network-based integration of molecular and physiological data elucidates regulatory mechanisms underlying adaptation to high-fat diet
Source: Genes Nutr. 2015 May 28;10(4):22. doi: 10.1007/s12263-015-0470-6 (PMC4446272; doi:10.1007/s12263-015-0470-6)
Supplement: Supplementary file 4 — Supplementary material 4 (ZIP 6984 kb) [file 12263_2015_470_MOESM4_ESM.zip › HF LF 5 d GSEA result/OXIDOREDUCTASE_ACTIVITY_GO_0016616.html]

Details for gene set OXIDOREDUCTASE\_ACTIVITY\_GO\_0016616[GSEA]

|  || Dataset | comp\_HF5d-LF5d\_collapsed |
| Phenotype | NoPhenotypeAvailable |
| Upregulated in class | na\_neg |
| GeneSet | OXIDOREDUCTASE\_ACTIVITY\_GO\_0016616 |
| Enrichment Score (ES) | -0.62130487 |
| Normalized Enrichment Score (NES) | -1.7942958 |
| Nominal p-value | 0.004901961 |
| FDR q-value | 0.024594583 |
| FWER p-Value | 0.518 |
Table: GSEA Results Summary

  

Fig 1: Enrichment plot: OXIDOREDUCTASE\_ACTIVITY\_GO\_0016616      
 Profile of the Running ES Score & Positions of GeneSet Members on the Rank Ordered List

  

| PROBE | GENE SYMBOL | GENE\_TITLE | RANK IN GENE LIST | RANK METRIC SCORE | RUNNING ES | CORE ENRICHMENT || 1 | HPGD |  |  | 1013 | 1.222 | -0.0989 | No |
| 2 | CBR3 |  |  | 2013 | 0.627 | -0.2174 | No |
| 3 | ME2 |  |  | 3778 | -0.199 | -0.4595 | No |
| 4 | ADH4 |  |  | 4666 | -0.601 | -0.5631 | No |
| 5 | LDHC |  |  | 5040 | -0.781 | -0.5875 | No |
| 6 | HSD17B4 |  |  | 5280 | -0.905 | -0.5885 | Yes |
| 7 | IDH1 |  |  | 5317 | -0.927 | -0.5599 | Yes |
| 8 | RDH10 |  |  | 5416 | -0.985 | -0.5381 | Yes |
| 9 | SPR |  |  | 5585 | -1.082 | -0.5225 | Yes |
| 10 | TSTA3 |  |  | 5736 | -1.172 | -0.5012 | Yes |
| 11 | IMPDH1 |  |  | 6094 | -1.411 | -0.5005 | Yes |
| 12 | IDH3G |  |  | 6535 | -1.872 | -0.4948 | Yes |
| 13 | HSD17B7 |  |  | 6625 | -2.001 | -0.4348 | Yes |
| 14 | RDH11 |  |  | 6665 | -2.070 | -0.3653 | Yes |
| 15 | HADHA |  |  | 6704 | -2.142 | -0.2929 | Yes |
| 16 | EHHADH |  |  | 6736 | -2.204 | -0.2174 | Yes |
| 17 | IDH3B |  |  | 6930 | -2.804 | -0.1429 | Yes |
| 18 | GPD1 |  |  | 7074 | -4.564 | 0.0024 | Yes |
Table: GSEA details [plain text format]

  

Fig 2: OXIDOREDUCTASE\_ACTIVITY\_GO\_0016616: Random ES distribution      
 Gene set null distribution of ES for **OXIDOREDUCTASE\_ACTIVITY\_GO\_0016616**
