# Supplementary material for: Network-based integration of molecular and physiological data elucidates regulatory mechanisms underlying adaptation to high-fat diet
Source: Genes Nutr. 2015 May 28;10(4):22. doi: 10.1007/s12263-015-0470-6 (PMC4446272; doi:10.1007/s12263-015-0470-6)
Supplement: Supplementary file 4 — Supplementary material 4 (ZIP 6984 kb) [file 12263_2015_470_MOESM4_ESM.zip › HF LF 5 d GSEA result/PEROXISOME.html]

Details for gene set PEROXISOME[GSEA]

|  || Dataset | comp\_HF5d-LF5d\_collapsed |
| Phenotype | NoPhenotypeAvailable |
| Upregulated in class | na\_neg |
| GeneSet | PEROXISOME |
| Enrichment Score (ES) | -0.6477017 |
| Normalized Enrichment Score (NES) | -2.0765562 |
| Nominal p-value | 0.0 |
| FDR q-value | 0.0011167685 |
| FWER p-Value | 0.022 |
Table: GSEA Results Summary

  

Fig 1: Enrichment plot: PEROXISOME      
 Profile of the Running ES Score & Positions of GeneSet Members on the Rank Ordered List

  

| PROBE | GENE SYMBOL | GENE\_TITLE | RANK IN GENE LIST | RANK METRIC SCORE | RUNNING ES | CORE ENRICHMENT || 1 | ECH1 |  |  | 1361 | 0.990 | -0.1670 | No |
| 2 | PIPOX |  |  | 1554 | 0.877 | -0.1715 | No |
| 3 | ABCD2 |  |  | 2907 | 0.213 | -0.3573 | No |
| 4 | SLC25A17 |  |  | 3807 | -0.214 | -0.4790 | No |
| 5 | PHYH |  |  | 4017 | -0.315 | -0.5004 | No |
| 6 | CROT |  |  | 4088 | -0.350 | -0.5013 | No |
| 7 | ACOX3 |  |  | 4494 | -0.530 | -0.5449 | No |
| 8 | GNPAT |  |  | 4655 | -0.596 | -0.5521 | No |
| 9 | PXMP4 |  |  | 4842 | -0.688 | -0.5606 | No |
| 10 | ISOC1 |  |  | 4914 | -0.722 | -0.5520 | No |
| 11 | MLYCD |  |  | 5042 | -0.781 | -0.5498 | No |
| 12 | HSD17B4 |  |  | 5280 | -0.905 | -0.5599 | No |
| 13 | ABCD3 |  |  | 5545 | -1.062 | -0.5698 | No |
| 14 | PEX6 |  |  | 5931 | -1.283 | -0.5911 | No |
| 15 | PEX12 |  |  | 6332 | -1.629 | -0.6056 | Yes |
| 16 | SCP2 |  |  | 6376 | -1.673 | -0.5684 | Yes |
| 17 | PEX7 |  |  | 6429 | -1.740 | -0.5308 | Yes |
| 18 | AMACR |  |  | 6543 | -1.877 | -0.4982 | Yes |
| 19 | PEX19 |  |  | 6727 | -2.183 | -0.4677 | Yes |
| 20 | EHHADH |  |  | 6736 | -2.204 | -0.4118 | Yes |
| 21 | PEX14 |  |  | 6856 | -2.478 | -0.3646 | Yes |
| 22 | PEX16 |  |  | 6993 | -3.210 | -0.3009 | Yes |
| 23 | IDE |  |  | 7042 | -3.748 | -0.2108 | Yes |
| 24 | PEX11A |  |  | 7049 | -3.865 | -0.1117 | Yes |
| 25 | PMVK |  |  | 7072 | -4.545 | 0.0027 | Yes |
Table: GSEA details [plain text format]

  

Fig 2: PEROXISOME: Random ES distribution      
 Gene set null distribution of ES for **PEROXISOME**
