# Supplementary material for: Network-based integration of molecular and physiological data elucidates regulatory mechanisms underlying adaptation to high-fat diet
Source: Genes Nutr. 2015 May 28;10(4):22. doi: 10.1007/s12263-015-0470-6 (PMC4446272; doi:10.1007/s12263-015-0470-6)
Supplement: Supplementary file 4 — Supplementary material 4 (ZIP 6984 kb) [file 12263_2015_470_MOESM4_ESM.zip › HF LF 5 d GSEA result/PROTEIN_KINASE_CASCADE.html]

Details for gene set PROTEIN\_KINASE\_CASCADE[GSEA]

|  || Dataset | comp\_HF5d-LF5d\_collapsed |
| Phenotype | NoPhenotypeAvailable |
| Upregulated in class | na\_pos |
| GeneSet | PROTEIN\_KINASE\_CASCADE |
| Enrichment Score (ES) | 0.40178692 |
| Normalized Enrichment Score (NES) | 1.9145472 |
| Nominal p-value | 0.0 |
| FDR q-value | 0.018892115 |
| FWER p-Value | 0.191 |
Table: GSEA Results Summary

  

Fig 1: Enrichment plot: PROTEIN\_KINASE\_CASCADE      
 Profile of the Running ES Score & Positions of GeneSet Members on the Rank Ordered List

  

| PROBE | GENE SYMBOL | GENE\_TITLE | RANK IN GENE LIST | RANK METRIC SCORE | RUNNING ES | CORE ENRICHMENT || 1 | RHOC |  |  | 71 | 2.717 | 0.0136 | Yes |
| 2 | DAXX |  |  | 116 | 2.503 | 0.0292 | Yes |
| 3 | DUSP10 |  |  | 133 | 2.425 | 0.0481 | Yes |
| 4 | LATS2 |  |  | 182 | 2.274 | 0.0611 | Yes |
| 5 | PLCE1 |  |  | 195 | 2.243 | 0.0790 | Yes |
| 6 | CCR2 |  |  | 219 | 2.188 | 0.0948 | Yes |
| 7 | MDFIC |  |  | 226 | 2.178 | 0.1130 | Yes |
| 8 | NEK6 |  |  | 264 | 2.102 | 0.1260 | Yes |
| 9 | MAP4K1 |  |  | 270 | 2.093 | 0.1436 | Yes |
| 10 | DUSP2 |  |  | 278 | 2.072 | 0.1607 | Yes |
| 11 | MAP3K6 |  |  | 281 | 2.067 | 0.1785 | Yes |
| 12 | ECM1 |  |  | 288 | 2.049 | 0.1956 | Yes |
| 13 | DUSP6 |  |  | 347 | 1.958 | 0.2044 | Yes |
| 14 | SOCS3 |  |  | 369 | 1.925 | 0.2182 | Yes |
| 15 | SOCS1 |  |  | 450 | 1.783 | 0.2223 | Yes |
| 16 | AKAP11 |  |  | 470 | 1.759 | 0.2350 | Yes |
| 17 | ATP6AP2 |  |  | 598 | 1.588 | 0.2307 | Yes |
| 18 | GADD45G |  |  | 608 | 1.582 | 0.2432 | Yes |
| 19 | PLK2 |  |  | 625 | 1.560 | 0.2546 | Yes |
| 20 | HMOX1 |  |  | 653 | 1.538 | 0.2641 | Yes |
| 21 | TLR6 |  |  | 679 | 1.511 | 0.2738 | Yes |
| 22 | CFLAR |  |  | 680 | 1.511 | 0.2870 | Yes |
| 23 | STK4 |  |  | 696 | 1.500 | 0.2979 | Yes |
| 24 | CD24 |  |  | 804 | 1.398 | 0.2948 | Yes |
| 25 | STK17B |  |  | 849 | 1.354 | 0.3004 | Yes |
| 26 | CCL2 |  |  | 875 | 1.330 | 0.3084 | Yes |
| 27 | PIAS1 |  |  | 902 | 1.306 | 0.3161 | Yes |
| 28 | MAP3K13 |  |  | 921 | 1.288 | 0.3248 | Yes |
| 29 | RPS6KA4 |  |  | 923 | 1.287 | 0.3359 | Yes |
| 30 | PTEN |  |  | 967 | 1.258 | 0.3407 | Yes |
| 31 | TNFRSF1A |  |  | 973 | 1.253 | 0.3510 | Yes |
| 32 | DUSP9 |  |  | 1112 | 1.144 | 0.3412 | Yes |
| 33 | HCLS1 |  |  | 1144 | 1.127 | 0.3466 | Yes |
| 34 | TGFA |  |  | 1152 | 1.124 | 0.3554 | Yes |
| 35 | SECTM1 |  |  | 1174 | 1.111 | 0.3621 | Yes |
| 36 | TRADD |  |  | 1228 | 1.080 | 0.3640 | Yes |
| 37 | CD81 |  |  | 1230 | 1.078 | 0.3732 | Yes |
| 38 | PAK1 |  |  | 1237 | 1.074 | 0.3818 | Yes |
| 39 | RIPK1 |  |  | 1239 | 1.073 | 0.3910 | Yes |
| 40 | STAT3 |  |  | 1269 | 1.057 | 0.3961 | Yes |
| 41 | PPP5C |  |  | 1309 | 1.017 | 0.3994 | Yes |
| 42 | LTBR |  |  | 1354 | 0.994 | 0.4018 | Yes |
| 43 | BST2 |  |  | 1485 | 0.912 | 0.3911 | No |
| 44 | WNK1 |  |  | 1512 | 0.898 | 0.3953 | No |
| 45 | BIRC2 |  |  | 1573 | 0.868 | 0.3943 | No |
| 46 | MBIP |  |  | 1617 | 0.847 | 0.3955 | No |
| 47 | BCL10 |  |  | 1824 | 0.734 | 0.3724 | No |
| 48 | LGALS1 |  |  | 1924 | 0.679 | 0.3642 | No |
| 49 | WNK4 |  |  | 2014 | 0.626 | 0.3569 | No |
| 50 | CXCR4 |  |  | 2015 | 0.626 | 0.3624 | No |
| 51 | AMBP |  |  | 2026 | 0.619 | 0.3664 | No |
| 52 | TPD52L1 |  |  | 2142 | 0.558 | 0.3548 | No |
| 53 | VAPA |  |  | 2264 | 0.500 | 0.3418 | No |
| 54 | GJA1 |  |  | 2268 | 0.499 | 0.3458 | No |
| 55 | STK38 |  |  | 2402 | 0.441 | 0.3306 | No |
| 56 | DAPK2 |  |  | 2420 | 0.433 | 0.3319 | No |
| 57 | SOCS2 |  |  | 2487 | 0.404 | 0.3260 | No |
| 58 | STAMBP |  |  | 2529 | 0.381 | 0.3235 | No |
| 59 | MAPK13 |  |  | 2567 | 0.360 | 0.3213 | No |
| 60 | ITGB1BP1 |  |  | 2593 | 0.347 | 0.3208 | No |
| 61 | SRPK1 |  |  | 2644 | 0.325 | 0.3164 | No |
| 62 | TRAF3IP2 |  |  | 2730 | 0.285 | 0.3068 | No |
| 63 | TBK1 |  |  | 2914 | 0.209 | 0.2824 | No |
| 64 | TSPAN6 |  |  | 3021 | 0.155 | 0.2686 | No |
| 65 | STAT4 |  |  | 3097 | 0.116 | 0.2588 | No |
| 66 | F2R |  |  | 3144 | 0.095 | 0.2531 | No |
| 67 | GADD45B |  |  | 3155 | 0.090 | 0.2524 | No |
| 68 | IKBKE |  |  | 3276 | 0.037 | 0.2356 | No |
| 69 | MAP3K11 |  |  | 3461 | -0.039 | 0.2096 | No |
| 70 | ZDHHC17 |  |  | 3611 | -0.117 | 0.1893 | No |
| 71 | CRKL |  |  | 3650 | -0.136 | 0.1850 | No |
| 72 | MAP3K3 |  |  | 3725 | -0.170 | 0.1759 | No |
| 73 | TRAF6 |  |  | 3749 | -0.180 | 0.1742 | No |
| 74 | CARD10 |  |  | 3813 | -0.217 | 0.1670 | No |
| 75 | OXSR1 |  |  | 3854 | -0.240 | 0.1634 | No |
| 76 | IFNAR2 |  |  | 3964 | -0.291 | 0.1503 | No |
| 77 | CANT1 |  |  | 4029 | -0.320 | 0.1440 | No |
| 78 | CXXC5 |  |  | 4035 | -0.321 | 0.1461 | No |
| 79 | LITAF |  |  | 4057 | -0.333 | 0.1460 | No |
| 80 | IKBKG |  |  | 4303 | -0.449 | 0.1148 | No |
| 81 | TRIP6 |  |  | 4306 | -0.450 | 0.1185 | No |
| 82 | EGF |  |  | 4373 | -0.476 | 0.1132 | No |
| 83 | EPGN |  |  | 4398 | -0.488 | 0.1140 | No |
| 84 | DBNL |  |  | 4486 | -0.528 | 0.1062 | No |
| 85 | MAP3K5 |  |  | 4634 | -0.587 | 0.0902 | No |
| 86 | ICK |  |  | 4656 | -0.596 | 0.0924 | No |
| 87 | FGF2 |  |  | 4723 | -0.629 | 0.0885 | No |
| 88 | CASP8 |  |  | 4731 | -0.633 | 0.0930 | No |
| 89 | MAP3K7 |  |  | 4858 | -0.695 | 0.0811 | No |
| 90 | GOLT1B |  |  | 5018 | -0.768 | 0.0650 | No |
| 91 | UBE2V1 |  |  | 5060 | -0.789 | 0.0660 | No |
| 92 | MAPKAPK2 |  |  | 5061 | -0.790 | 0.0729 | No |
| 93 | GNG3 |  |  | 5227 | -0.876 | 0.0570 | No |
| 94 | RHOA |  |  | 5295 | -0.914 | 0.0554 | No |
| 95 | MYD88 |  |  | 5367 | -0.958 | 0.0536 | No |
| 96 | PIK3CB |  |  | 5683 | -1.143 | 0.0185 | No |
| 97 | FADD |  |  | 5764 | -1.187 | 0.0174 | No |
| 98 | MAP4K2 |  |  | 5789 | -1.197 | 0.0244 | No |
| 99 | MAPK9 |  |  | 5794 | -1.200 | 0.0343 | No |
| 100 | IL31RA |  |  | 6036 | -1.355 | 0.0117 | No |
| 101 | DAPK3 |  |  | 6102 | -1.420 | 0.0148 | No |
| 102 | IRAK1 |  |  | 6121 | -1.431 | 0.0247 | No |
| 103 | MAPK11 |  |  | 6231 | -1.538 | 0.0225 | No |
| 104 | UBE2N |  |  | 6548 | -1.884 | -0.0063 | No |
| 105 | MKNK2 |  |  | 6599 | -1.971 | 0.0038 | No |
| 106 | TMED4 |  |  | 6613 | -1.989 | 0.0193 | No |
| 107 | DUSP4 |  |  | 6927 | -2.780 | -0.0012 | No |
| 108 | FAF1 |  |  | 6932 | -2.805 | 0.0228 | No |
Table: GSEA details [plain text format]

  

Fig 2: PROTEIN\_KINASE\_CASCADE: Random ES distribution      
 Gene set null distribution of ES for **PROTEIN\_KINASE\_CASCADE**
