# Supplementary material for: Network-based integration of molecular and physiological data elucidates regulatory mechanisms underlying adaptation to high-fat diet
Source: Genes Nutr. 2015 May 28;10(4):22. doi: 10.1007/s12263-015-0470-6 (PMC4446272; doi:10.1007/s12263-015-0470-6)
Supplement: Supplementary file 4 — Supplementary material 4 (ZIP 6984 kb) [file 12263_2015_470_MOESM4_ESM.zip › HF LF 5 d GSEA result/PROTEINACEOUS_EXTRACELLULAR_MATRIX.html]

Details for gene set PROTEINACEOUS\_EXTRACELLULAR\_MATRIX[GSEA]

|  || Dataset | comp\_HF5d-LF5d\_collapsed |
| Phenotype | NoPhenotypeAvailable |
| Upregulated in class | na\_pos |
| GeneSet | PROTEINACEOUS\_EXTRACELLULAR\_MATRIX |
| Enrichment Score (ES) | 0.6204753 |
| Normalized Enrichment Score (NES) | 2.2652273 |
| Nominal p-value | 0.0 |
| FDR q-value | 0.0016278437 |
| FWER p-Value | 0.002 |
Table: GSEA Results Summary

  

Fig 1: Enrichment plot: PROTEINACEOUS\_EXTRACELLULAR\_MATRIX      
 Profile of the Running ES Score & Positions of GeneSet Members on the Rank Ordered List

  

| PROBE | GENE SYMBOL | GENE\_TITLE | RANK IN GENE LIST | RANK METRIC SCORE | RUNNING ES | CORE ENRICHMENT || 1 | EFEMP2 |  |  | 6 | 4.373 | 0.0922 | Yes |
| 2 | CD248 |  |  | 15 | 3.586 | 0.1674 | Yes |
| 3 | COL1A2 |  |  | 52 | 2.854 | 0.2230 | Yes |
| 4 | DST |  |  | 58 | 2.797 | 0.2818 | Yes |
| 5 | COL6A3 |  |  | 100 | 2.565 | 0.3306 | Yes |
| 6 | LUM |  |  | 208 | 2.217 | 0.3626 | Yes |
| 7 | COL3A1 |  |  | 221 | 2.186 | 0.4075 | Yes |
| 8 | ECM1 |  |  | 288 | 2.049 | 0.4417 | Yes |
| 9 | SGCD |  |  | 524 | 1.686 | 0.4443 | Yes |
| 10 | FBN1 |  |  | 540 | 1.661 | 0.4775 | Yes |
| 11 | COL5A1 |  |  | 566 | 1.630 | 0.5087 | Yes |
| 12 | FBLN1 |  |  | 587 | 1.604 | 0.5400 | Yes |
| 13 | FBLN2 |  |  | 614 | 1.574 | 0.5698 | Yes |
| 14 | FMOD |  |  | 841 | 1.362 | 0.5667 | Yes |
| 15 | SNTB2 |  |  | 873 | 1.333 | 0.5907 | Yes |
| 16 | LAMB2 |  |  | 984 | 1.243 | 0.6016 | Yes |
| 17 | CTGF |  |  | 1033 | 1.207 | 0.6205 | Yes |
| 18 | MGP |  |  | 1234 | 1.077 | 0.6151 | No |
| 19 | COL8A1 |  |  | 1546 | 0.881 | 0.5897 | No |
| 20 | COL4A3 |  |  | 1585 | 0.862 | 0.6027 | No |
| 21 | COMP |  |  | 2208 | 0.527 | 0.5258 | No |
| 22 | DMD |  |  | 2317 | 0.479 | 0.5207 | No |
| 23 | TINAG |  |  | 2691 | 0.305 | 0.4743 | No |
| 24 | LTBP4 |  |  | 2951 | 0.190 | 0.4417 | No |
| 25 | COL4A2 |  |  | 3247 | 0.051 | 0.4010 | No |
| 26 | CHAD |  |  | 3254 | 0.047 | 0.4011 | No |
| 27 | NID2 |  |  | 3369 | -0.007 | 0.3851 | No |
| 28 | LAMA4 |  |  | 4050 | -0.329 | 0.2958 | No |
| 29 | COL15A1 |  |  | 4545 | -0.552 | 0.2375 | No |
| 30 | ADAMTS9 |  |  | 4985 | -0.756 | 0.1914 | No |
| 31 | TGFB1 |  |  | 5288 | -0.910 | 0.1680 | No |
| 32 | COL5A3 |  |  | 5473 | -1.023 | 0.1637 | No |
| 33 | SNTB1 |  |  | 5613 | -1.097 | 0.1673 | No |
| 34 | MAGEE1 |  |  | 6598 | -1.971 | 0.0698 | No |
Table: GSEA details [plain text format]

  

Fig 2: PROTEINACEOUS\_EXTRACELLULAR\_MATRIX: Random ES distribution      
 Gene set null distribution of ES for **PROTEINACEOUS\_EXTRACELLULAR\_MATRIX**
