# Supplementary material for: Network-based integration of molecular and physiological data elucidates regulatory mechanisms underlying adaptation to high-fat diet
Source: Genes Nutr. 2015 May 28;10(4):22. doi: 10.1007/s12263-015-0470-6 (PMC4446272; doi:10.1007/s12263-015-0470-6)
Supplement: Supplementary file 4 — Supplementary material 4 (ZIP 6984 kb) [file 12263_2015_470_MOESM4_ESM.zip › HF LF 5 d GSEA result/REGULATION_OF_MAP_KINASE_ACTIVITY.html]

Details for gene set REGULATION\_OF\_MAP\_KINASE\_ACTIVITY[GSEA]

|  || Dataset | comp\_HF5d-LF5d\_collapsed |
| Phenotype | NoPhenotypeAvailable |
| Upregulated in class | na\_pos |
| GeneSet | REGULATION\_OF\_MAP\_KINASE\_ACTIVITY |
| Enrichment Score (ES) | 0.5973757 |
| Normalized Enrichment Score (NES) | 2.1281688 |
| Nominal p-value | 0.0 |
| FDR q-value | 0.0039343033 |
| FWER p-Value | 0.017 |
Table: GSEA Results Summary

  

Fig 1: Enrichment plot: REGULATION\_OF\_MAP\_KINASE\_ACTIVITY      
 Profile of the Running ES Score & Positions of GeneSet Members on the Rank Ordered List

  

| PROBE | GENE SYMBOL | GENE\_TITLE | RANK IN GENE LIST | RANK METRIC SCORE | RUNNING ES | CORE ENRICHMENT || 1 | DAXX |  |  | 116 | 2.503 | 0.0573 | Yes |
| 2 | PLCE1 |  |  | 195 | 2.243 | 0.1122 | Yes |
| 3 | EGFR |  |  | 222 | 2.185 | 0.1729 | Yes |
| 4 | MDFIC |  |  | 226 | 2.178 | 0.2366 | Yes |
| 5 | MAP4K1 |  |  | 270 | 2.093 | 0.2921 | Yes |
| 6 | DUSP2 |  |  | 278 | 2.072 | 0.3521 | Yes |
| 7 | MAP3K6 |  |  | 281 | 2.067 | 0.4126 | Yes |
| 8 | DUSP6 |  |  | 347 | 1.958 | 0.4611 | Yes |
| 9 | TRIB1 |  |  | 639 | 1.549 | 0.4655 | Yes |
| 10 | TLR6 |  |  | 679 | 1.511 | 0.5044 | Yes |
| 11 | CD24 |  |  | 804 | 1.398 | 0.5280 | Yes |
| 12 | DUSP9 |  |  | 1112 | 1.144 | 0.5182 | Yes |
| 13 | TGFA |  |  | 1152 | 1.124 | 0.5458 | Yes |
| 14 | CD81 |  |  | 1230 | 1.078 | 0.5666 | Yes |
| 15 | PAK1 |  |  | 1237 | 1.074 | 0.5974 | Yes |
| 16 | MBIP |  |  | 1617 | 0.847 | 0.5686 | No |
| 17 | CXCR4 |  |  | 2015 | 0.626 | 0.5309 | No |
| 18 | TPD52L1 |  |  | 2142 | 0.558 | 0.5295 | No |
| 19 | TRIB2 |  |  | 2680 | 0.310 | 0.4626 | No |
| 20 | MAP3K11 |  |  | 3461 | -0.039 | 0.3533 | No |
| 21 | EGF |  |  | 4373 | -0.476 | 0.2383 | No |
| 22 | EPGN |  |  | 4398 | -0.488 | 0.2493 | No |
| 23 | DBNL |  |  | 4486 | -0.528 | 0.2525 | No |
| 24 | MAP3K5 |  |  | 4634 | -0.587 | 0.2490 | No |
| 25 | FGF2 |  |  | 4723 | -0.629 | 0.2551 | No |
| 26 | ERBB2 |  |  | 4844 | -0.690 | 0.2584 | No |
| 27 | GNG3 |  |  | 5227 | -0.876 | 0.2301 | No |
| 28 | PIK3CB |  |  | 5683 | -1.143 | 0.1993 | No |
Table: GSEA details [plain text format]

  

Fig 2: REGULATION\_OF\_MAP\_KINASE\_ACTIVITY: Random ES distribution      
 Gene set null distribution of ES for **REGULATION\_OF\_MAP\_KINASE\_ACTIVITY**
