# Supplementary material for: Network-based integration of molecular and physiological data elucidates regulatory mechanisms underlying adaptation to high-fat diet
Source: Genes Nutr. 2015 May 28;10(4):22. doi: 10.1007/s12263-015-0470-6 (PMC4446272; doi:10.1007/s12263-015-0470-6)
Supplement: Supplementary file 4 — Supplementary material 4 (ZIP 6984 kb) [file 12263_2015_470_MOESM4_ESM.zip › HF LF 5 d GSEA result/RESPONSE_TO_EXTERNAL_STIMULUS.html]

Details for gene set RESPONSE\_TO\_EXTERNAL\_STIMULUS[GSEA]

|  || Dataset | comp\_HF5d-LF5d\_collapsed |
| Phenotype | NoPhenotypeAvailable |
| Upregulated in class | na\_pos |
| GeneSet | RESPONSE\_TO\_EXTERNAL\_STIMULUS |
| Enrichment Score (ES) | 0.4035097 |
| Normalized Enrichment Score (NES) | 1.8959274 |
| Nominal p-value | 0.0 |
| FDR q-value | 0.020237088 |
| FWER p-Value | 0.22 |
Table: GSEA Results Summary

  

Fig 1: Enrichment plot: RESPONSE\_TO\_EXTERNAL\_STIMULUS      
 Profile of the Running ES Score & Positions of GeneSet Members on the Rank Ordered List

  

| PROBE | GENE SYMBOL | GENE\_TITLE | RANK IN GENE LIST | RANK METRIC SCORE | RUNNING ES | CORE ENRICHMENT || 1 | LBP |  |  | 43 | 2.939 | 0.0205 | Yes |
| 2 | CCL8 |  |  | 56 | 2.814 | 0.0444 | Yes |
| 3 | THBD |  |  | 70 | 2.727 | 0.0673 | Yes |
| 4 | LEP |  |  | 74 | 2.711 | 0.0915 | Yes |
| 5 | CCL7 |  |  | 128 | 2.452 | 0.1062 | Yes |
| 6 | FOS |  |  | 207 | 2.218 | 0.1151 | Yes |
| 7 | CCR2 |  |  | 219 | 2.188 | 0.1334 | Yes |
| 8 | CCL5 |  |  | 241 | 2.144 | 0.1499 | Yes |
| 9 | CCR5 |  |  | 244 | 2.139 | 0.1690 | Yes |
| 10 | ALOX5AP |  |  | 273 | 2.083 | 0.1840 | Yes |
| 11 | PF4 |  |  | 290 | 2.044 | 0.2002 | Yes |
| 12 | LTB4R |  |  | 336 | 1.973 | 0.2117 | Yes |
| 13 | C2 |  |  | 340 | 1.966 | 0.2291 | Yes |
| 14 | SPN |  |  | 342 | 1.962 | 0.2468 | Yes |
| 15 | CXCL14 |  |  | 428 | 1.821 | 0.2512 | Yes |
| 16 | PLAT |  |  | 435 | 1.810 | 0.2668 | Yes |
| 17 | GGCX |  |  | 468 | 1.762 | 0.2782 | Yes |
| 18 | TRPV4 |  |  | 472 | 1.756 | 0.2937 | Yes |
| 19 | CCBP2 |  |  | 498 | 1.707 | 0.3056 | Yes |
| 20 | CXCR3 |  |  | 527 | 1.680 | 0.3169 | Yes |
| 21 | AIF1 |  |  | 528 | 1.679 | 0.3321 | Yes |
| 22 | TGFB2 |  |  | 572 | 1.624 | 0.3407 | Yes |
| 23 | TFPI |  |  | 616 | 1.567 | 0.3488 | Yes |
| 24 | PROS1 |  |  | 720 | 1.474 | 0.3475 | Yes |
| 25 | S100A8 |  |  | 817 | 1.384 | 0.3463 | Yes |
| 26 | CCL2 |  |  | 875 | 1.330 | 0.3502 | Yes |
| 27 | TRPC3 |  |  | 960 | 1.264 | 0.3497 | Yes |
| 28 | TNFRSF1A |  |  | 973 | 1.253 | 0.3593 | Yes |
| 29 | CCL17 |  |  | 986 | 1.243 | 0.3689 | Yes |
| 30 | CTGF |  |  | 1033 | 1.207 | 0.3733 | Yes |
| 31 | CCR3 |  |  | 1095 | 1.159 | 0.3751 | Yes |
| 32 | F13A1 |  |  | 1175 | 1.110 | 0.3739 | Yes |
| 33 | DOCK2 |  |  | 1191 | 1.098 | 0.3817 | Yes |
| 34 | PROC |  |  | 1441 | 0.944 | 0.3546 | Yes |
| 35 | CX3CL1 |  |  | 1442 | 0.942 | 0.3632 | Yes |
| 36 | PLA2G2D |  |  | 1474 | 0.920 | 0.3671 | Yes |
| 37 | F10 |  |  | 1477 | 0.918 | 0.3752 | Yes |
| 38 | MEFV |  |  | 1521 | 0.893 | 0.3771 | Yes |
| 39 | RTN4RL1 |  |  | 1571 | 0.869 | 0.3780 | Yes |
| 40 | OGT |  |  | 1579 | 0.865 | 0.3849 | Yes |
| 41 | HDAC4 |  |  | 1621 | 0.844 | 0.3867 | Yes |
| 42 | INHBA |  |  | 1623 | 0.843 | 0.3942 | Yes |
| 43 | AOC3 |  |  | 1640 | 0.835 | 0.3995 | Yes |
| 44 | CCL4 |  |  | 1696 | 0.805 | 0.3989 | Yes |
| 45 | ITGB2 |  |  | 1794 | 0.753 | 0.3919 | Yes |
| 46 | RALBP1 |  |  | 1893 | 0.694 | 0.3842 | Yes |
| 47 | AHSG |  |  | 1908 | 0.685 | 0.3884 | Yes |
| 48 | PLAU |  |  | 1938 | 0.671 | 0.3903 | Yes |
| 49 | F11R |  |  | 1952 | 0.663 | 0.3945 | Yes |
| 50 | RASGRP4 |  |  | 1954 | 0.663 | 0.4004 | Yes |
| 51 | ADM |  |  | 1975 | 0.649 | 0.4034 | Yes |
| 52 | CXCR4 |  |  | 2015 | 0.626 | 0.4035 | Yes |
| 53 | WAS |  |  | 2103 | 0.575 | 0.3963 | No |
| 54 | CXCL13 |  |  | 2207 | 0.528 | 0.3863 | No |
| 55 | ELF3 |  |  | 2261 | 0.503 | 0.3833 | No |
| 56 | STC2 |  |  | 2271 | 0.498 | 0.3866 | No |
| 57 | NFRKB |  |  | 2330 | 0.476 | 0.3826 | No |
| 58 | PLAUR |  |  | 2391 | 0.447 | 0.3781 | No |
| 59 | CXCL9 |  |  | 2452 | 0.418 | 0.3733 | No |
| 60 | CCL11 |  |  | 2459 | 0.413 | 0.3762 | No |
| 61 | VWF |  |  | 2528 | 0.381 | 0.3699 | No |
| 62 | CX3CR1 |  |  | 2643 | 0.326 | 0.3566 | No |
| 63 | AOX1 |  |  | 2693 | 0.304 | 0.3523 | No |
| 64 | CXCL12 |  |  | 2746 | 0.280 | 0.3474 | No |
| 65 | CDH13 |  |  | 2816 | 0.246 | 0.3398 | No |
| 66 | CCL24 |  |  | 2825 | 0.243 | 0.3409 | No |
| 67 | CCRL1 |  |  | 2863 | 0.234 | 0.3377 | No |
| 68 | LMAN1 |  |  | 3100 | 0.114 | 0.3050 | No |
| 69 | F2R |  |  | 3144 | 0.095 | 0.2997 | No |
| 70 | NFATC4 |  |  | 3166 | 0.084 | 0.2974 | No |
| 71 | MMRN1 |  |  | 3185 | 0.078 | 0.2956 | No |
| 72 | RTN4RL2 |  |  | 3267 | 0.040 | 0.2843 | No |
| 73 | RAC1 |  |  | 3275 | 0.037 | 0.2837 | No |
| 74 | CDKN1A |  |  | 3390 | -0.016 | 0.2675 | No |
| 75 | C3AR1 |  |  | 3413 | -0.022 | 0.2646 | No |
| 76 | F8 |  |  | 3441 | -0.033 | 0.2610 | No |
| 77 | PARP4 |  |  | 3757 | -0.185 | 0.2176 | No |
| 78 | ALOX15 |  |  | 4011 | -0.312 | 0.1843 | No |
| 79 | CCL25 |  |  | 4155 | -0.381 | 0.1673 | No |
| 80 | ABCF1 |  |  | 4489 | -0.528 | 0.1244 | No |
| 81 | NFATC3 |  |  | 4518 | -0.541 | 0.1254 | No |
| 82 | CD36 |  |  | 4522 | -0.544 | 0.1299 | No |
| 83 | EIF2AK1 |  |  | 4526 | -0.545 | 0.1344 | No |
| 84 | MAPK1 |  |  | 4676 | -0.606 | 0.1186 | No |
| 85 | INHA |  |  | 4699 | -0.622 | 0.1211 | No |
| 86 | NFX1 |  |  | 4712 | -0.625 | 0.1250 | No |
| 87 | FGF2 |  |  | 4723 | -0.629 | 0.1293 | No |
| 88 | F9 |  |  | 4784 | -0.660 | 0.1267 | No |
| 89 | TGFB1 |  |  | 5288 | -0.910 | 0.0630 | No |
| 90 | RALA |  |  | 5411 | -0.982 | 0.0545 | No |
| 91 | TMPRSS6 |  |  | 5453 | -1.010 | 0.0578 | No |
| 92 | NPY |  |  | 5639 | -1.111 | 0.0414 | No |
| 93 | PIK3CB |  |  | 5683 | -1.143 | 0.0457 | No |
| 94 | MAP2K1 |  |  | 5962 | -1.304 | 0.0177 | No |
| 95 | KLK8 |  |  | 6075 | -1.396 | 0.0144 | No |
| 96 | GP9 |  |  | 6169 | -1.470 | 0.0145 | No |
| 97 | PPARG |  |  | 6525 | -1.855 | -0.0195 | No |
| 98 | ORM2 |  |  | 6678 | -2.093 | -0.0222 | No |
| 99 | ORM1 |  |  | 6710 | -2.152 | -0.0071 | No |
| 100 | CDO1 |  |  | 6998 | -3.263 | -0.0185 | No |
| 101 | FADS1 |  |  | 7018 | -3.490 | 0.0104 | No |
Table: GSEA details [plain text format]

  

Fig 2: RESPONSE\_TO\_EXTERNAL\_STIMULUS: Random ES distribution      
 Gene set null distribution of ES for **RESPONSE\_TO\_EXTERNAL\_STIMULUS**
